# A Self-Driving Lab for Nano- and Advanced Materials Synthesis

**DOI:** 10.1021/acsnano.4c17504

**Published:** 2025-02-25

**Authors:** Mohammad Zaki, Carsten Prinz, Bastian Ruehle

**Affiliations:** † Federal Institute for Materials Research and Testing (BAM), Richard-Willstätter-Strasse 11, D-12489 Berlin, Germany; § Humboldt University Berlin, Unter den Linden 6, D-10117 Berlin, Germany

**Keywords:** Self-driving laboratories, materials acceleration platforms, nanomaterials, advanced materials, automation, robotics, in-line characterization

## Abstract

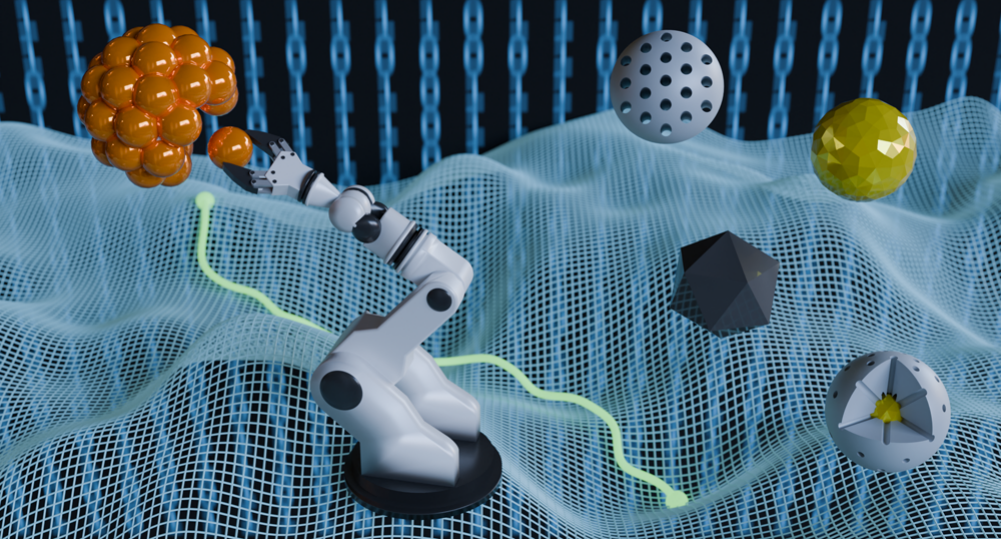

The recent emergence of self-driving laboratories (SDL) and material acceleration platforms (MAPs) demonstrates the ability of these systems to change the way chemistry and material syntheses will be performed in the future. Especially in conjunction with nano- and advanced materials which are generally recognized for their great potential in solving current material science challenges, such systems can make disrupting contributions. Here, we describe in detail MINERVA, an SDL specifically built and designed for the synthesis, purification, and in line characterization of nano- and advanced materials. By fully automating these three process steps for seven different materials from five representative, completely different classes of nano- and advanced materials (metal, metal oxide, silica, metal organic framework, and core–shell particles) that follow different reaction mechanisms, we demonstrate the great versatility and flexibility of the platform. We further study the reproducibility and particle size distributions of these seven representative materials in depth and show the excellent performance of the platform when synthesizing these material classes. Lastly, we discuss the design considerations as well as the hardware and software components that went into building the platform and make all of the components publicly available.

## Introduction

Nano and advanced materials have been recognized as a key enabling technology of the 21st century due to their high potential for contributions to disruptive innovations in new clean energy technologies, substitution of certain critical raw materials, replacement of hazardous substances, improvement of the environmental performance of products and processes, and facilitation of circularity.^1^ This is expressed in the foundation of the Innovative Advanced Materials Initiative and the Advanced Materials Initiative 2030 with over 500 partners from industry and academia, in documents such as the Communication on Advanced Materials for Industrial Leadership,^1^ and the fact that advanced materials are included in the list of ten critical technology areas for the European Union’s economic security.^2^ The importance in science, research, and academia is reflected by the large number of results when searching for publications (>1.8 million results) or journals (>60 results) with the keywords “Nano” or “Advanced Materials” on Clarivate Web of Science. Advanced materials are generally understood as materials that are rationally designed to have new or enhanced properties, and/or targeted or enhanced structural features with the objective of achieving specific or improved functional performance.^3^ This rational and intentional design as well as the improved functional performance make them prime candidates for AI-assisted, accelerated discovery and (closed-loop) optimization in Self-Driving Laboratories (SDLs)^4^ and Material Acceleration Platforms (MAPs),^5^ which will be needed to harvest the full potential of this class of materials.

SDLs and MAPs represent recent advances in the way scientific research is conducted by combining and leveraging the advantages of machine learning, lab automation, and robotics in modular platforms that can help conduct experiments autonomously. Lab automation and robotics can increase the efficiency of discovering and developing new materials through faster experimentation, reduce costs, free up resources by allowing researchers to focus on creative tasks rather than repetitive and laborious manual labor tasks, and increase the reliability and reproducibility of experiments,^6,7^ which is of great interest considering the reproducibility crisis in science.^8^ Incorporating machine learning and artificial intelligence into the process will considerably speed up data processing and interpretation, e.g., by helping to reduce bottlenecks in evaluating characterization data,^9^ and help mapping the vast chemical reaction space more efficiently to arrive at user-defined goals or (pareto) optima faster and more efficiently.^10−12^

There are many impressive examples of state-of-the-art MAPs and SDLs for various tasks in the literature, covering different levels of autonomy.^10,13−25^ At one end of the spectrum of self-driving laboratories are mobile platforms with robot arms that mimic a human chemist and do not require strong modifications to the laboratory equipment or infrastructure. These mobile platforms can navigate between multiple stations with high precision, handle glassware and chemicals, and operate standard laboratory equipment.^10,21^ While these examples are highly advanced and very impressive, they are also very elaborate, expensive, and very difficult to implement for most synthetically focused groups working in standard chemical synthesis laboratories.

Another perhaps more widely used approach to SDLs is the use of stationary robotic arms serving different stationary stations. These MAPs are often specifically built for a certain purpose, such as the production and testing of photovoltaic devices^5^ or the assembly and testing of lithium-ion batteries.^15,19^ Yet another design paradigm for MAPs is the use of liquid reagent transfer backbones or flow setups based on pumps and valves.^13,14,16,18,22−24^ While these “flow-based” MAPs have demonstrated their ability to cover a wide chemical space for synthesis and discovery of materials, most current examples are focused on organic syntheses. There are also examples of flow-based MAPs employing microfluidics for the synthesis of nanoparticles such as quantum dots^14^ and gold nanoparticles.^25^ However, such microfluidic-based systems are usually more explorative in nature. Moreover, most nanoparticle syntheses in the literature are based on batch processes, and their translation to (micro)fluidic setups can be time-consuming and challenging, and the upscaling of the processes is often not trivial. Modular SDLs specifically designed for batched nanomaterial synthesis are also starting to emerge, such as a robotic platform recently published by Dembski et al. for the synthesis of silica nanoparticles.^17^

In this work, we present our “**M**AP for **I**ntelligent **N**anomaterial synthesis **E**nabled by **R**obotics for **V**ersatile **A**pplications”, MINERVA, a modular robotic synthesis platform that can reproducibly synthesize a large variety of nano- and advanced materials and perform their in-line characterization regarding size, surface charge, and optical properties. The system employs standard laboratory hardware (or the corresponding automated counterparts), making the adaptation of literature or in-house procedures for the batch synthesis of nanomaterials straightforward, helping with the upscaling, which is often required for transitioning into higher-tier technology readiness levels (TRLs), and enabling easier collaborative development of syntheses between different groups. The versatility of the platform and the ease of adaptation of synthesis procedures from different fields are demonstrated for six representative nano- and advanced materials, including metal, metal-oxide, silica, metal–organic framework, and core–shell particles synthesized via different reaction mechanisms, including chemical reduction, coprecipitation, sol–gel reaction, coordination chemistry, and seed-mediated growth.

Besides covering a large range of “typical” nano- and advanced material classes and synthesis mechanisms, these examples were chosen due to their high relevance in many areas and current and future applications. Since one striking advantage of an automated robotic synthesis platform is the high reproducibility of the chemical syntheses, such a system is ideally suited for areas requiring consistent and reproducible nanomaterial production, such as applications as sensor materials, in life sciences, or as reference materials. Therefore, gold nanoparticles (NPs) and Stöber silica NPs were chosen for detailed reproducibility studies as reference material candidates. The other nanomaterials cover various other relevant use cases and were also studied for reproducibility. Specifically, those are (i) mesoporous silica NPs that are often used in catalysis and for triggered release applications,^26−28^ (ii) metal organic frameworks (MOFs) for gas storage, catalysis, and sensing,^29−32^ (iii) copper oxide NPs (CuO) for antimicrobial applications,^33,34^ and (iv) core–shell particles, specifically, copper oxide coated with a silica shell (CuO@SiO_2_) and gold coated with a silica shell (Au@SiO_2_).^35,36^

In its current form, our platform can readily carry out chemical syntheses and (postsynthetic) functionalization reactions that involve liquid handling and heating under ambient atmosphere and pressure. With only minor modifications, sample degassing and heating or stirring under an inert atmosphere would also be possible, which could further extend the use of the platform to the synthesis of polymer particles or surface functionalization with reactive chemicals. However, for a rigorous exclusion of oxygen or water that is required for some nano and advanced material syntheses, transferring the system into a glovebox would be required.^5,15^ Solid-state reactions or solvothermal reactions in autoclaves under high pressure can, in principle, be automated as well but are beyond the current scope of the system presented here.

Following the current effort of “democratizing” MAPs, we will also give a detailed description of the design considerations that went into building the MAP, the hardware and software of the system, and make the orchestrating backend MINERVA-OS and all subclasses for communicating with the hardware devices publicly available as open-source software, paving the way for increased adaptation of SDLs either entirely or in part in typical synthetic chemistry laboratories.

## Results and Discussion

### Hardware (MINERVA)

The system we present here is not a single, “turn-key” solution from a specific vendor but rather a custom-made setup consisting of hardware components from different vendors and suppliers. We did this to prevent a “vendor lock-in” and to specifically tailor each component to the special needs and requirements associated with nano- and advanced materials syntheses. The hardware components were carefully selected based on different criteria: Fitness for purpose, availability of appropriate hardware interfaces (USB, serial ports, ethernet ports, etc.), availability of appropriate software interfaces (APIs, SDKs, well-documented instructions that can be sent via serial communication, etc.), cost effectiveness and operating efficiency, and previous experience with hardware components.

#### Robot Arm

The robot arm represents the central component of the platform and serves all other hardware components by moving the required containers with reactants, reagents, solvents, or reaction vessels between them. This contrasts with flow-based SDL setups^13,14,16,18,22−24^ in which the reactors and containers are stationary, and the synthesis solutions are brought to the reactors and distributed among the various hardware components through pumps and tubes. However, it more closely resembles the “traditional” way of performing laboratory work and experimentation, can make the adaptation of existing workflows or literature protocols faster and easier, and provides a high degree of flexibility for running reactions on different scales. The robot arm was chosen based on its hardware specifications (six degrees of freedom, a maximum reach of 700 mm, a repetition accuracy of ±0.1 mm, and a payload of 5 kg, which should be enough to handle all components usually needed for laboratory-scale reactions), its open Python API, the availability of different end effectors and grippers, and its cost-effectiveness.

#### Addition Hardware

The lab scale synthesis of nano- and advanced materials often requires liquid dosing of reactants and reagents over a large range of volumes. Some catalysts or highly specialized or very expensive (bio)molecules are often required on the microliter scale, while others such as solvents are often required on a larger scale of tens to a few hundreds of milliliters. To accommodate these needs, we opted for a combination of different liquid addition hardware. First, there is a pipetting robot with open hardware and open software (Python API) and the ability to handle microliter to milliliter amounts with different single- and multichannel pipettes that use conventional disposable pipet tips, as well as a high flexibility and easy reconfiguration of its deck layout for using small to medium quantities of frequently changing chemicals. The robot arm can also be used to bring new chemicals, reactants, or reagents to the pipetting robot or remove them from its deck and bring them to other hardware components such as hot plates or a centrifuge for further processing. Second, we use a combination of syringe pumps and multiposition selector valves for dispensing larger amounts of commonly used liquids and solvents that do not need to be changed frequently (water, ethanol, toluene, etc.). These are stationary and have to be configured before a reaction (or campaign) is run on the SDL. There is however also a common outlet on one of the ports of the valve that can be used flexibly for transferring the contents of one container to another (e.g., from a reaction flask to a centrifuge tube), to remove liquids from containers (e.g., the supernatant after a centrifugation step), or to move the contents of a container to other characterization hardware outside the reach of the robot arm if that instrument is equipped with a flow cell for in-line characterization and closed-loop optimization (e.g., dynamic light scattering or optical spectroscopy).

#### Heating and Stirring

Almost all chemical syntheses require heating or stirring at some point during the reaction. Here, we used hot plates equipped with magnetic stirrers that have also been used previously in other SDL setups.^13^ We used solid heating blocks with different inlays to accommodate different round-bottom flask sizes. Currently, the appropriately sized heating blocks have to be chosen and placed on the hot plates manually by a user before running a reaction. If different sizes of heating blocks are available on the different hot plates, the software will however automatically identify and use the hot plate with a heating block that is appropriate for the currently used flask size (see software section). There is also the possibility to add chemicals to the flasks at controllable rates while they are heating or stirring on the hot plate through different syringes and/or multiport valves, which is another important requirement for many nano and advanced materials syntheses to control the nucleation and growth rate of the nanomaterials.

#### Centrifugation

Centrifugation is a key step in the synthesis of many nano- and advanced materials especially for their purification prior to further processing. Centrifuges with a high relative centrifugal force are usually required to be able to separate nanomaterials from a synthesis solution and for washing them during purification steps. Generally, fixed-angle rotors are capable of delivering higher centrifugation speeds than swing out rotors, which is why we opted for a centrifuge with a fixed angle rotor that can reach up to >14000*g*. Other important features for the integration of a centrifuge into an SDL are its ability to move the rotor to a well-defined position for loading and unloading with a robot arm before and after each centrifugation run, and having a lid that can be opened and closed remotely through an API. While the latter two features are perhaps not strictly necessary and can in principle also be achieved with careful movements of a robot arm and integrating computer vision for finding the current rotor position, they make the system integration much easier.

#### Sonication

Another piece of hardware that is very important (and also somewhat specific) to nano- and advanced materials synthesis is sonication devices. These are regularly needed to (re)disperse (nano)materials, e.g., after a centrifugation step, before using them as building blocks in a reaction, or before further characterization, e.g., by DLS. The SDL described here features two different types of sonicators, a probe sonicator and a bath sonicator. The probe sonicator has a very high power output at the tip of the sonotrode; however, since the sonotrode is directly submerged in the sample solution, wear of the tip can lead to sample contamination. During bath sonication, the sample is submerged in a water bath; i.e., there is no contamination of the sample solution. However, the sonication power is generally lower than with a probe sonicator, and the outside of the containers is wet after removing them from the sonication bath. In our current setup, the robot arm holds the container in place under the sonotrode during a probe sonication step. Due to the high power, a short duration of typically 15–30 s is enough for dispersing most materials and can help minimizing excessive sonotrode wear. In a bath sonicator, usually longer centrifugation times (15–30 min) are required to disperse the same material, so the robot arm typically places the containers in specially designed holders that hold them tightly in place and prevent them from floating up in the water bath when they are only partially filled. Ultimately, the choice of the appropriate sonication device depends on the material that is being dispersed and considerations such as processing time and the importance of avoiding potential contamination from sonotrode wear.

#### Characterization Hardware

A common challenge with integrating highly specialized hardware (either legacy devices that already exist in a laboratory or even newly procured hardware) can be the lack of readily available or well-documented software interfaces, APIs, or SDKs for controlling this hardware. While we could circumvent this problem for all the synthesis hardware components described above by carefully selecting the most appropriate hardware models and vendors according to the provided hardware and software specifications, we could not find a combined DLS and Zeta measurement device at the time of building our SDL for which the hardware specifications met all our needs, i.e., the precise and flexible characterization of nano- and advanced materials with a high dynamic measurement range while at the same time offering programmatic interfaces that gave full control over data acquisition parameters and allowed remote control of the hardware.

In this case, we had to resort to a different solution, namely, emulating human actions such as keyboard button presses or mouse clicks in software. While this approach in principle allows for automating the interaction with any device via its native control software (proprietary or free) and completely removes the need for direct communication with the hardware via low-level commands, APIs, or SDKs, it is, of course, not very robust. If any unforeseen events occur during the emulated interactions (such as a notification or popup grabbing keyboard or mouse input focus), it can lead to the automation routine sending the inputs to the wrong window, which can result in unforeseen or undesired actions. Moreover, it is very hard to account for unexpected errors that can occur during the measurement with this approach. We took great care to ensure that the correct window is maximized and has input focus before sending any commands, used a dedicated PC for running the measurement software and automation procedure, and read pixel values from the screen to ensure that the hardware is online and ready to measure before attempting the automatic measurement. However, the caveats mentioned above still remain, to some extent. Nevertheless, so far, this approach worked very well for us, and we were able to reliably automate DLS and Zeta potential measurements following this approach. By carefully analyzing the database to which the measurement results are saved, we can also extract all relevant data and metadata when the measurements are complete, upload the results to an electronic lab notebook (see also the next section on software), and use the characterization results as input for ML or AI models in closed loop optimization processes without any human interaction.

For the in-line characterization of optical properties of the materials (such as absorbance, fluorescence, and chemiluminescence), we used a multiwell plate reader that also has a cuvette port that can hold a flow-through cuvette. Here, we encountered another problem with automating measurements: while the device has an API that allows querying the hardware status and starting measurements, all acquisition parameters are stored in separate protocol files and cannot be set via the API. These protocol files follow a proprietary and undocumented file format, for which the device manufacturer could not supply any automatable file editing or conversion software or even just the raw file format specifications. Instead of following the same approach as outlined above for the DLS/Zeta hardware and automating the generation of the protocol files via emulated user inputs in the proprietary software, we carefully studied exemplary protocols manually generated for different acquisition parameters. This way, we were able to write our own Python code for automatically generating similar protocol files in which the most relevant acquisition parameters could be freely specified (measurement mode, well positions, excitation and emission wavelengths, etc.). This gave us the necessary flexibility for incorporating the device in our SDL for the characterization of arbitrary samples and for use in closed-loop processes without having to resort to emulated keyboard or mouse inputs. While this makes the process more robust, the approach is still somewhat vulnerable toward the file format specifications changing in future releases of the proprietary software and also carries some risks associated with parameters not being correctly set or identified. However, in our tests, this method gave us the expected results.

#### Other Hardware Components

In addition to the aforementioned hardware that is readily available from commercial vendors, we found that several other components with small to medium complexity were also crucial or at least very beneficial for the smooth operation of the SDL. These often have very specific requirements regarding their geometry, size, or hardware specifications and can be exceedingly difficult to find from commercial sources at reasonable costs. For this reason, we custom-designed many of these components and built them from 3D-printed parts and basic electronic components, such as DC, stepper, or servo motors. They are controlled by an Arduino board via self-written software that communicates with the software backend (see next section) through a custom communication protocol for easy task scheduling and synchronization by the same backend that also controls the other, commercially available hardware. These custom-built components include a device for capping and uncapping chemical containers and centrifugation tubes with lids of different dimension, thread pitch, and number of turns required for opening and closing, remote-controlled clamps on moving stages that can be attached to a hot plate and raised and lowered, as well as safely hold a flask in place during heating and stirring, and various sensors such as a temperature/humidity sensor, a camera, and an emergency stop button. We also built actuators for the multiport selection valves based on the design described by Steiner et al.^13^ with some modifications to make them work with off-the-shelf electronic components rather than custom-made PCBs and simple serial commands that can be sent to the Arduino via a serial port for controlling valve positions, and used them alongside commercial multiport valve actuators. The Arduino source code as well as the .stl files for the 3D printed components will be made available here, and a more detailed description and assembly instructions for all of these components will be made available in a follow-up publication.

### Software (MINERVA-OS)

**Figure 1:**
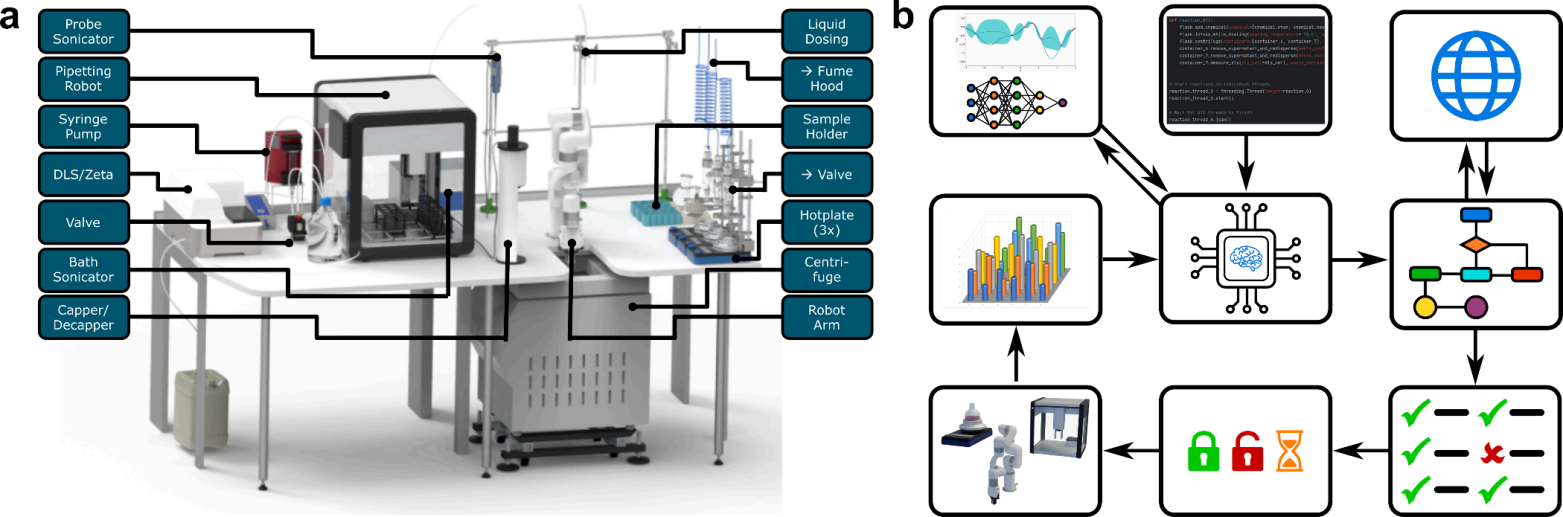
(a) 3D rendering of the SDL platform with the most important hardware components labeled. (b) Schematic representation of a typical workflow. The user supplies high-level synthesis commands as Python code for the API of MINERVA-OS (middle). The backend applies heuristics to determine any values not explicitly specified by the user, potentially looks up missing values for chemicals (such as the density or molar mass) from online resources, performs unit conversions, checks the supplied parameters for integrity and sensibility, determines which hardware will be required in each step, uses the task scheduler to lock/unlock required hardware or enter a waiting queue if the hardware is currently not available, issues the low-level hardware commands to trigger the execution of the high level commands described in the Python script, performs in-line characterization after synthesis and purification of the nanomaterials are complete, and optionally reports the characterization data to a machine learning algorithm to rerun the synthesis with newly suggested, optimized parameters.

Like the hardware, the software backend that orchestrates and drives all the individual hardware components of the platform, provides machine learning feedback when using the platform for optimization tasks in closed-loop processes, and serves as a human-machine interface via an API was developed from scratch for this project. Several design considerations were taken into account when developing the software components and API. There are low-level modules that inherit abstract base classes and take care of hardware integration. These communicate with the hardware directly by sending hardware-specific commands via, e.g., USB, serial ports, or vendor-specific APIs, depending on the automation interfaces exposed by the individual hardware components and devices. However, in general it is easier to not interact with these low-level modules directly, but rather use the high-level API we built on top of these, that also serves as the central orchestrator for scheduling and executing parallel, multithreaded tasks on the hardware and has several built-in heuristics, automations, error-corrections, and sanity-checks that are run before and while executing commands. The API defines a couple of very basic and easy to understand high-level instructions (such as adding a chemical, heating, sonicating, centrifuging, etc.) that are familiar to each chemist. When the user issues such a command, the backend will check the hardware configuration of all connected components, identify which components are needed for the requested action, put these into a priority queue of a task scheduler, subject the user-defined parameters to internal checks to ensure their feasibility and integrity, look up missing values (e.g., molar masses or densities of reactants that are needed for calculating the required amounts of that chemical in an addition step) from publicly available resources such as Wiki data or Pubchem,^37^ convert the units of quantities supplied by the user so that they match each other (e.g., μL into mL or even mol to mL), use heuristics to define the most sensible values for optional parameters that were not provided by the user, and once all checks are passed and all required components are available, start the execution of the requested step (see Figure. 1).

The task scheduler or traffic controller, an abstract class with only static methods that is based on a producer-consumer design scheme operating on a priority queue, is used to parallelize task execution, prevent race conditions and deadlocks, and at the same time keep certain tasks grouped together and ensure that tasks belonging to the same reaction run (mostly) sequentially. For example, if the same reaction requires adding a chemical and a heating step, the task scheduler would ensure that the heating is not done before adding the reactants, just because the hot plate is currently free, but the addition hardware is currently tied up in a different, parallel reaction. At the same time, it would optimize resource use by using the hot plate in a different, parallel reaction that requires it while the first reaction is still waiting for the addition hardware to become available. It also takes care to lock and unlock hardware components and containers to “reserve” them for certain planned steps and ensure that the same resource is not used multiple times concurrently. The grouping, locking, queuing, and synchronization are all organized by TaskGroupSynchronizationObjects handled by the task scheduler (see the github repository for details). Additionally, every newly initialized hardware that inherits from the abstract base class “Hardware” automatically registers with the backend and the task scheduler, ensuring that each available hardware component is known to the orchestrating backend but also that each device is only initialized once.

To facilitate path planning with the robot arm and make the definition of sample positions within sample holders as well as the extension or reconfiguration of the platform with new sample holders easier, sample holder geometry definitions are provided as json files. These definition files include the geometry of the sample holder, the number and arrangement of slots, and what types of containers or samples it can hold (e.g., flasks, centrifuge tubes, etc.). When read in by the backend, the coordinate offsets for finding the individual positions of the sample holder are calculated automatically by the backend based on the orientation of the respective holder on the deck of the platform and the holder geometry and used for path planning with the robot arm. The availability of slots in the holder is also automatically managed by the backend, and “translation” between different coordinate systems of individual hardware components (e.g., the pipetting robot uses a different coordinate system than the robot arm) is automatically taken care of.

During execution, the backend also creates a detailed log file with time stamps and additional information on the (low-level) tasks that are currently executed and offers the possibility to automatically upload the experimental steps, protocols, executed Python scripts, and characterization data including all metadata to an electronic lab notebook (ELN). Here, we used OpenBIS,^38,39^ an open-source ELN-LIMS software with a Python API. An exemplary log file for the synthesis of MSN particles (3 batches synthesized in parallel) as well as a screen shot of the automatically generated ELN entries for the synthesis and in line characterization with DLS can be found in the Supporting Information, Figure S8.

The entire software MINERVA-OS is made publicly available; more details can be found on the accompanying github repository and in the Supporting Information.

### Chemical Syntheses

In this work, we chose different material classes synthesized by different reaction mechanisms and having multiple use cases to highlight the flexibility and versatility of our MAP for the syntheses of nano- and advanced materials.

### Gold-NPs

**Figure 2:**
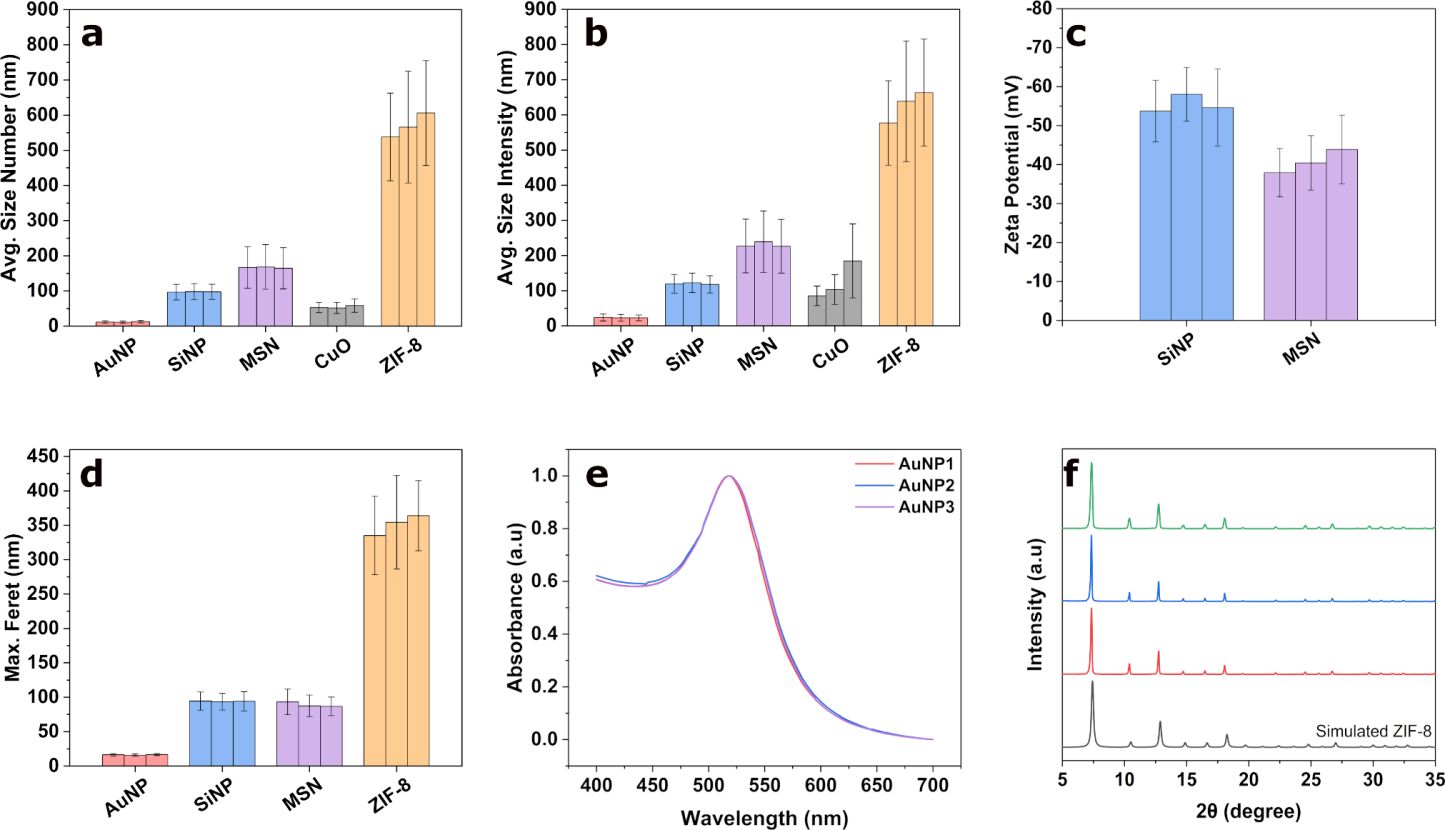
Average size of particle systems compared among three batches. (a) Comparison between mean particle sizes from DLS measurements, weighted by number and (b) weighted by intensity, with the error bars representing the peak width. Differences between Z-average values and volume-weighted mean particle sizes were also similar (Figure S6). (c) Comparison of zeta-potential measurements of three batches of SiNPs and MSN. (d) Mean particle sizes from TEM images for three batches from automatic image segmentation for AuNP and SiNP and manual measurement for MSN and ZIF-8. Manually measured size distributions for AuNP and SiNP were very similar (see Table 1 and Figure S1, Figure S2, and Figure S7). (e) Absorbance spectra of three batches of AuNPs. (f) Comparison of XRD patterns of three ZIF-8 batches against the simulated pattern (COD 4118891). The length of the error bars in a–d represents the peak width for each sample, not the standard deviation between samples, which is much lower (see text).

The reproducibility of the synthesis of Gold NPs (AuNPs) as reference material candidates was investigated. This material class is particularly interesting because of its unique optical properties^40,41^ and the resultant use in bioimaging, biomedical applications and microelectronics. Moreover, AuNPs with suitable surface ligands are highly colloidally stable in water, provide excellent contrast in electron microscopy, and have strong scattering of visible light and X-rays, making them very suitable as reference materials for nanoparticle sizing methods such as scanning or transmission electron microscopy (SEM and TEM), dynamic light scattering (DLS), or small-angle X-ray scattering (SAXS). AuNPs stabilized with citrate were synthesized as described in the Methods/Experimental Section and the reproducibility of the synthesis was assessed by analyzing three different batches (AuNP1, AuNP2, AuNP3). From the DLS measurements, the average intensity weighted (di), volume weighted (dv), and number weighted size distributions (dn) revealed consistently similar hydrodynamic diameters, size distributions, distribution widths (polydispersity index; PI) and *Z*-averages (Figure S1). The mean values and standard deviations were calculated as 23.0 ± 0.7 nm for di, 15.1 ± 0.7 nm for dv, 11.7 ± 0.9 nm for dn, and 19.1 ± 0.9 nm for *Z*-average for the three batches, confirming a very small standard deviation and high reproducibility (see Figure 2(a-b) and Figure S1). Since DLS measurements can be somewhat sensitive to changes in concentration, colloidal stability, and pH, the slightly different intensities and peak positions can most likely be attributed to differences in concentration of the AuNP dispersions or small aggregates present in the nanoparticle dispersion.

**Figure 3:**
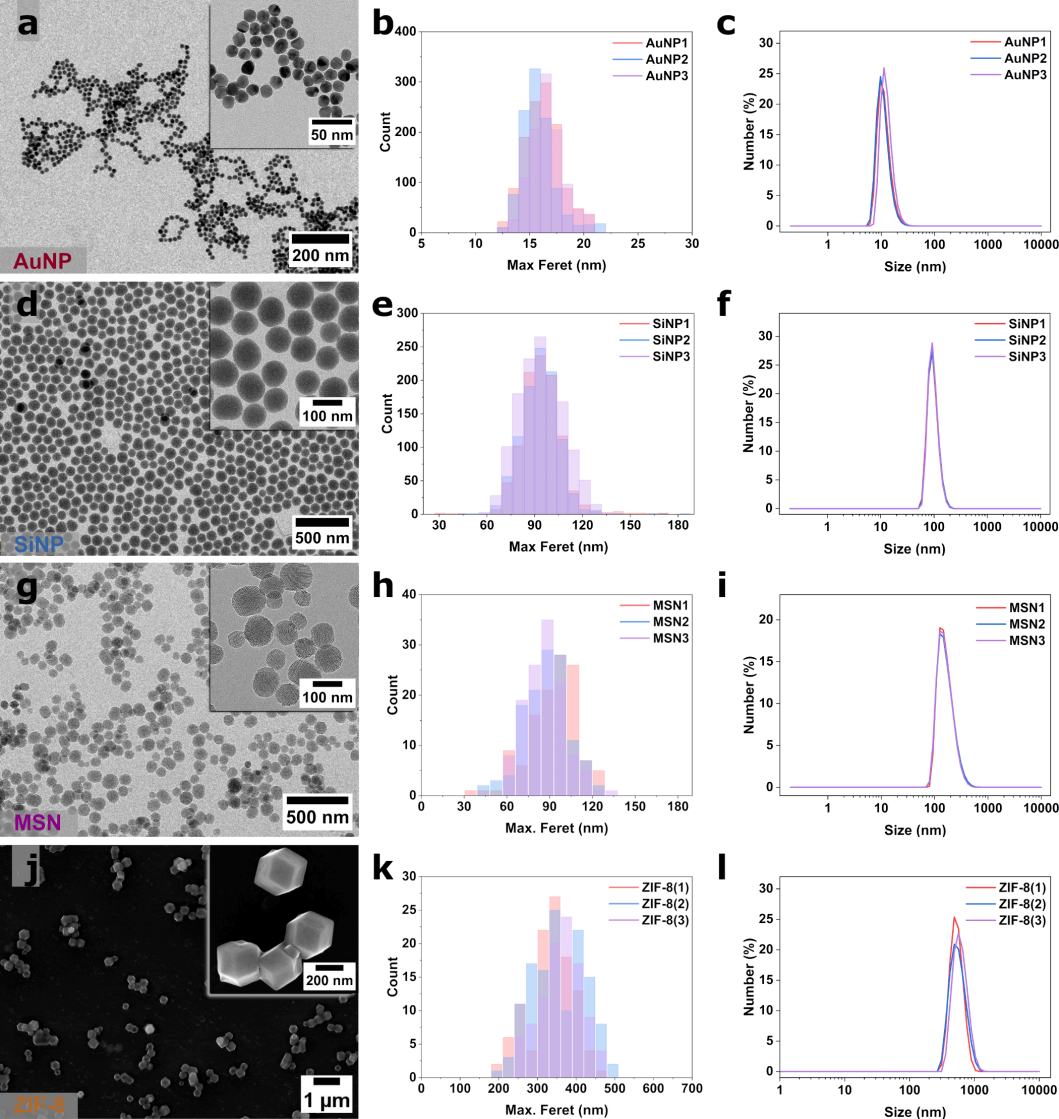
Representative EM images (left column) with comparisons of particle size distributions (max. Feret) from EM image analysis (middle column) and number weighted DLS size distributions (dn) (right column) for various NP systems, namely (a–c) AuNPs, (d–f) SiNPs, (g–i) MSNs, and (j–l) ZIF-8 NPs. The particle size distributions for AuNPs and SiNPs were generated by automatic segmentation.

The analysis of TEM micrographs of the samples showed a monodispersed population of nearly spherical particles (Figure S1). Especially particles that are designed as reference material candidates require the measurement of a very large number of particles to achieve a high statistical significance. Therefore, and for the sake of demonstrating how bottle necks can be removed or at least mitigated when using characterization techniques that cannot be fully automated or used in-line, we investigated the use of automatic image segmentation with the help of an artificial intelligence model. This allows the measurement of >1000 particles within a couple of minutes, speeding up this task by at least 1–2 orders of magnitude compared to manual measurement while generating similar results (see Table 1 and Figure S7). The particle size distributions were very similar across batches (Figure 2d) with both automatic (Figure 3b) segmentation and manual measurement (Figure S1) of particles resulting in similar mean particle sizes, namely, 16.2 ± 0.4 nm from automatic segmentation and 16.5 ± 0.4 nm from manual measurement. Thus, considering all of the size distributions from DLS and TEM (Figure S1), the variation between batches was below 5%. The particle size measured in DLS is usually larger than that measured in TEM, because DLS particle size distributions are intensity-based and also account for the solvent shell around the particles, whereas TEM provides number-based size distributions of the particles. This difference can also be observed here for all batches. However, it is notable that the dv and dn values (Figure S1(f,i)) are smaller than the mean particle size determined from TEM. This can be attributed to the width-error^42^ where a broad size distribution produced by DLS can yield a significantly underestimated dn value when transformed from di. That is evident from Figure S1 where the di distribution is broader for each batch as compared to the distribution that resulted from TEM. Absorbance spectra for the Au NP solutions as depicted in Figure 2e also show excellent reproducibility across the three batches, with surface plasmon resonance (SPR) peak maxima occurring at 518 nm for all batches.

**Table 1:** Mean Particle Sizes for Reference Material Candidate Systems (AuNPs and SiNPs) from Automated Image Segmentation (> 1000 Particles) and Manual Measurements (> 200 Particles)^a^

		size distribution generation (nm)	
nanoparticle system		automatic segmentation	manual measurement
Gold NPs	AuNP1	16.2 ± 1.7	16.6 ± 1.3
	AuNP2	15.8 ± 1.6	16.2 ± 1.2
	AuNP3	16.6 ± 1.5	16.9 ± 1.0
Silica NPs	SiNP1	94.4 ± 13.2	98.0 ± 11.3
	SiNP2	93.4 ± 12.1	94.8 ± 9.8
	SiNP3	94.1 ± 13.9	93.1 ± 8.3

a Size distributions for each batch for both systems are shown in Figure S7.

### Silica-NPs

Another reproducibility study was performed on silica NPs (SiNPs) for their use as reference material candidates. These particles are of great interest due to their well-developed synthesis, tunable particle size, and ease of surface modifications with different functional groups. Furthermore, unfunctionalized SiNPs exhibit excellent colloidal stability in aqueous media and various organic solvents, while appropriate functionalization can also make them colloidally stable in other media such as biological buffers. They also provide good contrast in electron microscopy and good scattering of visible light and X-rays, making them suitable as reference materials for the same characterization techniques that were also discussed for AuNPs. Unfunctionalized silica NPs were synthesized as described in the Methods/Experimental Section to yield three batches (SiNP1, SiNP2, SiNP3) in parallel on the SDL and analysis was run to verify the reproducibility between the batches. The DLS measurements demonstrated an excellent consistency across batches in terms of size distributions for di, dv, dn, and in values of PI, *Z*-average, and zeta potential (Figure S2). The comparison of these values resulted in a mean of 119 ± 2.3 nm for di, 111.1 ± 1.8 nm for dv, 97.3 ± 0.9 for dn, and 115.2 ± 1.6 nm for *Z*-average, which confirm a low standard deviation, as also shown for dn and di in Figure 2(a-b). Zeta potential measurements of aqueous suspensions at pH ∼7 were performed, which all resulted in very similar values with a small standard deviation between the batches, with the mean zeta potential calculated at −55.4 ± 2.3 mV (see Figure 2c). The negative values are typical for unfunctionalized SiNPs due to the presence of deprotonated silanol groups. The slight variation in zeta potential can be attributed to the small changes in pH or different particle environment stemming from the washing steps. The TEM micrographs show a monodispersed population of spherical particles across batches (Figure S2). The size distributions obtained with both automatic segmentation and manual measurement (Figure S7) give remarkably similar mean particle sizes across batches, as summarized in Table 1. The average mean particle size from automatic segmentation was 94.0 ± 0.5 nm, which shows very low variation between batches, as also illustrated in Figure 2d. The mean particle size from manual measurement of particles was also very similar at 95.3 ± 2.5 nm, confirming the reliability of automatic segmentation. As discussed before, the size values measured from DLS for these samples were larger than the values measured from TEM images, as expected. Altogether, we see a very low variation between batches of below 3% in mean particle sizes from all size distributions.

### MSNs

Mesoporous silica NPs (MSN) were also synthesized on the SDL, as they are a versatile nanomaterial with many applications due to their tunable porous structure that enables the loading of ions or other compounds and their subsequent release in a controlled manner. Three syntheses were performed as described in the Methods/Experimental Section in parallel to yield three batches (MSN1, MSN2, MSN3), which were then analyzed for reproducibility. DLS measurements as represented in Figure S3 yield size distributions that are monodispersed and consistent across the batches. Notably, the distributions are not as narrow compared to the systems we studied previously as reference material candidates. This is well-known for MSN synthesis in the literature.^43^ Nevertheless, the mean sizes of the particle size distributions from the three individual batches were in excellent agreement. Mean sizes were recorded at 230.8 ± 7.4 nm for di, 241.7 ± 10.5 nm for dv, 166.3 ± 1.7 nm for dn and 208.6 ± 6 nm for *Z*-average, which confirms the low variation (see Figure 2(a-b) and Figure S3). Zeta potential measured for aqueous suspensions of samples at pH ∼ 7 was negative with a mean value of −40.7 ± 3 mV (see Figure 2c), due to deprotonated silanol groups on the unfunctionalized MSN. The TEM images show a population of spherical, monodispersed particles with a mesoporous structure (see Figure S3), and a slightly broader, but similar size distribution across batches (Figure 3h), as also evidenced from DLS. The mean size distribution calculated from these measurements confirms a good reproducibility of batches with a mean particle size of 89.1 ± 3.6 nm (see Figure 2d). It is worthwhile to mention that the DLS values are larger in this case compared to actual mean particle size from TEM, due to some smaller aggregates or conjoined particles, which can additionally skew the DLS results, apart from the other effects discussed above. As evident from the DLS and TEM results (Figure S3), the standard deviation of mean sizes produced from the characterization results were very low at 5%.

### ZIF-8

Zeolitic imidazolate framework-8 (ZIF-8) is a class of metal organic frameworks (MOFs) that were synthesized as described in the Methods/Experimental Section (ZIF-8(1), ZIF-8(2), ZIF-8(3)) and analyzed for reproducibility. MOFs in general and ZIFs in particular are of great interest due to their simple synthesis and versatile applications.^29,31,32,44^ The DLS measurements were performed with the refractive index for ZIF-8 set to 1.355^45^ which gave monodispersed and similar size distributions across all three batches as represented in Figure S4. Mean sizes and standard deviation across three batches were measured at 592.5 ± 40.6 nm for di, 629 ± 50.9 nm for dv, 543.6 ± 19.9 nm for dn, and 648.1 ± 19.7 nm for Z-average, which shows a low variability between batches (see Figure 2(a-b), Figure S4). Further, the morphology of the ZIF-8 samples was analyzed by scanning electron microscopy (SEM) and transmission-mode scanning electron microscopy (tSEM) (Figure S4). The images show crystalline, well dispersed particles with clearly visible crystal facets. The particle size distribution obtained from the images was consistent across the three batches (Figure 3k). The mean particle size for the three batches was measured as 351.1 ± 14.7 nm, which shows a low standard deviation between batches, as also represented in Figure 2d. XRD patterns were recorded for the three batches to confirm their crystallinity, as illustrated in Figure 2f. The diffraction patterns show characteristic peaks around 2θ = 7.3°, 10.38°, 12.74°, 14.88°, 16.65°, and 18.2° which correspond well with the diffraction patterns reported in the literature, closely matching the simulated diffraction pattern from a Crystallographic Information File (COD 4118891) confirming a ZIF-8 structure with a sodalite (SOD) crystal phase.^29^ Interestingly, the peak shape is somewhat asymmetric, and there are small shifts in the peak positions, which will be further investigated in the future.

### CuO

**Figure 4:**
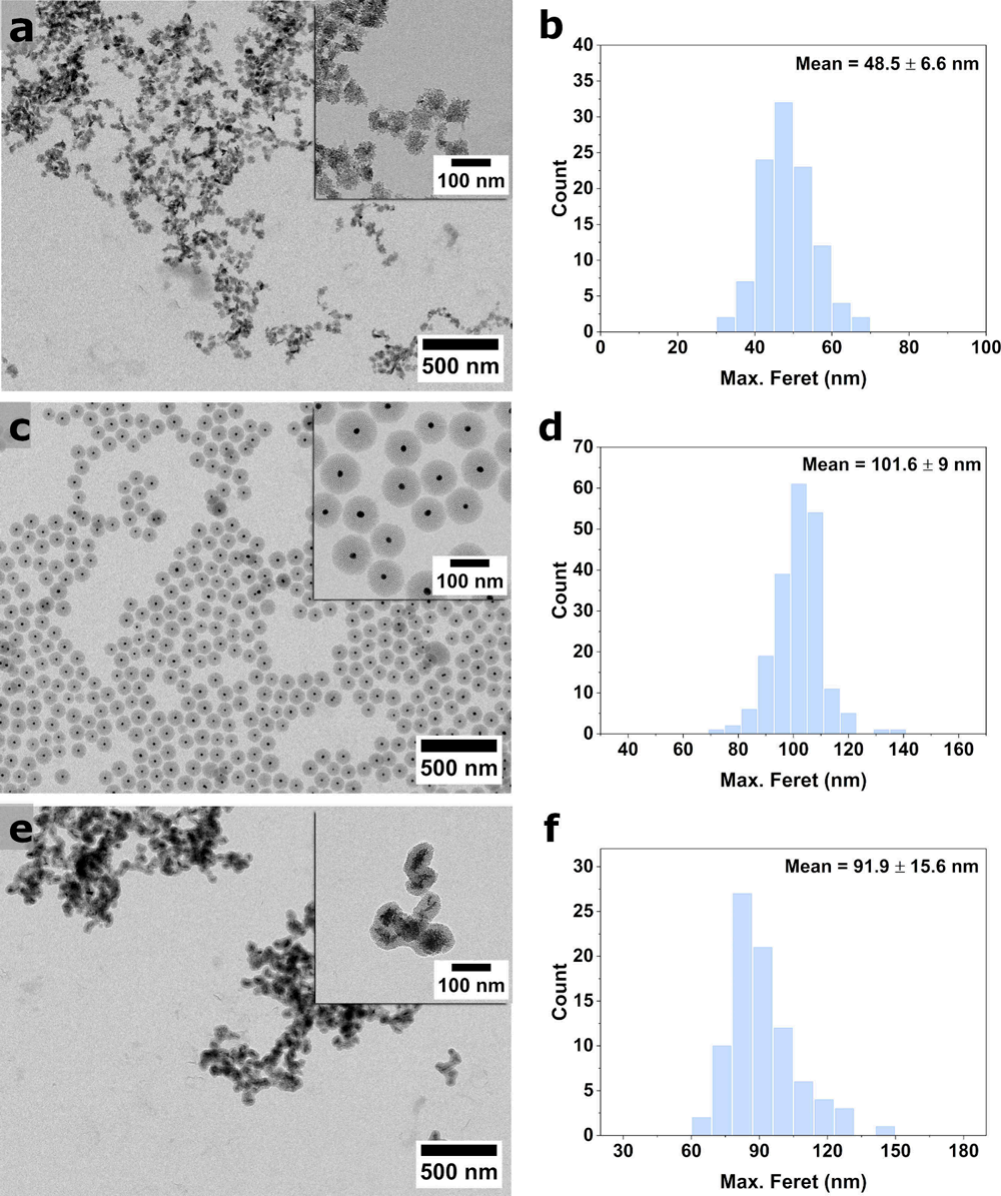
Representative TEM images with particle size distribution for (a, b) CuO NPs and core–shell particles: (c, d) Au@SiO_2_ system. (e, f) CuO@SiO_2_ system.

Copper oxide NPs (CuO NPs) are another important set of NPs with various applications, e.g., as antimicrobial coatings.^33,34^ CuO NPs were synthesized as described in the experimental section, in three parallel reactions (CuO1, CuO2, and CuO3), and then analyzed for reproducibility. The DLS measurements show monodispersed and similar size distributions across batches with very good agreement in average values of di, dv, and dn and *Z*-average (Figure S5). The mean value and standard deviations were recorded at 124.4 ± 52.9 nm for di, 74.6 ± 12.4 nm for dv, 54.2 ± 3.6 nm for dn, and 102.3 ± 34.1 for *Z*-average (see Figure 2(a-b), Figure S5). Notably the intensity-based values, di- and *Z*-average, did not agree well between the batches, presumably because the samples show clusters of smaller particles as seen from the TEM images of one of the batches (Figure 4a), with aggregates potentially influencing the intensity-based values. Nonetheless, dn values were found very similar to the cluster size as measured from TEM (Figure 4b) with the mean size measured at 48.5 ± 6.6 nm, thus establishing a good reproducibility of the synthesis and the possibility of reaction control from the dn values even in the case of suboptimal dispersion of particles.

### Core–Shell Nanoparticles

The ability of the system to synthesize even more complex structures, comprising multiple reaction steps that involve using purified products of one reaction as a precursor for the next reaction, was assessed for core–shell nanoparticle syntheses. Au@SiO_2_ nanoparticles were synthesized as described in the Methods/Experimental Section. Since the size distributions of AuNPs as described above were extremely similar for different batches, we were able to combine several batches and use the resulting dispersion as the cores. This procedure can help to avoid the need to scale up the reaction. The synthesized core–shell particles were analyzed by TEM (see Figure 4(c)), which showed a monodisperse population of spherical particles, very well spread out on the grid, with a thick mesoporous silica shell and a small gold core. The size distribution recorded from the images, represented in Figure 4d, resulted in a mean particle size of 101.7 ± 8.7 nm. The extremely homogeneous population of Au@SiO_2_ particles is further indicative of the high reproducibility of synthesis with the SDL which allows for combining of reaction products.

CuO@SiO_2_ was also synthesized by the SDL in a similar manner, as described in the Experimental Section. The synthesis used the products of the CuO NPs synthesis described before, again after combining several batches. The resulting particles were analyzed by TEM (see Figure 4(e)) and show CuO cores with a thin mesoporous shell. The size distribution recorded from the images, represented in Figure 4f gave a mean particle size of 91.9 ± 15.6 nm.

## Conclusions

We demonstrated that the SDL presented here can be used for synthesizing various nano- and advanced materials with a large variety of synthesis chemistries, i.e., precursor reduction for the synthesis of AuNPs, sol–gel synthesis for silica NPs and MSN, coordination chemistry for the MOF ZIF-8, coprecipitation for CuO, and seed-mediated growth for Au@SiO_2_ and CuO@SiO_2_ core–shell nanoparticles. We further showed the fully automatic synthesis, purification, and in-line characterization of these materials with DLS and absorbance measurements.

The mean particle sizes as well as the particle size distributions from DLS and electron microscopy measurements for the seven particle systems from different material classes were investigated, and they all showed very low batch-to-batch variation, demonstrating the excellent reproducibility of these syntheses when they are automated on our platform. This means that tedious and laborious “standard syntheses” of simple and composite (core–shell) structures can readily and reliably be automated, ensuring high reproducibility and freeing up human resources for other, more creative and stimulating tasks. Furthermore, the very good monodispersity and high degree of reproducibility for gold and silica nanoparticles enable on-demand synthesis of these materials for their potential use as reference materials, which we will further investigate in the future.

The flexibility of the SDL in terms of reaction volume and reaction scale was demonstrated as well, with reaction scales ranging from sub 10 mL to a few hundreds of milliliters. The high homogeneity across batches also allowed us to mix different particle batches and use the resulting mixture for further functionalization or analysis, which can be highly beneficial when scaling up a synthesis proves to be difficult, which is often the case for nanomaterials.

The hardware that was used in the SDL was described in detail, and selection criteria, limitations, and possible workarounds were discussed. We also describe the software backend MINERVA-OS and make all hardware and software components developed for this project publicly available. This includes components such as the Task Scheduler that is used by the backend for locking and unlocking hardware and queueing, grouping, and synchronizing multithreaded tasks, software modules for low-level hardware communication with different synthesis and characterization devices, methods for converting units and looking up chemical information from online resources such as Wikidata and Pubchem, and modules for uploading relevant synthesis and characterization data and metadata to an electronic lab notebook. The considerations for hardware components described in the text and the provided software tools and modules can greatly benefit other groups working on implementing and automating similar synthesis workflows in the future.

Next, we will work on integrating simulation components into the material design phase of the process, develop a graphical user interface for enhanced user-friendliness of the software, and use different machine learning algorithms for closed-loop optimization of nano- and advanced materials. Another currently ongoing effort focuses on integrating solid dosing into the system, which can be a challenge, especially with reactive or hygroscopic chemicals.

## Methods/Experimental Section

### Synthesis Hardware

#### Robot Arm

The synthesis workflow is primarily facilitated by the xArm6 robotic arm (UFactory), equipped with an xArm gripper bracket as the end effector.

#### Chemical addition

For the precise addition of small amounts of liquid reactants, the workflow utilizes an OT-2 pipetting robot (Opentrons). We used P20 GEN2 (1 μL–20 μL), P300 GEN2 (20 μL–300 μL), and P1000 GEN2 (100 μL–1000 μL) single-channel pipettes based on the desired volume ranges. Transfer of larger volumes of liquid is managed by low pressure multiposition selector valves, Cheminert C25 (10 positions, Vici) and Idex V-340 (6 positions, Idex Health&Science) paired with Vici universal actuators (Vici) and in-house built actuators, respectively.^13^ The valves operate in conjunction with Aladdin AL-1050 high flow syringe pumps or AL-1010 high pressure syringe pumps (WPI), based on the requirements.

#### Heating and stirring

Heating and stirring was carried out on RCT 5 digital hot plates (IKA) coupled with PT-100 temperature probes and equipped with exchangeable solid heating blocks with different inlays suitable for flasks with a volume ranging from 10 to 250 mL. For cooling down the reaction flask after the reaction, axial fans (Sunon, 12 V DC) were installed next to each hot plate.

#### Purification

A temperature-controlled RobotCen (Herolab) centrifuge amenable to automation with precise positioning of the rotor was used for centrifugation with an AF 8.50.3 rotor with a maximum capacity of 8 × 50 mL. For subsequent sonication and particle redispersion, a probe sonicator UP200 St (Hielscher, 200W, 26 kHz) and a bath sonicator Sonorex Digitec DT 255 H-RC (Bandelin) were used.

### Characterization Hardware

#### Dynamic Light Scattering (DLS) and Electrophoretic Light Scattering (ELS)

The DLS and ELS measurements were conducted on a Zetasizer Ultra Red (Malvern Panalytical) instrument to determine the hydrodynamic diameter and the Zeta potential of the particles, respectively. A dispersion of nanoparticles in Milli-Q water was introduced into a 1 mL Malvern DTS 1070 folded capillary cell by using a system of syringe pumps and valves. Three sets of both DLS and Zeta potential measurements were performed.

#### Absorbance Measurement (Abs)

The absorbance spectra of the particles were recorded using a SpectraMax M3 reader (Molecular Devices) using disposable polystyrene cuvettes (DTS0012, Malvern). Two mL of the dispersed nanoparticles in Milli-Q water were introduced into the cuvette and the measurement was run. A blank spectrum was also recorded each time for background correction.

#### Transmission Electron Microscopy (TEM)

The TEM images of the samples were recorded on a Talos F200S Microscope (ThermoFisher Scientific) at 200 kV. The samples were prepared by drop casting 10 μL of a nanoparticle suspension onto carbon-coated copper grids and drying at room temperature. For gold nanoparticles, the grids were subjected to plasma cleaning with a gas mixture of 25% oxygen and 75% argon for 8 s prior the drop casting to improve the wettability of the grids and achieve better dispersion of gold nanoparticles on the grids when drop-casting from aqueous suspensions. The obtained electron microscopy images were then analyzed by manually measuring the maximum Feret diameters of the particles from at least 3 different images with the software ImageJ. For the reference material candidates Au NP and SiO_2_ NP, automatic image segmentation with a convolutional neural network^9^ was performed for at least 1000 particles additionally, and the results are compared with manual measurements of at least 200 particles. For all other particle systems, at least 120 particles from at least 3 different images were measured manually.

#### Scanning Electron Microscopy (SEM)

The SEM images were obtained on a Supra 40 Microscope (Carl Zeiss AG) at 10 kV, with the images recorded in both SEM mode and transmission-mode (tSEM). The sample preparation and analysis were performed as described for TEM. The particle size measurements here were taken from tSEM images.

#### Powder X-ray Diffraction Analysis (XRD)

The XRD spectra were recorded on a D8 Advance A25-X1–1 Diffractometer (Bruker) with Cu Kα radiation (1.541874 Å).

### Chemicals

Tetraethyl orthosilicate (TEOS, ≥ 99%, Sigma-Aldrich), hexadecyltrimethylammonium bromide (CTAB, ≥ 99%, ROTH), sodium hydroxide (NaOH, 1 M, ChemSolute), Ethanol (Absolute, ≥ 99.9%, ChemSolute), ethanol (Denatured, ≥ 99.8%, ChemSolute), ammonium nitrate (≥98%, Chemlab), ammonia solution (25%, ChemSolute), Nile blue chloride (ROTH), l-lysine monohydrate (≥98.5%, ROTH), copper(II) acetate monohydrate (≥98.0%, ThermoScientific), acetic acid (AcOH, ≥ 99.8%, ChemSolute), l-arginine (>98.5%, Sigma-Aldrich), gold(III) chloride trihydrate (HAuCl_4_, ≥ 99.9%, Sigma-Aldrich), sodium citrate tribasic dihydrate (≥99%, Sigma-Aldrich), 2-methylimidazole (≥98%, ROTH), zinc nitrate hexahydrate (98%, Sigma-Aldrich), and methanol (99.8%, ChemSolute) were used as received. Milli-Q water (Merck) was used throughout this study.

### General Synthesis Procedures

The synthesis procedures from the literature were directly adapted to the automated synthesis workflow with slight modifications, mostly for adjusting the reaction scale. All solid chemicals were added from stock solutions at the indicated concentrations, and addition during stirring was performed by using PTFE tubes connected to valves and syringe pumps. When heating was applied, the flasks were cooled by using fans after the reactions were finished. The python code that was run for each synthesis can be found in the Supporting Information (SI) and on github.

*Gold nanoparticles (AuNPs)* were synthesized following a procedure described by Niu et al.^46^ with slight modifications. An aqueous solution of 10 mL of 2.5 × 10^–4^ M HAuCl_4_ was heated under stirring at 750 rpm to a temperature set point of 95 °C for the heating blocks which results in an internal temperature of the synthesis solution of approximately 75 °C. After temperature stabilization for 5 min, 350 μL of an aqueous sodium citrate solution (10 mg/mL) were added at a rate of 10 mL/min. The reaction mixture changed to a wine-red color within several minutes, indicating gold NP formation, which were ca. 16 nm in diameter and had an estimated concentration of approximately 9 × 10^11^ NPs/mL, assuming all HAuCl_4_ was converted to NPs. The solution was cooled, and particles were purified by centrifugation (3500 rcf, 90 min, 5 °C), then ultrasonically redispersed (5 s) in 10 mL of Milli-Q water.

*Silica nanoparticles* were synthesized by adapting the Stöber procedure.^47^ An ethanolic mixture of ammonium hydroxide comprising 6.67 mL of ethanol, 167 μL of Milli-Q water, and 2 mL of aqueous ammonia solution (25 wt %) was stirred at 25 °C and 300 rpm. 4.5 mmol of TEOS in 26.7 mL of ethanol was added at a rate of 30 mL/min, resulting in a white suspension that was maintained under stirring for 24 h. The particles were then purified by three cycles of centrifugation (14k rcf, 15 min, 25 °C) and ultrasonic redispersion (90 s), once with water (40 mL) and twice with ethanol (40 mL).

*Mesoporous silica nanoparticles (MSNs)* were synthesized following a published procedure with slight modifications.^26^ A mixture was prepared by combining 50 mL of Milli-Q water, 120 mg of CTAB in 6 mL of water, and 720 μL of 1 M NaOH, which was then heated to a temperature set point of 94 °C for the heating blocks (resulting in an internal temperature of the synthesis solution of ca. 80 °C) under stirring at 300 rpm. After temperature stabilization for 30 min, the stirring speed was increased to 820 rpm and 2.7 mmol of TEOS in 1 mL of ethanol were added at a rate of 2 mL/min, while the stirring and heating were continued for 2 h. The particles were then cooled and washed by two cycles of centrifugation (14 000 rcf, 20 min, 25 °C) and ultrasonic redispersion (90 s) in ethanol (40 mL).

To extract the template, the particles were redispersed in 54 mL of ethanolic ammonium nitrate solution (1 g/50 mL) and heated to a temperature set point of 72 °C for the heating blocks (internal temperature ca. 57 °C) under stirring at 300 rpm for 1.5 h. Following that, they were washed twice with ethanol (40 mL), as described above.

ZIF-8 was synthesized following a procedure described by Ostad et al.^29^ A solution containing 820 mg of 2-methylimidazole (0.01 mol) in 25 mL of methanol was added into a solution of 742.5 mg of zinc nitrate hexahydrate in 25 mL of methanol at a rate of 18 mL/min under stirring at 500 rpm and 25 °C, and the stirring was continued for 24 h. The particles were then purified by 3 cycles of centrifugation (9k rcf, 15 min, 25 °C) and ultrasonic redispersion (25 s) in methanol (30 mL), followed by drying at 55 °C for 12 h.

*Copper oxide nanoparticles (CuO)* were synthesized as described by Zhu et al. with slight modifications.^48^ A mixture was prepared by combining 40.1 mL of water, 3.2 mL of copper acetate monohydrate (0.25 M, aq.), and 66.7 μL of glacial acetic acid and heating to a temperature set point of 95 °C for the heating block (resulting in an internal temperature of ca. 80 °C) under stirring at 700 rpm. After temperature stabilization for 5 min, 2.67 mL of NaOH (1M, aq.) was added at a rate of 1 mL/min, while the heating and stirring were continued for 20 min. The particles were then cooled and purified by 2 cycles of centrifugation (14k rcf, 10 min, 25 °C) and ultrasonic redispersion (20 s) in water (20 mL).

*Core–shell nanoparticles* were synthesized by adapting the procedure described by Cricq et al. to CuO and Au cores.^49^ For the synthesis of gold nanoparticles coated with a mesoporous silica shell (Au@SiO_2_), aqueous solutions of 5.6 mg of CTAB in 0.93 mL of water, 2 mg of l-arginine in 200 μL of water, and 0.5 mL of 1× washed and 9× concentrated gold core nanoparticles in water (∼4 × 10^12^ particles based on estimated NP concentration from AuNP synthesis reaction) were added to 2.17 mL of Milli-Q water. After brief sonication (20 s), the solution was heated to a temperature set point of 100 °C for the heating blocks (resulting in an internal temperature of ca. 80 °C) with stirring at 590 rpm for 30 min. Subsequently, 90 μmol of TEOS in 100 μL of ethanol were added at a rate of 0.8 mL/min, while the heating and stirring were continued for another 3 h. The particles were then cooled and washed by three cycles of centrifugation (14k rcf, 15 min, 25 °C) and ultrasonic redispersion (30 s), once with water (5 mL) and twice with ethanol (5 mL).

To extract the template, the particles were redispersed in 3 mL of ethanolic ammonium nitrate solution (1 g/50 mL) and heated to a temperature set point of 72 °C for the heating blocks (internal temperature ca. 57 °C), under stirring at 300 rpm for 1.5 h. Following that, they were washed twice with ethanol (5 mL) as described above.

For the synthesis of copper oxide nanoparticles coated with mesoporous silica shells (CuO@SiO_2_), aqueous solutions of 37.5 mg of CTAB in 8.90 mL of water, 10 mg of l-arginine in 1 mL of water and 0.5 mL of CuO core nanoparticles in water (∼1.99 × 10^13^ particles, calculated based on 45 nm particle size, 12 mg/mL particle amount, and a CuO density of 6.31 g/cm^3^) were added to 4.5 mL of Milli-Q water. After brief sonication (20 s), the resulting solution was heated to a temperature set point of 95 °C for the heating blocks (resulting in an internal temperature of ca. 80 °C) with stirring at 400 rpm for 30 min. Subsequently, 100 μL of TEOS were added at a rate of 5 mL/min, while the heating and stirring were continued for another 3 h. The particles were then cooled and washed by three cycles of centrifugation (14 000 rcf, 15 min, 25 °C) and ultrasonic redispersion (30 s), once with water (12 mL) and twice with ethanol (12 mL).

To extract the template, the particles were redispersed in 12 mL of an ethanolic ammonium nitrate solution (1 g/50 mL) and heated to a temperature set point of 72 °C for the heating blocks (internal temperature ca. 57 °C), under stirring at 300 rpm for 1.5 h. Following that, they were washed twice with ethanol (12 mL), as described above.

## Supporting Information Available

Additional figures, individual synthesis scripts of all material classes that were discussed in the manuscript, and an exemplary log file (PDF)

## Notes

A preprint of this article has been published on *ChemRxiv*.^50^
