# Supplementary material for: A Self-Driving Lab for Nano- and Advanced Materials Synthesis
Source: ACS Nano. 2025 Feb 25;19(9):9029–41. doi: 10.1021/acsnano.4c17504 (PMC11912568; doi:10.1021/acsnano.4c17504)
Supplement: Supplementary file 1 — nn4c17504_si_001.pdf [file nn4c17504_si_001.pdf]

Supplementary Information

A Self-Driving Lab for Nano- and Advanced Materials Synthesis

Mohammad Zaki[†,§,‡], Carsten Prinz[†], Bastian Ruehle*[,†,‡]

[†]Federal Institute for Materials Research and Testing (BAM), Richard-Willstätter-Strasse 11, D-12489 Berlin, Germany

[§]Humboldt University Berlin, Unter den Linden 6, D-10117 Berlin, Germany

[‡]These authors contributed equally

*Correspondence: bastian.ruehle@bam.de

1. Further Resources:

The github repository accompanying this publication can be found here:

https://github.com/BAMresearch/MAPz_at_BAM/tree/main/Minerva

It contains all the Python code and modules discussed in the text, as well as more extensive feature descriptions, documentations, a video showcasing the MAP and some exemplary synthesis, purification, and characterization steps, and the Arduino code and stl files of the custom-built hardware. It also contains all the Python scripts that were executed for synthesizing the materials discussed in the publication (see also section 4. of the SI) and an exemplary detailed log file (see also section 5. of the SI).

2. Experimental:

To showcase the batch-to-batch consistency of syntheses, EM microscopy images of each batch with the size distribution (Max. Feret) from EM microscopy and size distributions from DLS measurements weighted by intensity, volume and number were measured and are shown below.

AuNP:

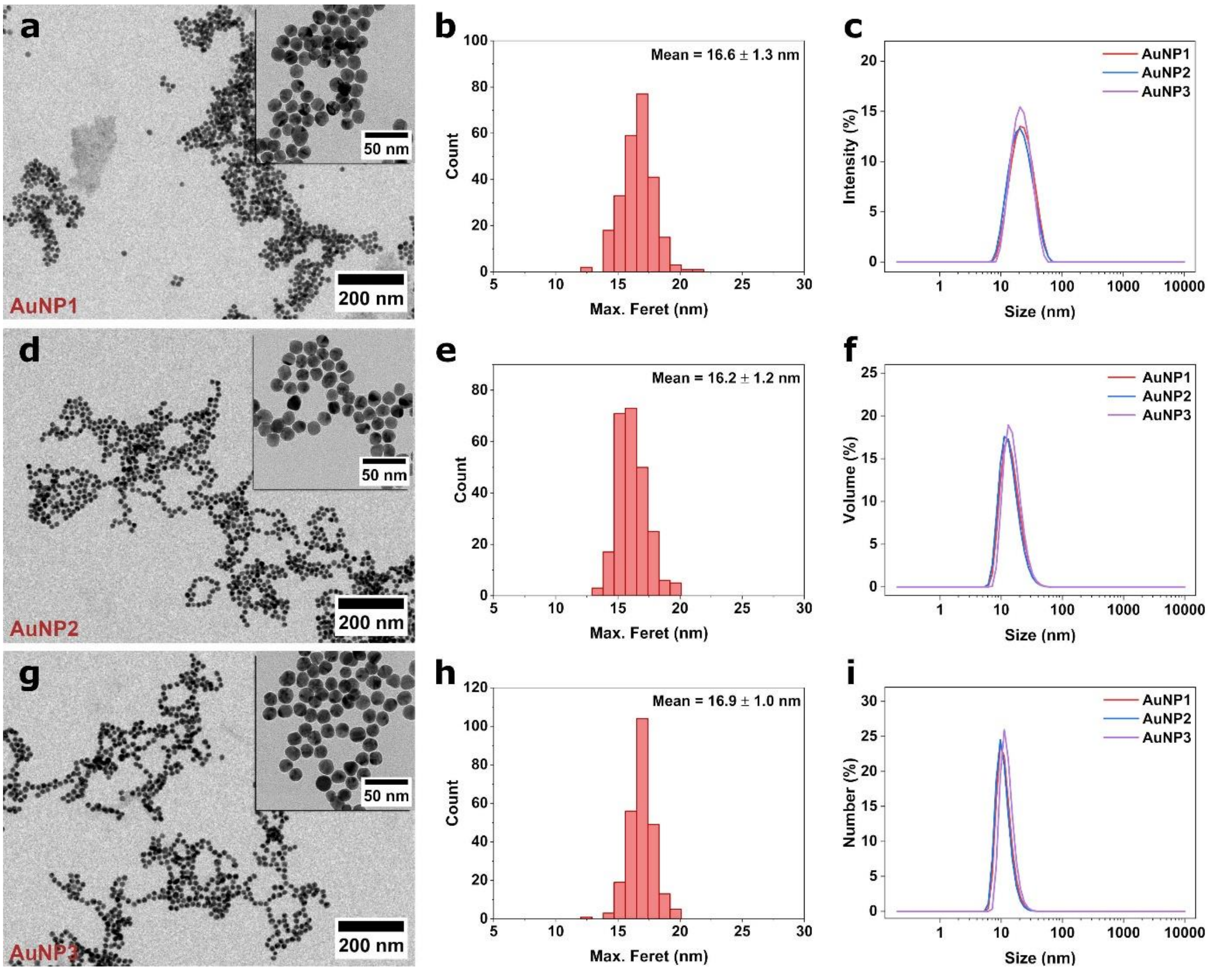

Fig. S1. Representative TEM images of three batches of AuNP system, with size distributions (Max. Feret) resulting from manual particle measurement from TEM images along with accumulated DLS results in the right column. TEM images and respective size distributions for a,b) AuNP1; d,e) AuNP2; g,h) AuNP3 are depicted. c,f,i) Accumulated di, dv and dn results respectively for the three batches.

SiNP:

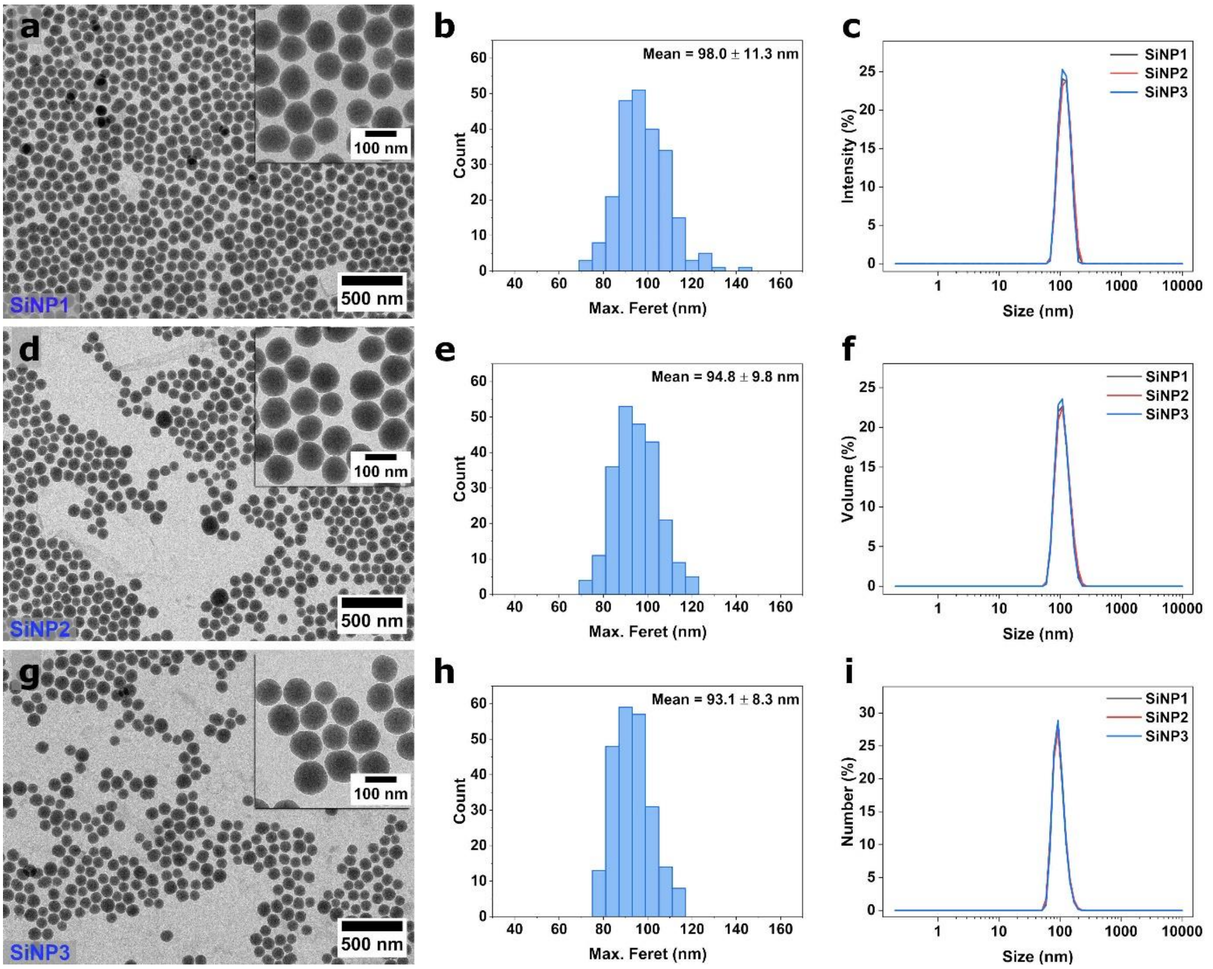

Fig. S2. Representative TEM images of three batches of SiNP system, with size distributions (Max. Feret) resulting from manual particle measurement from TEM images along with accumulated DLS results in the right column. TEM images and respective size distributions for a,b) SiNP1; d,e) SiNP2; g,h) SiNP3 are depicted. c,f,i) Accumulated di, dv and dn results respectively for the three batches.

MSN:

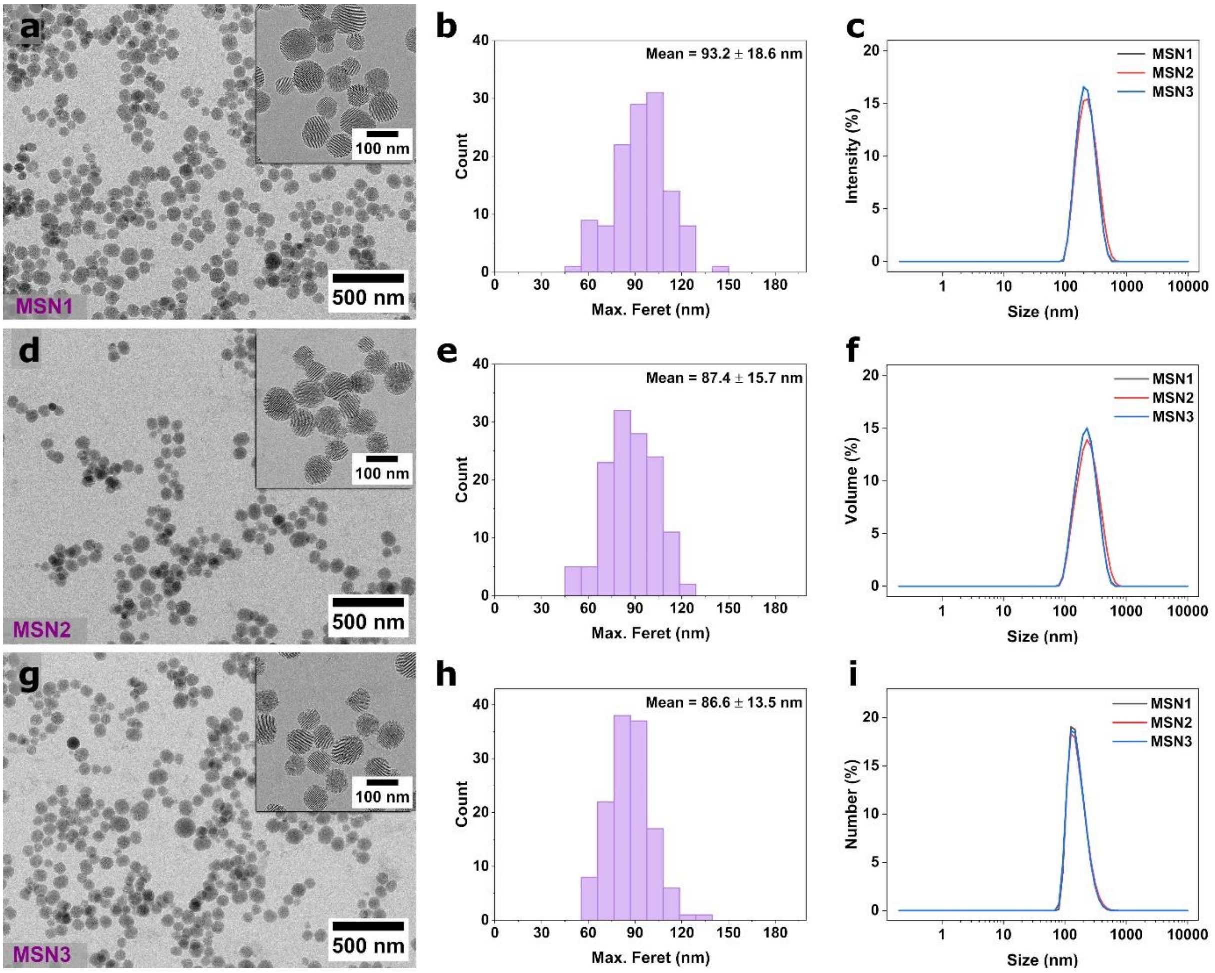

Fig. S3. Representative TEM images of three batches of MSN system, with size distributions (Max. Feret) resulting from manual particle measurement from TEM images along with accumulated DLS results in the right column. TEM images and respective size distributions for a,b) MSN1; d,e) MSN2; g,h) MSN3 are depicted. c,f,i) Accumulated di, dv and dn results respectively for the three batches.

ZIF-8 particles:

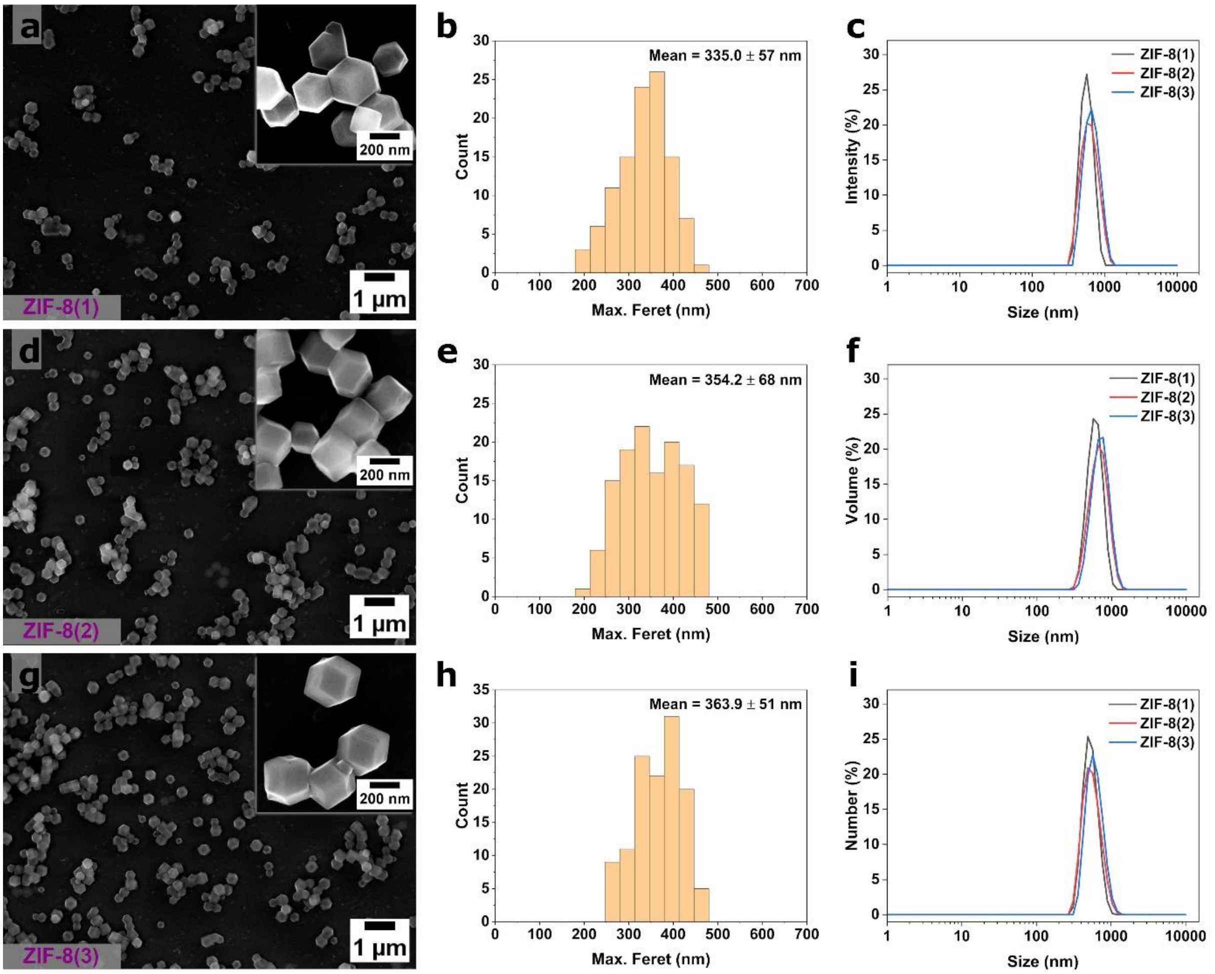

Fig. S4. Representative SEM images of three batches of ZIF-8 system, with size distributions (Max. Feret) from tSEM images along with accumulated DLS results in the right column. SEM images and respective size distributions for a,b) ZIF-8(1); d,e) ZIF-8(2); g,h) ZIF-8(3) are depicted. c,f,i) Accumulated di, dv and dn results respectively for the three batches

CuO NP:

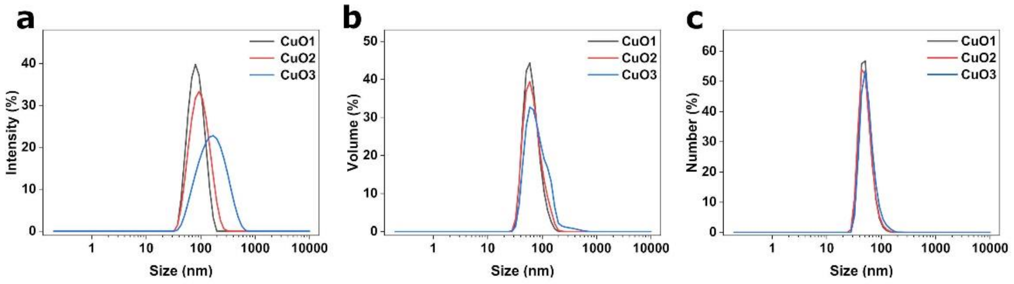

Fig. S5. Comparison of average size distributions from DLS measurements for CuO system weighted by a) intensity, b) volume and c) number.

Batch to batch variation dv and Z-average from DLS measurements:

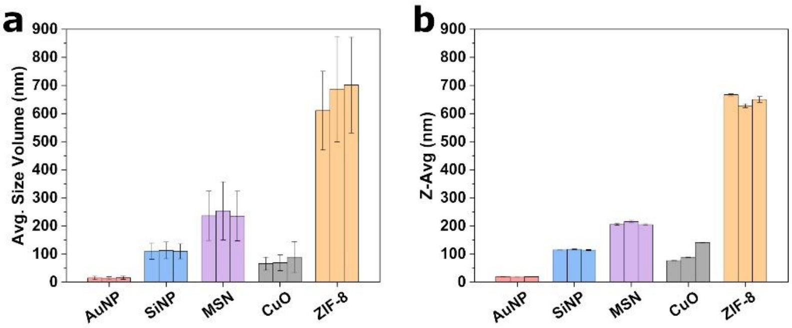

Fig. S6. Batch to batch comparison of three batches of all particle systems based on, a) Volume weighted mean particle size. b) Z-average values

Automated EM image analysis:

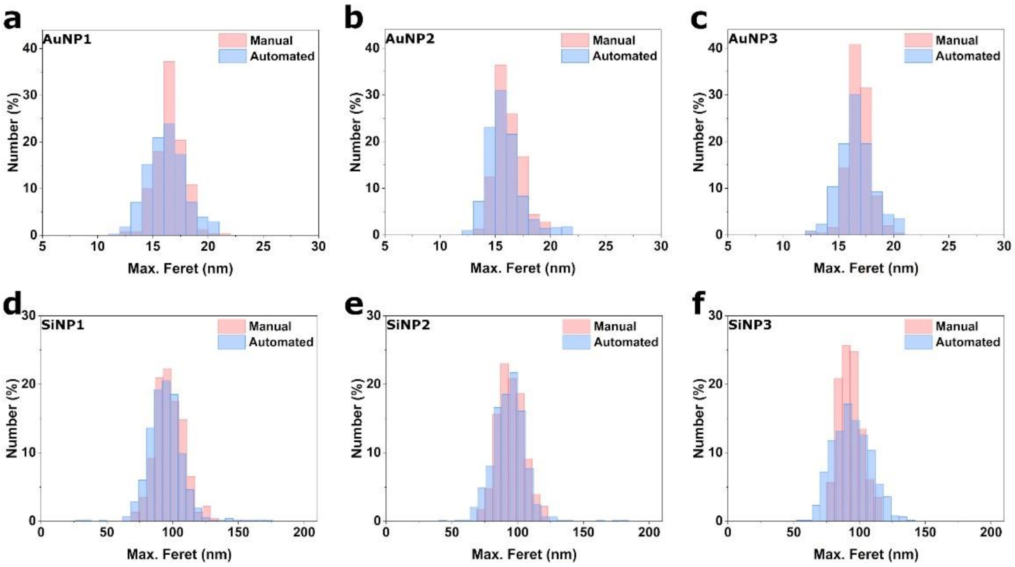

Fig. S7. Comparison of size distributions (Max. Feret) generated by manual measurement of particles (red) and automatic segmentation of the particles (blue) for AuNP system (a-c) and SiNP system (d-f). At least 200 and 1000 particles were analyzed by manual and automated segmentation respectively.

3. Electronic Lab Notebook

After the reaction is completed, an entry is created in the ELN with summarized reaction steps by parsing the log file. The log file, python script used to run the reaction, protocols from the pipetting robot and characterization data including metadata and a preview plot from inline characterization instruments (Zetasizer, Spectramax M3) are also uploaded.

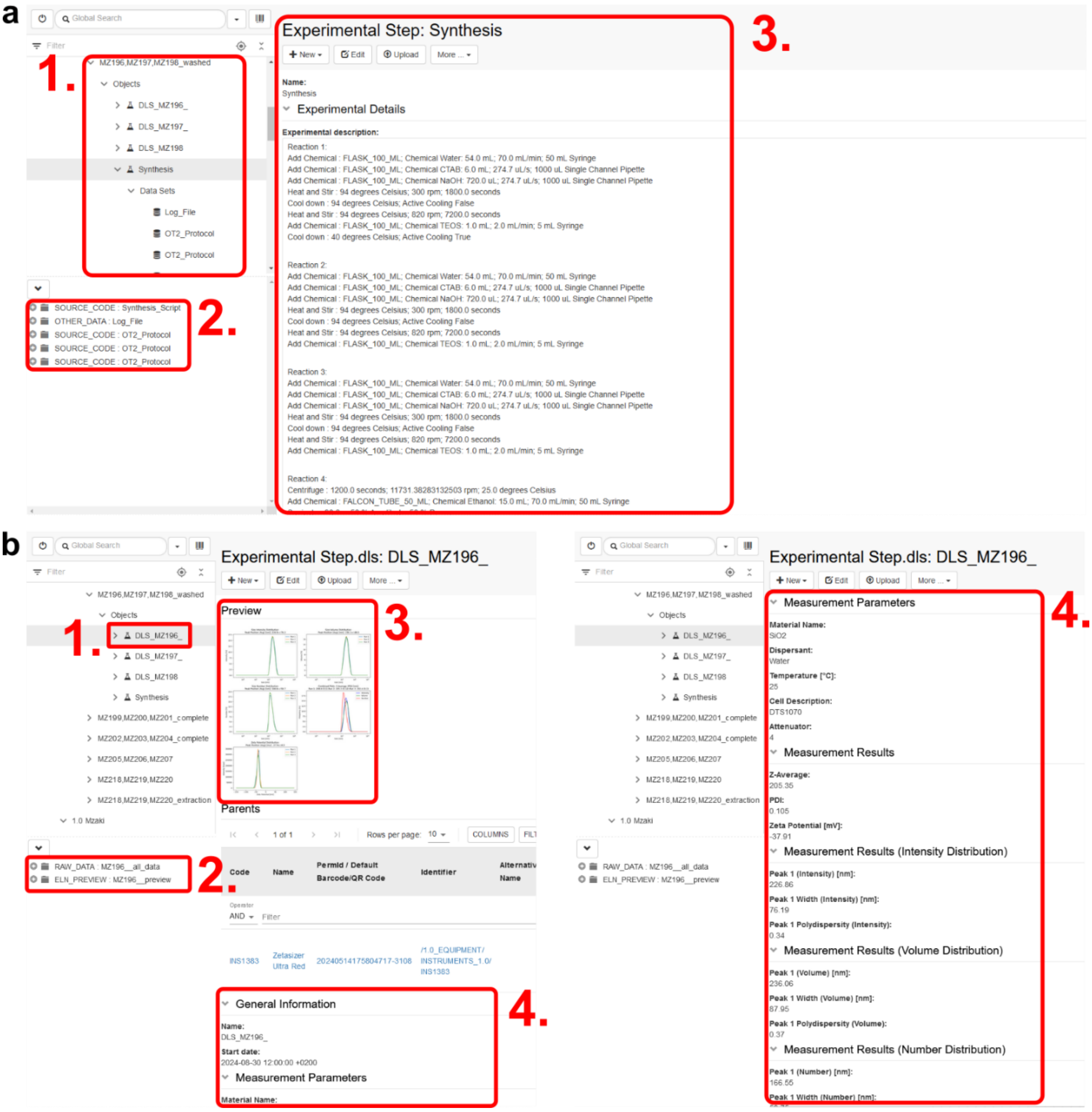

Fig. S8. a) The overview page of the electronic lab notebook, with automatically generated entries for each experimental step associated with the reaction (1.: the synthesis and DLS characterization), all files associated with the currently selected experimental step (2.: the Python script that was run on the SDL for generating the ELN entry, the associated detailed log file, and the automatically generated protocol files for the OT2 pipetting robot that were used during chemical addition steps), and more details on the selected step. For the Synthesis step, this is an automatically generated short description of the reaction that was performed (3.). Each group of steps that is performed during execution of the Python script is labelled as a "Reaction" in this description, i.e., 'Reaction 1' to 'Reaction 3' refer to the synthesis steps in three parallel reactions, while purification steps are represented from 'Reaction 4' onwards. b) The expanded view of the DLS characterization step (1.), showing again the files associated with the step (2.: raw data including metadata as well as an automatically generated preview image), the aforementioned preview image of the automatically generated plots (3.) and the most important data and metadata of the characterization step (4.)

4. Python scripts for the syntheses of Au NPs, SiO_2 NPs, MSNs, ZIF-8, CuO NPs, Au@SiO_2 NPs and CuO@SiO_2 NPs:

The synthesis scripts that were used for synthesizing the various nanomaterial systems discussed in this publication are given below:

4.1. Au NPs

```
import time
import os
import threading

import API.MinervaAPI
import Minerva.Software.OpenBISELNIntegration.OpenBisElnIntegration
from Minerva import *
from Minerva.API.HelperClassDefinitions import PathNames


def Au_NP_Synthesis(container: Container, falcontube1: Container):

    HAuCl4 = Chemical(container=HAuCl4_Container, name='Tetrachloroaurate
trihydrate', volume='1 mL', concentration='2.5 mM', lookup_missing_values=True)
    MilliQ_dilution = Chemical(container=Water_dilution_container, name='Water',
volume='9.0 mL', lookup_missing_values=True)
    Sodium_Citrate = Chemical(container=Citrate_container, name='Sodium citrate
dihydrate', volume='350 uL', lookup_missing_values=True)


    container.add_chemical([HAuCl4, MilliQ_dilution])


    container.infuse_while_heating(chemical=Sodium_Citrate,
                                   heating_temperature=95,
                                   stirring_speed=750,
                                   heating_time='30 min',
                                   addition_hardware=valve2,
                                   withdraw_rate='5 mL/min',
                                   addition_rate='10 mL/min',
                                   purging_addition_rate='10 mL/min',
                                   priming_volume='1 mL',
                                   purging_volume='2.5 mL',
                                   purging_port=5,
                                   priming_waste_container=WasteContainerValve2,
                                   cooldown_temperature=cooling_temperature,

maximum_temperature_deviation=temperature_error,
                                   temperature_stabilization_time='20 min',
                                   chemical_for_cleaning=Water_wash_valve2,
                                   active_cooling=True)


    container.transfer_content_to_container(target_containers=[falcontube1],
transfer_hardware=valve1, dropoff_locations=[(flaskstation, 0),
(falcon_tube_holder_15ml, 0)], bottom_clearance_withdrawing=5)
```

```
if __name__ == '__main__':

    esb = EmergencyStopButton.EmergencyStopButton(com_port='COM34')

    eln =
Minerva.Software.OpenBISELNIntegration.OpenBisElnIntegration.ElectronicLabNotebook(
space_name='1.0_MINERVA', project_code='Gold_RM')

    local_server = LocalPCServer.LocalPCServer(com_port='COM7')

    arduino = ArduinoController.ArduinoController(com_port='COM25')

    dht22 = DHT22Sensor.DHT22Sensor(arduino_controller=arduino)

    robotarm = UFactory.XArm6(ip_address='192.168.1.204',
levelling_data_file=os.path.join('..', 'SampleHolder',
'Table_Levelling_Data.json'))

    ot2 = OpentronsOT2.OT2(ip_address='OT2CEP20210918R04.local')

    hotplate1 = IkaHotplate.RCTDigital5(com_port='COM6')

    hotplate2 = IkaHotplate.RCTDigital5(com_port='COM4')

    hotplate3 = IkaHotplate.RCTDigital5(com_port='COM18')

    hotplate1_clamp =
HotplateClamp.HotplateClampDCMotor(arduino_controller=arduino,
parent_hardware=hotplate1, clamp_number=1)

    hotplate2_clamp =
HotplateClamp.HotplateClampDCMotor(arduino_controller=arduino,
parent_hardware=hotplate2, clamp_number=2)

    hotplate3_clamp =
HotplateClamp.HotplateClampDCMotor(arduino_controller=arduino,
parent_hardware=hotplate3, clamp_number=3)

    hotplate_fan_1 = HotplateFan.HotplateFan(hotplate1, arduino_controller=arduino,
fan_number=1)

    hotplate_fan_2 = HotplateFan.HotplateFan(hotplate2, arduino_controller=arduino,
fan_number=2)

    hotplate_fan_3 = HotplateFan.HotplateFan(hotplate3, arduino_controller=arduino,
fan_number=3)

    capper = CapperDecapper.CapperDecapper(arduino_controller=arduino)

    valve1 = SwitchingValve.SwitchingValveVici(com_port='COM3')

    valve2 = SwitchingValve.SwitchingValveVici(com_port='COM14')

    pump1 = WPI.Aladdin(com_port='COM12')

    pump2 = WPI.Aladdin(com_port='COM9', baud_rate=9600,
pump_type=SyringePumpType.AL_1010)

    probesonicator = Hielscher.UP200ST(ip_address='192.168.233.233')

    centrifuge = Herolab.RobotCen(com_port='COM8', initialize_rotor=True,
home_rotor=True)

    zetasizer = MalvernPanalytical.ZetaSizer(local_controller=local_server)



    gripchange_holder =
SampleHolder.SampleHolder(SampleHolder.SampleHolderDefinitions.Corkring_Small,
deck_position=4)

    hotplate1_holder =
SampleHolder.SampleHolder(SampleHolderDefinitions.Ika_25mL_Heating_Block,
parent_hardware=hotplate1, deck_position=1)
```

```
    hotplate2_holder = 
SampleHolder.SampleHolder(SampleHolderDefinitions.Ika_25mL_Heating_Block, 
parent_hardware=hotplate2, deck_position=2)

    hotplate3_holder = 
SampleHolder.SampleHolder(SampleHolderDefinitions.Ika_25mL_Heating_Block, 
parent_hardware=hotplate3, deck_position=3)

    ot2_holder_15ml = 
SampleHolder.SampleHolder(SampleHolder.SampleHolderDefinitions.Opentrons_15mL_Tube_
Rack, parent_hardware=ot2, deck_position=1)

    ot2_holder_50ml = 
SampleHolder.SampleHolder(SampleHolder.SampleHolderDefinitions.Opentrons_50mL_Tube_
Rack, parent_hardware=ot2, deck_position=4)

    ot2_holder_flask_10ml = 
SampleHolder.SampleHolder(SampleHolder.SampleHolderDefinitions.Opentrons_10mL_Flask
_Rack, parent_hardware=ot2, deck_position=2)

    ot2_holder_flask_25ml = 
SampleHolder.SampleHolder(SampleHolder.SampleHolderDefinitions.Opentrons_25mL_Flask
_Rack, parent_hardware=ot2, deck_position=3)

    falcon_tube_holder_50ml = 
SampleHolder.SampleHolder(SampleHolder.SampleHolderDefinitions.Isolab_50mL_Foldable
_Tube_Rack, deck_position=1, leave_even_rows_empty=True)

    falcon_tube_holder_15ml = 
SampleHolder.SampleHolder(SampleHolder.SampleHolderDefinitions.Isolab_15mL_Foldable
_Tube_Rack, deck_position=2, leave_even_rows_empty=True)

    flaskstation = 
SampleHolder.SampleHolder(SampleHolder.SampleHolderDefinitions.Flask_Station, 
deck_position=3, leave_even_rows_empty=False)



    flaskflush = Container(flaskstation, slot_number=5, 
container_type=ContainerTypeCollection.FLASK_100_ML, current_volume=Volume(0, 
'mL'), has_stirbar=True, name='flask_flush')

    flask_1_25ml = Container(flaskstation, slot_number=1, 
container_type=ContainerTypeCollection.FLASK_25_ML, current_volume=Volume(0, 'mL'), 
has_stirbar=True, name='flask1')

    flask_2_25ml = Container(flaskstation, slot_number=2, 
container_type=ContainerTypeCollection.FLASK_25_ML, current_volume=Volume(0, 'mL'), 
has_stirbar=True, name='flask2')

    flask_3_25ml = Container(flaskstation, slot_number=3, 
container_type=ContainerTypeCollection.FLASK_25_ML, current_volume=Volume(0, 'mL'), 
has_stirbar=True, name='flask3')

    falcontube1_15ml = Container(falcon_tube_holder_15ml, slot_number=1, 
container_type=ContainerTypeCollection.FALCON_TUBE_15_ML, current_volume='0 mL', 
is_capped=True)

    falcontube2_15ml = Container(falcon_tube_holder_15ml, slot_number=2, 
container_type=ContainerTypeCollection.FALCON_TUBE_15_ML, current_volume='0 mL', 
is_capped=True)

    falcontube3_15ml = Container(falcon_tube_holder_15ml, slot_number=3, 
container_type=ContainerTypeCollection.FALCON_TUBE_15_ML, current_volume='0 mL', 
is_capped=True)

    falcontube4_15ml = Container(falcon_tube_holder_15ml, slot_number=4, 
container_type=ContainerTypeCollection.FALCON_TUBE_15_ML, current_volume='0 mL', 
is_capped=True)

    falcontube5_15ml = Container(falcon_tube_holder_15ml, slot_number=5, 
container_type=ContainerTypeCollection.FALCON_TUBE_15_ML, current_volume='0 mL', 
is_capped=True)
```

```
    falcontube_for_cleaning = Container(falcon_tube_holder_50ml, slot_number=14,
container_type=ContainerTypeCollection.FALCON_TUBE_50_ML, current_volume='40 mL',
is_capped=True)



    WasteContainerValve1 = Container(current_hardware=valve1, slot_number=3,
name='Waste_Container', current_volume='2 L', max_volume='5 L')

    WasteContainerValve2 = Container(current_hardware=valve2, slot_number=6,
name='Waste_ContainerValve2', current_volume='0 mL', max_volume='50 mL')

    DLSCell = Container(current_hardware=valve1, slot_number=4, name='DLS_Cell',
current_volume='0 mL')

    MilliQ_ContainerValve1 = Container(current_hardware=valve1, slot_number=1,
name='MilliQ_ContainerValve1', current_volume='1.2 L')

    MilliQ_ContainerValve2 = Container(current_hardware=valve2, slot_number=4,
name='CleaningChemicalValve2', current_volume='50 mL')

    Ethanol_wash_container = Container(current_hardware=valve1, slot_number=2,
name='Ethanol_wash', current_volume='5 L')



    HAuCl4_Container = Container(current_hardware=ot2, deck_position=8,
slot_number=1, name='HAuCl4_Container', current_volume='2.5 mL',
container_type=ContainerTypeCollection.FALCON_TUBE_15_ML)

    Water_dilution_container = Container(current_hardware=ot2, deck_position=8,
slot_number=7, name='Water_Container', current_volume='25 mL',
container_type=ContainerTypeCollection.FALCON_TUBE_50_ML)

    Citrate_container = Container(current_hardware=valve2, slot_number=0,
name='Sodium_Citrate_Container', current_volume='3 mL')



    Water_wash_valve2 = Chemical(container=MilliQ_ContainerValve2, name='Water',
volume='2 mL', lookup_missing_values=True)

    Water_wash = Chemical(container=MilliQ_ContainerValve1, name='Water',
volume='10 mL', lookup_missing_values=True)

    Ethanol_wash = Chemical(container=Ethanol_wash_container, name='Ethanol',
volume='20 mL', lookup_missing_values=True)



    temperature_error = 2  # °C
    cooling_temperature = 30  # °C
    sonication_time = '5 s'
    washing_steps = 1
    centrifugation_speed = '3500 rcf'
    centrifugation_time = '90 min'
    centrifugation_temp = 5  # °C
    sample_name_1 = 'MZ146'
    sample_name_2 = 'MZ147'


    ot2_configuration = {
        1: ot2_holder_15ml,
        2: ot2_holder_flask_10ml,
        3: ot2_holder_flask_25ml,
        4: None,
        5: None,
        6: None,
        7: None,
        8: 'opentrons_10_tuberack_falcon_4x50ml_6x15ml_conical',
        9: None,
        10:'opentrons_96_tiprack_20ul',    # 'opentrons_96_tiprack_300ul'
        11:'labsolute_96_tiprack_1000ul',
        12:'p1000_single_gen2',  # left pipette
        13:'p20_single_gen2',  # right pipette  'p300_single_gen2'
    }
```

```
    valve1_configuration = {
        0: None,
        1: MilliQ_ContainerValve1,
        2: Ethanol_wash_container,
        3: WasteContainerValve1,
        4: DLSCell,
        5: None,
        6: None,
        7: None,
        8: 'Outlet',
        9: None,
        10: pump1
    }

    valve2_configuration = {
        0: Citrate_container,
        1: hotplate1,
        2: hotplate2,
        3: hotplate3,
        4: MilliQ_ContainerValve2,
        5: None,
        6: WasteContainerValve2,
        7: None,
        8: None,
        9: None,
        10: pump2
    }

    valve2_dead_volumes = {
        0: None,
        1: '1.390 mL',
        2: '1.35 mL',
        3: '1.87 mL',
        4: None,
        5: None,
        6: None,
        7: None,
        8: None,
        9: None,
        10: None
    }

    ot2.set_hardware_configuration(ot2_configuration)

    pump1.set_syringe(Syringes.GLASS_SYRINGE_SOCOREX_50ML,
default_addition_rate='50 mL/min')

    valve1.set_configuration(valve1_configuration)

    pump2.set_syringe(Syringes.GLASS_SYRINGE_SOCOREX_5ML, default_addition_rate='10
mL/min')

    valve2.set_configuration(valve2_configuration)

    valve2.set_dead_volumes(valve2_dead_volumes)


    Au_NP_Synthesis_reaction_parameters = ((flask_1_25ml, falcontube1_15ml),
(flask_2_25ml, falcontube2_15ml))


    threads = []

    for i, p in enumerate(Au_NP_Synthesis_reaction_parameters):

        threads.append(threading.Thread(name=f'Thread_{i}', target=Au_NP_Synthesis,
args=tuple(p)))
```

```
        threads[-1].start()

        time.sleep(300)


    for t in threads:

       t.join()


    # Washing

    for _ in washing_steps:

        falcontube1_15ml.centrifuge([falcontube2_15ml],
centrifugation_speed=centrifugation_speed, centrifugation_time=centrifugation_time,
centrifugation_temperature=centrifugation_temp)


falcontube1_15ml.remove_supernatant_and_redisperse(waste_container=WasteContainerVa
lve1, sonication_time=sonication_time, redispersion_chemical=Water_wash,
sonication_power=50, sonication_amplitude=50, sonicator=probesonicator,
bottom_clearance_sonication=10, container_for_cleaning=falcontube_for_cleaning)


falcontube2_15ml.remove_supernatant_and_redisperse(waste_container=WasteContainerVa
lve1, sonication_time=sonication_time, redispersion_chemical=Water_wash,
sonication_power=50, sonication_amplitude=50, sonicator=probesonicator,
bottom_clearance_sonication=10, container_for_cleaning=falcontube_for_cleaning)


    # Dilution for DLS measurement

    falcontube1_15ml.transfer_content_to_container(falcontube3_15ml,
transfer_hardware=valve1, volume='1 mL', bottom_clearance_withdrawing=10)

    falcontube1_15ml.add_chemical(chemical=Chemical.from_stock_chemical(Water_wash,
volume='4 mL'))

    falcontube2_15ml.transfer_content_to_container(falcontube4_15ml,
transfer_hardware=valve1, volume='1 mL', bottom_clearance_withdrawing=10)

    falcontube2_15ml.add_chemical(chemical=Chemical.from_stock_chemical(Water_wash,
volume='4 mL'))


    # DLS measurement

    falcontube3_15ml.measure_dls(dls_cell=DLSCell, sop_path=os.path.join('C:\\',
'users', 'WS8717-apollo', 'Desktop', 'Apollo', 'Characterization', 'DLS', 'SOP',
'DLS_Au_in_Water.zskd'), sample_name=sample_name_1,
chemical_for_cleaning=Water_wash, dls_device=zetasizer,
bottom_clearance_sampling=9, waste_container=WasteContainerValve1)

    falcontube4_15ml.measure_dls(dls_cell=DLSCell, sop_path=os.path.join('C:\\',
'users', 'WS8717-apollo', 'Desktop', 'Apollo', 'Characterization', 'DLS', 'SOP',
'DLS_Au_in_Water.zskd'), sample_name=sample_name_2,
chemical_for_cleaning=Water_wash, dls_device=zetasizer,
bottom_clearance_sampling=9, waste_container=WasteContainerValve1)


    # ELN upload

    eln.write_synthesis_step(experiment_name=f"{sample_name1},{sample_name2}")
```

4.2. SiO_2 NPs

```
import time
import os
```

```
import threading

import API.MinervaAPI
import Minerva.Software.OpenBISELNIntegration.OpenBisElnIntegration
from Minerva import *
from Minerva.API.HelperClassDefinitions import PathNames


def Stober_Silica_Synthesis(container: Container, falcontube1: Container,
falcontube2: Container):

    EtOH_NH3_water_mix = Chemical(container=EtOH_NH3_water_container,
name='EtOH_NH3_water', volume='8.84 mL', lookup_missing_values=False)

    TEOS_ethanolic = Chemical(container=TEOS_container, name='TEOS', volume='27.67
mL', lookup_missing_values=True)

    Chemical_Cleaning_valve2 = Chemical(container=EthanolContainerValve2,
name='Ethanol', volume='5 mL')



    stirring_speed = 300  # rpm
    reaction_temperature = 25  # °C
    heating_time = '24 h'
    temperature_error = 2  # °C


    container.add_chemical(chemical=EtOH_NH3_water_mix, priming_volume='1 mL',
priming_waste_container=WasteContainerValve1)


    container.infuse_while_heating(chemical=[TEOS_ethanolic],

                                   heating_temperature=reaction_temperature,

                                   stirring_speed=stirring_speed,

                                   heating_time=heating_time,

                                   addition_hardware=valve2,

                                   withdraw_rate='20 mL/min',

                                   addition_rate='30 mL/min',

                                   priming_volume='1.5 mL',

                                   purging_volume='8 mL',

                                   purging_port=5,

                                   priming_waste_container=WasteContainerValve2,

                                   cooldown_temperature=None,

                                   maximum_temperature_deviation=temperature_error,

                                   temperature_stabilization_time='5 min',

                                   chemical_for_cleaning=Chemical_Cleaning_valve2,

                                   active_cooling=False)


    container.transfer_content_to_container(target_containers=[falcontube1,
falcontube2], transfer_hardware=valve1, dropoff_locations=[(flaskstation, 0),
(falcon_tube_holder_50ml, 0), (falcon_tube_holder_50ml, 0)],
bottom_clearance_withdrawing=5)


if __name__ == '__main__':

    esb = EmergencyStopButton.EmergencyStopButton(com_port='COM34')
```

```
    eln =
Minerva.Software.OpenBISELNIntegration.OpenBisElnIntegration.ElectronicLabNotebook(
space_name='1.0_MINERVA', project_code='SiNP')

    local_server = LocalPCServer.LocalPCServer(com_port='COM7')

    arduino = ArduinoController.ArduinoController(com_port='COM25')

    dht22 = DHT22Sensor.DHT22Sensor(arduino_controller=arduino)

    robotarm = UFactory.XArm6(ip_address='192.168.1.204',
levelling_data_file=os.path.join('..', 'SampleHolder',
'Table_Levelling_Data.json'))

    ot2 = OpentronsOT2.OT2(ip_address='OT2CEP20210918R04.local')

    hotplate1 = IkaHotplate.RCTDigital5(com_port='COM6')

    hotplate2 = IkaHotplate.RCTDigital5(com_port='COM4')

    hotplate3 = IkaHotplate.RCTDigital5(com_port='COM18')

    hotplate1_clamp =
HotplateClamp.HotplateClampDCMotor(arduino_controller=arduino,
parent_hardware=hotplate1, clamp_number=1)

    hotplate2_clamp =
HotplateClamp.HotplateClampDCMotor(arduino_controller=arduino,
parent_hardware=hotplate2, clamp_number=2)

    hotplate3_clamp =
HotplateClamp.HotplateClampDCMotor(arduino_controller=arduino,
parent_hardware=hotplate3, clamp_number=3)

    hotplate_fan_1 = HotplateFan.HotplateFan(hotplate1, arduino_controller=arduino,
fan_number=1)

    hotplate_fan_2 = HotplateFan.HotplateFan(hotplate2, arduino_controller=arduino,
fan_number=2)

    hotplate_fan_3 = HotplateFan.HotplateFan(hotplate3, arduino_controller=arduino,
fan_number=3)

    capper = CapperDecapper.CapperDecapper(arduino_controller=arduino)

    valve1 = SwitchingValve.SwitchingValveVici(com_port='COM3')

    valve2 = SwitchingValve.SwitchingValveVici(com_port='COM14')

    valve3 = SwitchingValve.SwitchingValveVici(com_port='COM17')

    pump1 = WPI.Aladdin(com_port='COM12')

    pump2 = WPI.Aladdin(com_port='COM9', baud_rate=9600,
pump_type=SyringePumpType.AL_1010)

    pump3 = WPI.Aladdin(com_port='COM5', baud_rate=9600,
pump_type=SyringePumpType.AL_1010)

    probesonicator = Hielscher.UP200ST(ip_address='192.168.233.233')

    centrifuge = Herolab.RobotCen(com_port='COM8', initialize_rotor=True,
home_rotor=True)

    zetasizer = MalvernPanalytical.ZetaSizer(local_controller=local_server)



    gripchange_holder =
SampleHolder.SampleHolder(SampleHolder.SampleHolderDefinitions.Corkring_Small,
deck_position=4)

    hotplate1_holder =
SampleHolder.SampleHolder(SampleHolderDefinitions.Ika_100mL_Heating_Block,
parent_hardware=hotplate1, deck_position=1)
```

```
    hotplate2_holder =
SampleHolder.SampleHolder(SampleHolderDefinitions.Ika_100mL_Heating_Block,
parent_hardware=hotplate2, deck_position=2)

    hotplate3_holder =
SampleHolder.SampleHolder(SampleHolderDefinitions.Ika_100mL_Heating_Block,
parent_hardware=hotplate3, deck_position=3)

    ot2_holder_15ml =
SampleHolder.SampleHolder(SampleHolder.SampleHolderDefinitions.Opentrons_15mL_Tube_
Rack, parent_hardware=ot2, deck_position=2)

    ot2_holder_50ml =
SampleHolder.SampleHolder(SampleHolder.SampleHolderDefinitions.Opentrons_50mL_Tube_
Rack, parent_hardware=ot2, deck_position=1)

    ot2_holder_flask_100ml =
SampleHolder.SampleHolder(SampleHolder.SampleHolderDefinitions.Opentrons_100mL_Flas
k_Rack, parent_hardware=ot2, deck_position=3)

    falcon_tube_holder_50ml =
SampleHolder.SampleHolder(SampleHolder.SampleHolderDefinitions.Isolab_50mL_Foldable
_Tube_Rack, deck_position=1)

    falcon_tube_holder_15ml =
SampleHolder.SampleHolder(SampleHolder.SampleHolderDefinitions.Isolab_15mL_Foldable
_Tube_Rack, deck_position=2)

    flaskstation =
SampleHolder.SampleHolder(SampleHolder.SampleHolderDefinitions.Flask_Station,
deck_position=3, leave_even_rows_empty=False)


    flask_1_100ml = Container(flaskstation, slot_number=1,
container_type=ContainerTypeCollection.FLASK_100_ML, current_volume=Volume(0,
'mL'), has_stirbar=True, name='flask1')

    flask_2_100ml = Container(flaskstation, slot_number=2,
container_type=ContainerTypeCollection.FLASK_100_ML, current_volume=Volume(0,
'mL'), has_stirbar=True, name='flask2')

    flask_3_100ml = Container(flaskstation, slot_number=3,
container_type=ContainerTypeCollection.FLASK_100_ML, current_volume=Volume(0,
'mL'), has_stirbar=True, name='flask3')

    flask_4_100ml = Container(flaskstation, slot_number=4,
container_type=ContainerTypeCollection.FLASK_100_ML, current_volume=Volume(0,
'mL'), has_stirbar=True, name='flask4')

    flask_5_100ml = Container(flaskstation, slot_number=5,
container_type=ContainerTypeCollection.FLASK_100_ML, current_volume=Volume(0,
'mL'), has_stirbar=False, name='flask5')

    falcontube1_15ml = Container(falcon_tube_holder_15ml, slot_number=1,
container_type=ContainerTypeCollection.FALCON_TUBE_15_ML, current_volume='0 mL',
is_capped=True)

    falcontube2_15ml = Container(falcon_tube_holder_15ml, slot_number=2,
container_type=ContainerTypeCollection.FALCON_TUBE_15_ML, current_volume='0 mL',
is_capped=True)

    falcontube3_15ml = Container(falcon_tube_holder_15ml, slot_number=3,
container_type=ContainerTypeCollection.FALCON_TUBE_15_ML, current_volume='0 mL',
is_capped=True)

    falcontube15ml_counter = Container(falcon_tube_holder_15ml, slot_number=3,
container_type=ContainerTypeCollection.FALCON_TUBE_15_ML, current_volume='10 mL',
is_capped=True)

    falcontube1_50ml = Container(falcon_tube_holder_50ml, slot_number=1,
container_type=ContainerTypeCollection.FALCON_TUBE_50_ML, current_volume='0 mL',
is_capped=False)
```

```
    falcontube2_50ml = Container(falcon_tube_holder_50ml, slot_number=2, container_type=ContainerTypeCollection.FALCON_TUBE_50_ML, current_volume='0 mL', is_capped=False)

    falcontube3_50ml = Container(falcon_tube_holder_50ml, slot_number=3, container_type=ContainerTypeCollection.FALCON_TUBE_50_ML, current_volume='0 mL', is_capped=False)

    falcontube4_50ml = Container(falcon_tube_holder_50ml, slot_number=4, container_type=ContainerTypeCollection.FALCON_TUBE_50_ML, current_volume='0 mL', is_capped=False)

    falcontube5_50ml = Container(falcon_tube_holder_50ml, slot_number=15, container_type=ContainerTypeCollection.FALCON_TUBE_50_ML, current_volume='0 mL', is_capped=False)

    falcontube6_50ml = Container(falcon_tube_holder_50ml, slot_number=17, container_type=ContainerTypeCollection.FALCON_TUBE_50_ML, current_volume='0 mL', is_capped=True)

    falcontube_for_cleaning = Container(falcon_tube_holder_50ml, slot_number=14, container_type=ContainerTypeCollection.FALCON_TUBE_50_ML, current_volume='40 mL', is_capped=False)


    WasteContainerValve1 = Container(current_hardware=valve1, slot_number=0, name='Waste_Container', current_volume='2 L', max_volume='5 L')

    WasteContainerValve2 = Container(current_hardware=valve2, slot_number=6, name='Waste_ContainerValve2', current_volume='0 mL', max_volume='50 mL')

    DLSCell = Container(current_hardware=valve1, slot_number=4, name='DLS_Cell', current_volume='0 mL')

    MilliQ_ContainerValve1 = Container(current_hardware=valve1, slot_number=1, name='MilliQ_ContainerValve1', current_volume='1.2 L')

    Ethanol_wash_container = Container(current_hardware=valve1, slot_number=5, name='Ethanol_wash_valve1', current_volume='5 L')

    EthanolContainerValve2 = Container(current_hardware=valve2, slot_number=9, name='Ethanol_wash_valve2', current_volume='500 mL')


    Ethanol_wash = Chemical(container=Ethanol_wash_container, name='Ethanol', volume='15 mL', lookup_missing_values=True)

    Water_wash = Chemical(container=MilliQ_ContainerValve1, name='Water', volume='15 mL', lookup_missing_values=True)

    TEOS_container = Container(current_hardware=valve2, slot_number=9, name='TEOS_container', current_volume='100 mL', max_volume='500 mL')  # dilution 3 ml in 80 mL EtOH

    EtOH_NH3_water_container = Container(current_hardware=valve1, slot_number=6, name='EtOH_NH3_water_container', current_volume='53 mL', max_volume='100 mL')


    sonication_time = '40 s'

    centrifugation_speed = '14000 rcf'

    centrifugation_time_water = '20 min'

    centrifugation_time_EtOH = '15 min'

    washing_steps = 2

    sample_name_1 = 'MZ026'

    sample_name_2 = 'MZ027'

    sample_name_3 = 'MZ028'
```

```
ot2_configuration = {
    1: ot2_holder_50ml,
    2: ot2_holder_15ml,
    3: ot2_holder_flask_100ml,
    4: None,
    5: None,
    6: None,
    7: None,
    8: 'opentrons_10_tuberack_falcon_4x50ml_6x15ml_conical',
    9: None,
    10: 'opentrons_96_tiprack_300ul',
    11: 'labsolute_96_tiprack_1000ul',
    12: 'p1000_single_gen2',  # left pipette
    13: 'p300_single_gen2',  # right pipette
}


valve1_configuration = {
    0: None,
    1: MilliQ_ContainerValve1,
    2: Ethanol_wash_container,
    3: WasteContainerValve1,
    4: DLSCell,
    5: None,
    6: EtOH_NH3_water_container,
    7: None,
    8: 'Outlet',
    9: None,
    10: pump1
}


valve2_configuration = {
    0: None,
    1: hotplate1,
    2: hotplate2,
    3: hotplate3,
    4: MilliQ_ContainerValve2,
    5: None,
    6: WasteContainerValve2,
    7: None,
    8: None,
    9: TEOSContainer,
    10: pump2
}


valve3_configuration = {
    0: None,
    1: hotplate1,
    2: hotplate2,
    3: hotplate3,
    4: MilliQ_ContainerValve3,
    5: None,
    6: None,
    7: None,
    8: None,
    9: WasteContainerValve3,
    10: pump3
}


valve2_dead_volumes = {
    0: None,
    1: '1.390 mL',
    2: '1.35 mL',
    3: '1.87 mL',
    4: None,
    5: None,
    6: None,
    7: None,
```

```
        8: None,
        9: None,
        10: None
    }


    ot2.set_hardware_configuration(ot2_configuration)

    pump1.set_syringe(Syringes.GLASS_SYRINGE_SOCOREX_50ML,
default_addition_rate='70 mL/min')

    valve1.set_configuration(valve1_configuration)

    pump2.set_syringe(Syringes.PLASTICSYRINGE_LABSOLUTE_24ML,
default_addition_rate='40 mL/min')

    valve2.set_configuration(valve2_configuration)

    valve2.set_dead_volumes(valve2_dead_volumes)

    pump3.set_syringe(Syringes.GLASS_SYRINGE_SOCOREX_5ML, default_addition_rate='20
mL/min')

    valve3.set_configuration(valve3_configuration)



    Stober_Silica_Synthesis_parameters = ((flask_1_100ml, falcontube1_50ml,
falcontube2_50ml),

    (flask_2_100ml, falcontube3_50ml, falcontube4_50ml), (flask_3_100ml,
falcontube5_50ml, falcontube6_50ml))

    threads = []

    for i, p in enumerate(Stober_Silica_Synthesis_parameters):

        threads.append(threading.Thread(name=f'Thread_{i}',
target=Stober_Silica_Synthesis, args=tuple(p)))

        threads[0].start()

        time.sleep(40)

    for t in threads:

        t.join()



    # Washing

    falcontube1_50ml.centrifuge([falcontube2_50ml, falcontube3_50ml,
falcontube4_50ml, falcontube5_50ml, falcontube6_50ml],
centrifugation_speed=centrifugation_speed,
centrifugation_time=centrifugation_time_water)


falcontube1_50ml.remove_supernatant_and_redisperse(waste_container=WasteContainerVa
lve1, redispersion_chemical=Water_wash, sonicator=probesonicator,
sonication_time=sonication_time, sonication_power=50, sonication_amplitude=50,
container_for_cleaning=falcontube_for_cleaning)


falcontube2_50ml.remove_supernatant_and_redisperse(waste_container=WasteContainerVa
lve1, redispersion_chemical=Water_wash, sonicator=probesonicator,
sonication_time=sonication_time, sonication_power=50, sonication_amplitude=50,
container_for_cleaning=falcontube_for_cleaning)


falcontube3_50ml.remove_supernatant_and_redisperse(waste_container=WasteContainerVa
lve1, redispersion_chemical=Water_wash, sonicator=probesonicator,
sonication_time=sonication_time, sonication_power=50, sonication_amplitude=50,
container_for_cleaning=falcontube_for_cleaning)


falcontube4_50ml.remove_supernatant_and_redisperse(waste_container=WasteContainerVa
```

```
lve1, redispersion_chemical=Water_wash, sonicator=probesonicator,
sonication_time=sonication_time, sonication_power=50, sonication_amplitude=50,
container_for_cleaning=falcontube_for_cleaning)


falcontube5_50ml.remove_supernatant_and_redisperse(waste_container=WasteContainerVa
lve1, redispersion_chemical=Water_wash, sonicator=probesonicator,
sonication_time=sonication_time, sonication_power=50, sonication_amplitude=50,
container_for_cleaning=falcontube_for_cleaning)


falcontube6_50ml.remove_supernatant_and_redisperse(waste_container=WasteContainerVa
lve1, redispersion_chemical=Water_wash, sonicator=probesonicator,
sonication_time=sonication_time, sonication_power=50, sonication_amplitude=50,
container_for_cleaning=falcontube_for_cleaning)

    for _ in washing_steps:

        falcontube1_50ml.centrifuge([falcontube2_50ml, falcontube3_50ml,
falcontube4_50ml, falcontube5_50ml, falcontube6_50ml],
centrifugation_speed=centrifugation_speed,
centrifugation_time=centrifugation_time_EtOH)


falcontube1_50ml.remove_supernatant_and_redisperse(waste_container=WasteContainerVa
lve1, redispersion_chemical=Ethanol_wash, sonicator=probesonicator,
sonication_time=sonication_time, sonication_power=50, sonication_amplitude=50,
container_for_cleaning=falcontube_for_cleaning)


falcontube2_50ml.remove_supernatant_and_redisperse(waste_container=WasteContainerVa
lve1, redispersion_chemical=Ethanol_wash, sonicator=probesonicator,
sonication_time=sonication_time, sonication_power=50, sonication_amplitude=50,
container_for_cleaning=falcontube_for_cleaning)


falcontube3_50ml.remove_supernatant_and_redisperse(waste_container=WasteContainerVa
lve1, redispersion_chemical=Ethanol_wash, sonicator=probesonicator,
sonication_time=sonication_time, sonication_power=50, sonication_amplitude=50,
container_for_cleaning=falcontube_for_cleaning)


falcontube4_50ml.remove_supernatant_and_redisperse(waste_container=WasteContainerVa
lve1, redispersion_chemical=Ethanol_wash, sonicator=probesonicator,
sonication_time=sonication_time, sonication_power=50, sonication_amplitude=50,
container_for_cleaning=falcontube_for_cleaning)


falcontube5_50ml.remove_supernatant_and_redisperse(waste_container=WasteContainerVa
lve1, redispersion_chemical=Ethanol_wash, sonicator=probesonicator,
sonication_time=sonication_time, sonication_power=50, sonication_amplitude=50,
container_for_cleaning=falcontube_for_cleaning)


falcontube6_50ml.remove_supernatant_and_redisperse(waste_container=WasteContainerVa
lve1, redispersion_chemical=Ethanol_wash, sonicator=probesonicator,
sonication_time=sonication_time, sonication_power=50, sonication_amplitude=50,
container_for_cleaning=falcontube_for_cleaning)



    # Redispersion in water for DLS

    falcontube1_50ml.transfer_content_to_container(falcontube1_15ml, volume='2 mL',
bottom_clearance_withdrawing=15, transfer_hardware=valve1, purging_volume='10 mL')

    falcontube1_15ml.add_chemical([Chemical.from_stock_chemical(Ethanol_wash,
volume='8 mL')])

    falcontube3_50ml.transfer_content_to_container(falcontube2_15ml, volume='2 mL',
bottom_clearance_withdrawing=15, transfer_hardware=valve1, purging_volume='10 mL')

    falcontube2_15ml.add_chemical([Chemical.from_stock_chemical(Ethanol_wash,
volume='8 mL')])
```

```
    falcontube5_50ml.transfer_content_to_container(falcontube3_15ml, volume='2 mL', bottom_clearance_withdrawing=15, transfer_hardware=valve1, purging_volume='10 mL')

    falcontube3_15ml.add_chemical([Chemical.from_stock_chemical(Ethanol_wash, volume='8 mL')])



    falcontube1_15ml.centrifuge(containers=[falcontube2_15ml, falcontube3_15ml, falcontube4_15ml], centrifugation_time='12 min', centrifugation_speed=centrifugation_speed)


falcontube1_15ml.remove_supernatant_and_redisperse(waste_container=WasteContainerValve1, sonication_time='30 s', redispersion_chemical=Chemical.from_stock_chemical(Water_wash, volume='10 mL'), sonication_power=50, sonication_amplitude=50, sonicator=probesonicator, bottom_clearance_sonication=20, purging_volume='10 mL', bottom_clearance_withdrawing=2, container_for_cleaning=falcontube_for_cleaning)


falcontube2_15ml.remove_supernatant_and_redisperse(waste_container=WasteContainerValve1, sonication_time='30 s', redispersion_chemical=Chemical.from_stock_chemical(Water_wash, volume='10 mL'), sonication_power=50, sonication_amplitude=50, sonicator=probesonicator, bottom_clearance_sonication=20, purging_volume='10 mL', bottom_clearance_withdrawing=2, container_for_cleaning=falcontube_for_cleaning)


falcontube3_15ml.remove_supernatant_and_redisperse(waste_container=WasteContainerValve1, sonication_time='30 s', redispersion_chemical=Chemical.from_stock_chemical(Water_wash, volume='10 mL'), sonication_power=50, sonication_amplitude=50, sonicator=probesonicator, bottom_clearance_sonication=20, purging_volume='10 mL', bottom_clearance_withdrawing=2, container_for_cleaning=falcontube_for_cleaning)



    # DLS measurement

    falcontube1_15ml.measure_dls(dls_cell=DLSCell, sop_path=os.path.join('C:\\', 'users', 'WS8717-apollo', 'Desktop',  'Apollo', 'Characterization', 'DLS', 'SOP', 'DLS_ZETA_SiO2_in_Water.zskd'), sample_name=sample_name_1, chemical_for_cleaning=Water_wash, dls_device=zetasizer, bottom_clearance_sampling=9, waste_container=WasteContainerValve1)

    falcontube2_15ml.measure_dls(dls_cell=DLSCell, sop_path=os.path.join('C:\\', 'users', 'WS8717-apollo', 'Desktop', 'Apollo', 'Characterization', 'DLS', 'SOP', 'DLS_ZETA_SiO2_in_Water.zskd'), sample_name=sample_name_2, chemical_for_cleaning=Water_wash, dls_device=zetasizer, bottom_clearance_sampling=9, waste_container=WasteContainerValve1)

    falcontube3_15ml.measure_dls(dls_cell=DLSCell, sop_path=os.path.join('C:\\', 'users', 'WS8717-apollo', 'Desktop', 'Apollo', 'Characterization', 'DLS', 'SOP', 'DLS_ZETA_SiO2_in_Water.zskd'), sample_name=sample_name_3, chemical_for_cleaning=Water_wash, dls_device=zetasizer, bottom_clearance_sampling=9, waste_container=WasteContainerValve1)

    # ELN upload


eln.write_synthesis_step(experiment_name=f"{sample_name1},{sample_name2},{sample_name_3}")
```

4.3. MSNs

```
import time
import datetime
import os
import threading

import API.MinervaAPI
```

```python
import Minerva.Software.OpenBISELNIntegration.OpenBisElnIntegration
from Minerva import *
from Minerva.API.HelperClassDefinitions import PathNames


def MSN_Synthesis(container: Container, falcontube1: Container, falcontube2: Container, priming_volume):

    CTAB = Chemical(container=CTABContainer, name='CTAB', mass_concentration='20 mg/mL', mass='120 mg', lookup_missing_values=False)

    NaOH = Chemical(container=NaOHContainer, name='NaOH', volume='720 uL', concentration='1 M', lookup_missing_values=False)

    Water = Chemical(container=MilliQ_ContainerValve1, name='Water', volume='54 mL', lookup_missing_values=False)

    Ethanolic_TEOS = Chemical(container=TEOSContainer, name='TEOS', volume='1 mL', concentration='2.7 mol/L', lookup_missing_values=False)



    stirring_speed = 820  # rpm
    reaction_temperature = 95  # °C
    heating_time = '2 h'
    temperature_error = 2  # °C
    cooling_temperature = 40  # °C
    teos_infusion_rate = '2.0 mL/min'


    container.add_chemical([Water, CTAB, NaOH])

    container.heat(heating_temperature=94, stirring_speed=300, heating_time='30 min', temperature_stabilization_time='1 min', cooldown_temperature=94, active_cooling=False)

    container.infuse_while_heating(chemical=[TEOS],
                                   heating_temperature=reaction_temperature,
                                   stirring_speed=stirring_speed,
                                   heating_time=heating_time,
                                   addition_hardware=valve2,
                                   withdraw_rate='5 mL/min',
                                   addition_rate=teos_infusion_rate,
                                   purging_addition_rate='10 mL/min',
                                   priming_volume=priming_volume,
                                   purging_volume='4 mL',
                                   purging_port=5,
                                   priming_waste_container=WasteContainerValve2,
                                   cooldown_temperature=cooling_temperature,
                                   maximum_temperature_deviation=temperature_error,
                                   temperature_stabilization_time='1 min',
                                   chemical_for_cleaning=Water_wash_valve2,
                                   active_cooling=True)


    container.transfer_content_to_container(target_containers=[falcontube1, falcontube2], transfer_hardware=valve1, bottom_clearance_withdrawing=8, dropoff_locations=[(flaskstation, 0), (falcon_tube_holder_50ml, 0), (falcon_tube_holder_50ml, 0)], purging_volume='15 mL')



def NH4NO3_extraction(container: Container, falcontube1: Container, falcontube2: Container):

    stirring_speed = 600  # rpm

    reaction_temperature = 72  # °C
```

```
    heating_time = '1.5 h'

    temperature_error = 2  # °C

    cooling_temperature = 40  # °C


    falcontube1.transfer_content_to_container(container, transfer_hardware=valve1, dropoff_locations=[(falcon_tube_holder_50ml, 0), (flaskstation, 0)], bottom_clearance_withdrawing=4, purging_volume='15 mL')

    falcontube2.transfer_content_to_container(container, transfer_hardware=valve1, dropoff_locations=[(falcon_tube_holder_50ml, 0), (flaskstation, 0)], bottom_clearance_withdrawing=4, purging_volume='15 mL')


    container.heat(heating_temperature=reaction_temperature, stirring_speed=stirring_speed, heating_time=heating_time, cooldown_temperature=cooling_temperature, active_cooling=True, temperature_stabilization_time='10 min')


    container.transfer_content_to_container(target_containers=[falcontube1, falcontube2], transfer_hardware=valve1, bottom_clearance_withdrawing=8, dropoff_locations=[(flaskstation, 0), (falcon_tube_holder_50ml, 0), (falcon_tube_holder_50ml, 0)], purging_volume='15 mL')



if __name__ == '__main__':

    esb = EmergencyStopButton.EmergencyStopButton(com_port='COM34')

    eln = Minerva.Software.OpenBISELNIntegration.OpenBisElnIntegration.ElectronicLabNotebook(space_name='1.0_MINERVA', project_code='MSN')

    local_server = LocalPCServer.LocalPCServer(com_port='COM7')

    arduino = ArduinoController.ArduinoController(com_port='COM25')

    dht22 = DHT22Sensor.DHT22Sensor(arduino_controller=arduino)

    robotarm = UFactory.XArm6(ip_address='192.168.1.204', levelling_data_file=os.path.join('..', 'SampleHolder', 'Table_Levelling_Data.json'))

    ot2 = OpentronsOT2.OT2(ip_address='OT2CEP20210918R04.local')

    hotplate1 = IkaHotplate.RCTDigital5(com_port='COM6')

    hotplate2 = IkaHotplate.RCTDigital5(com_port='COM4')

    hotplate3 = IkaHotplate.RCTDigital5(com_port='COM18')

    hotplate1_clamp = HotplateClamp.HotplateClampDCMotor(arduino_controller=arduino, parent_hardware=hotplate1, clamp_number=1)

    hotplate2_clamp = HotplateClamp.HotplateClampDCMotor(arduino_controller=arduino, parent_hardware=hotplate2, clamp_number=2)

    hotplate3_clamp = HotplateClamp.HotplateClampDCMotor(arduino_controller=arduino, parent_hardware=hotplate3, clamp_number=3)

    hotplate_fan_1 = HotplateFan.HotplateFan(hotplate1, arduino_controller=arduino, fan_number=1)

    hotplate_fan_2 = HotplateFan.HotplateFan(hotplate2, arduino_controller=arduino, fan_number=2)
```

```
    hotplate_fan_3 = HotplateFan.HotplateFan(hotplate3, arduino_controller=arduino,
fan_number=3)

    capper = CapperDecapper.CapperDecapper(arduino_controller=arduino)

    valve1 = SwitchingValve.SwitchingValveVici(com_port='COM3')

    valve2 = SwitchingValve.SwitchingValveVici(com_port='COM14')

    valve3 = SwitchingValve.SwitchingValveVici(com_port='COM17')

    pump1 = WPI.Aladdin(com_port='COM12')

    pump2 = WPI.Aladdin(com_port='COM9', baud_rate=9600,
pump_type=SyringePumpType.AL_1010)

    pump3 = WPI.Aladdin(com_port='COM5', baud_rate=9600,
pump_type=SyringePumpType.AL_1010)

    probesonicator = Hielscher.UP200ST(ip_address='192.168.233.233')

    centrifuge = Herolab.RobotCen(com_port='COM8', initialize_rotor=True,
home_rotor=True)

    zetasizer = MalvernPanalytical.ZetaSizer(local_controller=local_server)



    gripchange_holder =
SampleHolder.SampleHolder(SampleHolder.SampleHolderDefinitions.Corkring_Small,
deck_position=4)

    hotplate1_holder =
SampleHolder.SampleHolder(SampleHolderDefinitions.Ika_100mL_Heating_Block,
parent_hardware=hotplate1, deck_position=1)

    hotplate2_holder =
SampleHolder.SampleHolder(SampleHolderDefinitions.Ika_100mL_Heating_Block,
parent_hardware=hotplate2, deck_position=2)

    hotplate3_holder =
SampleHolder.SampleHolder(SampleHolderDefinitions.Ika_100mL_Heating_Block,
parent_hardware=hotplate3, deck_position=3)

    ot2_holder_15ml =
SampleHolder.SampleHolder(SampleHolder.SampleHolderDefinitions.Opentrons_15mL_Tube_
Rack, parent_hardware=ot2, deck_position=1)

    ot2_holder_50ml =
SampleHolder.SampleHolder(SampleHolder.SampleHolderDefinitions.Opentrons_50mL_Tube_
Rack, parent_hardware=ot2, deck_position=4)

    ot2_holder_flask_10ml =
SampleHolder.SampleHolder(SampleHolder.SampleHolderDefinitions.Opentrons_10mL_Flask
_Rack, parent_hardware=ot2, deck_position=4)

    ot2_holder_flask_25ml =
SampleHolder.SampleHolder(SampleHolder.SampleHolderDefinitions.Opentrons_25mL_Flask
_Rack, parent_hardware=ot2, deck_position=3)

    ot2_holder_flask_100ml =
SampleHolder.SampleHolder(SampleHolder.SampleHolderDefinitions.Opentrons_100mL_Flas
k_Rack, parent_hardware=ot2, deck_position=2)

    falcon_tube_holder_50ml =
SampleHolder.SampleHolder(SampleHolder.SampleHolderDefinitions.Isolab_50mL_Foldable
_Tube_Rack, deck_position=1, leave_even_rows_empty=True)

    falcon_tube_holder_15ml =
SampleHolder.SampleHolder(SampleHolder.SampleHolderDefinitions.Isolab_15mL_Foldable
_Tube_Rack, deck_position=2, leave_even_rows_empty=True)

    flaskstation =
SampleHolder.SampleHolder(SampleHolder.SampleHolderDefinitions.Flask_Station,
deck_position=3, leave_even_rows_empty=False)
```

```
    flaskflush = Container(flaskstation, slot_number=5,
container_type=ContainerTypeCollection.FLASK_100_ML, current_volume=Volume(0,
'mL'), has_stirbar=True, name='flask_flush')

    flask_1_100ml = Container(flaskstation, slot_number=1,
container_type=ContainerTypeCollection.FLASK_100_ML, current_volume=Volume(0,
'mL'), has_stirbar=True, name='flask1')

    flask_2_100ml = Container(flaskstation, slot_number=2,
container_type=ContainerTypeCollection.FLASK_100_ML, current_volume=Volume(0,
'mL'), has_stirbar=True, name='flask2')

    flask_3_100ml = Container(flaskstation, slot_number=3,
container_type=ContainerTypeCollection.FLASK_100_ML, current_volume=Volume(0,
'mL'), has_stirbar=True, name='flask3')

    falcontube1_50ml = Container(falcon_tube_holder_50ml, slot_number=1,
container_type=ContainerTypeCollection.FALCON_TUBE_50_ML, current_volume='0 mL',
is_capped=True)

    falcontube2_50ml = Container(falcon_tube_holder_50ml, slot_number=2,
container_type=ContainerTypeCollection.FALCON_TUBE_50_ML, current_volume='0 mL',
is_capped=True)

    falcontube3_50ml = Container(falcon_tube_holder_50ml, slot_number=4,
container_type=ContainerTypeCollection.FALCON_TUBE_50_ML, current_volume='0 mL',
is_capped=True)

    falcontube4_50ml = Container(falcon_tube_holder_50ml, slot_number=14,
container_type=ContainerTypeCollection.FALCON_TUBE_50_ML, current_volume='0 mL',
is_capped=True)

    falcontube5_50ml = Container(falcon_tube_holder_50ml, slot_number=15,
container_type=ContainerTypeCollection.FALCON_TUBE_50_ML, current_volume='0 mL',
is_capped=True)

    falcontube6_50ml = Container(falcon_tube_holder_50ml, slot_number=17,
container_type=ContainerTypeCollection.FALCON_TUBE_50_ML, current_volume='0 mL',
is_capped=True)

    falcontube_counter_50ml = Container(falcon_tube_holder_50ml, slot_number=16,
container_type=ContainerTypeCollection.FALCON_TUBE_50_ML, current_volume='30 mL',
is_capped=True)

    falcontube_for_cleaning = Container(falcon_tube_holder_50ml, slot_number=3,
container_type=ContainerTypeCollection.FALCON_TUBE_50_ML, current_volume='40 mL',
is_capped=False)


    falcontube1_15ml = Container(falcon_tube_holder_15ml, slot_number=1,
container_type=ContainerTypeCollection.FALCON_TUBE_15_ML, current_volume='0 mL',
is_capped=True)

    falcontube2_15ml = Container(falcon_tube_holder_15ml, slot_number=2,
container_type=ContainerTypeCollection.FALCON_TUBE_15_ML, current_volume='0 mL',
is_capped=True)

    falcontube3_15ml = Container(falcon_tube_holder_15ml, slot_number=3,
container_type=ContainerTypeCollection.FALCON_TUBE_15_ML, current_volume='0 mL',
is_capped=True)

    falcontube_15ml_counter = Container(falcon_tube_holder_15ml, slot_number=4,
container_type=ContainerTypeCollection.FALCON_TUBE_15_ML, current_volume='10 mL',
is_capped=True)


    WasteContainerValve1 = Container(current_hardware=valve1, slot_number=3,
name='Waste_Container', current_volume='2 L', max_volume='5 L')

    WasteContainerValve2 = Container(current_hardware=valve2, slot_number=6,
name='Waste_ContainerValve2', current_volume='0 mL', max_volume='50 mL')

    WasteContainerValve3 = Container(current_hardware=valve3, slot_number=9,
name='Waste_ContainerValve3', current_volume='0 mL', max_volume='20 mL')
```

```
    DLSCell = Container(current_hardware=valve1, slot_number=4, name='DLS_Cell', current_volume='0 mL')

    MilliQ_ContainerValve1 = Container(current_hardware=valve1, slot_number=1, name='MilliQ_ContainerValve1', current_volume='1.2 L')

    MilliQ_ContainerValve2 = Container(current_hardware=valve2, slot_number=4, name='CleaningChemicalValve2', current_volume='500 mL')

    MilliQ_ContainerValve3 = Container(current_hardware=valve3, slot_number=4, name='CleaningChemicalValve3', current_volume='15 mL')

    Ethanol_wash_container = Container(current_hardware=valve1, slot_number=2, name='Ethanol_wash', current_volume='5 L')


    CTABContainer = Container(current_hardware=ot2, deck_position=8, slot_number=7, name='CTAB_Container', current_volume='19 mL', container_type=ContainerTypeCollection.FALCON_TUBE_50_ML)

    NaOHContainer = Container(current_hardware=ot2, deck_position=8, slot_number=1, name='NaOH_Container', current_volume='2.5 mL', container_type=ContainerTypeCollection.FALCON_TUBE_15_ML)

    TEOSContainer = Container(current_hardware=valve2, slot_number=9, name='TEOS_Container', current_volume='4 mL')

    NH4NO3Container = Container(current_hardware=valve1, slot_number=6, name='NH4NO3_Container', current_volume='170 mL')


    NH4NO3 = Chemical(container=NH4NO3Container, volume='25 mL', name='Ammonium nitrate', mass_concentration='20 mg/mL', lookup_missing_values=True)

    Ethanol_wash = Chemical(container=Ethanol_wash_container, name='Ethanol', volume='20 mL', lookup_missing_values=True)

    Water_wash = Chemical(container=MilliQ_ContainerValve1, name='Water', volume='30 mL', lookup_missing_values=True)

    Water_wash_valve2 = Chemical(container=MilliQ_ContainerValve2, name='Water', volume='1 mL', lookup_missing_values=True)


    sonication_time = '90 s'

    washing_steps = 3

    centrifugation_speed = '14000 rcf'

    centrifugation_time = '20 min'

    sample_name1 = 'MZ196'

    sample_name2 = 'MZ197'

    sample_name3 = 'MZ198'


    ot2_configuration = {
        1: ot2_holder_15ml,
        2: ot2_holder_flask_100ml,
        3: ot2_holder_flask_25ml,
        4: ot2_holder_flask_10ml,
        5: None,
        6: None,
        7: None,
        8: 'opentrons_10_tuberack_falcon_4x50ml_6x15ml_conical',
        9: None,
        10:'opentrons_96_tiprack_20ul',    # 'opentrons_96_tiprack_300ul'
        11:'labsolute_96_tiprack_1000ul',
        12:'p1000_single_gen2',  # left pipette
        13:'p20_single_gen2',  # right pipette  'p300_single_gen2'
```

```
    }

    valve1_configuration = {
        0: None,
        1: MilliQ_ContainerValve1,
        2: Ethanol_wash_container,
        3: WasteContainerValve1,
        4: DLSCell,
        5: None,
        6: NH4NO3Container,
        7: None,
        8: 'Outlet',
        9: None,
        10: pump1
    }

    valve2_configuration = {
        0: None,
        1: hotplate1,
        2: hotplate2,
        3: hotplate3,
        4: MilliQ_ContainerValve2,
        5: None,
        6: WasteContainerValve2,
        7: None,
        8: None,
        9: TEOSContainer,
        10: pump2
    }

    valve3_configuration = {
        0: None,
        1: hotplate1,
        2: hotplate2,
        3: hotplate3,
        4: MilliQ_ContainerValve3,
        5: None,
        6: None,
        7: None,
        8: None,
        9: WasteContainerValve3,
        10: pump3
    }

    valve2_dead_volumes = {
        0: None,
        1: '1.390 mL',
        2: '1.35 mL',
        3: '1.87 mL',
        4: None,
        5: None,
        6: None,
        7: None,
        8: None,
        9: None,
        10: None
    }

    ot2.set_hardware_configuration(ot2_configuration)

    pump1.set_syringe(Syringes.GLASS_SYRINGE_SOCOREX_50ML,
default_addition_rate='90 mL/min')    #'70 mL/min' with hose_inner_d = 2.4 mm

    valve1.set_configuration(valve1_configuration)

    pump2.set_syringe(Syringes.GLASS_SYRINGE_SOCOREX_5ML, default_addition_rate='20
mL/min')     # minimum speed = 0.00014 cm/min , 1 mL syringe: 1.46 uL/min , 6.75 mm
diameter
```

```
    valve2.set_configuration(valve2_configuration)

    valve2.set_dead_volumes(valve2_dead_volumes)

    pump3.set_syringe(Syringes.GLASS_SYRINGE_SOCOREX_5ML, default_addition_rate='20 mL/min')

    valve3.set_configuration(valve3_configuration)



    MSN_Synthesis_reaction_parameters = ((flask_1_100ml, falcontube1_50ml, falcontube2_50ml, '1400 uL'),

                                         (flask_2_100ml, falcontube3_50ml, falcontube4_50ml, '200 uL'),

                                         (flask_3_100ml, falcontube5_50ml, falcontube6_50ml, '200 uL'))



    threads = []

    for i, p in enumerate(MSN_Synthesis_reaction_parameters):

        threads.append(threading.Thread(name=f'Thread_{i}', target=MSN_Synthesis, args=tuple(p)))

        threads[-1].start()

        time.sleep(180)



    for t in threads:

        t.join()



    # Washing after synthesis

    for _ in range(0, washing_steps):

        falcontube1_50ml.centrifuge([falcontube2_50ml, falcontube3_50ml, falcontube4_50ml, falcontube5_50ml, falcontube6_50ml], centrifugation_speed=centrifugation_speed, centrifugation_time=centrifugation_time)


falcontube1_50ml.remove_supernatant_and_redisperse(waste_container=WasteContainerValve1, sonication_time=sonication_time, redispersion_chemical=Chemical.from_stock_chemical(Ethanol_wash, volume='15 mL'), sonication_power=50, sonication_amplitude=50, sonicator=probesonicator, bottom_clearance_sonication=15, purging_volume='10 mL', bottom_clearance_withdrawing=8, container_for_cleaning=falcontube_for_cleaning)


falcontube2_50ml.remove_supernatant_and_redisperse(waste_container=WasteContainerValve1, sonication_time=sonication_time, redispersion_chemical=Chemical.from_stock_chemical(Ethanol_wash, volume='15 mL'), sonication_power=50, sonication_amplitude=50, sonicator=probesonicator, bottom_clearance_sonication=15, purging_volume='10 mL', bottom_clearance_withdrawing=8, container_for_cleaning=falcontube_for_cleaning)


falcontube3_50ml.remove_supernatant_and_redisperse(waste_container=WasteContainerValve1, sonication_time=sonication_time, redispersion_chemical=Chemical.from_stock_chemical(Ethanol_wash, volume='15 mL'), sonication_power=50, sonication_amplitude=50, sonicator=probesonicator, bottom_clearance_sonication=15, purging_volume='10 mL', bottom_clearance_withdrawing=8, container_for_cleaning=falcontube_for_cleaning)


falcontube4_50ml.remove_supernatant_and_redisperse(waste_container=WasteContainerValve1, sonication_time=sonication_time, redispersion_chemical=Chemical.from_stock_chemical(Ethanol_wash, volume='15 mL'),
```

```
sonication_power=50, sonication_amplitude=50, sonicator=probesonicator,
bottom_clearance_sonication=15, purging_volume='10 mL',
bottom_clearance_withdrawing=8, container_for_cleaning=falcontube_for_cleaning)


falcontube5_50ml.remove_supernatant_and_redisperse(waste_container=WasteContainerVa
lve1, sonication_time=sonication_time,
redispersion_chemical=Chemical.from_stock_chemical(Ethanol_wash, volume='15 mL'),
sonication_power=50, sonication_amplitude=50, sonicator=probesonicator,
bottom_clearance_sonication=15, purging_volume='10 mL',
bottom_clearance_withdrawing=8, container_for_cleaning=falcontube_for_cleaning)


falcontube6_50ml.remove_supernatant_and_redisperse(waste_container=WasteContainerVa
lve1, sonication_time=sonication_time,
redispersion_chemical=Chemical.from_stock_chemical(Ethanol_wash, volume='15 mL'),
sonication_power=50, sonication_amplitude=50, sonicator=probesonicator,
bottom_clearance_sonication=15, purging_volume='10 mL',
bottom_clearance_withdrawing=8, container_for_cleaning=falcontube_for_cleaning)

    centrifuge.close_lid()


    # Redispersion in ammonium nitrate

    falcontube1_50ml.centrifuge([falcontube2_50ml, falcontube3_50ml,
falcontube4_50ml, falcontube5_50ml, falcontube6_50ml],
centrifugation_speed=centrifugation_speed, centrifugation_time=centrifugation_time)


falcontube1_50ml.remove_supernatant_and_redisperse(waste_container=WasteContainerVa
lve1, sonication_time=sonication_time, redispersion_chemical=NH4NO3,
sonication_power=50, sonication_amplitude=50, sonicator=probesonicator,
bottom_clearance_sonication=13, purging_volume='10 mL',
bottom_clearance_withdrawing=8, container_for_cleaning=None)


falcontube2_50ml.remove_supernatant_and_redisperse(waste_container=WasteContainerVa
lve1, sonication_time=sonication_time, redispersion_chemical=NH4NO3,
sonication_power=50, sonication_amplitude=50, sonicator=probesonicator,
bottom_clearance_sonication=13, purging_volume='10 mL',
bottom_clearance_withdrawing=8, container_for_cleaning=falcontube_for_cleaning)


falcontube3_50ml.remove_supernatant_and_redisperse(waste_container=WasteContainerVa
lve1, sonication_time=sonication_time, redispersion_chemical=NH4NO3,
sonication_power=50, sonication_amplitude=50, sonicator=probesonicator,
bottom_clearance_sonication=13, purging_volume='10 mL',
bottom_clearance_withdrawing=8, container_for_cleaning=None)


falcontube4_50ml.remove_supernatant_and_redisperse(waste_container=WasteContainerVa
lve1, sonication_time=sonication_time, redispersion_chemical=NH4NO3,
sonication_power=50, sonication_amplitude=50, sonicator=probesonicator,
bottom_clearance_sonication=13, purging_volume='10 mL',
bottom_clearance_withdrawing=8, container_for_cleaning=falcontube_for_cleaning)


falcontube5_50ml.remove_supernatant_and_redisperse(waste_container=WasteContainerVa
lve1, sonication_time=sonication_time, redispersion_chemical=NH4NO3,
sonication_power=50, sonication_amplitude=50, sonicator=probesonicator,
bottom_clearance_sonication=13, purging_volume='10 mL',
bottom_clearance_withdrawing=8, container_for_cleaning=None)


falcontube6_50ml.remove_supernatant_and_redisperse(waste_container=WasteContainerVa
lve1, sonication_time=sonication_time, redispersion_chemical=NH4NO3,
sonication_power=50, sonication_amplitude=50, sonicator=probesonicator,
bottom_clearance_sonication=13, purging_volume='10 mL',
bottom_clearance_withdrawing=8, container_for_cleaning=falcontube_for_cleaning)


    #extraction protocol
```

```
    NH4NO3_Extraction_parameters = ((flask_1_100ml, falcontube1_50ml,
falcontube2_50ml),

    (flask_2_100ml, falcontube3_50ml, falcontube4_50ml), (flask_3_100ml,
falcontube5_50ml, falcontube6_50ml))


    threads = []

    for i, p in enumerate(NH4NO3_Extraction_parameters):

         threads.append(threading.Thread(name=f'Thread_{i}',
target=NH4NO3_extraction, args=tuple(p)))

         threads[-1].start()

         time.sleep(450)


    for t in threads:

        t.join()


    # Washing after ammonium nitrate extraction

    for _ in range(0, washing_steps):

        falcontube1_50ml.centrifuge([falcontube2_50ml, falcontube3_50ml,
falcontube4_50ml, falcontube5_50ml, falcontube6_50ml],
centrifugation_speed=centrifugation_speed, centrifugation_time=centrifugation_time)


falcontube1_50ml.remove_supernatant_and_redisperse(waste_container=WasteContainerVa
lve1, sonication_time=sonication_time,
redispersion_chemical=Chemical.from_stock_chemical(Ethanol_wash, volume='15 mL'),
sonication_power=50, sonication_amplitude=50, sonicator=probesonicator,
bottom_clearance_sonication=15, purging_volume='10 mL',
bottom_clearance_withdrawing=8, container_for_cleaning=None)


falcontube2_50ml.remove_supernatant_and_redisperse(waste_container=WasteContainerVa
lve1, sonication_time=sonication_time,
redispersion_chemical=Chemical.from_stock_chemical(Ethanol_wash, volume='15 mL'),
sonication_power=50, sonication_amplitude=50, sonicator=probesonicator,
bottom_clearance_sonication=15, purging_volume='10 mL',
bottom_clearance_withdrawing=8, container_for_cleaning=falcontube_for_cleaning)


falcontube3_50ml.remove_supernatant_and_redisperse(waste_container=WasteContainerVa
lve1, sonication_time=sonication_time,
redispersion_chemical=Chemical.from_stock_chemical(Ethanol_wash, volume='15 mL'),
sonication_power=50, sonication_amplitude=50, sonicator=probesonicator,
bottom_clearance_sonication=15, purging_volume='10 mL',
bottom_clearance_withdrawing=8, container_for_cleaning=None)


falcontube4_50ml.remove_supernatant_and_redisperse(waste_container=WasteContainerVa
lve1, sonication_time=sonication_time,
redispersion_chemical=Chemical.from_stock_chemical(Ethanol_wash, volume='15 mL'),
sonication_power=50, sonication_amplitude=50, sonicator=probesonicator,
bottom_clearance_sonication=15, purging_volume='10 mL',
bottom_clearance_withdrawing=8, container_for_cleaning=falcontube_for_cleaning)


falcontube5_50ml.remove_supernatant_and_redisperse(waste_container=WasteContainerVa
lve1, sonication_time=sonication_time,
redispersion_chemical=Chemical.from_stock_chemical(Ethanol_wash, volume='15 mL'),
sonication_power=50, sonication_amplitude=50, sonicator=probesonicator,
bottom_clearance_sonication=15, purging_volume='10 mL',
bottom_clearance_withdrawing=8, container_for_cleaning=None)
```

```
falcontube6_50ml.remove_supernatant_and_redisperse(waste_container=WasteContainerValve1, sonication_time=sonication_time,
redispersion_chemical=Chemical.from_stock_chemical(Ethanol_wash, volume='15 mL'),
sonication_power=50, sonication_amplitude=50, sonicator=probesonicator,
bottom_clearance_sonication=15, purging_volume='10 mL',
bottom_clearance_withdrawing=8, container_for_cleaning=falcontube_for_cleaning)

    centrifuge.close_lid()


    # Redispersion in water for DLS

    falcontube1_50ml.transfer_content_to_container(falcontube1_15ml, volume='2 mL',
bottom_clearance_withdrawing=15, transfer_hardware=valve1, purging_volume='10 mL')

    falcontube1_15ml.add_chemical([Chemical.from_stock_chemical(Ethanol_wash,
volume='8 mL')])

    falcontube3_50ml.transfer_content_to_container(falcontube2_15ml, volume='2 mL',
bottom_clearance_withdrawing=15, transfer_hardware=valve1, purging_volume='10 mL')

    falcontube2_15ml.add_chemical([Chemical.from_stock_chemical(Ethanol_wash,
volume='8 mL')])

    falcontube5_50ml.transfer_content_to_container(falcontube3_15ml, volume='2 mL',
bottom_clearance_withdrawing=15, transfer_hardware=valve1, purging_volume='10 mL')

    falcontube3_15ml.add_chemical([Chemical.from_stock_chemical(Ethanol_wash,
volume='8 mL')])


    falcontube1_15ml.centrifuge(containers=[falcontube2_15ml, falcontube3_15ml,
falcontube4_15ml], centrifugation_time='12 min',
centrifugation_speed=centrifugation_speed)


falcontube1_15ml.remove_supernatant_and_redisperse(waste_container=WasteContainerValve1, sonication_time='30 s',
redispersion_chemical=Chemical.from_stock_chemical(Water_wash, volume='10 mL'),
sonication_power=50, sonication_amplitude=50, sonicator=probesonicator,
bottom_clearance_sonication=20, purging_volume='10 mL',
bottom_clearance_withdrawing=2, container_for_cleaning=falcontube_for_cleaning)


falcontube2_15ml.remove_supernatant_and_redisperse(waste_container=WasteContainerValve1, sonication_time='30 s',
redispersion_chemical=Chemical.from_stock_chemical(Water_wash, volume='10 mL'),
sonication_power=50, sonication_amplitude=50, sonicator=probesonicator,
bottom_clearance_sonication=20, purging_volume='10 mL',
bottom_clearance_withdrawing=2, container_for_cleaning=falcontube_for_cleaning)


falcontube3_15ml.remove_supernatant_and_redisperse(waste_container=WasteContainerValve1, sonication_time='30 s',
redispersion_chemical=Chemical.from_stock_chemical(Water_wash, volume='10 mL'),
sonication_power=50, sonication_amplitude=50, sonicator=probesonicator,
bottom_clearance_sonication=20, purging_volume='10 mL',
bottom_clearance_withdrawing=2, container_for_cleaning=falcontube_for_cleaning)


    # Measure DLS

    falcontube1_15ml.measure_dls(dls_cell=DLSCell, sop_path=os.path.join('C:\\',
'users', 'WS8717-apollo', 'Desktop', 'Apollo', 'Characterization', 'DLS', 'SOP',
'DLS_ZETA_SiO2_in_Water.zskd'), sample_name=f"{sample_name1}" ,
chemical_for_cleaning=Chemical.from_stock_chemical(Water_wash, volume='10 mL'),
dls_device=zetasizer, bottom_clearance_sampling=20,
waste_container=WasteContainerValve1, dls_volume='3 mL', dead_volume_dls='5.6 mL')

    falcontube2_15ml.measure_dls(dls_cell=DLSCell, sop_path=os.path.join('C:\\',
'users', 'WS8717-apollo', 'Desktop', 'Apollo', 'Characterization', 'DLS', 'SOP',
'DLS_ZETA_SiO2_in_Water.zskd'), sample_name=f"{sample_name2}" ,
```

```
chemical_for_cleaning=Chemical.from_stock_chemical(Water_wash, volume='10 mL'), dls_device=zetasizer, bottom_clearance_sampling=20, waste_container=WasteContainerValve1, dls_volume='3 mL', dead_volume_dls='5.6 mL')

    falcontube3_15ml.measure_dls(dls_cell=DLSCell, sop_path=os.path.join('C:\\', 'users', 'WS8717-apollo', 'Desktop', 'Apollo', 'Characterization', 'DLS', 'SOP', 'DLS_ZETA_SiO2_in_Water.zskd'), sample_name=f"{sample_name3}" , chemical_for_cleaning=Chemical.from_stock_chemical(Water_wash, volume='10 mL'), dls_device=zetasizer, bottom_clearance_sampling=20, waste_container=WasteContainerValve1, dls_volume='3 mL', dead_volume_dls='5.6 mL')


    #ELN upload


eln.write_synthesis_step(experiment_name=f"{sample_name1},{sample_name2},{sample_name3}")
```

4.4. ZIF-8

```
import time
import datetime
import os
import threading

import API.MinervaAPI
import Minerva.Software.OpenBISELNIntegration.OpenBisElnIntegration
from Minerva import *
from Minerva.API.HelperClassDefinitions import PathNames


def ZIF8_Synthesis(container: Container, falcontube1: Container, falcontube2: Container):

    MIM = Chemical(container=MIMContainer, name='2MethylImidazole', mass_concentration='32.8 mg/mL', mass='0.82 g', lookup_missing_values=True)

    Zinc_Nitrate = Chemical(container=ZnNO3Container, name='ZincNitrate', mass_concentration='29.7 mg/mL', mass='0.7425 g', lookup_missing_values=True)


    stirring_speed = 500  # rpm
    reaction_temperature = 24  # °C
    reaction_time = '24 h'
    temperature_error = 2  # °C
    cooling_temperature = 30  # °C
    MIM_infusion_rate = '18 mL/min'


    container.add_chemical(Zinc_Nitrate, priming_volume='2 mL', priming_waste_container=WasteContainerValve1)


    container.infuse_while_heating(chemical=[MIM],
                                   heating_temperature=reaction_temperature,
                                   stirring_speed=stirring_speed,
                                   heating_time=reaction_time,
                                   addition_hardware=valve2,
                                   addition_rate=MIM_infusion_rate,
                                   priming_volume='2 mL',
                                   purging_volume='10 mL',
                                   purging_port=5,
```

```
                                          priming_waste_container=WasteContainerValve2,
                                          cooldown_temperature=cooling_temperature,
                                          maximum_temperature_deviation=temperature_error,
                                          temperature_stabilization_time='5 min',
                                          chemical_for_cleaning=Water_wash_valve2,
                                          active_cooling=True)

    container.transfer_content_to_container(target_containers=[falcontube1,
falcontube2], transfer_hardware=valve1, bottom_clearance_withdrawing=7,
dropoff_locations=[(flaskstation, 0), (falcon_tube_holder_50ml, 0),
(falcon_tube_holder_50ml, 0)], purging_volume='15 mL')


if __name__ == '__main__':
    esb = EmergencyStopButton.EmergencyStopButton(com_port='COM34')
    eln =
Minerva.Software.OpenBISELNIntegration.OpenBisElnIntegration.ElectronicLabNotebook(
space_name='1.0_MINERVA', project_code='MOF')
    local_server = LocalPCServer.LocalPCServer(com_port='COM7')
    arduino = ArduinoController.ArduinoController(com_port='COM25')
    dht22 = DHT22Sensor.DHT22Sensor(arduino_controller=arduino)
    robotarm = UFactory.XArm6(ip_address='192.168.1.204',
levelling_data_file=os.path.join('..', 'SampleHolder',
'Table_Levelling_Data.json'))
    ot2 = OpentronsOT2.OT2(ip_address='OT2CEP20210918R04.local')
    hotplate1 = IkaHotplate.RCTDigital5(com_port='COM6')
    hotplate2 = IkaHotplate.RCTDigital5(com_port='COM4')
    hotplate3 = IkaHotplate.RCTDigital5(com_port='COM18')
    hotplate1_clamp =
HotplateClamp.HotplateClampDCMotor(arduino_controller=arduino,
parent_hardware=hotplate1, clamp_number=1)
    hotplate2_clamp =
HotplateClamp.HotplateClampDCMotor(arduino_controller=arduino,
parent_hardware=hotplate2, clamp_number=2)
    hotplate3_clamp =
HotplateClamp.HotplateClampDCMotor(arduino_controller=arduino,
parent_hardware=hotplate3, clamp_number=3)
    hotplate_fan_1 = HotplateFan.HotplateFan(hotplate1, arduino_controller=arduino,
fan_number=1)
    hotplate_fan_2 = HotplateFan.HotplateFan(hotplate2, arduino_controller=arduino,
fan_number=2)
    hotplate_fan_3 = HotplateFan.HotplateFan(hotplate3, arduino_controller=arduino,
fan_number=3)
    capper = CapperDecapper.CapperDecapper(arduino_controller=arduino)
    valve1 = SwitchingValve.SwitchingValveVici(com_port='COM3')
    valve2 = SwitchingValve.SwitchingValveVici(com_port='COM14')
    valve3 = SwitchingValve.SwitchingValveVici(com_port='COM17')
    pump1 = WPI.Aladdin(com_port='COM12')
```

```
    pump2 = WPI.Aladdin(com_port='COM9', baud_rate=9600,
pump_type=SyringePumpType.AL_1010)

    pump3 = WPI.Aladdin(com_port='COM5', baud_rate=9600,
pump_type=SyringePumpType.AL_1010)

    probesonicator = Hielscher.UP200ST(ip_address='192.168.233.233')

    centrifuge = Herolab.RobotCen(com_port='COM8', initialize_rotor=True,
home_rotor=True)

    zetasizer = MalvernPanalytical.ZetaSizer(local_controller=local_server)



    gripchange_holder =
SampleHolder.SampleHolder(SampleHolder.SampleHolderDefinitions.Corkring_Small,
deck_position=4)

    hotplate1_holder =
SampleHolder.SampleHolder(SampleHolderDefinitions.Ika_100mL_Heating_Block,
parent_hardware=hotplate1, deck_position=1)

    hotplate2_holder =
SampleHolder.SampleHolder(SampleHolderDefinitions.Ika_100mL_Heating_Block,
parent_hardware=hotplate2, deck_position=2)

    hotplate3_holder =
SampleHolder.SampleHolder(SampleHolderDefinitions.Ika_100mL_Heating_Block,
parent_hardware=hotplate3, deck_position=3)

    ot2_holder_15ml =
SampleHolder.SampleHolder(SampleHolder.SampleHolderDefinitions.Opentrons_15mL_Tube_
Rack, parent_hardware=ot2, deck_position=1)

    ot2_holder_50ml =
SampleHolder.SampleHolder(SampleHolder.SampleHolderDefinitions.Opentrons_50mL_Tube_
Rack, parent_hardware=ot2, deck_position=4)

    ot2_holder_flask_25ml =
SampleHolder.SampleHolder(SampleHolder.SampleHolderDefinitions.Opentrons_25mL_Flask
_Rack, parent_hardware=ot2, deck_position=3)

    ot2_holder_flask_100ml =
SampleHolder.SampleHolder(SampleHolder.SampleHolderDefinitions.Opentrons_100mL_Flas
k_Rack, parent_hardware=ot2, deck_position=2)

    falcon_tube_holder_50ml =
SampleHolder.SampleHolder(SampleHolder.SampleHolderDefinitions.Isolab_50mL_Foldable
_Tube_Rack, deck_position=1, leave_even_rows_empty=True)

    falcon_tube_holder_15ml =
SampleHolder.SampleHolder(SampleHolder.SampleHolderDefinitions.Isolab_15mL_Foldable
_Tube_Rack, deck_position=2, leave_even_rows_empty=True)

    flaskstation =
SampleHolder.SampleHolder(SampleHolder.SampleHolderDefinitions.Flask_Station,
deck_position=3, leave_even_rows_empty=False)



    flaskflush = Container(flaskstation, slot_number=5,
container_type=ContainerTypeCollection.FLASK_100_ML, current_volume=Volume(0,
'mL'), has_stirbar=True, name='flask_flush')

    flask_1_100ml = Container(flaskstation, slot_number=1,
container_type=ContainerTypeCollection.FLASK_100_ML, current_volume=Volume(0,
'mL'), has_stirbar=True, name='flask1')

    flask_2_100ml = Container(flaskstation, slot_number=2,
container_type=ContainerTypeCollection.FLASK_100_ML, current_volume=Volume(0,
'mL'), has_stirbar=True, name='flask2')

    flask_3_100ml = Container(flaskstation, slot_number=3,
container_type=ContainerTypeCollection.FLASK_100_ML, current_volume=Volume(0,
'mL'), has_stirbar=True, name='flask3')
```

```
    falcontube1_50ml = Container(falcon_tube_holder_50ml, slot_number=1,
container_type=ContainerTypeCollection.FALCON_TUBE_50_ML, current_volume='0 mL',
is_capped=True)

    falcontube2_50ml = Container(falcon_tube_holder_50ml, slot_number=2,
container_type=ContainerTypeCollection.FALCON_TUBE_50_ML, current_volume='0 mL',
is_capped=True)

    falcontube3_50ml = Container(falcon_tube_holder_50ml, slot_number=4,
container_type=ContainerTypeCollection.FALCON_TUBE_50_ML, current_volume='0 mL',
is_capped=True)

    falcontube4_50ml = Container(falcon_tube_holder_50ml, slot_number=14,
container_type=ContainerTypeCollection.FALCON_TUBE_50_ML, current_volume='0 mL',
is_capped=True)

    falcontube5_50ml = Container(falcon_tube_holder_50ml, slot_number=15,
container_type=ContainerTypeCollection.FALCON_TUBE_50_ML, current_volume='0 mL',
is_capped=True)

    falcontube6_50ml = Container(falcon_tube_holder_50ml, slot_number=17,
container_type=ContainerTypeCollection.FALCON_TUBE_50_ML, current_volume='0 mL',
is_capped=True)


    falcontube1_15ml = Container(falcon_tube_holder_15ml, slot_number=1,
container_type=ContainerTypeCollection.FALCON_TUBE_15_ML, current_volume='0 mL',
is_capped=True)

    falcontube2_15ml = Container(falcon_tube_holder_15ml, slot_number=2,
container_type=ContainerTypeCollection.FALCON_TUBE_15_ML, current_volume='0 mL',
is_capped=True)

    falcontube3_15ml = Container(falcon_tube_holder_15ml, slot_number=3,
container_type=ContainerTypeCollection.FALCON_TUBE_15_ML, current_volume='0 mL',
is_capped=True)

    falcontube_15ml_counter = Container(falcon_tube_holder_15ml, slot_number=4,
container_type=ContainerTypeCollection.FALCON_TUBE_15_ML, current_volume='10 mL',
is_capped=True)


    falcontube_for_cleaning = Container(falcon_tube_holder_50ml, slot_number=3,
container_type=ContainerTypeCollection.FALCON_TUBE_50_ML, current_volume='40 mL',
is_capped=False)


    WasteContainerValve1 = Container(current_hardware=valve1, slot_number=3,
name='Waste_Container', current_volume='2 L', max_volume='5 L')

    WasteContainerValve2 = Container(current_hardware=valve2, slot_number=6,
name='Waste_ContainerValve2', current_volume='0 mL', max_volume='50 mL')

    WasteContainerValve3 = Container(current_hardware=valve3, slot_number=9,
name='Waste_ContainerValve3', current_volume='0 mL', max_volume='20 mL')

    DLSCell = Container(current_hardware=valve1, slot_number=4, name='DLS_Cell',
current_volume='0 mL')

    MilliQ_ContainerValve1 = Container(current_hardware=valve1, slot_number=1,
name='MilliQ_ContainerValve1', current_volume='1.2 L')

    MilliQ_ContainerValve2 = Container(current_hardware=valve2, slot_number=4,
name='CleaningChemicalValve2', current_volume='500 mL')

    MilliQ_ContainerValve3 = Container(current_hardware=valve3, slot_number=4,
name='CleaningChemicalValve3', current_volume='15 mL')


    Methanol_wash_container = Container(current_hardware=valve1, slot_number=7,
name='Methanol_wash', current_volume='2 L')
```

```
    MIMContainer = Container(current_hardware=valve2, slot_number=9,
name='MIM_Container', current_volume='1 L')

    ZnNO3Container = Container(current_hardware=valve1, slot_number=6,
name='ZnNO3_Container', current_volume='1 L')



    Zinc_Nitrate = Chemical(container=ZnNO3Container, name='ZincNitrate',
mass_concentration='29.7 mg/mL', mass='0.7425 g', lookup_missing_values=True)

    Water_wash_valve2 = Chemical(container=MilliQ_ContainerValve2, name='Water',
volume='2 mL', lookup_missing_values=False)

    Methanol_wash = Chemical(container=Methanol_wash_container, name='Methanol',
volume='15 mL', lookup_missing_values=True)

    Water_wash = Chemical(container=MilliQ_ContainerValve1, name='Water',
volume='30 mL', lookup_missing_values=True)



    sonication_time = '25 s'
    washing_steps = 4
    centrifugation_speed = '9000 rcf'
    centrifugation_time = '15 min'
    sample_name1 = 'MZ215'
    sample_name2 = 'MZ216'
    sample_name3 = 'MZ217'


    ot2_configuration = {
        1: ot2_holder_15ml,
        2: ot2_holder_flask_100ml,
        3: ot2_holder_flask_25ml,
        4: None,
        5: None,
        6: None,
        7: None,
        8: 'opentrons_10_tuberack_falcon_4x50ml_6x15ml_conical',
        9: None,
        10:'opentrons_96_tiprack_20ul',    # 'opentrons_96_tiprack_300ul'
        11:'labsolute_96_tiprack_1000ul',
        12:'p1000_single_gen2',  # left pipette
        13:'p20_single_gen2',  # right pipette  'p300_single_gen2'
    }


    valve1_configuration = {
        0: None,
        1: MilliQ_ContainerValve1,
        2: None,
        3: WasteContainerValve1,
        4: DLSCell,
        5: None,
        6: ZnNO3Container,
        7: Methanol_wash_container,
        8: 'Outlet',
        9: None,
        10: pump1
    }


    valve2_configuration = {
        0: None,
        1: hotplate1,
        2: hotplate2,
        3: hotplate3,
        4: MilliQ_ContainerValve2,
        5: None,
        6: WasteContainerValve2,
        7: None,
        8: None,
        9: MIMContainer,
```

```
        10: pump2
    }


    valve3_configuration = {
        0: None,
        1: hotplate1,
        2: hotplate2,
        3: hotplate3,
        4: MilliQ_ContainerValve3,
        5: None,
        6: None,
        7: None,
        8: None,
        9: WasteContainerValve3,
        10: pump3
    }


    valve2_dead_volumes = {
        0: None,
        1: '1.390 mL',
        2: '1.35 mL',
        3: '1.87 mL',
        4: None,
        5: None,
        6: None,
        7: None,
        8: None,
        9: None,
        10: None
    }


    ot2.set_hardware_configuration(ot2_configuration)

    pump1.set_syringe(Syringes.GLASS_SYRINGE_SOCOREX_50ML,
default_addition_rate='70 mL/min')    #'70 mL/min' with hose_inner_d = 2.4 mm

    valve1.set_configuration(valve1_configuration)

    pump2.set_syringe(Syringes.GLASS_SYRINGE_SOCOREX_50ML,
default_addition_rate='50 mL/min')     # minimum speed = 0.00014 cm/min , 1 mL
syringe: 1.46 uL/min , 6.75 mm diameter

    valve2.set_configuration(valve2_configuration)

    valve2.set_dead_volumes(valve2_dead_volumes)

    pump3.set_syringe(Syringes.GLASS_SYRINGE_SOCOREX_5ML, default_addition_rate='20
mL/min')

    valve3.set_configuration(valve3_configuration)



    ZIF8_Synthesis_parameters = ((flask_1_100ml, falcontube1_50ml,
falcontube2_50ml),

    (flask_2_100ml, falcontube3_50ml, falcontube4_50ml), (flask_3_100ml,
falcontube5_50ml, falcontube6_50ml))



    threads = []

    for i, p in enumerate(ZIF8_Synthesis_parameters):

        threads.append(threading.Thread(name=f'Thread_{i}', target=ZIF8_Synthesis,
args=tuple(p)))

        threads[-1].start()

        time.sleep(60)
```

```
    for t in threads:
        t.join()

    # Washing after synthesis
    for _ in range(0, washing_steps):
        falcontube1_50ml.centrifuge([falcontube2_50ml, falcontube3_50ml, falcontube4_50ml, falcontube5_50ml, falcontube6_50ml], centrifugation_speed=centrifugation_speed, centrifugation_time=centrifugation_time, centrifugation_temperature=25)

falcontube1_50ml.remove_supernatant_and_redisperse(waste_container=WasteContainerValve1, sonication_time=sonication_time, redispersion_chemical=Methanol_wash, sonication_power=20, sonication_amplitude=20, sonicator=probesonicator, bottom_clearance_sonication=15, purging_volume='10 mL', bottom_clearance_withdrawing=8, container_for_cleaning=None)

falcontube2_50ml.remove_supernatant_and_redisperse(waste_container=WasteContainerValve1, sonication_time=sonication_time, redispersion_chemical=Methanol_wash, sonication_power=20, sonication_amplitude=20, sonicator=probesonicator, bottom_clearance_sonication=15, purging_volume='10 mL', bottom_clearance_withdrawing=8, container_for_cleaning=falcontube_for_cleaning)

falcontube3_50ml.remove_supernatant_and_redisperse(waste_container=WasteContainerValve1, sonication_time=sonication_time, redispersion_chemical=Methanol_wash, sonication_power=20, sonication_amplitude=20, sonicator=probesonicator, bottom_clearance_sonication=15, purging_volume='10 mL', bottom_clearance_withdrawing=8, container_for_cleaning=None)

falcontube4_50ml.remove_supernatant_and_redisperse(waste_container=WasteContainerValve1, sonication_time=sonication_time, redispersion_chemical=Methanol_wash, sonication_power=20, sonication_amplitude=20, sonicator=probesonicator, bottom_clearance_sonication=15, purging_volume='10 mL', bottom_clearance_withdrawing=8, container_for_cleaning=falcontube_for_cleaning)

falcontube5_50ml.remove_supernatant_and_redisperse(waste_container=WasteContainerValve1, sonication_time=sonication_time, redispersion_chemical=Methanol_wash, sonication_power=20, sonication_amplitude=20, sonicator=probesonicator, bottom_clearance_sonication=15, purging_volume='10 mL', bottom_clearance_withdrawing=8, container_for_cleaning=None)

falcontube6_50ml.remove_supernatant_and_redisperse(waste_container=WasteContainerValve1, sonication_time=sonication_time, redispersion_chemical=Methanol_wash, sonication_power=20, sonication_amplitude=20, sonicator=probesonicator, bottom_clearance_sonication=15, purging_volume='10 mL', bottom_clearance_withdrawing=8, container_for_cleaning=falcontube_for_cleaning)
    centrifuge.close_lid()

    # Dilution for DLS
    falcontube1_50ml.transfer_content_to_container(falcontube1_15ml, transfer_hardware=valve1, volume='1 mL', bottom_clearance_withdrawing=10)
    falcontube1_15ml.add_chemical(chemical=Chemical.from_stock_chemical(Water_wash, volume='9 mL'))
    falcontube3_50ml.transfer_content_to_container(falcontube2_15ml, transfer_hardware=valve1, volume='1 mL', bottom_clearance_withdrawing=10)
    falcontube2_15ml.add_chemical(chemical=Chemical.from_stock_chemical(Water_wash, volume='9 mL'))
```

```
    falcontube5_50ml.transfer_content_to_container(falcontube3_15ml,
transfer_hardware=valve1, volume='1 mL', bottom_clearance_withdrawing=10)

    falcontube3_15ml.add_chemical(chemical=Chemical.from_stock_chemical(Water_wash,
volume='9 mL'))



    # DLS

    falcontube1_15ml.measure_dls(dls_cell=DLSCell, sop_path=os.path.join('C:\\',
'users', 'WS8717-apollo', 'Desktop', 'Apollo', 'Characterization', 'DLS', 'SOP',
'DLS_ZIF8_in_methanol.zskd'), sample_name=f"{sample_name1}_MSN" ,
chemical_for_cleaning=Chemical.from_stock_chemical(Water_wash, volume='10 mL'),
dls_device=zetasizer, bottom_clearance_sampling=20,
waste_container=WasteContainerValve1, dls_volume='3 mL', dead_volume_dls='5.6 mL')

    falcontube2_15ml.measure_dls(dls_cell=DLSCell, sop_path=os.path.join('C:\\',
'users', 'WS8717-apollo', 'Desktop', 'Apollo', 'Characterization', 'DLS', 'SOP',
'DLS_ZIF8_in_methanol.zskd'), sample_name=f"{sample_name1}_MSN" ,
chemical_for_cleaning=Chemical.from_stock_chemical(Water_wash, volume='10 mL'),
dls_device=zetasizer, bottom_clearance_sampling=20,
waste_container=WasteContainerValve1, dls_volume='3 mL', dead_volume_dls='5.6 mL')

    falcontube3_15ml.measure_dls(dls_cell=DLSCell, sop_path=os.path.join('C:\\',
'users', 'WS8717-apollo', 'Desktop', 'Apollo', 'Characterization', 'DLS', 'SOP',
'DLS_ZIF8_in_methanol.zskd'), sample_name=f"{sample_name1}_MSN" ,
chemical_for_cleaning=Chemical.from_stock_chemical(Water_wash, volume='10 mL'),
dls_device=zetasizer, bottom_clearance_sampling=20,
waste_container=WasteContainerValve1, dls_volume='3 mL', dead_volume_dls='5.6 mL')



    #ELN upload


eln.write_synthesis_step(experiment_name=f"{sample_name1},{sample_name2},{sample_na
me3}")
```

4.5. CuO NPs

```
import time
import os
import threading

import API.MinervaAPI
import Minerva.Software.OpenBISELNIntegration.OpenBisElnIntegration
from Minerva import *
from Minerva.API.HelperClassDefinitions import PathNames


def CuO_Synthesis(container: Container, falcontube1: Container, falcontube2:
Container, priming_volume):



    CuOAc = Chemical(container=CuOAcContainer, name='Cu(OAc)2.H2O', volume='3.2
mL', concentration='0.25 M', lookup_missing_values=True)

    AcOH = Chemical(container=AcOHContainer, name='AcOH', volume='66.7 uL',
lookup_missing_values=True)

    NaOH = Chemical(container=NaOHContainer, name='NaOH', volume='2.67 mL',
concentration='1 M', lookup_missing_values=True)

    Water = Chemical(container=MilliQ_ContainerValve1, name='Water', volume='40.1
mL', lookup_missing_values=True)

    Water_wash_valve2 = Chemical(container=MilliQ_ContainerValve2, name='Water',
volume='3 mL', lookup_missing_values=True)
```

```
    heating_time = '20 min'
    temperature_error = 2  # °C
    reaction_temperature = 95  # °C
    cooling_temperature = 35  # °C
    stirring_speed = 700 # rpm
    NaOH_addition_rate = '1 mL/min'


    container.add_chemical([Water, CuOAc, AcOH])


    container.infuse_while_heating(chemical=NaOH,
                                   heating_temperature=reaction_temperature,
                                   stirring_speed=stirring_speed,
                                   heating_time=heating_time,
                                   addition_hardware=valve2,
                                   withdraw_rate='10 mL/min',
                                   addition_rate=NaOH_addition_rate,
                                   purging_addition_rate='10 mL/min',
                                   priming_volume=priming_volume,
                                   purging_volume='2.5 mL',
                                   purging_port=5,
                                   priming_waste_container=WasteContainerValve2,
                                   cooldown_temperature=cooling_temperature,
                                   maximum_temperature_deviation=temperature_error,
                                   temperature_stabilization_time='5 min',
                                   chemical_for_cleaning=Water_wash_valve2,
                                   active_cooling=True)


    container.transfer_content_to_container(target_containers=[falcontube1,
falcontube2], transfer_hardware=valve1, bottom_clearance_withdrawing=5,
dropoff_locations=[(flaskstation, 0), (falcon_tube_holder_50ml, 0),
(falcon_tube_holder_50ml, 0)])




if __name__ == '__main__':

    esb = EmergencyStopButton.EmergencyStopButton(com_port='COM34')

    eln =
Minerva.Software.OpenBISELNIntegration.OpenBisElnIntegration.ElectronicLabNotebook(
space_name='1.0_MINERVA', project_code='CuO')

    local_server = LocalPCServer.LocalPCServer(com_port='COM7')

    arduino = ArduinoController.ArduinoController(com_port='COM25')

    dht22 = DHT22Sensor.DHT22Sensor(arduino_controller=arduino)

    robotarm = UFactory.XArm6(ip_address='192.168.1.204',
levelling_data_file=os.path.join('..', 'SampleHolder',
'Table_Levelling_Data.json'))

    ot2 = OpentronsOT2.OT2(ip_address='OT2CEP20210918R04.local')

    hotplate1 = IkaHotplate.RCTDigital5(com_port='COM6')
```

```
    hotplate2 = IkaHotplate.RCTDigital5(com_port='COM4')

    hotplate3 = IkaHotplate.RCTDigital5(com_port='COM18')

    hotplate1_clamp =
HotplateClamp.HotplateClampDCMotor(arduino_controller=arduino,
parent_hardware=hotplate1, clamp_number=1)

    hotplate2_clamp =
HotplateClamp.HotplateClampDCMotor(arduino_controller=arduino,
parent_hardware=hotplate2, clamp_number=2)

    hotplate3_clamp =
HotplateClamp.HotplateClampDCMotor(arduino_controller=arduino,
parent_hardware=hotplate3, clamp_number=3)

    hotplate_fan_1 = HotplateFan.HotplateFan(hotplate1, arduino_controller=arduino,
fan_number=1)

    hotplate_fan_2 = HotplateFan.HotplateFan(hotplate2, arduino_controller=arduino,
fan_number=2)

    hotplate_fan_3 = HotplateFan.HotplateFan(hotplate3, arduino_controller=arduino,
fan_number=3)

    capper = CapperDecapper.CapperDecapper(arduino_controller=arduino)

    valve1 = SwitchingValve.SwitchingValveVici(com_port='COM3')

    valve2 = SwitchingValve.SwitchingValveVici(com_port='COM14')

    valve3 = SwitchingValve.SwitchingValveVici(com_port='COM17')

    pump1 = WPI.Aladdin(com_port='COM12')

    pump2 = WPI.Aladdin(com_port='COM9', baud_rate=9600,
pump_type=SyringePumpType.AL_1010)

    pump3 = WPI.Aladdin(com_port='COM5', baud_rate=9600,
pump_type=SyringePumpType.AL_1010)

    probesonicator = Hielscher.UP200ST(ip_address='192.168.233.233')

    centrifuge = Herolab.RobotCen(com_port='COM8', initialize_rotor=True,
home_rotor=True)

    zetasizer = MalvernPanalytical.ZetaSizer(local_controller=local_server)



    gripchange_holder =
SampleHolder.SampleHolder(SampleHolder.SampleHolderDefinitions.Corkring_Small,
deck_position=4)

    hotplate1_holder =
SampleHolder.SampleHolder(SampleHolderDefinitions.Ika_100mL_Heating_Block,
parent_hardware=hotplate1, deck_position=1)

    hotplate2_holder =
SampleHolder.SampleHolder(SampleHolderDefinitions.Ika_100mL_Heating_Block,
parent_hardware=hotplate2, deck_position=2)

    hotplate3_holder =
SampleHolder.SampleHolder(SampleHolderDefinitions.Ika_100mL_Heating_Block,
parent_hardware=hotplate3, deck_position=3)

    ot2_holder_15ml =
SampleHolder.SampleHolder(SampleHolder.SampleHolderDefinitions.Opentrons_15mL_Tube_
Rack, parent_hardware=ot2, deck_position=2)

    ot2_holder_50ml =
SampleHolder.SampleHolder(SampleHolder.SampleHolderDefinitions.Opentrons_50mL_Tube_
Rack, parent_hardware=ot2, deck_position=1)

    ot2_holder_flask_100ml =
SampleHolder.SampleHolder(SampleHolder.SampleHolderDefinitions.Opentrons_100mL_Flas
k_Rack, parent_hardware=ot2, deck_position=3)
```

```
    falcon_tube_holder_50ml =
SampleHolder.SampleHolder(SampleHolder.SampleHolderDefinitions.Isolab_50mL_Foldable
_Tube_Rack, deck_position=1, leave_even_rows_empty=True)

    falcon_tube_holder_15ml =
SampleHolder.SampleHolder(SampleHolder.SampleHolderDefinitions.Isolab_15mL_Foldable
_Tube_Rack, deck_position=2, leave_even_rows_empty=True)

    flaskstation =
SampleHolder.SampleHolder(SampleHolder.SampleHolderDefinitions.Flask_Station,
deck_position=3, leave_even_rows_empty=False)


    flask_1_100ml = Container(flaskstation, slot_number=1,
container_type=ContainerTypeCollection.FLASK_100_ML, current_volume=Volume(0,
'mL'), has_stirbar=True, name='flask1')

    flask_2_100ml = Container(flaskstation, slot_number=2,
container_type=ContainerTypeCollection.FLASK_100_ML, current_volume=Volume(0,
'mL'), has_stirbar=True, name='flask2')

    flask_3_100ml = Container(flaskstation, slot_number=3,
container_type=ContainerTypeCollection.FLASK_100_ML, current_volume=Volume(0,
'mL'), has_stirbar=True, name='flask3')

    flask_4_100ml = Container(flaskstation, slot_number=4,
container_type=ContainerTypeCollection.FLASK_100_ML, current_volume=Volume(0,
'mL'), has_stirbar=True, name='flask4')

    falcontube1_15ml = Container(falcon_tube_holder_15ml, slot_number=1,
container_type=ContainerTypeCollection.FALCON_TUBE_15_ML, current_volume='0 mL',
is_capped=True)

    falcontube2_15ml = Container(falcon_tube_holder_15ml, slot_number=2,
container_type=ContainerTypeCollection.FALCON_TUBE_15_ML, current_volume='0 mL',
is_capped=True)

    falcontube1_50ml = Container(falcon_tube_holder_50ml, slot_number=1,
container_type=ContainerTypeCollection.FALCON_TUBE_50_ML, current_volume='0 mL',
is_capped=True)

    falcontube2_50ml = Container(falcon_tube_holder_50ml, slot_number=2,
container_type=ContainerTypeCollection.FALCON_TUBE_50_ML, current_volume='0 mL',
is_capped=True)

    falcontube3_50ml = Container(falcon_tube_holder_50ml, slot_number=3,
container_type=ContainerTypeCollection.FALCON_TUBE_50_ML, current_volume='0 mL',
is_capped=True)

    falcontube4_50ml = Container(falcon_tube_holder_50ml, slot_number=4,
container_type=ContainerTypeCollection.FALCON_TUBE_50_ML, current_volume='0 mL',
is_capped=True)

    falcontube5_50ml = Container(falcon_tube_holder_50ml, slot_number=5,
container_type=ContainerTypeCollection.FALCON_TUBE_50_ML, current_volume='0 mL',
is_capped=True)

    falcontube6_50ml = Container(falcon_tube_holder_50ml, slot_number=6,
container_type=ContainerTypeCollection.FALCON_TUBE_50_ML, current_volume='0 mL',
is_capped=True)


    falcontube_for_cleaning = Container(falcon_tube_holder_50ml, slot_number=14,
container_type=ContainerTypeCollection.FALCON_TUBE_50_ML, current_volume='40 mL',
is_capped=False)


    WasteContainerValve1 = Container(current_hardware=valve1, slot_number=0,
name='Waste_Container', current_volume='2 L', max_volume='5 L')

    WasteContainerValve2 = Container(current_hardware=valve2, slot_number=6,
name='Waste_ContainerValve2', current_volume='0 mL', max_volume='50 mL')
```

```
    DLSCell = Container(current_hardware=valve1, slot_number=4, name='DLS_Cell', current_volume='0 mL')

    MilliQ_ContainerValve1 = Container(current_hardware=valve1, slot_number=1, name='MilliQ_ContainerValve1', current_volume='1.2 L')

    MilliQ_ContainerValve2 = Container(current_hardware=valve2, slot_number=9, name='CleaningChemicalValve2', current_volume='500 mL')

    Ethanol_wash_container = Container(current_hardware=valve1, slot_number=5, name='Ethanol_wash', current_volume='5 L')


    NaOHContainer = Container(current_hardware=valve2, slot_number=4, name='NaOH_Container', current_volume='50 mL')

    CuOAcContainer = Container(current_hardware=ot2, deck_position=8, slot_number=7, name='CuOAc_Container', current_volume='15 mL', container_type=ContainerTypeCollection.FALCON_TUBE_50_ML)

    AcOHContainer = Container(current_hardware=ot2, deck_position=8, slot_number=1, name='AcOH_Container', current_volume='2.5 mL', container_type=ContainerTypeCollection.FALCON_TUBE_15_ML)


    Water_wash = Chemical(container=MilliQ_ContainerValve1, name='Water', volume='10 mL', lookup_missing_values=False)

    water_dls = Chemical(container=MilliQ_ContainerValve1, name='Water', volume='5 mL', lookup_missing_values=False)


    sonication_time = '20 s'

    washing_steps = 3

    centrifugation_speed = '14000 rcf'

    centrifugation_time = '10 min'

    sonication_time = '20 s'

    sample_name_1 = 'MZ039'

    sample_name_2 = 'MZ040'


    ot2_configuration = {
        1: ot2_holder_50ml,
        2: ot2_holder_15ml,
        3: ot2_holder_flask_100ml,
        4: None,
        5: None,
        6: None,
        7: None,
        8: 'opentrons_10_tuberack_falcon_4x50ml_6x15ml_conical',
        9: None,
        10: 'opentrons_96_tiprack_300ul',
        11: 'labsolute_96_tiprack_1000ul',
        12: 'p1000_single_gen2',  # left pipette
        13: 'p300_single_gen2',  # right pipette
    }


    valve1_configuration = {
        0: None,
        1: MilliQ_ContainerValve1,
        2: Ethanol_wash_container,
        3: WasteContainerValve1,
        4: DLSCell,
```

```
        5: None,
        6: EtOH_NH3_water_container,
        7: None,
        8: 'Outlet',
        9: None,
        10: pump1
    }

    valve2_configuration = {
        0: None,
        1: hotplate1,
        2: hotplate2,
        3: hotplate3,
        4: MilliQ_ContainerValve2,
        5: None,
        6: WasteContainerValve2,
        7: None,
        8: None,
        9: TEOSContainer,
        10: pump2
    }

    valve3_configuration = {
        0: None,
        1: hotplate1,
        2: hotplate2,
        3: hotplate3,
        4: MilliQ_ContainerValve3,
        5: None,
        6: None,
        7: None,
        8: None,
        9: WasteContainerValve3,
        10: pump3
    }

    valve2_dead_volumes = {
        0: None,
        1: '1.390 mL',
        2: '1.35 mL',
        3: '1.87 mL',
        4: None,
        5: None,
        6: None,
        7: None,
        8: None,
        9: None,
        10: None
    }

    ot2.set_hardware_configuration(ot2_configuration)

    pump1.set_syringe(Syringes.GLASS_SYRINGE_SOCOREX_50ML,
default_addition_rate='60 mL/min')

    valve1.set_configuration(valve1_configuration)

    pump2.set_syringe(Syringes.GLASS_SYRINGE_SOCOREX_5ML, default_addition_rate='10
mL/min')     #(Syringes.GLASS_SYRINGE_SOCOREX_10ML, default_addition_rate='20
mL/min')

    valve2.set_configuration(valve2_configuration)

    valve2.set_dead_volumes(valve2_dead_volumes)

    pump3.set_syringe(Syringes.GLASS_SYRINGE_SOCOREX_5ML, default_addition_rate='20
mL/min')

    valve3.set_configuration(valve3_configuration)
```

```
    CuO_Synthesis_reaction_parameters = ((flask_1_100ml, falcontube1_50ml,
falcontube2_50ml, '1.5 mL'),
                                         (flask_2_100ml, falcontube3_50ml,
falcontube4_50ml, '0.7 mL'))


    threads = []
    for i, p in enumerate(CuO_Synthesis_reaction_parameters):
        threads.append(threading.Thread(name=f'Thread_{i}', target=CuO_Synthesis,
args=tuple(p)))
        threads[-1].start()
        time.sleep(450)


    for t in threads:
        t.join()


    # Washing
    for _ in range(0, washing_steps):
        falcontube1_50ml.centrifuge([falcontube2_50ml, falcontube3_50ml,
falcontube4_50ml, falcontube5_50ml, falcontube6_50ml],
centrifugation_speed=centrifugation_speed, centrifugation_time=centrifugation_time)


falcontube1_50ml.remove_supernatant_and_redisperse(waste_container=WasteContainerVa
lve1, sonication_time=sonication_time, redispersion_chemical=Water_wash,
sonication_power=50, sonication_amplitude=50, sonicator=probesonicator,
bottom_clearance_sonication=15, purging_volume='10 mL',
bottom_clearance_withdrawing=8, container_for_cleaning=falcontube_for_cleaning)


falcontube2_50ml.remove_supernatant_and_redisperse(waste_container=WasteContainerVa
lve1, sonication_time=sonication_time, redispersion_chemical=Water_wash,
sonication_power=50, sonication_amplitude=50, sonicator=probesonicator,
bottom_clearance_sonication=15, purging_volume='10 mL',
bottom_clearance_withdrawing=8, container_for_cleaning=falcontube_for_cleaning)


falcontube3_50ml.remove_supernatant_and_redisperse(waste_container=WasteContainerVa
lve1, sonication_time=sonication_time, redispersion_chemical=Water_wash,
sonication_power=50, sonication_amplitude=50, sonicator=probesonicator,
bottom_clearance_sonication=15, purging_volume='10 mL',
bottom_clearance_withdrawing=8, container_for_cleaning=falcontube_for_cleaning)


falcontube4_50ml.remove_supernatant_and_redisperse(waste_container=WasteContainerVa
lve1, sonication_time=sonication_time, redispersion_chemical=Water_wash,
sonication_power=50, sonication_amplitude=50, sonicator=probesonicator,
bottom_clearance_sonication=15, purging_volume='10 mL',
bottom_clearance_withdrawing=8, container_for_cleaning=falcontube_for_cleaning)


    centrifuge.close_lid()


    # Dilution for DLS
    falcontube1_50ml.transfer_content_to_container(falcontube1_15ml, volume='1 mL',
transfer_hardware=valve1, bottom_clearance_withdrawing=10)
    falcontube1_15ml.add_chemical(chemical=Chemical.from_stock_chemical(Water_wash,
volume='9 mL'))
```

```
    falcontube3_50ml.transfer_content_to_container(falcontube2_15ml, volume='1 mL',
transfer_hardware=valve1, bottom_clearance_withdrawing=10)

    falcontube2_15ml.add_chemical(chemical=Chemical.from_stock_chemical(Water_wash,
volume='9 mL'))


    # DLS measurement

    falcontube1_15ml.measure_dls(dls_cell=DLSCell, sop_path=os.path.join('C:\\',
'users', 'WS8717-apollo', 'Desktop', 'Apollo', 'Characterization', 'DLS', 'SOP',
'DLS_ZETA_CuO_in_Water.zskd'), sample_name=f"{sample_name_1}" ,
chemical_for_cleaning=Chemical.from_stock_chemical(Water_wash, volume='10 mL'),
dls_device=zetasizer, bottom_clearance_sampling=20,
waste_container=WasteContainerValve1, dls_volume='3 mL', dead_volume_dls='5.6 mL')

    falcontube2_15ml.measure_dls(dls_cell=DLSCell, sop_path=os.path.join('C:\\',
'users', 'WS8717-apollo', 'Desktop', 'Apollo', 'Characterization', 'DLS', 'SOP',
'DLS_ZETA_CuO_in_Water.zskd'), sample_name=f"{sample_name_2}" ,
chemical_for_cleaning=Chemical.from_stock_chemical(Water_wash, volume='10 mL'),
dls_device=zetasizer, bottom_clearance_sampling=20,
waste_container=WasteContainerValve1, dls_volume='3 mL', dead_volume_dls='5.6 mL')


    # ELN upload

    eln.write_synthesis_step(experiment_name=f"{sample_name1},{sample_name2}")
```

4.6. Au@SiO_2 NPs

```
import time
import datetime
import os
import threading

import API.MinervaAPI
import Minerva.Software.OpenBISELNIntegration.OpenBisElnIntegration
from Minerva import *
from Minerva.API.HelperClassDefinitions import PathNames


def Au_core_SiO2_Synthesis(sonication_container:Container, container: Container,
falcontube1: Container, priming_volume):

    CTAB = Chemical(container=CTABContainer, name='CTAB', mass_concentration='6
mg/mL', mass='5.6 mg', lookup_missing_values=True)

    Water = Chemical(container=MilliQ_ContainerOT2, name='Water', volume='2.17 mL',
lookup_missing_values=True)

    Ethanolic_TEOS = Chemical(container=TEOSContainer, name='TEOS',
concentration='0.9 mol/L', volume='100 uL', lookup_missing_values=True)

    Arginine = Chemical(container=L_ArginineContainer, name='Arginine',
mass_concentration='10 mg/mL', mass='2 mg', lookup_missing_values=True)

    Au_Cores = Chemical(container=Au_Core_Container, name='Au_Cores', volume='0.5
mL', lookup_missing_values=False)


    stirring_speed = 590  # rpm
    reaction_temperature = 100  # °C
    heating_time = '3 h'
    temperature_error = 2  # °C
    cooling_temperature = 40  # °C
    teos_infusion_rate = '0.8 mL/min'
```

```
    sonication_container.add_chemical([Water, CTAB, Arginine, Au_Cores])


    dropoff = sonication_container.slot_number

    reaction_vol_transfer = sonication_container.current_volume

    sonication_container._current_volume =
Volume((sonication_container.current_volume), 'mL')


    sonication_container.move(target_hardware=probesonicator, bottom_clearance=18)

    probesonicator.start_sonication(sonication_time=20, sonication_amplitude=10,
sonication_power=10)

    sonication_container.move(target_hardware=ot2_holder_15ml,
target_slot_number=dropoff)

    falcontube_for_cleaning.sonicate(sonicator=probesonicator, sonication_time=5,
sonication_power=50, sonication_amplitude=50, bottom_clearance=20)


    sonicated_reaction_mix = Chemical(container=sonication_container,
name='sonicated_reaction_mix', volume=reaction_vol_transfer,
lookup_missing_values=False)


    container.add_chemical(sonicated_reaction_mix)


    container.infuse_while_heating(chemical=[TEOS],
                                   heating_temperature=reaction_temperature,
                                   stirring_speed=stirring_speed,
                                   heating_time=heating_time,
                                   addition_hardware=valve2,
                                   withdraw_rate='5 mL/min',
                                   addition_rate=teos_infusion_rate,
                                   purging_addition_rate='10 mL/min',
                                   priming_volume=priming_volume,
                                   purging_volume='2.9 mL',
                                   purging_port=5,
                                   priming_waste_container=WasteContainerValve2,
                                   cooldown_temperature=cooling_temperature,
                                   maximum_temperature_deviation=temperature_error,
                                   temperature_stabilization_time='30 min',
                                   chemical_for_cleaning=Water_wash_valve2,
                                   active_cooling=True)


    dropoff1 = falcontube1.slot_number

    container.transfer_content_to_container(target_containers=[falcontube1],
transfer_hardware=valve1, bottom_clearance_withdrawing=8,
dropoff_locations=[(flaskstation, 0), (falcon_tube_holder_15ml, dropoff1)],
purging_volume='15 mL')
```

```
def NH4NO3_extraction(sonication_container:Container, container: Container,
washed_falcontube: Container, falcontube1: Container):

    stirring_speed = 300  # rpm

    reaction_temperature = 72  # °C

    heating_time = '1.5 h'

    temperature_error = 2  # °C

    cooling_temperature = 40  # °C


    ot2_dropoff = sonication_container.slot_number


    sonication_container.move(target_hardware=falcon_tube_holder_15ml)


    extraction_vol = washed_falcontube.current_volume


    washed_falcontube.move(target_hardware=ot2_holder_15ml,
target_slot_number=ot2_dropoff)


    extraction_mix = Chemical(container=washed_falcontube, name='extraction_mix',
volume=extraction_vol, lookup_missing_values=False)


    container.add_chemical(extraction_mix)


    container.heat(heating_temperature=reaction_temperature,
stirring_speed=stirring_speed, heating_time=heating_time,
cooldown_temperature=cooling_temperature, active_cooling=True,
temperature_stabilization_time='10 min')


    dropoff1 = falcontube1.slot_number

    container.transfer_content_to_container(target_containers=[falcontube1],
transfer_hardware=valve1, bottom_clearance_withdrawing=8,
dropoff_locations=[(flaskstation, 0), (falcon_tube_holder_15ml, dropoff1)],
purging_volume='15 mL')




if __name__ == '__main__':

    esb = EmergencyStopButton.EmergencyStopButton(com_port='COM34')

    eln =
Minerva.Software.OpenBISELNIntegration.OpenBisElnIntegration.ElectronicLabNotebook(
space_name='1.0_MINERVA', project_code='CORE_SHELL')

    local_server = LocalPCServer.LocalPCServer(com_port='COM7')

    arduino = ArduinoController.ArduinoController(com_port='COM25')

    dht22 = DHT22Sensor.DHT22Sensor(arduino_controller=arduino)

    robotarm = UFactory.XArm6(ip_address='192.168.1.204',
levelling_data_file=os.path.join('..', 'SampleHolder',
'Table_Levelling_Data.json'))
```

```
    ot2 = OpentronsOT2.OT2(ip_address='OT2CEP20210918R04.local')

    hotplate1 = IkaHotplate.RCTDigital5(com_port='COM6')

    hotplate2 = IkaHotplate.RCTDigital5(com_port='COM4')

    hotplate3 = IkaHotplate.RCTDigital5(com_port='COM18')

    hotplate1_clamp =
HotplateClamp.HotplateClampDCMotor(arduino_controller=arduino,
parent_hardware=hotplate1, clamp_number=1)

    hotplate2_clamp =
HotplateClamp.HotplateClampDCMotor(arduino_controller=arduino,
parent_hardware=hotplate2, clamp_number=2)

    hotplate3_clamp =
HotplateClamp.HotplateClampDCMotor(arduino_controller=arduino,
parent_hardware=hotplate3, clamp_number=3)

    hotplate_fan_1 = HotplateFan.HotplateFan(hotplate1, arduino_controller=arduino,
fan_number=1)

    hotplate_fan_2 = HotplateFan.HotplateFan(hotplate2, arduino_controller=arduino,
fan_number=2)

    hotplate_fan_3 = HotplateFan.HotplateFan(hotplate3, arduino_controller=arduino,
fan_number=3)

    capper = CapperDecapper.CapperDecapper(arduino_controller=arduino)

    valve1 = SwitchingValve.SwitchingValveVici(com_port='COM3')

    valve2 = SwitchingValve.SwitchingValveVici(com_port='COM14')

    valve3 = SwitchingValve.SwitchingValveVici(com_port='COM17')

    pump1 = WPI.Aladdin(com_port='COM12')

    pump2 = WPI.Aladdin(com_port='COM9', baud_rate=9600,
pump_type=SyringePumpType.AL_1010)

    pump3 = WPI.Aladdin(com_port='COM5', baud_rate=9600,
pump_type=SyringePumpType.AL_1010)

    probesonicator = Hielscher.UP200ST(ip_address='192.168.233.233')

    centrifuge = Herolab.RobotCen(com_port='COM8', initialize_rotor=True,
home_rotor=True)

    zetasizer = MalvernPanalytical.ZetaSizer(local_controller=local_server)



    gripchange_holder =
SampleHolder.SampleHolder(SampleHolder.SampleHolderDefinitions.Corkring_Small,
deck_position=4)

    hotplate1_holder =
SampleHolder.SampleHolder(SampleHolderDefinitions.Ika_10mL_Heating_Block,
parent_hardware=hotplate1, deck_position=1)

    hotplate2_holder =
SampleHolder.SampleHolder(SampleHolderDefinitions.Ika_10mL_Heating_Block,
parent_hardware=hotplate2, deck_position=2)

    hotplate3_holder =
SampleHolder.SampleHolder(SampleHolderDefinitions.Ika_10mL_Heating_Block,
parent_hardware=hotplate3, deck_position=3)

    ot2_holder_15ml =
SampleHolder.SampleHolder(SampleHolder.SampleHolderDefinitions.Opentrons_15mL_Tube_
Rack, parent_hardware=ot2, deck_position=1)

    ot2_holder_50ml =
SampleHolder.SampleHolder(SampleHolder.SampleHolderDefinitions.Opentrons_50mL_Tube_
Rack, parent_hardware=ot2, deck_position=4)
```

```
    ot2_holder_flask_10ml =
SampleHolder.SampleHolder(SampleHolder.SampleHolderDefinitions.Opentrons_10mL_Flask
_Rack, parent_hardware=ot2, deck_position=2)

    ot2_holder_flask_25ml =
SampleHolder.SampleHolder(SampleHolder.SampleHolderDefinitions.Opentrons_25mL_Flask
_Rack, parent_hardware=ot2, deck_position=3)

    ot2_holder_flask_100ml =
SampleHolder.SampleHolder(SampleHolder.SampleHolderDefinitions.Opentrons_100mL_Flas
k_Rack, parent_hardware=ot2, deck_position=4)

    falcon_tube_holder_50ml =
SampleHolder.SampleHolder(SampleHolder.SampleHolderDefinitions.Isolab_50mL_Foldable
_Tube_Rack, deck_position=1, leave_even_rows_empty=True)

    falcon_tube_holder_15ml =
SampleHolder.SampleHolder(SampleHolder.SampleHolderDefinitions.Isolab_15mL_Foldable
_Tube_Rack, deck_position=2, leave_even_rows_empty=True)

    flaskstation =
SampleHolder.SampleHolder(SampleHolder.SampleHolderDefinitions.Flask_Station,
deck_position=3, leave_even_rows_empty=False)



    flaskflush = Container(flaskstation, slot_number=5,
container_type=ContainerTypeCollection.FLASK_100_ML, current_volume=Volume(0,
'mL'), has_stirbar=True, name='flask_flush')

    flask_1_10ml = Container(flaskstation, slot_number=1,
container_type=ContainerTypeCollection.FLASK_10_ML, current_volume=Volume(0, 'mL'),
has_stirbar=True, name='flask1')

    flask_2_10ml = Container(flaskstation, slot_number=2,
container_type=ContainerTypeCollection.FLASK_10_ML, current_volume=Volume(0, 'mL'),
has_stirbar=True, name='flask2')

    flask_3_10ml = Container(flaskstation, slot_number=3,
container_type=ContainerTypeCollection.FLASK_10_ML, current_volume=Volume(0, 'mL'),
has_stirbar=True, name='flask3')

    falcontube1_50ml = Container(falcon_tube_holder_50ml, slot_number=1,
container_type=ContainerTypeCollection.FALCON_TUBE_50_ML, current_volume='0 mL',
is_capped=True)

    falcontube2_50ml = Container(falcon_tube_holder_50ml, slot_number=2,
container_type=ContainerTypeCollection.FALCON_TUBE_50_ML, current_volume='0 mL',
is_capped=True)

    falcontube_for_cleaning = Container(falcon_tube_holder_50ml, slot_number=3,
container_type=ContainerTypeCollection.FALCON_TUBE_50_ML, current_volume='40 mL',
is_capped=False)



    falcontube1_sonication_ot2 = Container(ot2_holder_15ml, slot_number=5,
container_type=ContainerTypeCollection.FALCON_TUBE_15_ML, current_volume='0 mL',
is_capped=False)

    falcontube2_sonication_ot2 = Container(ot2_holder_15ml, slot_number=6,
container_type=ContainerTypeCollection.FALCON_TUBE_15_ML, current_volume='0 mL',
is_capped=False)

    falcontube3_sonication_ot2 = Container(ot2_holder_15ml, slot_number=15,
container_type=ContainerTypeCollection.FALCON_TUBE_15_ML, current_volume='0 mL',
is_capped=False)



    falcontube1_15ml = Container(falcon_tube_holder_15ml, slot_number=1,
container_type=ContainerTypeCollection.FALCON_TUBE_15_ML, current_volume='0 mL',
is_capped=True)
```

```
    falcontube2_15ml = Container(falcon_tube_holder_15ml, slot_number=2,
container_type=ContainerTypeCollection.FALCON_TUBE_15_ML, current_volume='0 mL',
is_capped=True)

    falcontube3_15ml = Container(falcon_tube_holder_15ml, slot_number=3,
container_type=ContainerTypeCollection.FALCON_TUBE_15_ML, current_volume='0 mL',
is_capped=True)

    falcontube4_15ml = Container(falcon_tube_holder_15ml, slot_number=4,
container_type=ContainerTypeCollection.FALCON_TUBE_15_ML, current_volume='0 mL',
is_capped=True)

    falcontube15ml_counter_core = Container(falcon_tube_holder_15ml, slot_number=5,
container_type=ContainerTypeCollection.FALCON_TUBE_15_ML, current_volume='10 mL',
is_capped=True)

    falcontube15ml_counter_reaction = Container(falcon_tube_holder_15ml,
slot_number=6, container_type=ContainerTypeCollection.FALCON_TUBE_15_ML,
current_volume='3.9 mL', is_capped=True)

    falcontube15ml_counter_extraction = Container(falcon_tube_holder_15ml,
slot_number=7, container_type=ContainerTypeCollection.FALCON_TUBE_15_ML,
current_volume='3 mL', is_capped=True)


    WasteContainerValve1 = Container(current_hardware=valve1, slot_number=3,
name='Waste_Container', current_volume='2 L', max_volume='5 L')

    WasteContainerValve2 = Container(current_hardware=valve2, slot_number=6,
name='Waste_ContainerValve2', current_volume='0 mL', max_volume='50 mL')

    WasteContainerValve3 = Container(current_hardware=valve3, slot_number=9,
name='Waste_ContainerValve3', current_volume='0 mL', max_volume='20 mL')

    DLSCell = Container(current_hardware=valve1, slot_number=4, name='DLS_Cell',
current_volume='0 mL')

    MilliQ_ContainerValve1 = Container(current_hardware=valve1, slot_number=1,
name='MilliQ_ContainerValve1', current_volume='1.2 L')

    MilliQ_ContainerValve2 = Container(current_hardware=valve2, slot_number=4,
name='CleaningChemicalValve2', current_volume='500 mL')

    MilliQ_ContainerValve3 = Container(current_hardware=valve3, slot_number=4,
name='CleaningChemicalValve3', current_volume='15 mL')

    Ethanol_wash_container = Container(current_hardware=valve1, slot_number=2,
name='Ethanol_wash', current_volume='5 L')


    falcontube_waste_ot2 = Container(current_hardware=ot2, deck_position=8,
slot_number=9, name='ot2_waste_container', current_volume='0 mL',
container_type=ContainerTypeCollection.FALCON_TUBE_50_ML)

    MilliQ_ContainerOT2 = Container(current_hardware=ot2, deck_position=8,
slot_number=7, name='MilliQ_ContainerOT2', current_volume='10 mL',
container_type=ContainerTypeCollection.FALCON_TUBE_50_ML)

    CTABContainer = Container(current_hardware=ot2, deck_position=8, slot_number=8,
name='CTAB_Container', current_volume='3 mL',
container_type=ContainerTypeCollection.FALCON_TUBE_50_ML)

    NH4NO3Container = Container(current_hardware=valve1, slot_number=6,
name='NH4NO3_Container', current_volume='10 mL')

    L_ArginineContainer = Container(current_hardware=ot2, deck_position=8,
slot_number=2, name='L_Arg_Container', current_volume='1 mL',
container_type=ContainerTypeCollection.FALCON_TUBE_15_ML)

    Au_Core_Container = Container(current_hardware=ot2_holder_15ml, slot_number=14,
name='Au_Cores_Container', current_volume='10 mL',
container_type=ContainerTypeCollection.FALCON_TUBE_15_ML, is_capped=True)
```

```
    TEOSContainer = Container(current_hardware=valve2, slot_number=9,
name='TEOS_Container', current_volume='2.6 mL')


    Water_wash_valve2 = Chemical(container=MilliQ_ContainerValve2, name='Water',
volume='0.8 mL', lookup_missing_values=False)

    NH4NO3 = Chemical(container=NH4NO3Container, volume='3 mL',
mass_concentration='20 mg/mL', lookup_missing_values=True)

    Ethanol_wash = Chemical(container=Ethanol_wash_container, name='Ethanol',
volume='5 mL', lookup_missing_values=True)

    Water_wash = Chemical(container=MilliQ_ContainerValve1, name='Water', volume='5
mL', lookup_missing_values=True)


    sonication_time = '30 s'

    washing_steps = 3

    centrifugation_speed = '14000 rcf'

    centrifugation_time = '15 min'

    sample_name = 'MZ208'


    ot2_configuration = {
        1: ot2_holder_15ml,
        2: ot2_holder_flask_10ml,
        3: ot2_holder_flask_25ml,
        4: ot2_holder_flask_100ml,
        5: None,
        6: None,
        7: None,
        8: 'opentrons_10_tuberack_falcon_4x50ml_6x15ml_conical',
        9: None,
        10:'opentrons_96_tiprack_20ul',    # 'opentrons_96_tiprack_300ul'
        11:'labsolute_96_tiprack_1000ul',
        12:'p1000_single_gen2',  # left pipette
        13:'p20_single_gen2',  # right pipette  'p300_single_gen2'
    }


    valve1_configuration = {
        0: None,
        1: MilliQ_ContainerValve1,
        2: Ethanol_wash_container,
        3: WasteContainerValve1,
        4: DLSCell,
        5: None,
        6: NH4NO3Container,
        7: None,
        8: 'Outlet',
        9: None,
        10: pump1
    }


    valve2_configuration = {
        0: None,
        1: hotplate1,
        2: hotplate2,
        3: hotplate3,
        4: MilliQ_ContainerValve2,
        5: None,
        6: WasteContainerValve2,
        7: None,
        8: None,
        9: TEOSContainer,
        10: pump2
```

```
    }

    valve3_configuration = {
        0: None,
        1: hotplate1,
        2: hotplate2,
        3: hotplate3,
        4: MilliQ_ContainerValve3,
        5: None,
        6: None,
        7: None,
        8: None,
        9: WasteContainerValve3,
        10: pump3
    }

    valve2_dead_volumes = {
        0: None,
        1: '1.390 mL',
        2: '1.35 mL',
        3: '1.87 mL',
        4: None,
        5: None,
        6: None,
        7: None,
        8: None,
        9: None,
        10: None
    }

    ot2.set_hardware_configuration(ot2_configuration)

    pump1.set_syringe(Syringes.GLASS_SYRINGE_SOCOREX_50ML,
default_addition_rate='70 mL/min')

    valve1.set_configuration(valve1_configuration)

    pump2.set_syringe(Syringes.GLASS_SYRINGE_SOCOREX_1ML, default_addition_rate='3
mL/min')

    valve2.set_configuration(valve2_configuration)

    valve2.set_dead_volumes(valve2_dead_volumes)

    pump3.set_syringe(Syringes.GLASS_SYRINGE_SOCOREX_5ML, default_addition_rate='20
mL/min')

    valve3.set_configuration(valve3_configuration)


    # washing and concentrating the cores

    Au_Core_Container.centrifuge(falcontube15ml_counter_core,
centrifugation_time='90 min', centrifugation_speed='3500 rcf',
centrifugation_temperature=5)


Au_Core_Container.remove_supernatant_and_redisperse(waste_container=WasteContainerV
alve1, redispersion_chemical=Chemical.from_stock_chemical(Water_wash, volume='1
mL'), bottom_clearance_withdrawing=7, sonicator=probesonicator, sonication_time='5
s', sonication_power=50, sonication_amplitude=50, bottom_clearance_sonication=10)


    Au_core_SiO2_Synthesis_parameters = ((falcontube1_sonication_ot2, flask_1_10ml,
falcontube1_15ml, '1200 uL'))


    threads = []

    for i, p in enumerate(Au_core_SiO2_Synthesis_parameters):
```

```
        threads.append(threading.Thread(name=f'Thread_{i}', 
target=Au_core_SiO2_Synthesis, args=tuple(p)))

        threads[-1].start()


    for t in threads:

        t.join()


    # Washing after synthesis

    falcontube1_15ml.centrifuge([falcontube15ml_counter_reaction], 
centrifugation_speed=centrifugation_speed, centrifugation_time=centrifugation_time)


falcontube1_15ml.remove_supernatant_and_redisperse(waste_container=WasteContainerVa
lve1, sonication_time=sonication_time, redispersion_chemical=Water_wash, 
sonication_power=20, sonication_amplitude=20, sonicator=probesonicator, 
bottom_clearance_sonication=18, bottom_clearance_withdrawing=12, 
container_for_cleaning=falcontube_for_cleaning)


falcontube15ml_counter_reaction.remove_supernatant_and_redisperse(waste_container=W
asteContainerValve1, sonication_time=sonication_time, 
redispersion_chemical=Water_wash, sonication_power=20, sonication_amplitude=20, 
sonicator=probesonicator, bottom_clearance_sonication=18, 
bottom_clearance_withdrawing=12, container_for_cleaning=falcontube_for_cleaning)

    for _ in range(0, washing_steps-1):

        falcontube1_15ml.centrifuge([falcontube15ml_counter_reaction], 
centrifugation_speed=centrifugation_speed, centrifugation_time=centrifugation_time)


falcontube1_15ml.remove_supernatant_and_redisperse(waste_container=WasteContainerVa
lve1, sonication_time=sonication_time, redispersion_chemical=Ethanol_wash, 
sonication_power=20, sonication_amplitude=20, sonicator=probesonicator, 
bottom_clearance_sonication=18, bottom_clearance_withdrawing=12, 
container_for_cleaning=falcontube_for_cleaning)


falcontube15ml_counter_reaction.remove_supernatant_and_redisperse(waste_container=W
asteContainerValve1, sonication_time=sonication_time, 
redispersion_chemical=Ethanol_wash, sonication_power=20, sonication_amplitude=20, 
sonicator=probesonicator, bottom_clearance_sonication=18, 
bottom_clearance_withdrawing=12, container_for_cleaning=falcontube_for_cleaning)

    centrifuge.close_lid()


    # Redispersion in ammonium nitrate

    falcontube1_15ml.centrifuge([falcontube15ml_counter_reaction], 
centrifugation_speed=centrifugation_speed, centrifugation_time=centrifugation_time)


falcontube1_15ml.remove_supernatant_and_redisperse(waste_container=WasteContainerVa
lve1, sonication_time=sonication_time, redispersion_chemical=NH4NO3, 
sonication_power=20, sonication_amplitude=20, sonicator=probesonicator, 
bottom_clearance_sonication=18, bottom_clearance_withdrawing=12, 
container_for_cleaning=falcontube_for_cleaning)


    # extraction protocol

    NH4NO3_Extraction_parameters = ((falcontube1_sonication_ot2, flask_1_10ml, 
falcontube1_15ml, falcontube3_15ml))


    threads = []
```

```
    for i, p in enumerate(NH4NO3_Extraction_parameters):

        threads.append(threading.Thread(name=f'Thread_{i}',
target=NH4NO3_extraction, args=tuple(p)))

        threads[-1].start()


    for t in threads:

        t.join()


    # washing after extraction

    for _ in range(0, washing_steps):

        falcontube3_15ml.centrifuge([falcontube15ml_counter_extraction],
centrifugation_speed=centrifugation_speed, centrifugation_time=centrifugation_time)


falcontube3_15ml.remove_supernatant_and_redisperse(waste_container=WasteContainerVa
lve1, sonication_time=sonication_time, redispersion_chemical=Ethanol_wash,
sonication_power=20, sonication_amplitude=20, sonicator=probesonicator,
bottom_clearance_sonication=15, bottom_clearance_withdrawing=12,
container_for_cleaning=falcontube_for_cleaning)


falcontube15ml_counter_extraction.remove_supernatant_and_redisperse(waste_container
=WasteContainerValve1, sonication_time=sonication_time,
redispersion_chemical=Ethanol_wash, sonication_power=20, sonication_amplitude=20,
sonicator=probesonicator, bottom_clearance_sonication=15,
bottom_clearance_withdrawing=12, container_for_cleaning=falcontube_for_cleaning)

    centrifuge.close_lid()


    #ELN upload

    eln.write_synthesis_step(experiment_name=f"{sample_name1} ")
```

4.7. CuO@SiO_2 NPs

```
import time
import datetime
import os
import threading

import API.MinervaAPI
import Minerva.Software.OpenBISELNIntegration.OpenBisElnIntegration
from Minerva import *
from Minerva.API.HelperClassDefinitions import PathNames


def CuO_CoreShell_Synthesis(container: Container, falcontube1: Container,
falcontube2: Container):

    CTAB = Chemical(container=CTABContainer, name='CTAB', mass_concentration='4.2
mg/mL', mass='37.5 mg', lookup_missing_values=True)

    CuO_cores = Chemical(container=CuOContainer, name='CuO_cores', volume='0.5 mL',
mass_concentration='12 mg/mL', lookup_missing_values=False)

    L_Arginine = Chemical(container=L_ArgContainer, name='Arginine',
mass_concentration='10.0 mg/mL', mass='10.0 mg', lookup_missing_values=True)

    Water = Chemical(container=MilliQOT2, name='Water', volume='4.5 mL',
lookup_missing_values=True, is_stock_solution=True)

    TEOS = Chemical(container=TEOSContainer, name='TEOS', volume='100 uL',
lookup_missing_values=True)
```

```python
    stirring_speed = 400  # rpm
    reaction_temperature = 95  # °C
    cooling_temperature = 40   # °C
    heating_time = '3 h'
    temperature_error = 2  # °C
    teos_infusion_rate = '5 mL/min'

    container.add_chemical([Water, CTAB, L_Arginine, CuO_cores])

    container.sonicate(sonicator=probesonicator, sonication_time=sonication_time,
sonication_power=20, sonication_amplitude=20, bottom_clearance=12,
container_for_cleaning=falcontube_for_cleaning)

    container.infuse_while_heating(chemical=TEOS,
                                   heating_temperature=reaction_temperature,
                                   stirring_speed=stirring_speed,
                                   heating_time=heating_time,
                                   addition_hardware=valve2,
                                   withdraw_rate='5 mL/min',
                                   addition_rate='5 mL/min',
                                   priming_volume='1.0 mL',
                                   purging_volume='2.5 mL',
                                   purging_port=0,
                                   priming_waste_container=WasteContainerValve2,
                                   cooldown_temperature=cooling_temperature,
                                   maximum_temperature_deviation=temperature_error,
                                   temperature_stabilization_time='30 min',
                                   active_cooling=True)

    container.transfer_content_to_container(target_containers=[falcontube1,
falcontube2], transfer_hardware=valve1, bottom_clearance_withdrawing=8,
dropoff_locations=[(flaskstation, 0), (falcon_tube_holder_15ml, 0),
(falcon_tube_holder_15ml, 0)])

def NH4NO3_extraction(container: Container, falcontube1: Container, falcontube2:
Container, falcontube3: Container, falcontube4: Container):
    stirring_speed = 300  # rpm
    reaction_temperature = 72  # °C
    heating_time = '1.5 h'
    temperature_error = 2  # °C
    cooling_temperature = 40  # °C

    falcontube1.transfer_content_to_container(container, transfer_hardware=valve1,
bottom_clearance_withdrawing=7)
```

```
    falcontube2.transfer_content_to_container(container, transfer_hardware=valve1,
bottom_clearance_withdrawing=7)


    container.heat(heating_temperature=reaction_temperature,
stirring_speed=stirring_speed, heating_time=heating_time,
cooldown_temperature=cooling_temperature, active_cooling=True,
temperature_stabilization_time='10 min')


    container.transfer_content_to_container(target_containers=[falcontube3,
falcontube4], transfer_hardware=valve1, bottom_clearance_withdrawing=8,
dropoff_locations=[(flaskstation, 0), (falcon_tube_holder_15ml, 0),
(falcon_tube_holder_15ml, 0)])


if __name__ == '__main__':

    esb = EmergencyStopButton.EmergencyStopButton(com_port='COM34')

    eln =
Minerva.Software.OpenBISELNIntegration.OpenBisElnIntegration.ElectronicLabNotebook(
space_name='1.0_MINERVA', project_code='CORE_SHELL')

    local_server = LocalPCServer.LocalPCServer(com_port='COM7')

    arduino = ArduinoController.ArduinoController(com_port='COM25')

    dht22 = DHT22Sensor.DHT22Sensor(arduino_controller=arduino)

    robotarm = UFactory.XArm6(ip_address='192.168.1.204',
levelling_data_file=os.path.join('..', 'SampleHolder',
'Table_Levelling_Data.json'))

    ot2 = OpentronsOT2.OT2(ip_address='OT2CEP20210918R04.local')

    hotplate1 = IkaHotplate.RCTDigital5(com_port='COM6')

    hotplate2 = IkaHotplate.RCTDigital5(com_port='COM4')

    hotplate3 = IkaHotplate.RCTDigital5(com_port='COM18')

    hotplate1_clamp =
HotplateClamp.HotplateClampDCMotor(arduino_controller=arduino,
parent_hardware=hotplate1, clamp_number=1)

    hotplate2_clamp =
HotplateClamp.HotplateClampDCMotor(arduino_controller=arduino,
parent_hardware=hotplate2, clamp_number=2)

    hotplate3_clamp =
HotplateClamp.HotplateClampDCMotor(arduino_controller=arduino,
parent_hardware=hotplate3, clamp_number=3)

    hotplate_fan_1 = HotplateFan.HotplateFan(hotplate1, arduino_controller=arduino,
fan_number=1)

    hotplate_fan_2 = HotplateFan.HotplateFan(hotplate2, arduino_controller=arduino,
fan_number=2)

    hotplate_fan_3 = HotplateFan.HotplateFan(hotplate3, arduino_controller=arduino,
fan_number=3)

    capper = CapperDecapper.CapperDecapper(arduino_controller=arduino)

    valve1 = SwitchingValve.SwitchingValveVici(com_port='COM3')

    valve2 = SwitchingValve.SwitchingValveVici(com_port='COM14')

    valve3 = SwitchingValve.SwitchingValveVici(com_port='COM17')

    pump1 = WPI.Aladdin(com_port='COM12')
```

```
    pump2 = WPI.Aladdin(com_port='COM9', baud_rate=9600,
pump_type=SyringePumpType.AL_1010)

    pump3 = WPI.Aladdin(com_port='COM5', baud_rate=9600,
pump_type=SyringePumpType.AL_1010)

    probesonicator = Hielscher.UP200ST(ip_address='192.168.233.233')

    centrifuge = Herolab.RobotCen(com_port='COM8', initialize_rotor=True,
home_rotor=True)

    zetasizer = MalvernPanalytical.ZetaSizer(local_controller=local_server)



    gripchange_holder =
SampleHolder.SampleHolder(SampleHolder.SampleHolderDefinitions.Corkring_Small,
deck_position=4)

    hotplate1_holder =
SampleHolder.SampleHolder(SampleHolderDefinitions.Ika_50mL_Heating_Block,
parent_hardware=hotplate1, deck_position=1)

    hotplate2_holder =
SampleHolder.SampleHolder(SampleHolderDefinitions.Ika_50mL_Heating_Block,
parent_hardware=hotplate2, deck_position=2)

    hotplate3_holder =
SampleHolder.SampleHolder(SampleHolderDefinitions.Ika_50mL_Heating_Block,
parent_hardware=hotplate3, deck_position=3)

    ot2_holder_15ml =
SampleHolder.SampleHolder(SampleHolder.SampleHolderDefinitions.Opentrons_15mL_Tube_
Rack, parent_hardware=ot2, deck_position=2)

    ot2_holder_50ml =
SampleHolder.SampleHolder(SampleHolder.SampleHolderDefinitions.Opentrons_50mL_Tube_
Rack, parent_hardware=ot2, deck_position=1)

    ot2_holder_flask_100ml =
SampleHolder.SampleHolder(SampleHolder.SampleHolderDefinitions.Opentrons_100mL_Flas
k_Rack, parent_hardware=ot2, deck_position=6)

    ot2_holder_flask_50ml =
SampleHolder.SampleHolder(SampleHolder.SampleHolderDefinitions.Opentrons_50mL_Flask
_Rack, parent_hardware=ot2, deck_position=3)

    falcon_tube_holder_50ml =
SampleHolder.SampleHolder(SampleHolder.SampleHolderDefinitions.Isolab_50mL_Foldable
_Tube_Rack, deck_position=1)

    falcon_tube_holder_15ml =
SampleHolder.SampleHolder(SampleHolder.SampleHolderDefinitions.Isolab_15mL_Foldable
_Tube_Rack, deck_position=2)

    flaskstation =
SampleHolder.SampleHolder(SampleHolder.SampleHolderDefinitions.Flask_Station,
deck_position=3, leave_even_rows_empty=False)



    flask_1_50ml = Container(flaskstation, slot_number=1,
container_type=ContainerTypeCollection.FLASK_50_ML, current_volume=Volume(0, 'mL'),
has_stirbar=True, name='flask1')

    flask_2_50ml = Container(flaskstation, slot_number=2,
container_type=ContainerTypeCollection.FLASK_50_ML, current_volume=Volume(0, 'mL'),
has_stirbar=True, name='flask2')

    flask_3_50ml = Container(flaskstation, slot_number=3,
container_type=ContainerTypeCollection.FLASK_50_ML, current_volume=Volume(0, 'mL'),
has_stirbar=True, name='flask3')

    flask_1_100ml = Container(flaskstation, slot_number=5,
container_type=ContainerTypeCollection.FLASK_100_ML, current_volume=Volume(0,
'mL'), has_stirbar=False, name='flask5')
```

```
    falcontube1_15ml = Container(falcon_tube_holder_15ml, slot_number=1,
container_type=ContainerTypeCollection.FALCON_TUBE_15_ML, current_volume='0 mL',
is_capped=True)

    falcontube2_15ml = Container(falcon_tube_holder_15ml, slot_number=2,
container_type=ContainerTypeCollection.FALCON_TUBE_15_ML, current_volume='0 mL',
is_capped=True)

    falcontube3_15ml = Container(falcon_tube_holder_15ml, slot_number=3,
container_type=ContainerTypeCollection.FALCON_TUBE_15_ML, current_volume='0 mL',
is_capped=True)

    falcontube4_15ml = Container(falcon_tube_holder_15ml, slot_number=4,
container_type=ContainerTypeCollection.FALCON_TUBE_15_ML, current_volume='0 mL',
is_capped=True)

    falcontube_for_cleaning = Container(falcon_tube_holder_50ml, slot_number=14,
container_type=ContainerTypeCollection.FALCON_TUBE_50_ML, current_volume='40 mL',
is_capped=False)



    WasteContainerValve1 = Container(current_hardware=valve1, slot_number=0,
name='Waste_Container', current_volume='2 L', max_volume='5 L')

    WasteContainerValve2 = Container(current_hardware=valve2, slot_number=6,
name='Waste_ContainerValve2', current_volume='0 mL', max_volume='50 mL')

    DLSCell = Container(current_hardware=valve1, slot_number=4, name='DLS_Cell',
current_volume='0 mL')

    MilliQ_ContainerValve1 = Container(current_hardware=valve1, slot_number=1,
name='MilliQ_ContainerValve1', current_volume='1.2 L')

    Ethanol_wash_container = Container(current_hardware=valve1, slot_number=5,
name='Ethanol_wash', current_volume='5 L')

    NH4NO3Container = Container(current_hardware=valve1, slot_number=6,
name='NH4NO3_Container', current_volume='10 mL')



    Ethanol_wash = Chemical(container=Ethanol_wash_container, name='Ethanol',
volume='6 mL', lookup_missing_values=True)

    Water_wash = Chemical(container=MilliQ_ContainerValve1, name='Water', volume='6
mL', lookup_missing_values=True)



    TEOSContainer = Container(current_hardware=valve2, slot_number=9,
name='TEOS_Container', current_volume='35 mL',
container_type=ContainerTypeCollection.FALCON_TUBE_50_ML)

    MilliQOT2 = Container(current_hardware=ot2, deck_position=8, slot_number=8,
name='MilliQOT2', current_volume='31 mL',
container_type=ContainerTypeCollection.FALCON_TUBE_50_ML)

    CTABContainer = Container(current_hardware=ot2, deck_position=8, slot_number=7,
name='CTAB_Container', current_volume='17 mL',
container_type=ContainerTypeCollection.FALCON_TUBE_50_ML)

    L_ArgContainer = Container(current_hardware=ot2, deck_position=8,
slot_number=1, name='L_ArgContainer', current_volume='3 mL',
container_type=ContainerTypeCollection.FALCON_TUBE_15_ML)

    CuOContainer = Container(current_hardware=ot2, deck_position=8, slot_number=2,
name='CuOContainer', current_volume='5.1 mL',
container_type=ContainerTypeCollection.FALCON_TUBE_15_ML)

    NH4NO3 = Chemical(container=NH4NO3Container, volume='6 mL',
mass_concentration='20 mg/mL', lookup_missing_values=True)
```

```
sonication_time = '20 s'

centrifugation_speed = '14000 rcf'

centrifugation_time = '15 min'

washing_steps = 2

sample_name = 'MZ023'


ot2_configuration = {
    1: ot2_holder_15ml,
    2: ot2_holder_flask_10ml,
    3: ot2_holder_flask_25ml,
    4: ot2_holder_flask_100ml,
    5: None,
    6: None,
    7: None,
    8: 'opentrons_10_tuberack_falcon_4x50ml_6x15ml_conical',
    9: None,
    10:'opentrons_96_tiprack_20ul',    # 'opentrons_96_tiprack_300ul'
    11:'labsolute_96_tiprack_1000ul',
    12:'p1000_single_gen2',  # left pipette
    13:'p20_single_gen2',  # right pipette  'p300_single_gen2'
}


valve1_configuration = {
    0: None,
    1: MilliQ_ContainerValve1,
    2: Ethanol_wash_container,
    3: WasteContainerValve1,
    4: DLSCell,
    5: None,
    6: NH4NO3Container,
    7: None,
    8: 'Outlet',
    9: None,
    10: pump1
}


valve2_configuration = {
    0: None,
    1: hotplate1,
    2: hotplate2,
    3: hotplate3,
    4: MilliQ_ContainerValve2,
    5: None,
    6: WasteContainerValve2,
    7: None,
    8: None,
    9: TEOSContainer,
    10: pump2
}


valve3_configuration = {
    0: None,
    1: hotplate1,
    2: hotplate2,
    3: hotplate3,
    4: MilliQ_ContainerValve3,
    5: None,
    6: None,
    7: None,
    8: None,
    9: WasteContainerValve3,
    10: pump3
}
```

```
    valve2_dead_volumes = {
        0: None,
        1: '1.390 mL',
        2: '1.35 mL',
        3: '1.87 mL',
        4: None,
        5: None,
        6: None,
        7: None,
        8: None,
        9: None,
        10: None
    }


    ot2.set_hardware_configuration(ot2_configuration)

    pump1.set_syringe(Syringes.GLASS_SYRINGE_SOCOREX_50ML,
default_addition_rate='70 mL/min')

    valve1.set_configuration(valve1_configuration)

    pump2.set_syringe(Syringes.GLASS_SYRINGE_SOCOREX_1ML, default_addition_rate='3
mL/min')

    valve2.set_configuration(valve2_configuration)

    valve2.set_dead_volumes(valve2_dead_volumes)

    pump3.set_syringe(Syringes.GLASS_SYRINGE_SOCOREX_5ML, default_addition_rate='20
mL/min')

    valve3.set_configuration(valve3_configuration)



    CuO_CoreShell_Synthesis_parameters = ((flask_1_50ml, falcontube1_15ml,
falcontube2_15ml))



    threads = []

    for i, p in enumerate(CuO_CoreShell_Synthesis_parameters):

        threads.append(threading.Thread(name=f'Thread_{i}',
target=CuO_CoreShell_Synthesis, args=tuple(p)))

        threads[-1].start()



    for t in threads:

        t.join()



    # Washing after synthesis

    falcontube1_15ml.centrifuge([falcontube2_15ml],
centrifugation_speed=centrifugation_speed, centrifugation_time=centrifugation_time)


falcontube1_15ml.remove_supernatant_and_redisperse(waste_container=WasteContainerVa
lve1, sonication_time=sonication_time, redispersion_chemical=Water_wash,
sonication_power=20, sonication_amplitude=20, sonicator=probesonicator,
bottom_clearance_sonication=18, bottom_clearance_withdrawing=12,
container_for_cleaning=falcontube_for_cleaning)


falcontube2_15ml.remove_supernatant_and_redisperse(waste_container=WasteContainerVa
lve1, sonication_time=sonication_time, redispersion_chemical=Water_wash,
sonication_power=20, sonication_amplitude=20, sonicator=probesonicator,
bottom_clearance_sonication=18, bottom_clearance_withdrawing=12,
container_for_cleaning=falcontube_for_cleaning)
```

```
    for _ in range(0, washing_steps-1):

        falcontube1_15ml.centrifuge([falcontube15ml_counter_reaction],
centrifugation_speed=centrifugation_speed, centrifugation_time=centrifugation_time)


falcontube1_15ml.remove_supernatant_and_redisperse(waste_container=WasteContainerVa
lve1, sonication_time=sonication_time, redispersion_chemical=Ethanol_wash,
sonication_power=20, sonication_amplitude=20, sonicator=probesonicator,
bottom_clearance_sonication=18, bottom_clearance_withdrawing=12,
container_for_cleaning=falcontube_for_cleaning)


falcontube2_15ml.remove_supernatant_and_redisperse(waste_container=WasteContainerVa
lve1, sonication_time=sonication_time, redispersion_chemical=Ethanol_wash,
sonication_power=20, sonication_amplitude=20, sonicator=probesonicator,
bottom_clearance_sonication=18, bottom_clearance_withdrawing=12,
container_for_cleaning=falcontube_for_cleaning)

    centrifuge.close_lid()


    # Redispersion in ammonium nitrate

    falcontube1_15ml.centrifuge([falcontube2_15ml],
centrifugation_speed=centrifugation_speed, centrifugation_time=centrifugation_time)


falcontube1_15ml.remove_supernatant_and_redisperse(waste_container=WasteContainerVa
lve1, sonication_time=sonication_time, redispersion_chemical=NH4NO3,
sonication_power=20, sonication_amplitude=20, sonicator=probesonicator,
bottom_clearance_sonication=18, bottom_clearance_withdrawing=12,
container_for_cleaning=falcontube_for_cleaning)


falcontube2_15ml.remove_supernatant_and_redisperse(waste_container=WasteContainerVa
lve1, sonication_time=sonication_time, redispersion_chemical=NH4NO3,
sonication_power=20, sonication_amplitude=20, sonicator=probesonicator,
bottom_clearance_sonication=18, bottom_clearance_withdrawing=12,
container_for_cleaning=falcontube_for_cleaning)


    # extraction protocol

    NH4NO3_Extraction_parameters = ((flask_1_50ml, falcontube1_15ml,
falcontube2_15ml, falcontube3_15ml, falcontube4_15ml))


    threads = []

    for i, p in enumerate(NH4NO3_Extraction_parameters):

        threads.append(threading.Thread(name=f'Thread_{i}',
target=NH4NO3_extraction, args=tuple(p)))

        threads[-1].start()


    for t in threads:

        t.join()


    # washing after extraction

    for _ in range(0, washing_steps):

        falcontube3_15ml.centrifuge([falcontube4_15ml],
centrifugation_speed=centrifugation_speed, centrifugation_time=centrifugation_time)


falcontube3_15ml.remove_supernatant_and_redisperse(waste_container=WasteContainerVa
```

```
lve1, sonication_time=sonication_time, redispersion_chemical=Ethanol_wash,
sonication_power=20, sonication_amplitude=20, sonicator=probesonicator,
bottom_clearance_sonication=15, bottom_clearance_withdrawing=12,
container_for_cleaning=falcontube_for_cleaning)


falcontube4_15ml.remove_supernatant_and_redisperse(waste_container=WasteContainerVa
lve1, sonication_time=sonication_time, redispersion_chemical=Ethanol_wash,
sonication_power=20, sonication_amplitude=20, sonicator=probesonicator,
bottom_clearance_sonication=15, bottom_clearance_withdrawing=12,
container_for_cleaning=falcontube_for_cleaning)

    centrifuge.close_lid()


    #ELN upload

    eln.write_synthesis_step(experiment_name=f"{sample_name1}")
```

5. Exemplary log file

During execution, a detailed log file is written that includes time stamps and log messages from different levels (debugging, information, warnings, and errors). An exemplary log file for the parallel synthesis of three batches of MSNs is given in the following (see also Fig. S6 for the ELN entry that is automatically generated from this log file):

```
2024-09-27 11:45:00,267<11048>:EmergencyStopButton@COM34:INFO - Emergency Stop
Button connected on port COM34.

2024-09-27 11:45:05,452<11048>:ArduinoController@COM25:INFO - Connected to Arduino
Controller on COM25.

2024-09-27 11:45:05,517<20892>:DHT22Sensor@ArduinoController@COM25:INFO -
Temperature: 22.00 C, Humidity: 26.00 %

2024-09-27 11:45:18,866<11048>:RCTDigital5@COM6:INFO - Connected to RCTDigital5 on
COM6.

2024-09-27 11:45:18,912<11048>:RCTDigital5@COM4:INFO - Connected to RCTDigital5 on
COM4.

2024-09-27 11:45:18,957<11048>:RCTDigital5@COM18:INFO - Connected to RCTDigital5 on
COM18.

2024-09-27
11:45:25,527<11048>:HotplateClampDCMotor@RCTDigital5@COM6@ArduinoController@COM25:I
NFO - Clamp1 moved up.

2024-09-27
11:45:26,072<11048>:HotplateClampDCMotor@RCTDigital5@COM4@ArduinoController@COM25:I
NFO - Clamp2 moved up.

2024-09-27
11:45:26,617<11048>:HotplateClampDCMotor@RCTDigital5@COM18@ArduinoController@COM25:
INFO - Clamp3 moved up.

2024-09-27 11:45:26,734<11048>:SwitchingValveVici@COM3:INFO - Connected to Valve on
COM3.

2024-09-27 11:45:26,838<11048>:SwitchingValveVici@COM14:INFO - Connected to Valve
on COM14.

2024-09-27 11:45:26,939<11048>:SwitchingValveVici@COM17:INFO - Connected to Valve
on COM17.

2024-09-27 11:45:26,978<11048>:Aladdin@COM12:INFO - Connected to Pump on COM12.

2024-09-27 11:45:27,015<11048>:Aladdin@COM9:INFO - Connected to Pump on COM9.

2024-09-27 11:45:27,061<11048>:Aladdin@COM5:INFO - Connected to Pump on COM5.

2024-09-27 11:45:27,109<11048>:UP200ST@192.168.233.233:INFO - Probe Sonicator ready
on 192.168.233.233.

2024-09-27 11:45:27,191<11048>:RobotCen@COM8:INFO - Connected to RobotCen on COM8.

2024-09-27 11:45:46,079<11048>:RobotCen@COM8:INFO - Rotor Information: ROT,     7,
4,     2,     2,     1,AF 8.50.3,8,13500,1100

2024-09-27 11:45:46,079<11048>:RobotCen@COM8:INFO - Homing to first rotor
position...

2024-09-27 11:46:17,171<11048>:RobotCen@COM8:INFO - Rotor set to first position
1100

2024-09-27 11:46:17,172<11048>:RobotCen@COM8:INFO - Setting next rotor position...

2024-09-27 11:46:21,532<11048>:RobotCen@COM8:INFO - Bottle number set to 8.

2024-09-27 11:46:21,534<11048>:RobotCen@COM8:INFO - Rotor successfully initialized.

2024-09-27 11:46:21,607<11048>:Aladdin@COM12:INFO - Diameter set to 28.1 mm
```

2024-09-27 11:46:21,607<11048>:Aladdin@COM12:INFO - Default addition rate set to 70.0 mL/min

2024-09-27 11:46:21,627<11048>:Aladdin@COM9:INFO - Diameter set to 11.5 mm

2024-09-27 11:46:21,627<11048>:Aladdin@COM9:INFO - Default addition rate set to 19.08 mL/min

2024-09-27 11:46:21,653<11048>:Aladdin@COM5:INFO - Diameter set to 11.5 mm

2024-09-27 11:46:21,653<11048>:Aladdin@COM5:INFO - Default addition rate set to 19.08 mL/min

2024-09-27 11:46:38,576<10064>:XArm6@192.168.1.204:INFO - Finished moving robot arm to source destination: Flask_Station->deck 3.

2024-09-27 11:47:03,896<10064>:XArm6@192.168.1.204:INFO - Changed grip on container flask1 to a sideways grip.

2024-09-27 11:47:10,929<10064>:XArm6@192.168.1.204:INFO - Finished moving robot arm to target destination: SwitchingValveVici@COM3.

2024-09-27 11:47:11,473<20804>:SwitchingValveVici@COM3:INFO - Performing addition step for chemical Chemical Water: 54.0 mL (MilliQ_ContainerValve1 -> flask1)

2024-09-27 11:47:11,515<20804>:SwitchingValveVici@COM3:INFO - Valve SwitchingValveVici@COM3 is currently in position 8 (connected to Outlet).

2024-09-27 11:47:11,837<20804>:SwitchingValveVici@COM3:INFO - Moved valve SwitchingValveVici@COM3 to position 1 (connected to MilliQ_ContainerValve1).

2024-09-27 11:47:11,920<1316>:Aladdin@COM12:INFO - Infusion/withdrawing volume set to 27.0 mL at a rate of 70.0 mL/min

2024-09-27 11:47:35,366<1316>:Aladdin@COM12:INFO - Finished withdrawing 27.0 mL at a rate of 70.0 mL/min

2024-09-27 11:47:35,394<20804>:SwitchingValveVici@COM3:INFO - Valve SwitchingValveVici@COM3 is currently in position 1 (connected to MilliQ_ContainerValve1).

2024-09-27 11:47:35,715<20804>:SwitchingValveVici@COM3:INFO - Moved valve SwitchingValveVici@COM3 to position 8 (connected to Outlet).

2024-09-27 11:47:35,797<1316>:Aladdin@COM12:INFO - Infusion/withdrawing volume set to 27.0 mL at a rate of 70.0 mL/min

2024-09-27 11:47:59,131<1316>:Aladdin@COM12:INFO - Finished infusing 27.0 mL at a rate of 70.0 mL/min

2024-09-27 11:47:59,161<20804>:SwitchingValveVici@COM3:INFO - Valve SwitchingValveVici@COM3 is currently in position 8 (connected to Outlet).

2024-09-27 11:47:59,480<20804>:SwitchingValveVici@COM3:INFO - Moved valve SwitchingValveVici@COM3 to position 1 (connected to MilliQ_ContainerValve1).

2024-09-27 11:47:59,563<1316>:Aladdin@COM12:INFO - Infusion/withdrawing volume set to 27.0 mL at a rate of 70.0 mL/min

2024-09-27 11:48:22,898<1316>:Aladdin@COM12:INFO - Finished withdrawing 27.0 mL at a rate of 70.0 mL/min

2024-09-27 11:48:22,927<20804>:SwitchingValveVici@COM3:INFO - Valve SwitchingValveVici@COM3 is currently in position 1 (connected to MilliQ_ContainerValve1).

2024-09-27 11:48:23,246<20804>:SwitchingValveVici@COM3:INFO - Moved valve SwitchingValveVici@COM3 to position 8 (connected to Outlet).

2024-09-27 11:48:23,330<1316>:Aladdin@COM12:INFO - Infusion/withdrawing volume set to 27.0 mL at a rate of 70.0 mL/min

2024-09-27 11:48:46,743<1316>:Aladdin@COM12:INFO - Finished infusing 27.0 mL at a rate of 70.0 mL/min

2024-09-27 11:48:46,743<20804>:SwitchingValveVici@COM3:INFO - Finished adding Chemical Water: 54.0 mL (MilliQ_ContainerValve1 -> flask1)

2024-09-27 11:48:47,750<20804>:SwitchingValveVici@COM3:INFO - Performing purging step into container FLASK_100_ML[flask1]: 54.0 mL at SwitchingValveVici@COM3->slot 0...

2024-09-27 11:48:48,896<20804>:SwitchingValveVici@COM3:INFO - Valve SwitchingValveVici@COM3 is currently in position 8 (connected to Outlet).

2024-09-27 11:48:48,896<20804>:SwitchingValveVici@COM3:INFO - Moved valve SwitchingValveVici@COM3 to position 8 (connected to Outlet).

2024-09-27 11:48:48,981<1316>:Aladdin@COM12:INFO - Infusion/withdrawing volume set to 30.0 mL at a rate of 70.0 mL/min

2024-09-27 11:49:14,872<1316>:Aladdin@COM12:INFO - Finished withdrawing 30.0 mL at a rate of 70.0 mL/min

2024-09-27 11:49:16,055<1316>:Aladdin@COM12:INFO - Infusion/withdrawing volume set to 30.0 mL at a rate of 70.0 mL/min

2024-09-27 11:49:41,945<1316>:Aladdin@COM12:INFO - Finished infusing 30.0 mL at a rate of 70.0 mL/min

2024-09-27 11:49:41,946<20804>:Minerva.API.MinervaAPI:SYNTHESIS_STEP - Add Chemical [flask1]: FLASK_100_ML; Chemical Water: 54.0 mL; 70.0 mL/min; 50 mL Syringe

2024-09-27 11:49:43,607<10064>:XArm6@192.168.1.204:INFO - Finished moving robot arm to source destination: SwitchingValveVici@COM3.

2024-09-27 11:50:05,480<10064>:XArm6@192.168.1.204:INFO - Changed grip on container flask1 to a top grip.

2024-09-27 11:50:16,239<10064>:XArm6@192.168.1.204:INFO - Finished moving robot arm to target destination: 3DPrinted_100ml_round_bottom_flask_rack at OT2@OT2CEP20210918R04.local->deck 2.

2024-09-27 11:50:16,794<18048>:OT2:INFO - Automatically created protocol file for addition steps: C:\Users\WS10003-minerva\Desktop\Minerva\OT2_Temp_Protocols\tmp_2024-09-27_11-50-16-784060

2024-09-27 11:50:16,794<18048>:OT2:INFO - The following chemicals will be added when executing this protocol:

2024-09-27 11:50:16,794<18048>:OT2:INFO - Adding Chemical CTAB: 6.0 mL at a rate of 274.7 uL/s (CTAB_Container -> flask1)

2024-09-27 11:50:16,794<18048>:OT2:INFO - Adding Chemical NaOH: 720.0 uL at a rate of 274.7 uL/s (NaOH_Container -> flask1)

2024-09-27 11:50:24,982<18048>:OT2:INFO - Started executing protocol: tmp_2024-09-27_11-50-16-784060

2024-09-27 11:53:34,898<18048>:OT2:INFO - Protocol tmp_2024-09-27_11-50-16-784060 completed.

2024-09-27 11:53:51,781<10908>:Minerva.API.MinervaAPI:WARNING - Some required sample holders of the addition hardware are full: 3DPrinted_100ml_round_bottom_flask_rack at OT2@OT2CEP20210918R04.local->deck 2. Waiting for free slots to become available...

2024-09-27 11:53:57,184<20804>:Minerva.API.MinervaAPI:SYNTHESIS_STEP - Add Chemical [flask1]: FLASK_100_ML; Chemical CTAB: 6.0 mL; 274.7 uL/s; 1000 uL Single Channel Pipette

2024-09-27 11:53:57,185<20804>:Minerva.API.MinervaAPI:SYNTHESIS_STEP - Add Chemical [flask1]: FLASK_100_ML; Chemical NaOH: 720.0 uL; 274.7 uL/s; 1000 uL Single Channel Pipette

2024-09-27 11:53:57,730<20804>:HotplateClampDCMotor@RCTDigital5@COM6@ArduinoController@COM25:INFO - Clamp1 moved up.

2024-09-27 11:53:57,758<20804>:HotplateClampDCMotor@RCTDigital5@COM6@ArduinoController@COM25:INFO - Clamp1 opened.

2024-09-27 11:54:06,696<10064>:XArm6@192.168.1.204:INFO - Finished moving robot arm to source destination: 3DPrinted_100ml_round_bottom_flask_rack at OT2@OT2CEP20210918R04.local->deck 2.

2024-09-27 11:54:23,405<10064>:XArm6@192.168.1.204:INFO - Changed grip on container flask1 to a sideways grip.

2024-09-27 11:54:42,364<10064>:XArm6@192.168.1.204:INFO - Finished moving robot arm to target destination: Ika_100mL_Heating_Block at RCTDigital5@COM6->deck 1.

2024-09-27 11:54:57,263<20804>:HotplateClampDCMotor@RCTDigital5@COM6@ArduinoController@COM25:INFO - Clamp1 closed.

2024-09-27 11:55:11,651<10064>:XArm6@192.168.1.204:INFO - Finished moving robot arm to source destination: Flask_Station->deck 3.

2024-09-27 11:55:16,605<20804>:HotplateClampDCMotor@RCTDigital5@COM6@ArduinoController@COM25:INFO - Clamp1 moved down.

2024-09-27 11:55:16,606<23056>:RCTDigital5@COM6:INFO - Stirring Speed setpoint changed to: 300 rpm

2024-09-27 11:55:16,606<23056>:RCTDigital5@COM6:INFO - Started stirring...

2024-09-27 11:55:16,607<23056>:RCTDigital5@COM6:INFO - Temperature setpoint changed to: 94 degrees Celsius

2024-09-27 11:55:16,607<23056>:RCTDigital5@COM6:INFO - Started heating...

2024-09-27 11:55:16,685<23056>:RCTDigital5@COM6:INFO - Waiting for temperature to stabilize: 89.3 C / 94 C

2024-09-27 11:55:36,581<10064>:XArm6@192.168.1.204:INFO - Changed grip on container flask2 to a sideways grip.

2024-09-27 11:55:43,621<10064>:XArm6@192.168.1.204:INFO - Finished moving robot arm to target destination: SwitchingValveVici@COM3.

2024-09-27 11:55:44,168<10908>:SwitchingValveVici@COM3:INFO - Performing addition step for chemical Chemical Water: 54.0 mL (MilliQ_ContainerValve1 -> flask2)

2024-09-27 11:55:44,202<10908>:SwitchingValveVici@COM3:INFO - Valve SwitchingValveVici@COM3 is currently in position 8 (connected to Outlet).

2024-09-27 11:55:44,522<10908>:SwitchingValveVici@COM3:INFO - Moved valve SwitchingValveVici@COM3 to position 1 (connected to MilliQ_ContainerValve1).

2024-09-27 11:55:44,607<1316>:Aladdin@COM12:INFO - Infusion/withdrawing volume set to 27.0 mL at a rate of 70.0 mL/min

2024-09-27 11:55:46,732<23056>:RCTDigital5@COM6:INFO - Waiting for temperature to stabilize: 90.1 C / 94 C

2024-09-27 11:56:08,067<1316>:Aladdin@COM12:INFO - Finished withdrawing 27.0 mL at a rate of 70.0 mL/min

2024-09-27 11:56:08,096<10908>:SwitchingValveVici@COM3:INFO - Valve SwitchingValveVici@COM3 is currently in position 1 (connected to MilliQ_ContainerValve1).

2024-09-27 11:56:08,430<10908>:SwitchingValveVici@COM3:INFO - Moved valve SwitchingValveVici@COM3 to position 8 (connected to Outlet).

2024-09-27 11:56:08,515<1316>:Aladdin@COM12:INFO - Infusion/withdrawing volume set to 27.0 mL at a rate of 70.0 mL/min

2024-09-27 11:56:16,778<23056>:RCTDigital5@COM6:INFO - Waiting for temperature to stabilize: 90.8 C / 94 C

2024-09-27 11:56:31,975<1316>:Aladdin@COM12:INFO - Finished infusing 27.0 mL at a rate of 70.0 mL/min

2024-09-27 11:56:32,005<10908>:SwitchingValveVici@COM3:INFO - Valve SwitchingValveVici@COM3 is currently in position 8 (connected to Outlet).

2024-09-27 11:56:32,325<10908>:SwitchingValveVici@COM3:INFO - Moved valve SwitchingValveVici@COM3 to position 1 (connected to MilliQ_ContainerValve1).

2024-09-27 11:56:32,406<1316>:Aladdin@COM12:INFO - Infusion/withdrawing volume set to 27.0 mL at a rate of 70.0 mL/min

2024-09-27 11:56:46,809<23056>:RCTDigital5@COM6:INFO - Waiting for temperature to stabilize: 91.4 C / 94 C

2024-09-27 11:56:55,724<1316>:Aladdin@COM12:INFO - Finished withdrawing 27.0 mL at a rate of 70.0 mL/min

2024-09-27 11:56:55,755<10908>:SwitchingValveVici@COM3:INFO - Valve SwitchingValveVici@COM3 is currently in position 1 (connected to MilliQ_ContainerValve1).

2024-09-27 11:56:56,088<10908>:SwitchingValveVici@COM3:INFO - Moved valve SwitchingValveVici@COM3 to position 8 (connected to Outlet).

2024-09-27 11:56:56,170<1316>:Aladdin@COM12:INFO - Infusion/withdrawing volume set to 27.0 mL at a rate of 70.0 mL/min

2024-09-27 11:57:16,856<23056>:RCTDigital5@COM6:INFO - Waiting for temperature to stabilize: 91.8 C / 94 C

2024-09-27 11:57:19,439<1316>:Aladdin@COM12:INFO - Finished infusing 27.0 mL at a rate of 70.0 mL/min

2024-09-27 11:57:19,440<10908>:SwitchingValveVici@COM3:INFO - Finished adding Chemical Water: 54.0 mL (MilliQ_ContainerValve1 -> flask2)

2024-09-27 11:57:20,447<10908>:SwitchingValveVici@COM3:INFO - Performing purging step into container FLASK_100_ML[flask2]: 54.0 mL at SwitchingValveVici@COM3->slot 0...

2024-09-27 11:57:21,584<10908>:SwitchingValveVici@COM3:INFO - Valve SwitchingValveVici@COM3 is currently in position 8 (connected to Outlet).

2024-09-27 11:57:21,584<10908>:SwitchingValveVici@COM3:INFO - Moved valve SwitchingValveVici@COM3 to position 8 (connected to Outlet).

2024-09-27 11:57:21,676<1316>:Aladdin@COM12:INFO - Infusion/withdrawing volume set to 30.0 mL at a rate of 70.0 mL/min

2024-09-27 11:57:46,902<23056>:RCTDigital5@COM6:INFO - Waiting for temperature to stabilize: 92.3 C / 94 C, stable within specification since 30 seconds.

2024-09-27 11:57:47,679<1316>:Aladdin@COM12:INFO - Finished withdrawing 30.0 mL at a rate of 70.0 mL/min

2024-09-27 11:57:48,878<1316>:Aladdin@COM12:INFO - Infusion/withdrawing volume set to 30.0 mL at a rate of 70.0 mL/min

2024-09-27 11:58:14,767<1316>:Aladdin@COM12:INFO - Finished infusing 30.0 mL at a rate of 70.0 mL/min

2024-09-27 11:58:14,768<10908>:Minerva.API.MinervaAPI:SYNTHESIS_STEP - Add Chemical [flask2]: FLASK_100_ML; Chemical Water: 54.0 mL; 70.0 mL/min; 50 mL Syringe

2024-09-27 11:58:16,425<10064>:XArm6@192.168.1.204:INFO - Finished moving robot arm to source destination: SwitchingValveVici@COM3.

2024-09-27 11:58:16,916<23056>:RCTDigital5@COM6:INFO - Temperature stable at 94 +/- 2.0 degrees Celsius

2024-09-27 11:58:16,916<20804>:Minerva.API.MinervaAPI:SYNTHESIS_STEP - Heat and Stir [flask1]: 94 degrees Celsius; 300 rpm; 1800.0 seconds

2024-09-27 11:58:38,145<10064>:XArm6@192.168.1.204:INFO - Changed grip on container flask2 to a top grip.

2024-09-27 11:58:47,248<10064>:XArm6@192.168.1.204:INFO - Finished moving robot arm to target destination: 3DPrinted_100ml_round_bottom_flask_rack at OT2@OT2CEP20210918R04.local->deck 2.

2024-09-27 11:58:49,418<18048>:OT2:INFO - Automatically created protocol file for addition steps: C:\Users\WS10003-minerva\Desktop\Minerva\OT2_Temp_Protocols\tmp_2024-09-27_11-58-49-410869

2024-09-27 11:58:49,418<18048>:OT2:INFO - The following chemicals will be added when executing this protocol:

2024-09-27 11:58:49,418<18048>:OT2:INFO - Adding Chemical CTAB: 6.0 mL at a rate of 274.7 uL/s (CTAB_Container -> flask2)

2024-09-27 11:58:49,419<18048>:OT2:INFO - Adding Chemical NaOH: 720.0 uL at a rate of 274.7 uL/s (NaOH_Container -> flask2)

2024-09-27 11:58:58,005<18048>:OT2:INFO - Started executing protocol: tmp_2024-09-27_11-58-49-410869

2024-09-27 12:01:21,901<22280>:Minerva.API.MinervaAPI:WARNING - Some required sample holders of the addition hardware are full: 3DPrinted_100ml_round_bottom_flask_rack at OT2@OT2CEP20210918R04.local->deck 2. Waiting for free slots to become available...

2024-09-27 12:02:06,725<18048>:OT2:INFO - Protocol tmp_2024-09-27_11-58-49-410869 completed.

2024-09-27 12:02:28,800<10908>:Minerva.API.MinervaAPI:SYNTHESIS_STEP - Add Chemical [flask2]: FLASK_100_ML; Chemical CTAB: 6.0 mL; 274.7 uL/s; 1000 uL Single Channel Pipette

2024-09-27 12:02:28,801<10908>:Minerva.API.MinervaAPI:SYNTHESIS_STEP - Add Chemical [flask2]: FLASK_100_ML; Chemical NaOH: 720.0 uL; 274.7 uL/s; 1000 uL Single Channel Pipette

2024-09-27 12:02:29,345<10908>:HotplateClampDCMotor@RCTDigital5@COM4@ArduinoController@COM25:INFO - Clamp2 moved up.

2024-09-27 12:02:29,373<10908>:HotplateClampDCMotor@RCTDigital5@COM4@ArduinoController@COM25:INFO - Clamp2 opened.

2024-09-27 12:02:36,191<10064>:XArm6@192.168.1.204:INFO - Finished moving robot arm to source destination: 3DPrinted_100ml_round_bottom_flask_rack at OT2@OT2CEP20210918R04.local->deck 2.

2024-09-27 12:02:54,201<10064>:XArm6@192.168.1.204:INFO - Changed grip on container flask2 to a sideways grip.

2024-09-27 12:03:14,682<10064>:XArm6@192.168.1.204:INFO - Finished moving robot arm to target destination: Ika_100mL_Heating_Block at RCTDigital5@COM4->deck 2.

2024-09-27 12:03:30,163<10908>:HotplateClampDCMotor@RCTDigital5@COM4@ArduinoController@COM25:INFO - Clamp2 closed.

2024-09-27 12:03:44,453<10064>:XArm6@192.168.1.204:INFO - Finished moving robot arm to source destination: Flask_Station->deck 3.

2024-09-27 12:03:48,909<10908>:HotplateClampDCMotor@RCTDigital5@COM4@ArduinoController@COM25:INFO - Clamp2 moved down.

2024-09-27 12:03:48,910<20104>:RCTDigital5@COM4:INFO - Stirring Speed setpoint changed to: 300 rpm

2024-09-27 12:03:48,910<20104>:RCTDigital5@COM4:INFO - Started stirring...

2024-09-27 12:03:48,911<20104>:RCTDigital5@COM4:INFO - Temperature setpoint changed to: 94 degrees Celsius

2024-09-27 12:03:48,911<20104>:RCTDigital5@COM4:INFO - Started heating...

2024-09-27 12:03:48,987<20104>:RCTDigital5@COM4:INFO - Waiting for temperature to stabilize: 89.1 C / 94 C

2024-09-27 12:04:09,499<10064>:XArm6@192.168.1.204:INFO - Changed grip on container flask3 to a sideways grip.

2024-09-27 12:04:16,533<10064>:XArm6@192.168.1.204:INFO - Finished moving robot arm to target destination: SwitchingValveVici@COM3.

2024-09-27 12:04:17,079<22280>:SwitchingValveVici@COM3:INFO - Performing addition step for chemical Chemical Water: 54.0 mL (MilliQ_ContainerValve1 -> flask3)

2024-09-27 12:04:17,118<22280>:SwitchingValveVici@COM3:INFO - Valve SwitchingValveVici@COM3 is currently in position 8 (connected to Outlet).

2024-09-27 12:04:17,440<22280>:SwitchingValveVici@COM3:INFO - Moved valve SwitchingValveVici@COM3 to position 1 (connected to MilliQ_ContainerValve1).

2024-09-27 12:04:17,526<1316>:Aladdin@COM12:INFO - Infusion/withdrawing volume set to 27.0 mL at a rate of 70.0 mL/min

2024-09-27 12:04:19,031<20104>:RCTDigital5@COM4:INFO - Waiting for temperature to stabilize: 90.1 C / 94 C

2024-09-27 12:04:41,018<1316>:Aladdin@COM12:INFO - Finished withdrawing 27.0 mL at a rate of 70.0 mL/min

2024-09-27 12:04:41,048<22280>:SwitchingValveVici@COM3:INFO - Valve SwitchingValveVici@COM3 is currently in position 1 (connected to MilliQ_ContainerValve1).

2024-09-27 12:04:41,367<22280>:SwitchingValveVici@COM3:INFO - Moved valve SwitchingValveVici@COM3 to position 8 (connected to Outlet).

2024-09-27 12:04:41,449<1316>:Aladdin@COM12:INFO - Infusion/withdrawing volume set to 27.0 mL at a rate of 70.0 mL/min

2024-09-27 12:04:49,076<20104>:RCTDigital5@COM4:INFO - Waiting for temperature to stabilize: 90.7 C / 94 C

2024-09-27 12:05:04,926<1316>:Aladdin@COM12:INFO - Finished infusing 27.0 mL at a rate of 70.0 mL/min

2024-09-27 12:05:04,957<22280>:SwitchingValveVici@COM3:INFO - Valve SwitchingValveVici@COM3 is currently in position 8 (connected to Outlet).

2024-09-27 12:05:05,276<22280>:SwitchingValveVici@COM3:INFO - Moved valve SwitchingValveVici@COM3 to position 1 (connected to MilliQ_ContainerValve1).

2024-09-27 12:05:05,374<1316>:Aladdin@COM12:INFO - Infusion/withdrawing volume set to 27.0 mL at a rate of 70.0 mL/min

2024-09-27 12:05:19,120<20104>:RCTDigital5@COM4:INFO - Waiting for temperature to stabilize: 91.4 C / 94 C

2024-09-27 12:05:28,849<1316>:Aladdin@COM12:INFO - Finished withdrawing 27.0 mL at a rate of 70.0 mL/min

2024-09-27 12:05:28,883<22280>:SwitchingValveVici@COM3:INFO - Valve SwitchingValveVici@COM3 is currently in position 1 (connected to MilliQ_ContainerValve1).

2024-09-27 12:05:29,203<22280>:SwitchingValveVici@COM3:INFO - Moved valve SwitchingValveVici@COM3 to position 8 (connected to Outlet).

2024-09-27 12:05:29,297<1316>:Aladdin@COM12:INFO - Infusion/withdrawing volume set to 27.0 mL at a rate of 70.0 mL/min

2024-09-27 12:05:49,179<20104>:RCTDigital5@COM4:INFO - Waiting for temperature to stabilize: 91.9 C / 94 C

2024-09-27 12:05:52,644<1316>:Aladdin@COM12:INFO - Finished infusing 27.0 mL at a rate of 70.0 mL/min

2024-09-27 12:05:52,645<22280>:SwitchingValveVici@COM3:INFO - Finished adding Chemical Water: 54.0 mL (MilliQ_ContainerValve1 -> flask3)

2024-09-27 12:05:53,662<22280>:SwitchingValveVici@COM3:INFO - Performing purging step into container FLASK_100_ML[flask3]: 54.0 mL at SwitchingValveVici@COM3->slot 0...

2024-09-27 12:05:54,804<22280>:SwitchingValveVici@COM3:INFO - Valve SwitchingValveVici@COM3 is currently in position 8 (connected to Outlet).

2024-09-27 12:05:54,805<22280>:SwitchingValveVici@COM3:INFO - Moved valve SwitchingValveVici@COM3 to position 8 (connected to Outlet).

2024-09-27 12:05:54,898<1316>:Aladdin@COM12:INFO - Infusion/withdrawing volume set to 30.0 mL at a rate of 70.0 mL/min

2024-09-27 12:06:19,226<20104>:RCTDigital5@COM4:INFO - Waiting for temperature to stabilize: 92.3 C / 94 C, stable within specification since 30 seconds.

2024-09-27 12:06:20,787<1316>:Aladdin@COM12:INFO - Finished withdrawing 30.0 mL at a rate of 70.0 mL/min

2024-09-27 12:06:21,986<1316>:Aladdin@COM12:INFO - Infusion/withdrawing volume set to 30.0 mL at a rate of 70.0 mL/min

2024-09-27 12:06:47,875<1316>:Aladdin@COM12:INFO - Finished infusing 30.0 mL at a rate of 70.0 mL/min

2024-09-27 12:06:47,876<22280>:Minerva.API.MinervaAPI:SYNTHESIS_STEP - Add Chemical [flask3]: FLASK_100_ML; Chemical Water: 54.0 mL; 70.0 mL/min; 50 mL Syringe

2024-09-27 12:06:49,241<20104>:RCTDigital5@COM4:INFO - Temperature stable at 94 +/- 2.0 degrees Celsius

2024-09-27 12:06:49,241<10908>:Minerva.API.MinervaAPI:SYNTHESIS_STEP - Heat and Stir [flask2]: 94 degrees Celsius; 300 rpm; 1800.0 seconds

2024-09-27 12:06:49,534<10064>:XArm6@192.168.1.204:INFO - Finished moving robot arm to source destination: SwitchingValveVici@COM3.

2024-09-27 12:07:11,077<10064>:XArm6@192.168.1.204:INFO - Changed grip on container flask3 to a top grip.

2024-09-27 12:07:20,222<10064>:XArm6@192.168.1.204:INFO - Finished moving robot arm to target destination: 3DPrinted_100ml_round_bottom_flask_rack at OT2@OT2CEP20210918R04.local->deck 2.

2024-09-27 12:07:22,394<18048>:OT2:INFO - Automatically created protocol file for addition steps: C:\Users\WS10003-minerva\Desktop\Minerva\OT2_Temp_Protocols\tmp_2024-09-27_12-07-22-386328

2024-09-27 12:07:22,395<18048>:OT2:INFO - The following chemicals will be added when executing this protocol:

2024-09-27 12:07:22,395<18048>:OT2:INFO - Adding Chemical CTAB: 6.0 mL at a rate of 274.7 uL/s (CTAB_Container -> flask3)

2024-09-27 12:07:22,395<18048>:OT2:INFO - Adding Chemical NaOH: 720.0 uL at a rate of 274.7 uL/s (NaOH_Container -> flask3)

2024-09-27 12:07:30,241<18048>:OT2:INFO - Started executing protocol: tmp_2024-09-27_12-07-22-386328

2024-09-27 12:10:36,223<18048>:OT2:INFO - Protocol tmp_2024-09-27_12-07-22-386328 completed.

2024-09-27 12:10:58,575<22280>:Minerva.API.MinervaAPI:SYNTHESIS_STEP - Add Chemical [flask3]: FLASK_100_ML; Chemical CTAB: 6.0 mL; 274.7 uL/s; 1000 uL Single Channel Pipette

2024-09-27 12:10:58,575<22280>:Minerva.API.MinervaAPI:SYNTHESIS_STEP - Add Chemical [flask3]: FLASK_100_ML; Chemical NaOH: 720.0 uL; 274.7 uL/s; 1000 uL Single Channel Pipette

2024-09-27 12:10:59,123<22280>:HotplateClampDCMotor@RCTDigital5@COM18@ArduinoController@COM25: INFO - Clamp3 moved up.

2024-09-27 12:10:59,152<22280>:HotplateClampDCMotor@RCTDigital5@COM18@ArduinoController@COM25: INFO - Clamp3 opened.

2024-09-27 12:11:08,102<10064>:XArm6@192.168.1.204:INFO - Finished moving robot arm to source destination: 3DPrinted_100ml_round_bottom_flask_rack at OT2@OT2CEP20210918R04.local->deck 2.

2024-09-27 12:11:24,974<10064>:XArm6@192.168.1.204:INFO - Changed grip on container flask3 to a sideways grip.

2024-09-27 12:11:45,331<10064>:XArm6@192.168.1.204:INFO - Finished moving robot arm to target destination: Ika_100mL_Heating_Block at RCTDigital5@COM18->deck 3.

2024-09-27 12:12:02,237<22280>:HotplateClampDCMotor@RCTDigital5@COM18@ArduinoController@COM25:INFO - Clamp3 closed.

2024-09-27 12:12:21,291<22280>:HotplateClampDCMotor@RCTDigital5@COM18@ArduinoController@COM25:INFO - Clamp3 moved down.

2024-09-27 12:12:21,291<9732>:RCTDigital5@COM18:INFO - Stirring Speed setpoint changed to: 300 rpm

2024-09-27 12:12:21,292<9732>:RCTDigital5@COM18:INFO - Started stirring...

2024-09-27 12:12:21,292<9732>:RCTDigital5@COM18:INFO - Temperature setpoint changed to: 94 degrees Celsius

2024-09-27 12:12:21,292<9732>:RCTDigital5@COM18:INFO - Started heating...

2024-09-27 12:12:21,361<9732>:RCTDigital5@COM18:INFO - Waiting for temperature to stabilize: 89.2 C / 94 C

2024-09-27 12:12:51,406<9732>:RCTDigital5@COM18:INFO - Waiting for temperature to stabilize: 90.0 C / 94 C

2024-09-27 12:13:21,451<9732>:RCTDigital5@COM18:INFO - Waiting for temperature to stabilize: 90.8 C / 94 C

2024-09-27 12:13:51,498<9732>:RCTDigital5@COM18:INFO - Waiting for temperature to stabilize: 91.3 C / 94 C

2024-09-27 12:14:21,542<9732>:RCTDigital5@COM18:INFO - Waiting for temperature to stabilize: 91.8 C / 94 C

2024-09-27 12:14:51,588<9732>:RCTDigital5@COM18:INFO - Waiting for temperature to stabilize: 92.2 C / 94 C, stable within specification since 30 seconds.

2024-09-27 12:15:21,604<9732>:RCTDigital5@COM18:INFO - Temperature stable at 94 +/- 2.0 degrees Celsius

2024-09-27 12:15:21,604<22280>:Minerva.API.MinervaAPI:SYNTHESIS_STEP - Heat and Stir [flask3]: 94 degrees Celsius; 300 rpm; 1800.0 seconds

2024-09-27 12:28:17,277<23056>:RCTDigital5@COM6:INFO - Stopped heating.

2024-09-27 12:28:17,278<23056>:RCTDigital5@COM6:INFO - Temperature setpoint changed to: 0 degrees Celsius

2024-09-27 12:28:17,323<23056>:RCTDigital5@COM6:INFO - Waiting for hotplate to cool down: 94.0 C / 94 C, below setpoint since 0 seconds.

2024-09-27 12:28:47,369<23056>:RCTDigital5@COM6:INFO - Waiting for hotplate to cool down: 93.4 C / 94 C, below setpoint since 30 seconds.

2024-09-27 12:29:17,381<23056>:RCTDigital5@COM6:INFO - Temperature is now below 94 degrees Celsius.

2024-09-27 12:29:17,381<23056>:RCTDigital5@COM6:INFO - Stopped stirring.

2024-09-27 12:29:17,382<23056>:RCTDigital5@COM6:INFO - Stirring Speed setpoint changed to: 0 rpm

2024-09-27 12:29:17,382<20804>:Minerva.API.MinervaAPI:SYNTHESIS_STEP - Cool down [flask1]: 94 degrees Celsius; Active Cooling False

2024-09-27 12:29:22,751<20804>:HotplateClampDCMotor@RCTDigital5@COM6@ArduinoController@COM25:INFO - Clamp1 moved down.

2024-09-27 12:29:22,752<23056>:RCTDigital5@COM6:INFO - Stirring Speed setpoint changed to: 820 rpm

2024-09-27 12:29:22,752<23056>:RCTDigital5@COM6:INFO - Started stirring...

2024-09-27 12:29:22,753<23056>:RCTDigital5@COM6:INFO - Temperature setpoint changed to: 94 degrees Celsius

2024-09-27 12:29:22,753<23056>:RCTDigital5@COM6:INFO - Started heating...

2024-09-27 12:29:22,831<23056>:RCTDigital5@COM6:INFO - Waiting for temperature to stabilize: 92.2 C / 94 C, stable within specification since 0 seconds.

2024-09-27 12:29:52,876<23056>:RCTDigital5@COM6:INFO - Waiting for temperature to stabilize: 93.1 C / 94 C, stable within specification since 30 seconds.

2024-09-27 12:30:22,890<23056>:RCTDigital5@COM6:INFO - Temperature stable at 94 +/- 2 degrees Celsius

2024-09-27 12:30:22,890<20804>:Minerva.API.MinervaAPI:SYNTHESIS_STEP - Heat and Stir [flask1]: 94 degrees Celsius; 820 rpm; 7200.0 seconds

2024-09-27 12:30:22,891<20804>:Minerva.API.MinervaAPI:INFO - Priming addition hardware SwitchingValveVici@COM14 with chemical Chemical TEOS: 1.0 mL.

2024-09-27 12:30:22,892<20804>:SwitchingValveVici@COM14:INFO - Performing addition step for chemical Chemical TEOS: 1400.0 uL (TEOS_Container -> Waste_ContainerValve2)

2024-09-27 12:30:22,928<20804>:SwitchingValveVici@COM14:INFO - Valve SwitchingValveVici@COM14 is currently in position 9 (connected to TEOS_Container).

2024-09-27 12:30:22,928<20804>:SwitchingValveVici@COM14:INFO - Moved valve SwitchingValveVici@COM14 to position 9 (connected to TEOS_Container).

2024-09-27 12:30:23,017<17088>:Aladdin@COM9:INFO - Infusion/withdrawing volume set to 1400.0 uL at a rate of 19.08 mL/min

2024-09-27 12:30:27,715<17088>:Aladdin@COM9:INFO - Finished withdrawing 1400.0 uL at a rate of 19.08 mL/min

2024-09-27 12:30:27,753<20804>:SwitchingValveVici@COM14:INFO - Valve SwitchingValveVici@COM14 is currently in position 9 (connected to TEOS_Container).

2024-09-27 12:30:28,073<20804>:SwitchingValveVici@COM14:INFO - Moved valve SwitchingValveVici@COM14 to position 6 (connected to Waste_ContainerValve2).

2024-09-27 12:30:28,162<17088>:Aladdin@COM9:INFO - Infusion/withdrawing volume set to 1400.0 uL at a rate of 19.08 mL/min

2024-09-27 12:30:32,861<17088>:Aladdin@COM9:INFO - Finished infusing 1400.0 uL at a rate of 19.08 mL/min

2024-09-27 12:30:32,861<20804>:SwitchingValveVici@COM14:INFO - Finished adding Chemical TEOS: 1400.0 uL (TEOS_Container -> Waste_ContainerValve2)

2024-09-27 12:30:33,871<20804>:SwitchingValveVici@COM14:INFO - Performing purging step into container Container[Waste_ContainerValve2]: 1.4 mL at SwitchingValveVici@COM14->slot 6...

2024-09-27 12:30:33,905<20804>:SwitchingValveVici@COM14:INFO - Valve SwitchingValveVici@COM14 is currently in position 6 (connected to Waste_ContainerValve2).

2024-09-27 12:30:34,049<20804>:SwitchingValveVici@COM14:INFO - Moved valve SwitchingValveVici@COM14 to position 5 (connected to None).

2024-09-27 12:30:34,122<17088>:Aladdin@COM9:INFO - Infusion/withdrawing volume set to 4.0 mL at a rate of 19.08 mL/min

2024-09-27 12:30:46,875<17088>:Aladdin@COM9:INFO - Finished withdrawing 4.0 mL at a rate of 19.08 mL/min

2024-09-27 12:30:46,911<20804>:SwitchingValveVici@COM14:INFO - Valve SwitchingValveVici@COM14 is currently in position 5 (connected to None).

2024-09-27 12:30:47,056<20804>:SwitchingValveVici@COM14:INFO - Moved valve SwitchingValveVici@COM14 to position 6 (connected to Waste_ContainerValve2).

2024-09-27 12:30:47,130<17088>:Aladdin@COM9:INFO - Infusion/withdrawing volume set to 4.0 mL at a rate of 19.08 mL/min

2024-09-27 12:30:59,899<17088>:Aladdin@COM9:INFO - Finished infusing 4.0 mL at a rate of 19.08 mL/min

2024-09-27 12:30:59,899<20804>:SwitchingValveVici@COM14:INFO - Performing addition step for chemical Chemical TEOS: 1.0 mL (TEOS_Container -> flask1)

2024-09-27 12:30:59,933<20804>:SwitchingValveVici@COM14:INFO - Valve SwitchingValveVici@COM14 is currently in position 6 (connected to Waste_ContainerValve2).

2024-09-27 12:31:00,253<20804>:SwitchingValveVici@COM14:INFO - Moved valve SwitchingValveVici@COM14 to position 9 (connected to TEOS_Container).

2024-09-27 12:31:00,330<17088>:Aladdin@COM9:INFO - Infusion/withdrawing volume set to 1.0 mL at a rate of 5.0 mL/min

2024-09-27 12:31:12,603<17088>:Aladdin@COM9:INFO - Finished withdrawing 1.0 mL at a rate of 5.0 mL/min

2024-09-27 12:31:12,635<20804>:SwitchingValveVici@COM14:INFO - Valve SwitchingValveVici@COM14 is currently in position 9 (connected to TEOS_Container).

2024-09-27 12:31:12,875<20804>:SwitchingValveVici@COM14:INFO - Moved valve SwitchingValveVici@COM14 to position 1 (connected to RCTDigital5@COM6).

2024-09-27 12:31:12,938<17088>:Aladdin@COM9:INFO - Infusion/withdrawing volume set to 1.0 mL at a rate of 19.08 mL/min

2024-09-27 12:31:16,279<17088>:Aladdin@COM9:INFO - Finished infusing 1.0 mL at a rate of 19.08 mL/min

2024-09-27 12:31:16,279<20804>:SwitchingValveVici@COM14:INFO - Finished adding Chemical TEOS: 1.0 mL (TEOS_Container -> flask1)

2024-09-27 12:31:17,292<20804>:SwitchingValveVici@COM14:INFO - Performing purging step into container FLASK_100_ML[flask1]: 61.72 mL at SwitchingValveVici@COM14->slot 1...

2024-09-27 12:31:17,332<20804>:SwitchingValveVici@COM14:INFO - Valve SwitchingValveVici@COM14 is currently in position 1 (connected to RCTDigital5@COM6).

2024-09-27 12:31:17,747<20804>:SwitchingValveVici@COM14:INFO - Moved valve SwitchingValveVici@COM14 to position 5 (connected to None).

2024-09-27 12:31:17,829<17088>:Aladdin@COM9:INFO - Infusion/withdrawing volume set to 0.389 mL at a rate of 19.08 mL/min

2024-09-27 12:31:19,284<17088>:Aladdin@COM9:INFO - Finished withdrawing 0.389 mL at a rate of 19.08 mL/min

2024-09-27 12:31:19,315<20804>:SwitchingValveVici@COM14:INFO - Valve SwitchingValveVici@COM14 is currently in position 5 (connected to None).

2024-09-27 12:31:19,714<20804>:SwitchingValveVici@COM14:INFO - Moved valve SwitchingValveVici@COM14 to position 1 (connected to RCTDigital5@COM6).

2024-09-27 12:31:19,795<17088>:Aladdin@COM9:INFO - Infusion/withdrawing volume set to 0.389 mL at a rate of 19.08 mL/min

2024-09-27 12:31:21,265<17088>:Aladdin@COM9:INFO - Finished infusing 0.389 mL at a rate of 19.08 mL/min

2024-09-27 12:31:21,265<20804>:SwitchingValveVici@COM14:INFO - Performing purging step into container FLASK_100_ML[flask1]: 61.72 mL at SwitchingValveVici@COM14->slot 1...

2024-09-27 12:31:21,296<20804>:SwitchingValveVici@COM14:INFO - Valve SwitchingValveVici@COM14 is currently in position 1 (connected to RCTDigital5@COM6).

2024-09-27 12:31:21,709<20804>:SwitchingValveVici@COM14:INFO - Moved valve SwitchingValveVici@COM14 to position 5 (connected to None).

2024-09-27 12:31:21,776<17088>:Aladdin@COM9:INFO - Infusion/withdrawing volume set to 1.0 mL at a rate of 19.08 mL/min

2024-09-27 12:31:25,084<17088>:Aladdin@COM9:INFO - Finished withdrawing 1.0 mL at a rate of 19.08 mL/min

2024-09-27 12:31:25,114<20804>:SwitchingValveVici@COM14:INFO - Valve SwitchingValveVici@COM14 is currently in position 5 (connected to None).

2024-09-27 12:31:25,514<20804>:SwitchingValveVici@COM14:INFO - Moved valve SwitchingValveVici@COM14 to position 1 (connected to RCTDigital5@COM6).

2024-09-27 12:31:25,579<17088>:Aladdin@COM9:INFO - Infusion/withdrawing volume set to 1.0 mL at a rate of 2.0 mL/min

2024-09-27 12:31:55,863<17088>:Aladdin@COM9:INFO - Finished infusing 1.0 mL at a rate of 2.0 mL/min

2024-09-27 12:31:55,863<20804>:SwitchingValveVici@COM14:INFO - Performing purging step into container FLASK_100_ML[flask1]: 61.72 mL at SwitchingValveVici@COM14->slot 1...

2024-09-27 12:31:55,902<20804>:SwitchingValveVici@COM14:INFO - Valve SwitchingValveVici@COM14 is currently in position 1 (connected to RCTDigital5@COM6).

2024-09-27 12:31:56,304<20804>:SwitchingValveVici@COM14:INFO - Moved valve SwitchingValveVici@COM14 to position 5 (connected to None).

2024-09-27 12:31:56,375<17088>:Aladdin@COM9:INFO - Infusion/withdrawing volume set to 4.0 mL at a rate of 19.08 mL/min

2024-09-27 12:32:09,127<17088>:Aladdin@COM9:INFO - Finished withdrawing 4.0 mL at a rate of 19.08 mL/min

2024-09-27 12:32:09,164<20804>:SwitchingValveVici@COM14:INFO - Valve SwitchingValveVici@COM14 is currently in position 5 (connected to None).

2024-09-27 12:32:09,565<20804>:SwitchingValveVici@COM14:INFO - Moved valve SwitchingValveVici@COM14 to position 1 (connected to RCTDigital5@COM6).

2024-09-27 12:32:09,637<17088>:Aladdin@COM9:INFO - Infusion/withdrawing volume set to 4.0 mL at a rate of 10.0 mL/min

2024-09-27 12:32:33,929<17088>:Aladdin@COM9:INFO - Finished infusing 4.0 mL at a rate of 10.0 mL/min

2024-09-27 12:32:33,930<20804>:Minerva.API.MinervaAPI:SYNTHESIS_STEP - Add Chemical [flask1]: FLASK_100_ML; Chemical TEOS: 1.0 mL; 2.0 mL/min; 5 mL Syringe

2024-09-27 12:32:33,961<20804>:SwitchingValveVici@COM14:INFO - Valve SwitchingValveVici@COM14 is currently in position 1 (connected to RCTDigital5@COM6).

2024-09-27 12:32:33,961<20804>:SwitchingValveVici@COM14:INFO - Moved valve SwitchingValveVici@COM14 to position 1 (connected to RCTDigital5@COM6).

2024-09-27 12:32:34,041<17088>:Aladdin@COM9:INFO - Infusion/withdrawing volume set to 4.0 mL at a rate of 19.08 mL/min

2024-09-27 12:32:46,793<17088>:Aladdin@COM9:INFO - Finished withdrawing 4.0 mL at a rate of 19.08 mL/min

2024-09-27 12:32:46,823<20804>:SwitchingValveVici@COM14:INFO - Valve SwitchingValveVici@COM14 is currently in position 1 (connected to RCTDigital5@COM6).

2024-09-27 12:32:47,317<20804>:SwitchingValveVici@COM14:INFO - Moved valve SwitchingValveVici@COM14 to position 6 (connected to Waste_ContainerValve2).

2024-09-27 12:32:47,384<17088>:Aladdin@COM9:INFO - Infusion/withdrawing volume set to 4.0 mL at a rate of 19.08 mL/min

2024-09-27 12:33:00,327<17088>:Aladdin@COM9:INFO - Finished infusing 4.0 mL at a rate of 19.08 mL/min

2024-09-27 12:33:00,328<20804>:SwitchingValveVici@COM14:INFO - Performing addition step for chemical Chemical Water: 1.0 mL (CleaningChemicalValve2 -> Waste_ContainerValve2)

2024-09-27 12:33:00,356<20804>:SwitchingValveVici@COM14:INFO - Valve SwitchingValveVici@COM14 is currently in position 6 (connected to Waste_ContainerValve2).

2024-09-27 12:33:00,595<20804>:SwitchingValveVici@COM14:INFO - Moved valve SwitchingValveVici@COM14 to position 4 (connected to CleaningChemicalValve2).

2024-09-27 12:33:00,663<17088>:Aladdin@COM9:INFO - Infusion/withdrawing volume set to 1.0 mL at a rate of 19.08 mL/min

2024-09-27 12:33:03,955<17088>:Aladdin@COM9:INFO - Finished withdrawing 1.0 mL at a rate of 19.08 mL/min

2024-09-27 12:33:03,997<20804>:SwitchingValveVici@COM14:INFO - Valve SwitchingValveVici@COM14 is currently in position 4 (connected to CleaningChemicalValve2).

2024-09-27 12:33:04,236<20804>:SwitchingValveVici@COM14:INFO - Moved valve SwitchingValveVici@COM14 to position 6 (connected to Waste_ContainerValve2).

2024-09-27 12:33:04,306<17088>:Aladdin@COM9:INFO - Infusion/withdrawing volume set to 1.0 mL at a rate of 19.08 mL/min

2024-09-27 12:33:07,615<17088>:Aladdin@COM9:INFO - Finished infusing 1.0 mL at a rate of 19.08 mL/min

2024-09-27 12:33:07,615<20804>:SwitchingValveVici@COM14:INFO - Finished adding Chemical Water: 1.0 mL (CleaningChemicalValve2 -> Waste_ContainerValve2)

2024-09-27 12:33:08,619<20804>:SwitchingValveVici@COM14:INFO - Performing purging step into container Container[Waste_ContainerValve2]: 6.4 mL at SwitchingValveVici@COM14->slot 6...

2024-09-27 12:33:08,648<20804>:SwitchingValveVici@COM14:INFO - Valve SwitchingValveVici@COM14 is currently in position 6 (connected to Waste_ContainerValve2).

2024-09-27 12:33:08,792<20804>:SwitchingValveVici@COM14:INFO - Moved valve SwitchingValveVici@COM14 to position 5 (connected to None).

2024-09-27 12:33:08,877<17088>:Aladdin@COM9:INFO - Infusion/withdrawing volume set to 0.389 mL at a rate of 19.08 mL/min

2024-09-27 12:33:10,347<17088>:Aladdin@COM9:INFO - Finished withdrawing 0.389 mL at a rate of 19.08 mL/min

2024-09-27 12:33:10,374<20804>:SwitchingValveVici@COM14:INFO - Valve SwitchingValveVici@COM14 is currently in position 5 (connected to None).

2024-09-27 12:33:10,517<20804>:SwitchingValveVici@COM14:INFO - Moved valve SwitchingValveVici@COM14 to position 6 (connected to Waste_ContainerValve2).

2024-09-27 12:33:10,603<17088>:Aladdin@COM9:INFO - Infusion/withdrawing volume set to 0.389 mL at a rate of 19.08 mL/min

2024-09-27 12:33:12,073<17088>:Aladdin@COM9:INFO - Finished infusing 0.389 mL at a rate of 19.08 mL/min

2024-09-27 12:33:12,073<20804>:SwitchingValveVici@COM14:INFO - Performing purging step into container Container[Waste_ContainerValve2]: 6.4 mL at SwitchingValveVici@COM14->slot 6...

2024-09-27 12:33:12,113<20804>:SwitchingValveVici@COM14:INFO - Valve SwitchingValveVici@COM14 is currently in position 6 (connected to Waste_ContainerValve2).

2024-09-27 12:33:12,274<20804>:SwitchingValveVici@COM14:INFO - Moved valve SwitchingValveVici@COM14 to position 5 (connected to None).

2024-09-27 12:33:12,345<17088>:Aladdin@COM9:INFO - Infusion/withdrawing volume set to 1.0 mL at a rate of 19.08 mL/min

2024-09-27 12:33:15,653<17088>:Aladdin@COM9:INFO - Finished withdrawing 1.0 mL at a rate of 19.08 mL/min

2024-09-27 12:33:15,692<20804>:SwitchingValveVici@COM14:INFO - Valve SwitchingValveVici@COM14 is currently in position 5 (connected to None).

2024-09-27 12:33:15,838<20804>:SwitchingValveVici@COM14:INFO - Moved valve SwitchingValveVici@COM14 to position 6 (connected to Waste_ContainerValve2).

2024-09-27 12:33:15,908<17088>:Aladdin@COM9:INFO - Infusion/withdrawing volume set to 1.0 mL at a rate of 19.08 mL/min

2024-09-27 12:33:19,216<17088>:Aladdin@COM9:INFO - Finished infusing 1.0 mL at a rate of 19.08 mL/min

2024-09-27 12:33:19,216<20804>:SwitchingValveVici@COM14:INFO - Performing purging step into container Container[Waste_ContainerValve2]: 6.4 mL at SwitchingValveVici@COM14->slot 6...

2024-09-27 12:33:19,255<20804>:SwitchingValveVici@COM14:INFO - Valve SwitchingValveVici@COM14 is currently in position 6 (connected to Waste_ContainerValve2).

2024-09-27 12:33:19,401<20804>:SwitchingValveVici@COM14:INFO - Moved valve SwitchingValveVici@COM14 to position 5 (connected to None).

2024-09-27 12:33:19,472<17088>:Aladdin@COM9:INFO - Infusion/withdrawing volume set to 4.0 mL at a rate of 19.08 mL/min

2024-09-27 12:33:32,225<17088>:Aladdin@COM9:INFO - Finished withdrawing 4.0 mL at a rate of 19.08 mL/min

2024-09-27 12:33:32,261<20804>:SwitchingValveVici@COM14:INFO - Valve SwitchingValveVici@COM14 is currently in position 5 (connected to None).

2024-09-27 12:33:32,406<20804>:SwitchingValveVici@COM14:INFO - Moved valve SwitchingValveVici@COM14 to position 6 (connected to Waste_ContainerValve2).

2024-09-27 12:33:32,481<17088>:Aladdin@COM9:INFO - Infusion/withdrawing volume set to 4.0 mL at a rate of 19.08 mL/min

2024-09-27 12:33:45,233<17088>:Aladdin@COM9:INFO - Finished infusing 4.0 mL at a rate of 19.08 mL/min

2024-09-27 12:36:49,552<20104>:RCTDigital5@COM4:INFO - Stopped heating.

2024-09-27 12:36:49,552<20104>:RCTDigital5@COM4:INFO - Temperature setpoint changed to: 0 degrees Celsius

2024-09-27 12:36:49,597<20104>:RCTDigital5@COM4:INFO - Waiting for hotplate to cool down: 94.0 C / 94 C, below setpoint since 0 seconds.

2024-09-27 12:37:19,640<20104>:RCTDigital5@COM4:INFO - Waiting for hotplate to cool down: 93.4 C / 94 C, below setpoint since 30 seconds.

2024-09-27 12:37:49,651<20104>:RCTDigital5@COM4:INFO - Temperature is now below 94 degrees Celsius.

2024-09-27 12:37:49,651<20104>:RCTDigital5@COM4:INFO - Stopped stirring.

2024-09-27 12:37:49,651<20104>:RCTDigital5@COM4:INFO - Stirring Speed setpoint changed to: 0 rpm

2024-09-27 12:37:49,652<10908>:Minerva.API.MinervaAPI:SYNTHESIS_STEP - Cool down [flask2]: 94 degrees Celsius; Active Cooling False

2024-09-27 12:37:54,992<10908>:HotplateClampDCMotor@RCTDigital5@COM4@ArduinoController@COM25:INFO - Clamp2 moved down.

2024-09-27 12:37:54,992<20104>:RCTDigital5@COM4:INFO - Stirring Speed setpoint changed to: 820 rpm

2024-09-27 12:37:54,993<20104>:RCTDigital5@COM4:INFO - Started stirring...

2024-09-27 12:37:54,994<20104>:RCTDigital5@COM4:INFO - Temperature setpoint changed to: 94 degrees Celsius

2024-09-27 12:37:54,994<20104>:RCTDigital5@COM4:INFO - Started heating...

2024-09-27 12:37:55,067<20104>:RCTDigital5@COM4:INFO - Waiting for temperature to stabilize: 92.3 C / 94 C, stable within specification since 0 seconds.

2024-09-27 12:38:25,109<20104>:RCTDigital5@COM4:INFO - Waiting for temperature to stabilize: 93.0 C / 94 C, stable within specification since 30 seconds.

2024-09-27 12:38:55,129<20104>:RCTDigital5@COM4:INFO - Temperature stable at 94 +/- 2 degrees Celsius

2024-09-27 12:38:55,129<10908>:Minerva.API.MinervaAPI:SYNTHESIS_STEP - Heat and Stir [flask2]: 94 degrees Celsius; 820 rpm; 7200.0 seconds

2024-09-27 12:38:55,130<10908>:Minerva.API.MinervaAPI:INFO - Priming addition hardware SwitchingValveVici@COM14 with chemical Chemical TEOS: 1.0 mL.

2024-09-27 12:38:55,131<10908>:SwitchingValveVici@COM14:INFO - Performing addition step for chemical Chemical TEOS: 200.0 uL (TEOS_Container -> Waste_ContainerValve2)

2024-09-27 12:38:55,159<10908>:SwitchingValveVici@COM14:INFO - Valve SwitchingValveVici@COM14 is currently in position 6 (connected to Waste_ContainerValve2).

2024-09-27 12:38:55,477<10908>:SwitchingValveVici@COM14:INFO - Moved valve SwitchingValveVici@COM14 to position 9 (connected to TEOS_Container).

2024-09-27 12:38:55,572<17088>:Aladdin@COM9:INFO - Infusion/withdrawing volume set to 200.0 uL at a rate of 19.08 mL/min

2024-09-27 12:38:56,354<17088>:Aladdin@COM9:INFO - Finished withdrawing 200.0 uL at a rate of 19.08 mL/min

2024-09-27 12:38:56,388<10908>:SwitchingValveVici@COM14:INFO - Valve SwitchingValveVici@COM14 is currently in position 9 (connected to TEOS_Container).

2024-09-27 12:38:56,708<10908>:SwitchingValveVici@COM14:INFO - Moved valve SwitchingValveVici@COM14 to position 6 (connected to Waste_ContainerValve2).

2024-09-27 12:38:56,802<17088>:Aladdin@COM9:INFO - Infusion/withdrawing volume set to 200.0 uL at a rate of 19.08 mL/min

2024-09-27 12:38:57,569<17088>:Aladdin@COM9:INFO - Finished infusing 200.0 uL at a rate of 19.08 mL/min

2024-09-27 12:38:57,569<10908>:SwitchingValveVici@COM14:INFO - Finished adding Chemical TEOS: 200.0 uL (TEOS_Container -> Waste_ContainerValve2)

2024-09-27 12:38:58,575<10908>:SwitchingValveVici@COM14:INFO - Performing purging step into container Container[Waste_ContainerValve2]: 6.6 mL at SwitchingValveVici@COM14->slot 6...

2024-09-27 12:38:58,609<10908>:SwitchingValveVici@COM14:INFO - Valve SwitchingValveVici@COM14 is currently in position 6 (connected to Waste_ContainerValve2).

2024-09-27 12:38:58,753<10908>:SwitchingValveVici@COM14:INFO - Moved valve SwitchingValveVici@COM14 to position 5 (connected to None).

2024-09-27 12:38:58,831<17088>:Aladdin@COM9:INFO - Infusion/withdrawing volume set to 4.0 mL at a rate of 19.08 mL/min

2024-09-27 12:39:11,583<17088>:Aladdin@COM9:INFO - Finished withdrawing 4.0 mL at a rate of 19.08 mL/min

2024-09-27 12:39:11,615<10908>:SwitchingValveVici@COM14:INFO - Valve SwitchingValveVici@COM14 is currently in position 5 (connected to None).

2024-09-27 12:39:11,774<10908>:SwitchingValveVici@COM14:INFO - Moved valve SwitchingValveVici@COM14 to position 6 (connected to Waste_ContainerValve2).

2024-09-27 12:39:11,839<17088>:Aladdin@COM9:INFO - Infusion/withdrawing volume set to 4.0 mL at a rate of 19.08 mL/min

2024-09-27 12:39:24,639<17088>:Aladdin@COM9:INFO - Finished infusing 4.0 mL at a rate of 19.08 mL/min

2024-09-27 12:39:24,639<10908>:SwitchingValveVici@COM14:INFO - Performing addition step for chemical Chemical TEOS: 1.0 mL (TEOS_Container -> flask2)

2024-09-27 12:39:24,668<10908>:SwitchingValveVici@COM14:INFO - Valve SwitchingValveVici@COM14 is currently in position 6 (connected to Waste_ContainerValve2).

2024-09-27 12:39:24,987<10908>:SwitchingValveVici@COM14:INFO - Moved valve SwitchingValveVici@COM14 to position 9 (connected to TEOS_Container).

2024-09-27 12:39:25,055<17088>:Aladdin@COM9:INFO - Infusion/withdrawing volume set to 1.0 mL at a rate of 5.0 mL/min

2024-09-27 12:39:37,312<17088>:Aladdin@COM9:INFO - Finished withdrawing 1.0 mL at a rate of 5.0 mL/min

2024-09-27 12:39:37,337<10908>:SwitchingValveVici@COM14:INFO - Valve SwitchingValveVici@COM14 is currently in position 9 (connected to TEOS_Container).

2024-09-27 12:39:37,657<10908>:SwitchingValveVici@COM14:INFO - Moved valve SwitchingValveVici@COM14 to position 2 (connected to RCTDigital5@COM4).

2024-09-27 12:39:37,727<17088>:Aladdin@COM9:INFO - Infusion/withdrawing volume set to 1.0 mL at a rate of 19.08 mL/min

2024-09-27 12:39:41,227<17088>:Aladdin@COM9:INFO - Finished infusing 1.0 mL at a rate of 19.08 mL/min

2024-09-27 12:39:41,227<10908>:SwitchingValveVici@COM14:INFO - Finished adding Chemical TEOS: 1.0 mL (TEOS_Container -> flask2)

2024-09-27 12:39:42,240<10908>:SwitchingValveVici@COM14:INFO - Performing purging step into container FLASK_100_ML[flask2]: 61.72 mL at SwitchingValveVici@COM14->slot 1...

2024-09-27 12:39:42,274<10908>:SwitchingValveVici@COM14:INFO - Valve SwitchingValveVici@COM14 is currently in position 2 (connected to RCTDigital5@COM4).

2024-09-27 12:39:42,593<10908>:SwitchingValveVici@COM14:INFO - Moved valve SwitchingValveVici@COM14 to position 5 (connected to None).

2024-09-27 12:39:42,681<17088>:Aladdin@COM9:INFO - Infusion/withdrawing volume set to 0.35 mL at a rate of 19.08 mL/min

2024-09-27 12:39:43,928<17088>:Aladdin@COM9:INFO - Finished withdrawing 0.35 mL at a rate of 19.08 mL/min

2024-09-27 12:39:43,967<10908>:SwitchingValveVici@COM14:INFO - Valve SwitchingValveVici@COM14 is currently in position 5 (connected to None).

2024-09-27 12:39:44,287<10908>:SwitchingValveVici@COM14:INFO - Moved valve SwitchingValveVici@COM14 to position 2 (connected to RCTDigital5@COM4).

2024-09-27 12:39:44,375<17088>:Aladdin@COM9:INFO - Infusion/withdrawing volume set to 0.35 mL at a rate of 19.08 mL/min

2024-09-27 12:39:45,606<17088>:Aladdin@COM9:INFO - Finished infusing 0.35 mL at a rate of 19.08 mL/min

2024-09-27 12:39:45,606<10908>:SwitchingValveVici@COM14:INFO - Performing purging step into container FLASK_100_ML[flask2]: 61.72 mL at SwitchingValveVici@COM14->slot 1...

2024-09-27 12:39:45,644<10908>:SwitchingValveVici@COM14:INFO - Valve SwitchingValveVici@COM14 is currently in position 2 (connected to RCTDigital5@COM4).

2024-09-27 12:39:45,964<10908>:SwitchingValveVici@COM14:INFO - Moved valve SwitchingValveVici@COM14 to position 5 (connected to None).

2024-09-27 12:39:46,037<17088>:Aladdin@COM9:INFO - Infusion/withdrawing volume set to 1.0 mL at a rate of 19.08 mL/min

2024-09-27 12:39:49,329<17088>:Aladdin@COM9:INFO - Finished withdrawing 1.0 mL at a rate of 19.08 mL/min

2024-09-27 12:39:49,366<10908>:SwitchingValveVici@COM14:INFO - Valve SwitchingValveVici@COM14 is currently in position 5 (connected to None).

2024-09-27 12:39:49,687<10908>:SwitchingValveVici@COM14:INFO - Moved valve SwitchingValveVici@COM14 to position 2 (connected to RCTDigital5@COM4).

2024-09-27 12:39:49,759<17088>:Aladdin@COM9:INFO - Infusion/withdrawing volume set to 1.0 mL at a rate of 2.0 mL/min

2024-09-27 12:40:19,994<17088>:Aladdin@COM9:INFO - Finished infusing 1.0 mL at a rate of 2.0 mL/min

2024-09-27 12:40:19,994<10908>:SwitchingValveVici@COM14:INFO - Performing purging step into container FLASK_100_ML[flask2]: 61.72 mL at SwitchingValveVici@COM14->slot 1...

2024-09-27 12:40:20,028<10908>:SwitchingValveVici@COM14:INFO - Valve SwitchingValveVici@COM14 is currently in position 2 (connected to RCTDigital5@COM4).

2024-09-27 12:40:20,347<10908>:SwitchingValveVici@COM14:INFO - Moved valve SwitchingValveVici@COM14 to position 5 (connected to None).

2024-09-27 12:40:20,425<17088>:Aladdin@COM9:INFO - Infusion/withdrawing volume set to 4.0 mL at a rate of 19.08 mL/min

2024-09-27 12:40:33,194<17088>:Aladdin@COM9:INFO - Finished withdrawing 4.0 mL at a rate of 19.08 mL/min

2024-09-27 12:40:33,225<10908>:SwitchingValveVici@COM14:INFO - Valve SwitchingValveVici@COM14 is currently in position 5 (connected to None).

2024-09-27 12:40:33,545<10908>:SwitchingValveVici@COM14:INFO - Moved valve SwitchingValveVici@COM14 to position 2 (connected to RCTDigital5@COM4).

2024-09-27 12:40:33,609<17088>:Aladdin@COM9:INFO - Infusion/withdrawing volume set to 4.0 mL at a rate of 10.0 mL/min

2024-09-27 12:40:57,884<17088>:Aladdin@COM9:INFO - Finished infusing 4.0 mL at a rate of 10.0 mL/min

2024-09-27 12:40:57,884<10908>:Minerva.API.MinervaAPI:SYNTHESIS_STEP - Add Chemical [flask2]: FLASK_100_ML; Chemical TEOS: 1.0 mL; 2.0 mL/min; 5 mL Syringe

2024-09-27 12:40:57,911<10908>:SwitchingValveVici@COM14:INFO - Valve SwitchingValveVici@COM14 is currently in position 2 (connected to RCTDigital5@COM4).

2024-09-27 12:40:57,912<10908>:SwitchingValveVici@COM14:INFO - Moved valve SwitchingValveVici@COM14 to position 2 (connected to RCTDigital5@COM4).

2024-09-27 12:40:57,979<17088>:Aladdin@COM9:INFO - Infusion/withdrawing volume set to 4.0 mL at a rate of 19.08 mL/min

2024-09-27 12:41:10,763<17088>:Aladdin@COM9:INFO - Finished withdrawing 4.0 mL at a rate of 19.08 mL/min

2024-09-27 12:41:10,788<10908>:SwitchingValveVici@COM14:INFO - Valve SwitchingValveVici@COM14 is currently in position 2 (connected to RCTDigital5@COM4).

2024-09-27 12:41:11,202<10908>:SwitchingValveVici@COM14:INFO - Moved valve SwitchingValveVici@COM14 to position 6 (connected to Waste_ContainerValve2).

2024-09-27 12:41:11,275<17088>:Aladdin@COM9:INFO - Infusion/withdrawing volume set to 4.0 mL at a rate of 19.08 mL/min

2024-09-27 12:41:24,203<17088>:Aladdin@COM9:INFO - Finished infusing 4.0 mL at a rate of 19.08 mL/min

2024-09-27 12:41:24,204<10908>:SwitchingValveVici@COM14:INFO - Performing addition step for chemical Chemical Water: 1.0 mL (CleaningChemicalValve2 -> Waste_ContainerValve2)

2024-09-27 12:41:24,240<10908>:SwitchingValveVici@COM14:INFO - Valve SwitchingValveVici@COM14 is currently in position 6 (connected to Waste_ContainerValve2).

2024-09-27 12:41:24,479<10908>:SwitchingValveVici@COM14:INFO - Moved valve SwitchingValveVici@COM14 to position 4 (connected to CleaningChemicalValve2).

2024-09-27 12:41:24,554<17088>:Aladdin@COM9:INFO - Infusion/withdrawing volume set to 1.0 mL at a rate of 19.08 mL/min

2024-09-27 12:41:28,069<17088>:Aladdin@COM9:INFO - Finished withdrawing 1.0 mL at a rate of 19.08 mL/min

2024-09-27 12:41:28,106<10908>:SwitchingValveVici@COM14:INFO - Valve SwitchingValveVici@COM14 is currently in position 4 (connected to CleaningChemicalValve2).

2024-09-27 12:41:28,346<10908>:SwitchingValveVici@COM14:INFO - Moved valve SwitchingValveVici@COM14 to position 6 (connected to Waste_ContainerValve2).

2024-09-27 12:41:28,421<17088>:Aladdin@COM9:INFO - Infusion/withdrawing volume set to 1.0 mL at a rate of 19.08 mL/min

2024-09-27 12:41:31,746<17088>:Aladdin@COM9:INFO - Finished infusing 1.0 mL at a rate of 19.08 mL/min

2024-09-27 12:41:31,746<10908>:SwitchingValveVici@COM14:INFO - Finished adding Chemical Water: 1.0 mL (CleaningChemicalValve2 -> Waste_ContainerValve2)

2024-09-27 12:41:32,749<10908>:SwitchingValveVici@COM14:INFO - Performing purging step into container Container[Waste_ContainerValve2]: 11.6 mL at SwitchingValveVici@COM14->slot 6...

2024-09-27 12:41:32,787<10908>:SwitchingValveVici@COM14:INFO - Valve SwitchingValveVici@COM14 is currently in position 6 (connected to Waste_ContainerValve2).

2024-09-27 12:41:32,932<10908>:SwitchingValveVici@COM14:INFO - Moved valve SwitchingValveVici@COM14 to position 5 (connected to None).

2024-09-27 12:41:33,023<17088>:Aladdin@COM9:INFO - Infusion/withdrawing volume set to 0.35 mL at a rate of 19.08 mL/min

2024-09-27 12:41:34,462<17088>:Aladdin@COM9:INFO - Finished withdrawing 0.35 mL at a rate of 19.08 mL/min

2024-09-27 12:41:34,497<10908>:SwitchingValveVici@COM14:INFO - Valve SwitchingValveVici@COM14 is currently in position 5 (connected to None).

2024-09-27 12:41:34,642<10908>:SwitchingValveVici@COM14:INFO - Moved valve SwitchingValveVici@COM14 to position 6 (connected to Waste_ContainerValve2).

2024-09-27 12:41:34,734<17088>:Aladdin@COM9:INFO - Infusion/withdrawing volume set to 0.35 mL at a rate of 19.08 mL/min

2024-09-27 12:41:35,979<17088>:Aladdin@COM9:INFO - Finished infusing 0.35 mL at a rate of 19.08 mL/min

2024-09-27 12:41:35,980<10908>:SwitchingValveVici@COM14:INFO - Performing purging step into container Container[Waste_ContainerValve2]: 11.6 mL at SwitchingValveVici@COM14->slot 6...

2024-09-27 12:41:36,016<10908>:SwitchingValveVici@COM14:INFO - Valve SwitchingValveVici@COM14 is currently in position 6 (connected to Waste_ContainerValve2).

2024-09-27 12:41:36,161<10908>:SwitchingValveVici@COM14:INFO - Moved valve SwitchingValveVici@COM14 to position 5 (connected to None).

2024-09-27 12:41:36,236<17088>:Aladdin@COM9:INFO - Infusion/withdrawing volume set to 1.0 mL at a rate of 19.08 mL/min

2024-09-27 12:41:39,543<17088>:Aladdin@COM9:INFO - Finished withdrawing 1.0 mL at a rate of 19.08 mL/min

2024-09-27 12:41:39,577<10908>:SwitchingValveVici@COM14:INFO - Valve SwitchingValveVici@COM14 is currently in position 5 (connected to None).

2024-09-27 12:41:39,737<10908>:SwitchingValveVici@COM14:INFO - Moved valve SwitchingValveVici@COM14 to position 6 (connected to Waste_ContainerValve2).

2024-09-27 12:41:39,815<17088>:Aladdin@COM9:INFO - Infusion/withdrawing volume set to 1.0 mL at a rate of 19.08 mL/min

2024-09-27 12:41:43,140<17088>:Aladdin@COM9:INFO - Finished infusing 1.0 mL at a rate of 19.08 mL/min

2024-09-27 12:41:43,140<10908>:SwitchingValveVici@COM14:INFO - Performing purging step into container Container[Waste_ContainerValve2]: 11.6 mL at SwitchingValveVici@COM14->slot 6...

2024-09-27 12:41:43,174<10908>:SwitchingValveVici@COM14:INFO - Valve SwitchingValveVici@COM14 is currently in position 6 (connected to Waste_ContainerValve2).

2024-09-27 12:41:43,332<10908>:SwitchingValveVici@COM14:INFO - Moved valve SwitchingValveVici@COM14 to position 5 (connected to None).

2024-09-27 12:41:43,411<17088>:Aladdin@COM9:INFO - Infusion/withdrawing volume set to 4.0 mL at a rate of 19.08 mL/min

2024-09-27 12:41:56,132<17088>:Aladdin@COM9:INFO - Finished withdrawing 4.0 mL at a rate of 19.08 mL/min

2024-09-27 12:41:56,162<10908>:SwitchingValveVici@COM14:INFO - Valve SwitchingValveVici@COM14 is currently in position 5 (connected to None).

2024-09-27 12:41:56,320<10908>:SwitchingValveVici@COM14:INFO - Moved valve SwitchingValveVici@COM14 to position 6 (connected to Waste_ContainerValve2).

2024-09-27 12:41:56,388<17088>:Aladdin@COM9:INFO - Infusion/withdrawing volume set to 4.0 mL at a rate of 19.08 mL/min

2024-09-27 12:42:09,316<17088>:Aladdin@COM9:INFO - Finished infusing 4.0 mL at a rate of 19.08 mL/min

2024-09-27 12:45:06,397<20892>:DHT22Sensor@ArduinoController@COM25:INFO - Temperature: 22.00 C, Humidity: 26.00 %

2024-09-27 12:45:21,889<9732>:RCTDigital5@COM18:INFO - Stopped heating.

2024-09-27 12:45:21,889<9732>:RCTDigital5@COM18:INFO - Temperature setpoint changed to: 0 degrees Celsius

2024-09-27 12:45:21,941<9732>:RCTDigital5@COM18:INFO - Waiting for hotplate to cool down: 94.0 C / 94 C, below setpoint since 0 seconds.

2024-09-27 12:45:51,985<9732>:RCTDigital5@COM18:INFO - Waiting for hotplate to cool down: 93.5 C / 94 C, below setpoint since 30 seconds.

2024-09-27 12:46:21,996<9732>:RCTDigital5@COM18:INFO - Temperature is now below 94 degrees Celsius.

2024-09-27 12:46:21,996<9732>:RCTDigital5@COM18:INFO - Stopped stirring.

2024-09-27 12:46:21,996<9732>:RCTDigital5@COM18:INFO - Stirring Speed setpoint changed to: 0 rpm

2024-09-27 12:46:21,997<22280>:Minerva.API.MinervaAPI:SYNTHESIS_STEP - Cool down [flask3]: 94 degrees Celsius; Active Cooling False

2024-09-27 12:46:27,323<22280>:HotplateClampDCMotor@RCTDigital5@COM18@ArduinoController@COM25:INFO - Clamp3 moved down.

2024-09-27 12:46:27,323<9732>:RCTDigital5@COM18:INFO - Stirring Speed setpoint changed to: 820 rpm

2024-09-27 12:46:27,324<9732>:RCTDigital5@COM18:INFO - Started stirring...

2024-09-27 12:46:27,325<9732>:RCTDigital5@COM18:INFO - Temperature setpoint changed to: 94 degrees Celsius

2024-09-27 12:46:27,325<9732>:RCTDigital5@COM18:INFO - Started heating...

2024-09-27 12:46:27,399<9732>:RCTDigital5@COM18:INFO - Waiting for temperature to stabilize: 92.4 C / 94 C, stable within specification since 0 seconds.

2024-09-27 12:46:57,442<9732>:RCTDigital5@COM18:INFO - Waiting for temperature to stabilize: 93.6 C / 94 C, stable within specification since 30 seconds.

2024-09-27 12:47:27,455<9732>:RCTDigital5@COM18:INFO - Temperature stable at 94 +/- 2 degrees Celsius

2024-09-27 12:47:27,455<22280>:Minerva.API.MinervaAPI:SYNTHESIS_STEP - Heat and Stir [flask3]: 94 degrees Celsius; 820 rpm; 7200.0 seconds

2024-09-27 12:47:27,456<22280>:Minerva.API.MinervaAPI:INFO - Priming addition hardware SwitchingValveVici@COM14 with chemical Chemical TEOS: 1.0 mL.

2024-09-27 12:47:27,457<22280>:SwitchingValveVici@COM14:INFO - Performing addition step for chemical Chemical TEOS: 200.0 uL (TEOS_Container -> Waste_ContainerValve2)

2024-09-27 12:47:27,490<22280>:SwitchingValveVici@COM14:INFO - Valve SwitchingValveVici@COM14 is currently in position 6 (connected to Waste_ContainerValve2).

2024-09-27 12:47:27,809<22280>:SwitchingValveVici@COM14:INFO - Moved valve SwitchingValveVici@COM14 to position 9 (connected to TEOS_Container).

2024-09-27 12:47:27,894<17088>:Aladdin@COM9:INFO - Infusion/withdrawing volume set to 200.0 uL at a rate of 19.08 mL/min

2024-09-27 12:47:28,677<17088>:Aladdin@COM9:INFO - Finished withdrawing 200.0 uL at a rate of 19.08 mL/min

2024-09-27 12:47:28,704<22280>:SwitchingValveVici@COM14:INFO - Valve SwitchingValveVici@COM14 is currently in position 9 (connected to TEOS_Container).

2024-09-27 12:47:29,023<22280>:SwitchingValveVici@COM14:INFO - Moved valve SwitchingValveVici@COM14 to position 6 (connected to Waste_ContainerValve2).

2024-09-27 12:47:29,109<17088>:Aladdin@COM9:INFO - Infusion/withdrawing volume set to 200.0 uL at a rate of 19.08 mL/min

2024-09-27 12:47:29,876<17088>:Aladdin@COM9:INFO - Finished infusing 200.0 uL at a rate of 19.08 mL/min

2024-09-27 12:47:29,876<22280>:SwitchingValveVici@COM14:INFO - Finished adding Chemical TEOS: 200.0 uL (TEOS_Container -> Waste_ContainerValve2)

2024-09-27 12:47:30,881<22280>:SwitchingValveVici@COM14:INFO - Performing purging step into container Container[Waste_ContainerValve2]: 11.8 mL at SwitchingValveVici@COM14->slot 6...

2024-09-27 12:47:30,909<22280>:SwitchingValveVici@COM14:INFO - Valve SwitchingValveVici@COM14 is currently in position 6 (connected to Waste_ContainerValve2).

2024-09-27 12:47:31,053<22280>:SwitchingValveVici@COM14:INFO - Moved valve SwitchingValveVici@COM14 to position 5 (connected to None).

2024-09-27 12:47:31,122<17088>:Aladdin@COM9:INFO - Infusion/withdrawing volume set to 4.0 mL at a rate of 19.08 mL/min

2024-09-27 12:47:43,890<17088>:Aladdin@COM9:INFO - Finished withdrawing 4.0 mL at a rate of 19.08 mL/min

2024-09-27 12:47:43,929<22280>:SwitchingValveVici@COM14:INFO - Valve SwitchingValveVici@COM14 is currently in position 5 (connected to None).

2024-09-27 12:47:44,075<22280>:SwitchingValveVici@COM14:INFO - Moved valve SwitchingValveVici@COM14 to position 6 (connected to Waste_ContainerValve2).

2024-09-27 12:47:44,146<17088>:Aladdin@COM9:INFO - Infusion/withdrawing volume set to 4.0 mL at a rate of 19.08 mL/min

2024-09-27 12:47:56,913<17088>:Aladdin@COM9:INFO - Finished infusing 4.0 mL at a rate of 19.08 mL/min

2024-09-27 12:47:56,914<22280>:SwitchingValveVici@COM14:INFO - Performing addition step for chemical Chemical TEOS: 1.0 mL (TEOS_Container -> flask3)

2024-09-27 12:47:56,950<22280>:SwitchingValveVici@COM14:INFO - Valve SwitchingValveVici@COM14 is currently in position 6 (connected to Waste_ContainerValve2).

2024-09-27 12:47:57,270<22280>:SwitchingValveVici@COM14:INFO - Moved valve SwitchingValveVici@COM14 to position 9 (connected to TEOS_Container).

2024-09-27 12:47:57,345<17088>:Aladdin@COM9:INFO - Infusion/withdrawing volume set to 1.0 mL at a rate of 5.0 mL/min

2024-09-27 12:48:09,602<17088>:Aladdin@COM9:INFO - Finished withdrawing 1.0 mL at a rate of 5.0 mL/min

2024-09-27 12:48:09,636<22280>:SwitchingValveVici@COM14:INFO - Valve SwitchingValveVici@COM14 is currently in position 9 (connected to TEOS_Container).

2024-09-27 12:48:10,051<22280>:SwitchingValveVici@COM14:INFO - Moved valve SwitchingValveVici@COM14 to position 3 (connected to RCTDigital5@COM18).

2024-09-27 12:48:10,129<17088>:Aladdin@COM9:INFO - Infusion/withdrawing volume set to 1.0 mL at a rate of 19.08 mL/min

2024-09-27 12:48:13,437<17088>:Aladdin@COM9:INFO - Finished infusing 1.0 mL at a rate of 19.08 mL/min

2024-09-27 12:48:13,437<22280>:SwitchingValveVici@COM14:INFO - Finished adding Chemical TEOS: 1.0 mL (TEOS_Container -> flask3)

2024-09-27 12:48:14,452<22280>:SwitchingValveVici@COM14:INFO - Performing purging step into container FLASK_100_ML[flask3]: 61.72 mL at SwitchingValveVici@COM14->slot 1...

2024-09-27 12:48:14,492<22280>:SwitchingValveVici@COM14:INFO - Valve SwitchingValveVici@COM14 is currently in position 3 (connected to RCTDigital5@COM18).

2024-09-27 12:48:14,733<22280>:SwitchingValveVici@COM14:INFO - Moved valve SwitchingValveVici@COM14 to position 5 (connected to None).

2024-09-27 12:48:14,827<17088>:Aladdin@COM9:INFO - Infusion/withdrawing volume set to 0.87 mL at a rate of 19.08 mL/min

2024-09-27 12:48:17,912<17088>:Aladdin@COM9:INFO - Finished withdrawing 0.87 mL at a rate of 19.08 mL/min

2024-09-27 12:48:17,945<22280>:SwitchingValveVici@COM14:INFO - Valve SwitchingValveVici@COM14 is currently in position 5 (connected to None).

2024-09-27 12:48:18,184<22280>:SwitchingValveVici@COM14:INFO - Moved valve SwitchingValveVici@COM14 to position 3 (connected to RCTDigital5@COM18).

2024-09-27 12:48:18,279<17088>:Aladdin@COM9:INFO - Infusion/withdrawing volume set to 0.87 mL at a rate of 19.08 mL/min

2024-09-27 12:48:21,363<17088>:Aladdin@COM9:INFO - Finished infusing 0.87 mL at a rate of 19.08 mL/min

2024-09-27 12:48:21,364<22280>:SwitchingValveVici@COM14:INFO - Performing purging step into container FLASK_100_ML[flask3]: 61.72 mL at SwitchingValveVici@COM14->slot 1...

2024-09-27 12:48:21,396<22280>:SwitchingValveVici@COM14:INFO - Valve SwitchingValveVici@COM14 is currently in position 3 (connected to RCTDigital5@COM18).

2024-09-27 12:48:21,635<22280>:SwitchingValveVici@COM14:INFO - Moved valve SwitchingValveVici@COM14 to position 5 (connected to None).

2024-09-27 12:48:21,714<17088>:Aladdin@COM9:INFO - Infusion/withdrawing volume set to 1.0 mL at a rate of 19.08 mL/min

2024-09-27 12:48:25,022<17088>:Aladdin@COM9:INFO - Finished withdrawing 1.0 mL at a rate of 19.08 mL/min

2024-09-27 12:48:25,054<22280>:SwitchingValveVici@COM14:INFO - Valve SwitchingValveVici@COM14 is currently in position 5 (connected to None).

2024-09-27 12:48:25,293<22280>:SwitchingValveVici@COM14:INFO - Moved valve SwitchingValveVici@COM14 to position 3 (connected to RCTDigital5@COM18).

2024-09-27 12:48:25,358<17088>:Aladdin@COM9:INFO - Infusion/withdrawing volume set to 1.0 mL at a rate of 2.0 mL/min

2024-09-27 12:48:55,575<17088>:Aladdin@COM9:INFO - Finished infusing 1.0 mL at a rate of 2.0 mL/min

2024-09-27 12:48:55,575<22280>:SwitchingValveVici@COM14:INFO - Performing purging step into container FLASK_100_ML[flask3]: 61.72 mL at SwitchingValveVici@COM14->slot 1...

2024-09-27 12:48:55,604<22280>:SwitchingValveVici@COM14:INFO - Valve SwitchingValveVici@COM14 is currently in position 3 (connected to RCTDigital5@COM18).

2024-09-27 12:48:55,842<22280>:SwitchingValveVici@COM14:INFO - Moved valve SwitchingValveVici@COM14 to position 5 (connected to None).

2024-09-27 12:48:55,910<17088>:Aladdin@COM9:INFO - Infusion/withdrawing volume set to 4.0 mL at a rate of 19.08 mL/min

2024-09-27 12:49:08,695<17088>:Aladdin@COM9:INFO - Finished withdrawing 4.0 mL at a rate of 19.08 mL/min

2024-09-27 12:49:08,734<22280>:SwitchingValveVici@COM14:INFO - Valve SwitchingValveVici@COM14 is currently in position 5 (connected to None).

2024-09-27 12:49:08,973<22280>:SwitchingValveVici@COM14:INFO - Moved valve SwitchingValveVici@COM14 to position 3 (connected to RCTDigital5@COM18).

2024-09-27 12:49:09,047<17088>:Aladdin@COM9:INFO - Infusion/withdrawing volume set to 4.0 mL at a rate of 10.0 mL/min

2024-09-27 12:49:33,286<17088>:Aladdin@COM9:INFO - Finished infusing 4.0 mL at a rate of 10.0 mL/min

2024-09-27 12:49:33,287<22280>:Minerva.API.MinervaAPI:SYNTHESIS_STEP - Add Chemical [flask3]: FLASK_100_ML; Chemical TEOS: 1.0 mL; 2.0 mL/min; 5 mL Syringe

2024-09-27 12:49:33,324<22280>:SwitchingValveVici@COM14:INFO - Valve SwitchingValveVici@COM14 is currently in position 3 (connected to RCTDigital5@COM18).

2024-09-27 12:49:33,324<22280>:SwitchingValveVici@COM14:INFO - Moved valve SwitchingValveVici@COM14 to position 3 (connected to RCTDigital5@COM18).

2024-09-27 12:49:33,398<17088>:Aladdin@COM9:INFO - Infusion/withdrawing volume set to 4.0 mL at a rate of 19.08 mL/min

2024-09-27 12:49:46,200<17088>:Aladdin@COM9:INFO - Finished withdrawing 4.0 mL at a rate of 19.08 mL/min

2024-09-27 12:49:46,233<22280>:SwitchingValveVici@COM14:INFO - Valve SwitchingValveVici@COM14 is currently in position 3 (connected to RCTDigital5@COM18).

2024-09-27 12:49:46,553<22280>:SwitchingValveVici@COM14:INFO - Moved valve SwitchingValveVici@COM14 to position 6 (connected to Waste_ContainerValve2).

2024-09-27 12:49:46,630<17088>:Aladdin@COM9:INFO - Infusion/withdrawing volume set to 4.0 mL at a rate of 19.08 mL/min

2024-09-27 12:49:59,351<17088>:Aladdin@COM9:INFO - Finished infusing 4.0 mL at a rate of 19.08 mL/min

2024-09-27 12:49:59,352<22280>:SwitchingValveVici@COM14:INFO - Performing addition step for chemical Chemical Water: 1.0 mL (CleaningChemicalValve2 -> Waste_ContainerValve2)

2024-09-27 12:49:59,382<22280>:SwitchingValveVici@COM14:INFO - Valve SwitchingValveVici@COM14 is currently in position 6 (connected to Waste_ContainerValve2).

2024-09-27 12:49:59,621<22280>:SwitchingValveVici@COM14:INFO - Moved valve SwitchingValveVici@COM14 to position 4 (connected to CleaningChemicalValve2).

2024-09-27 12:49:59,686<17088>:Aladdin@COM9:INFO - Infusion/withdrawing volume set to 1.0 mL at a rate of 19.08 mL/min

2024-09-27 12:50:03,201<17088>:Aladdin@COM9:INFO - Finished withdrawing 1.0 mL at a rate of 19.08 mL/min

2024-09-27 12:50:03,232<22280>:SwitchingValveVici@COM14:INFO - Valve SwitchingValveVici@COM14 is currently in position 4 (connected to CleaningChemicalValve2).

2024-09-27 12:50:03,471<22280>:SwitchingValveVici@COM14:INFO - Moved valve SwitchingValveVici@COM14 to position 6 (connected to Waste_ContainerValve2).

2024-09-27 12:50:03,537<17088>:Aladdin@COM9:INFO - Infusion/withdrawing volume set to 1.0 mL at a rate of 19.08 mL/min

2024-09-27 12:50:06,829<17088>:Aladdin@COM9:INFO - Finished infusing 1.0 mL at a rate of 19.08 mL/min

2024-09-27 12:50:06,830<22280>:SwitchingValveVici@COM14:INFO - Finished adding Chemical Water: 1.0 mL (CleaningChemicalValve2 -> Waste_ContainerValve2)

2024-09-27 12:50:07,836<22280>:SwitchingValveVici@COM14:INFO - Performing purging step into container Container[Waste_ContainerValve2]: 16.8 mL at SwitchingValveVici@COM14->slot 6...

2024-09-27 12:50:07,866<22280>:SwitchingValveVici@COM14:INFO - Valve SwitchingValveVici@COM14 is currently in position 6 (connected to Waste_ContainerValve2).

2024-09-27 12:50:08,024<22280>:SwitchingValveVici@COM14:INFO - Moved valve SwitchingValveVici@COM14 to position 5 (connected to None).

2024-09-27 12:50:08,108<17088>:Aladdin@COM9:INFO - Infusion/withdrawing volume set to 0.87 mL at a rate of 19.08 mL/min

2024-09-27 12:50:11,192<17088>:Aladdin@COM9:INFO - Finished withdrawing 0.87 mL at a rate of 19.08 mL/min

2024-09-27 12:50:11,222<22280>:SwitchingValveVici@COM14:INFO - Valve SwitchingValveVici@COM14 is currently in position 5 (connected to None).

2024-09-27 12:50:11,366<22280>:SwitchingValveVici@COM14:INFO - Moved valve SwitchingValveVici@COM14 to position 6 (connected to Waste_ContainerValve2).

2024-09-27 12:50:11,447<17088>:Aladdin@COM9:INFO - Infusion/withdrawing volume set to 0.87 mL at a rate of 19.08 mL/min

2024-09-27 12:50:14,531<17088>:Aladdin@COM9:INFO - Finished infusing 0.87 mL at a rate of 19.08 mL/min

2024-09-27 12:50:14,532<22280>:SwitchingValveVici@COM14:INFO - Performing purging step into container Container[Waste_ContainerValve2]: 16.8 mL at SwitchingValveVici@COM14->slot 6...

2024-09-27 12:50:14,561<22280>:SwitchingValveVici@COM14:INFO - Valve SwitchingValveVici@COM14 is currently in position 6 (connected to Waste_ContainerValve2).

2024-09-27 12:50:14,719<22280>:SwitchingValveVici@COM14:INFO - Moved valve SwitchingValveVici@COM14 to position 5 (connected to None).

2024-09-27 12:50:14,787<17088>:Aladdin@COM9:INFO - Infusion/withdrawing volume set to 1.0 mL at a rate of 19.08 mL/min

2024-09-27 12:50:18,095<17088>:Aladdin@COM9:INFO - Finished withdrawing 1.0 mL at a rate of 19.08 mL/min

2024-09-27 12:50:18,123<22280>:SwitchingValveVici@COM14:INFO - Valve SwitchingValveVici@COM14 is currently in position 5 (connected to None).

2024-09-27 12:50:18,282<22280>:SwitchingValveVici@COM14:INFO - Moved valve SwitchingValveVici@COM14 to position 6 (connected to Waste_ContainerValve2).

2024-09-27 12:50:18,350<17088>:Aladdin@COM9:INFO - Infusion/withdrawing volume set to 1.0 mL at a rate of 19.08 mL/min

2024-09-27 12:50:21,658<17088>:Aladdin@COM9:INFO - Finished infusing 1.0 mL at a rate of 19.08 mL/min

2024-09-27 12:50:21,659<22280>:SwitchingValveVici@COM14:INFO - Performing purging step into container Container[Waste_ContainerValve2]: 16.8 mL at SwitchingValveVici@COM14->slot 6...

2024-09-27 12:50:21,686<22280>:SwitchingValveVici@COM14:INFO - Valve SwitchingValveVici@COM14 is currently in position 6 (connected to Waste_ContainerValve2).

2024-09-27 12:50:21,830<22280>:SwitchingValveVici@COM14:INFO - Moved valve SwitchingValveVici@COM14 to position 5 (connected to None).

2024-09-27 12:50:21,898<17088>:Aladdin@COM9:INFO - Infusion/withdrawing volume set to 4.0 mL at a rate of 19.08 mL/min

2024-09-27 12:50:34,682<17088>:Aladdin@COM9:INFO - Finished withdrawing 4.0 mL at a rate of 19.08 mL/min

2024-09-27 12:50:34,708<22280>:SwitchingValveVici@COM14:INFO - Valve SwitchingValveVici@COM14 is currently in position 5 (connected to None).

2024-09-27 12:50:34,852<22280>:SwitchingValveVici@COM14:INFO - Moved valve SwitchingValveVici@COM14 to position 6 (connected to Waste_ContainerValve2).

2024-09-27 12:50:34,921<17088>:Aladdin@COM9:INFO - Infusion/withdrawing volume set to 4.0 mL at a rate of 19.08 mL/min

2024-09-27 12:50:47,673<17088>:Aladdin@COM9:INFO - Finished infusing 4.0 mL at a rate of 19.08 mL/min

2024-09-27 13:45:06,786<20892>:DHT22Sensor@ArduinoController@COM25:INFO - Temperature: 22.00 C, Humidity: 26.00 %

2024-09-27 14:30:23,472<23056>:RCTDigital5@COM6:INFO - Stopped heating.

2024-09-27 14:30:23,472<23056>:RCTDigital5@COM6:INFO - Temperature setpoint changed to: 0 degrees Celsius

2024-09-27 14:30:23,495<20804>:HotplateFan@RCTDigital5@COM6@ArduinoController@COM25:INFO - Fan1 turned on.

2024-09-27 14:30:23,527<23056>:RCTDigital5@COM6:INFO - Waiting for hotplate to cool down: 94.0 C / 40 C

2024-09-27 14:30:53,569<23056>:RCTDigital5@COM6:INFO - Waiting for hotplate to cool down: 90.7 C / 40 C

2024-09-27 14:31:23,610<23056>:RCTDigital5@COM6:INFO - Waiting for hotplate to cool down: 86.5 C / 40 C

2024-09-27 14:31:53,636<23056>:RCTDigital5@COM6:INFO - Waiting for hotplate to cool down: 82.8 C / 40 C

2024-09-27 14:32:23,678<23056>:RCTDigital5@COM6:INFO - Waiting for hotplate to cool down: 79.3 C / 40 C

2024-09-27 14:32:53,702<23056>:RCTDigital5@COM6:INFO - Waiting for hotplate to cool down: 76.0 C / 40 C

2024-09-27 14:33:23,742<23056>:RCTDigital5@COM6:INFO - Waiting for hotplate to cool down: 73.0 C / 40 C

2024-09-27 14:33:53,784<23056>:RCTDigital5@COM6:INFO - Waiting for hotplate to cool down: 70.2 C / 40 C

2024-09-27 14:34:23,811<23056>:RCTDigital5@COM6:INFO - Waiting for hotplate to cool down: 67.5 C / 40 C

2024-09-27 14:34:53,853<23056>:RCTDigital5@COM6:INFO - Waiting for hotplate to cool down: 65.0 C / 40 C

2024-09-27 14:35:23,894<23056>:RCTDigital5@COM6:INFO - Waiting for hotplate to cool down: 62.7 C / 40 C

2024-09-27 14:35:53,935<23056>:RCTDigital5@COM6:INFO - Waiting for hotplate to cool down: 60.6 C / 40 C

2024-09-27 14:36:23,976<23056>:RCTDigital5@COM6:INFO - Waiting for hotplate to cool down: 58.6 C / 40 C

2024-09-27 14:36:54,017<23056>:RCTDigital5@COM6:INFO - Waiting for hotplate to cool down: 56.7 C / 40 C

2024-09-27 14:37:24,058<23056>:RCTDigital5@COM6:INFO - Waiting for hotplate to cool down: 54.9 C / 40 C

2024-09-27 14:37:54,098<23056>:RCTDigital5@COM6:INFO - Waiting for hotplate to cool down: 53.3 C / 40 C

2024-09-27 14:38:24,140<23056>:RCTDigital5@COM6:INFO - Waiting for hotplate to cool down: 51.7 C / 40 C

2024-09-27 14:38:54,181<23056>:RCTDigital5@COM6:INFO - Waiting for hotplate to cool down: 50.3 C / 40 C

2024-09-27 14:38:55,649<20104>:RCTDigital5@COM4:INFO - Stopped heating.

2024-09-27 14:38:55,649<20104>:RCTDigital5@COM4:INFO - Temperature setpoint changed to: 0 degrees Celsius

2024-09-27 14:38:55,672<10908>:HotplateFan@RCTDigital5@COM4@ArduinoController@COM25:INFO - Fan2 turned on.

2024-09-27 14:38:55,702<20104>:RCTDigital5@COM4:INFO - Waiting for hotplate to cool down: 93.6 C / 40 C

2024-09-27 14:39:24,206<23056>:RCTDigital5@COM6:INFO - Waiting for hotplate to cool down: 48.9 C / 40 C

2024-09-27 14:39:25,728<20104>:RCTDigital5@COM4:INFO - Waiting for hotplate to cool down: 90.7 C / 40 C

2024-09-27 14:39:54,247<23056>:RCTDigital5@COM6:INFO - Waiting for hotplate to cool down: 47.6 C / 40 C

2024-09-27 14:39:55,767<20104>:RCTDigital5@COM4:INFO - Waiting for hotplate to cool down: 86.5 C / 40 C

2024-09-27 14:40:24,287<23056>:RCTDigital5@COM6:INFO - Waiting for hotplate to cool down: 46.4 C / 40 C

2024-09-27 14:40:25,806<20104>:RCTDigital5@COM4:INFO - Waiting for hotplate to cool down: 82.5 C / 40 C

2024-09-27 14:40:54,330<23056>:RCTDigital5@COM6:INFO - Waiting for hotplate to cool down: 45.3 C / 40 C

2024-09-27 14:40:55,845<20104>:RCTDigital5@COM4:INFO - Waiting for hotplate to cool down: 78.9 C / 40 C

2024-09-27 14:41:24,371<23056>:RCTDigital5@COM6:INFO - Waiting for hotplate to cool down: 44.3 C / 40 C

2024-09-27 14:41:25,885<20104>:RCTDigital5@COM4:INFO - Waiting for hotplate to cool down: 75.5 C / 40 C

2024-09-27 14:41:54,413<23056>:RCTDigital5@COM6:INFO - Waiting for hotplate to cool down: 43.2 C / 40 C

2024-09-27 14:41:55,926<20104>:RCTDigital5@COM4:INFO - Waiting for hotplate to cool down: 72.4 C / 40 C

2024-09-27 14:42:24,454<23056>:RCTDigital5@COM6:INFO - Waiting for hotplate to cool down: 42.3 C / 40 C

2024-09-27 14:42:25,965<20104>:RCTDigital5@COM4:INFO - Waiting for hotplate to cool down: 69.5 C / 40 C

2024-09-27 14:42:54,498<23056>:RCTDigital5@COM6:INFO - Waiting for hotplate to cool down: 41.4 C / 40 C

2024-09-27 14:42:56,005<20104>:RCTDigital5@COM4:INFO - Waiting for hotplate to cool down: 66.8 C / 40 C

2024-09-27 14:43:24,538<23056>:RCTDigital5@COM6:INFO - Waiting for hotplate to cool down: 40.6 C / 40 C

2024-09-27 14:43:26,045<20104>:RCTDigital5@COM4:INFO - Waiting for hotplate to cool down: 64.2 C / 40 C

2024-09-27 14:43:54,580<23056>:RCTDigital5@COM6:INFO - Waiting for hotplate to cool down: 39.8 C / 40 C, below setpoint since 30 seconds.

2024-09-27 14:43:56,070<20104>:RCTDigital5@COM4:INFO - Waiting for hotplate to cool down: 61.9 C / 40 C

2024-09-27 14:44:24,589<23056>:RCTDigital5@COM6:INFO - Temperature is now below 40 degrees Celsius.

2024-09-27 14:44:24,589<23056>:RCTDigital5@COM6:INFO - Stopped stirring.

2024-09-27 14:44:24,590<23056>:RCTDigital5@COM6:INFO - Stirring Speed setpoint changed to: 0 rpm

2024-09-27 14:44:24,590<20804>:Minerva.API.MinervaAPI:SYNTHESIS_STEP - Cool down [flask1]: 40 degrees Celsius; Active Cooling True

2024-09-27 14:44:24,618<20804>:HotplateFan@RCTDigital5@COM6@ArduinoController@COM25:INFO - Fan1 turned off.

2024-09-27 14:44:24,639<20804>:HotplateFan@RCTDigital5@COM6@ArduinoController@COM25:INFO - Fan1 turned off.

2024-09-27 14:44:26,111<20104>:RCTDigital5@COM4:INFO - Waiting for hotplate to cool down: 59.7 C / 40 C

2024-09-27 14:44:44,998<20804>:HotplateClampDCMotor@RCTDigital5@COM6@ArduinoController@COM25:INFO - Clamp1 moved up.

2024-09-27 14:44:49,031<20804>:HotplateClampDCMotor@RCTDigital5@COM6@ArduinoController@COM25:INFO - Clamp1 opened.

2024-09-27 14:44:56,148<20104>:RCTDigital5@COM4:INFO - Waiting for hotplate to cool down: 57.8 C / 40 C

2024-09-27 14:45:01,530<10064>:XArm6@192.168.1.204:INFO - Finished moving robot arm to source destination: Ika_100mL_Heating_Block at RCTDigital5@COM6->deck 1.

2024-09-27 14:45:07,025<20892>:DHT22Sensor@ArduinoController@COM25:INFO - Temperature: 22.00 C, Humidity: 26.00 %

2024-09-27 14:45:21,071<10064>:XArm6@192.168.1.204:INFO - Finished moving robot arm to target destination: SwitchingValveVici@COM3.

2024-09-27 15:13:00,802<13700>:RobotCen@COM8:INFO - Opened RobotCen Lid.

2024-09-27 15:13:15,741<20644>:XArm6@192.168.1.204:INFO - Finished moving robot arm to source destination: Isolab_50mL_Foldable_Tube_Rack->deck 1.

2024-09-27 15:13:29,614<20644>:XArm6@192.168.1.204:INFO - Finished moving robot arm to target destination: RobotCen@COM8.

2024-09-27 15:13:40,594<13700>:RobotCen@COM8:INFO - Rotor moved to slot 5.

2024-09-27 15:13:40,640<13700>:RobotCen@COM8:INFO - Opened RobotCen Lid.

2024-09-27 15:13:56,203<20644>:XArm6@192.168.1.204:INFO - Finished moving robot arm to source destination: Isolab_50mL_Foldable_Tube_Rack->deck 1.

2024-09-27 15:14:10,546<20644>:XArm6@192.168.1.204:INFO - Finished moving robot arm to target destination: RobotCen@COM8.

2024-09-27 15:14:19,938<13700>:RobotCen@COM8:INFO - Rotor moved to slot 2.

2024-09-27 15:14:19,984<13700>:RobotCen@COM8:INFO - Opened RobotCen Lid.

2024-09-27 15:14:35,693<20644>:XArm6@192.168.1.204:INFO - Finished moving robot arm to source destination: Isolab_50mL_Foldable_Tube_Rack->deck 1.

2024-09-27 15:14:51,905<20644>:XArm6@192.168.1.204:INFO - Finished moving robot arm to target destination: RobotCen@COM8.

2024-09-27 15:15:00,622<13700>:RobotCen@COM8:INFO - Rotor moved to slot 6.

2024-09-27 15:15:00,669<13700>:RobotCen@COM8:INFO - Opened RobotCen Lid.

2024-09-27 15:15:16,746<20644>:XArm6@192.168.1.204:INFO - Finished moving robot arm to source destination: Isolab_50mL_Foldable_Tube_Rack->deck 1.

2024-09-27 15:15:31,163<20644>:XArm6@192.168.1.204:INFO - Finished moving robot arm to target destination: RobotCen@COM8.

2024-09-27 15:15:38,992<13700>:RobotCen@COM8:INFO - Rotor moved to slot 4.

2024-09-27 15:15:39,038<13700>:RobotCen@COM8:INFO - Opened RobotCen Lid.

2024-09-27 15:15:54,084<20644>:XArm6@192.168.1.204:INFO - Finished moving robot arm to source destination: Isolab_50mL_Foldable_Tube_Rack->deck 1.

2024-09-27 15:16:07,674<20644>:XArm6@192.168.1.204:INFO - Finished moving robot arm to target destination: RobotCen@COM8.

2024-09-27 15:16:18,686<13700>:RobotCen@COM8:INFO - Rotor moved to slot 8.

2024-09-27 15:16:18,733<13700>:RobotCen@COM8:INFO - Opened RobotCen Lid.

2024-09-27 15:16:33,801<20644>:XArm6@192.168.1.204:INFO - Finished moving robot arm to source destination: Isolab_50mL_Foldable_Tube_Rack->deck 1.

2024-09-27 15:16:48,289<20644>:XArm6@192.168.1.204:INFO - Finished moving robot arm to target destination: RobotCen@COM8.

2024-09-27 15:16:51,159<3028>:RobotCen@COM8:INFO - Time set to 1200 seconds

2024-09-27 15:16:51,207<3028>:RobotCen@COM8:INFO - Speed set to 11731 rpm

2024-09-27 15:16:51,255<3028>:RobotCen@COM8:INFO - Temperature set to 25 degrees Celsius

2024-09-27 15:16:54,242<3028>:RobotCen@COM8:INFO - Closed RobotCen Lid.

2024-09-27 15:16:54,274<3028>:RobotCen@COM8:INFO - Centrifugation run started. Time: 1200 seconds; Speed: 11731 rpm; Temperature: 25 degrees Celsius

2024-09-27 15:17:48,232<3028>:RobotCen@COM8:INFO - Centrifugation speed setpoint reached.

2024-09-27 15:39:08,550<3028>:RobotCen@COM8:INFO - Centrifugation run finished.

2024-09-27 15:39:10,556<3028>:RobotCen@COM8:INFO - Homing to first rotor position...

2024-09-27 15:39:41,331<3028>:RobotCen@COM8:INFO - Rotor set to first position 1100

2024-09-27 15:39:41,331<3028>:RobotCen@COM8:INFO - Setting next rotor position...

2024-09-27 15:39:45,691<3028>:RobotCen@COM8:INFO - Bottle number set to 8.

2024-09-27 15:39:52,259<13700>:RobotCen@COM8:INFO - Rotor moved to slot 1.

2024-09-27 15:39:55,259<13700>:RobotCen@COM8:INFO - Opened RobotCen Lid.

2024-09-27 15:40:04,055<20644>:XArm6@192.168.1.204:INFO - Finished moving robot arm to source destination: RobotCen@COM8.

2024-09-27 15:40:20,536<20644>:XArm6@192.168.1.204:INFO - Finished moving robot arm to target destination: Isolab_50mL_Foldable_Tube_Rack->deck 1.

2024-09-27 15:40:29,253<13700>:RobotCen@COM8:INFO - Rotor moved to slot 5.

2024-09-27 15:40:29,300<13700>:RobotCen@COM8:INFO - Opened RobotCen Lid.

2024-09-27 15:40:42,847<20644>:XArm6@192.168.1.204:INFO - Finished moving robot arm to source destination: RobotCen@COM8.

2024-09-27 15:40:59,502<20644>:XArm6@192.168.1.204:INFO - Finished moving robot arm to target destination: Isolab_50mL_Foldable_Tube_Rack->deck 1.

2024-09-27 15:41:06,630<13700>:RobotCen@COM8:INFO - Rotor moved to slot 2.

2024-09-27 15:41:06,677<13700>:RobotCen@COM8:INFO - Opened RobotCen Lid.

2024-09-27 15:41:20,795<20644>:XArm6@192.168.1.204:INFO - Finished moving robot arm to source destination: RobotCen@COM8.

2024-09-27 15:41:37,179<20644>:XArm6@192.168.1.204:INFO - Finished moving robot arm to target destination: Isolab_50mL_Foldable_Tube_Rack->deck 1.

2024-09-27 15:41:45,894<13700>:RobotCen@COM8:INFO - Rotor moved to slot 6.

2024-09-27 15:41:45,942<13700>:RobotCen@COM8:INFO - Opened RobotCen Lid.

2024-09-27 15:42:00,285<20644>:XArm6@192.168.1.204:INFO - Finished moving robot arm to source destination: RobotCen@COM8.

2024-09-27 15:42:16,444<20644>:XArm6@192.168.1.204:INFO - Finished moving robot arm to target destination: Isolab_50mL_Foldable_Tube_Rack->deck 1.

2024-09-27 15:42:21,962<13700>:RobotCen@COM8:INFO - Rotor moved to slot 4.

2024-09-27 15:42:22,009<13700>:RobotCen@COM8:INFO - Opened RobotCen Lid.

2024-09-27 15:42:36,188<20644>:XArm6@192.168.1.204:INFO - Finished moving robot arm to source destination: RobotCen@COM8.

2024-09-27 15:42:53,211<20644>:XArm6@192.168.1.204:INFO - Finished moving robot arm to target destination: Isolab_50mL_Foldable_Tube_Rack->deck 1.

2024-09-27 15:43:01,928<13700>:RobotCen@COM8:INFO - Rotor moved to slot 8.

2024-09-27 15:43:01,976<13700>:RobotCen@COM8:INFO - Opened RobotCen Lid.

2024-09-27 15:43:16,329<20644>:XArm6@192.168.1.204:INFO - Finished moving robot arm to source destination: RobotCen@COM8.

2024-09-27 15:43:33,667<20644>:XArm6@192.168.1.204:INFO - Finished moving robot arm to target destination: Isolab_50mL_Foldable_Tube_Rack->deck 1.

2024-09-27 15:43:34,210<13700>:Minerva.API.MinervaAPI:SYNTHESIS_STEP - Centrifuge [FALCON_TUBE_50_ML_2363304638688]: 1200.0 seconds; 11731.38283132503 rpm; 25.0 degrees Celsius

2024-09-27 15:43:40,666<20644>:XArm6@192.168.1.204:INFO - Finished moving robot arm to source destination: Isolab_50mL_Foldable_Tube_Rack->deck 1.

2024-09-27 15:44:05,419<20644>:XArm6@192.168.1.204:INFO - Changed grip on container FALCON_TUBE_50_ML_2363304638688 to a sideways grip.

2024-09-27 15:44:18,312<20644>:XArm6@192.168.1.204:INFO - Finished moving robot arm to target destination: CapperDecapper@ArduinoController@COM25.

2024-09-27 15:44:18,855<23296>:CapperDecapper@ArduinoController@COM25:INFO - Opening Container...

2024-09-27 15:44:18,951<23296>:CapperDecapper@ArduinoController@COM25:INFO - Clamp opened.

2024-09-27 15:44:23,394<23296>:CapperDecapper@ArduinoController@COM25:INFO - Clamp closed.

2024-09-27 15:44:23,426<23296>:CapperDecapper@ArduinoController@COM25:INFO - Wrist turning counterclockwise

2024-09-27 15:44:25,471<23296>:CapperDecapper@ArduinoController@COM25:INFO - Stopped turning wrist

2024-09-27 15:44:26,829<20644>:XArm6@192.168.1.204:INFO - Finished moving robot arm to source destination: CapperDecapper@ArduinoController@COM25.

2024-09-27 15:44:43,901<20644>:XArm6@192.168.1.204:INFO - Finished moving robot arm to target destination: SwitchingValveVici@COM3.

2024-09-27 15:44:44,476<13700>:SwitchingValveVici@COM3:INFO - Valve SwitchingValveVici@COM3 is currently in position 3 (connected to Waste_Container).

2024-09-27 15:44:44,972<13700>:SwitchingValveVici@COM3:INFO - Moved valve SwitchingValveVici@COM3 to position 8 (connected to Outlet).

2024-09-27 15:44:45,065<22688>:Aladdin@COM12:INFO - Infusion/withdrawing volume set to 30.0 mL at a rate of 70.0 mL/min

2024-09-27 15:45:10,998<22688>:Aladdin@COM12:INFO - Finished withdrawing 30.0 mL at a rate of 70.0 mL/min

2024-09-27 15:45:11,031<13700>:SwitchingValveVici@COM3:INFO - Valve SwitchingValveVici@COM3 is currently in position 8 (connected to Outlet).

2024-09-27 15:45:11,527<13700>:SwitchingValveVici@COM3:INFO - Moved valve SwitchingValveVici@COM3 to position 3 (connected to Waste_Container).

2024-09-27 15:45:11,621<22688>:Aladdin@COM12:INFO - Infusion/withdrawing volume set to 30.0 mL at a rate of 70.0 mL/min

2024-09-27 15:45:37,632<22688>:Aladdin@COM12:INFO - Finished infusing 30.0 mL at a rate of 70.0 mL/min

2024-09-27 15:45:37,633<13700>:SwitchingValveVici@COM3:INFO - Performing purging step into container Container[Waste_Container]: 2.03 L at SwitchingValveVici@COM3->slot 3...

2024-09-27 15:45:37,669<13700>:SwitchingValveVici@COM3:INFO - Valve SwitchingValveVici@COM3 is currently in position 3 (connected to Waste_Container).

2024-09-27 15:45:38,166<13700>:SwitchingValveVici@COM3:INFO - Moved valve SwitchingValveVici@COM3 to position 8 (connected to Outlet).

2024-09-27 15:45:38,257<22688>:Aladdin@COM12:INFO - Infusion/withdrawing volume set to 10.0 mL at a rate of 70.0 mL/min

2024-09-27 15:45:47,155<22688>:Aladdin@COM12:INFO - Finished withdrawing 10.0 mL at a rate of 70.0 mL/min

2024-09-27 15:45:47,192<13700>:SwitchingValveVici@COM3:INFO - Valve SwitchingValveVici@COM3 is currently in position 8 (connected to Outlet).

2024-09-27 15:45:47,689<13700>:SwitchingValveVici@COM3:INFO - Moved valve SwitchingValveVici@COM3 to position 3 (connected to Waste_Container).

2024-09-27 15:45:47,779<22688>:Aladdin@COM12:INFO - Infusion/withdrawing volume set to 10.0 mL at a rate of 70.0 mL/min

2024-09-27 15:45:56,679<22688>:Aladdin@COM12:INFO - Finished infusing 10.0 mL at a rate of 70.0 mL/min

2024-09-27 15:45:56,681<13700>:SwitchingValveVici@COM3:INFO - Performing addition step for chemical Chemical Ethanol: 15.0 mL (Ethanol_wash -> FALCON_TUBE_50_ML_2363304638688)

2024-09-27 15:45:56,715<13700>:SwitchingValveVici@COM3:INFO - Valve SwitchingValveVici@COM3 is currently in position 3 (connected to Waste_Container).

2024-09-27 15:45:56,874<13700>:SwitchingValveVici@COM3:INFO - Moved valve SwitchingValveVici@COM3 to position 2 (connected to Ethanol_wash).

2024-09-27 15:45:56,967<22688>:Aladdin@COM12:INFO - Infusion/withdrawing volume set to 15.0 mL at a rate of 70.0 mL/min

2024-09-27 15:46:10,006<22688>:Aladdin@COM12:INFO - Finished withdrawing 15.0 mL at a rate of 70.0 mL/min

2024-09-27 15:46:10,042<13700>:SwitchingValveVici@COM3:INFO - Valve SwitchingValveVici@COM3 is currently in position 2 (connected to Ethanol_wash).

2024-09-27 15:46:10,457<13700>:SwitchingValveVici@COM3:INFO - Moved valve SwitchingValveVici@COM3 to position 8 (connected to Outlet).

2024-09-27 15:46:10,549<22688>:Aladdin@COM12:INFO - Infusion/withdrawing volume set to 15.0 mL at a rate of 70.0 mL/min

2024-09-27 15:46:23,571<22688>:Aladdin@COM12:INFO - Finished infusing 15.0 mL at a rate of 70.0 mL/min

2024-09-27 15:46:23,572<13700>:SwitchingValveVici@COM3:INFO - Finished adding Chemical Ethanol: 15.0 mL (Ethanol_wash -> FALCON_TUBE_50_ML_2363304638688)

2024-09-27 15:46:24,574<13700>:SwitchingValveVici@COM3:INFO - Performing purging step into container FALCON_TUBE_50_ML: 15.0 mL at SwitchingValveVici@COM3->slot 0...

2024-09-27 15:46:25,781<13700>:SwitchingValveVici@COM3:INFO - Valve SwitchingValveVici@COM3 is currently in position 8 (connected to Outlet).

2024-09-27 15:46:25,782<13700>:SwitchingValveVici@COM3:INFO - Moved valve SwitchingValveVici@COM3 to position 8 (connected to Outlet).

2024-09-27 15:46:25,871<22688>:Aladdin@COM12:INFO - Infusion/withdrawing volume set to 30.0 mL at a rate of 70.0 mL/min

2024-09-27 15:46:51,917<22688>:Aladdin@COM12:INFO - Finished withdrawing 30.0 mL at a rate of 70.0 mL/min

2024-09-27 15:46:53,195<22688>:Aladdin@COM12:INFO - Infusion/withdrawing volume set to 30.0 mL at a rate of 70.0 mL/min

2024-09-27 15:47:19,209<22688>:Aladdin@COM12:INFO - Finished infusing 30.0 mL at a rate of 70.0 mL/min

2024-09-27 15:47:19,210<13700>:Minerva.API.MinervaAPI:SYNTHESIS_STEP - Add Chemical [FALCON_TUBE_50_ML_2363304638688]: FALCON_TUBE_50_ML; Chemical Ethanol: 15.0 mL; 70.0 mL/min; 50 mL Syringe

2024-09-27 15:47:20,755<20644>:XArm6@192.168.1.204:INFO - Finished moving robot arm to source destination: SwitchingValveVici@COM3.

2024-09-27 15:47:29,420<20644>:XArm6@192.168.1.204:INFO - Finished moving robot arm to target destination: UP200ST@192.168.233.233.

2024-09-27 15:47:30,017<14772>:UP200ST@192.168.233.233:INFO - Amplitude changed to 50.0 %.

2024-09-27 15:47:30,070<14772>:UP200ST@192.168.233.233:INFO - Power changed to 50.0 %.

2024-09-27 15:47:30,115<14772>:UP200ST@192.168.233.233:INFO - Time limit activated.

2024-09-27 15:47:30,161<14772>:UP200ST@192.168.233.233:INFO - Time limit changed to 90.0 s.

2024-09-27 15:47:30,161<14772>:UP200ST@192.168.233.233:INFO - Starting sonication...

2024-09-27 15:47:30,203<14772>:UP200ST@192.168.233.233:INFO - Ultrasound turned on.

2024-09-27 15:49:00,247<14772>:UP200ST@192.168.233.233:INFO - Ultrasound turned off.

2024-09-27 15:49:00,248<14772>:UP200ST@192.168.233.233:INFO - Finished sonication.

2024-09-27 15:49:00,248<13700>:Minerva.API.MinervaAPI:SYNTHESIS_STEP - Sonicate [FALCON_TUBE_50_ML_2363304638688]: 90.0 s; 50 % Amplitude; 50 % Power

2024-09-27 15:49:01,876<20644>:XArm6@192.168.1.204:INFO - Finished moving robot arm to source destination: UP200ST@192.168.233.233.

2024-09-27 15:49:14,368<20644>:XArm6@192.168.1.204:INFO - Finished moving robot arm to target destination: CapperDecapper@ArduinoController@COM25.

2024-09-27 15:49:14,914<23296>:CapperDecapper@ArduinoController@COM25:INFO - Closing Container...

2024-09-27 15:49:14,944<23296>:CapperDecapper@ArduinoController@COM25:INFO - Wrist turning clockwise

2024-09-27 15:49:18,072<23296>:CapperDecapper@ArduinoController@COM25:INFO - Wrist turning clockwise

2024-09-27 15:49:18,666<23296>:CapperDecapper@ArduinoController@COM25:INFO - DC Motor Current [mA]: 273.25

2024-09-27 15:49:18,748<23296>:CapperDecapper@ArduinoController@COM25:INFO - DC Motor Current [mA]: 272.33

2024-09-27 15:49:18,830<23296>:CapperDecapper@ArduinoController@COM25:INFO - DC Motor Current [mA]: 262.85

2024-09-27 15:49:18,912<23296>:CapperDecapper@ArduinoController@COM25:INFO - DC Motor Current [mA]: 264.02

2024-09-27 15:49:18,994<23296>:CapperDecapper@ArduinoController@COM25:INFO - DC Motor Current [mA]: 268.90

2024-09-27 15:49:19,076<23296>:CapperDecapper@ArduinoController@COM25:INFO - DC Motor Current [mA]: 276.95

2024-09-27 15:49:19,158<23296>:CapperDecapper@ArduinoController@COM25:INFO - DC Motor Current [mA]: 284.82

2024-09-27 15:49:19,239<23296>:CapperDecapper@ArduinoController@COM25:INFO - DC Motor Current [mA]: 301.83

2024-09-27 15:49:19,272<23296>:CapperDecapper@ArduinoController@COM25:INFO - Stopped turning wrist

2024-09-27 15:49:21,258<23296>:CapperDecapper@ArduinoController@COM25:INFO - Clamp opened.

2024-09-27 15:49:22,679<20644>:XArm6@192.168.1.204:INFO - Finished moving robot arm to source destination: CapperDecapper@ArduinoController@COM25.

2024-09-27 15:49:51,678<20644>:XArm6@192.168.1.204:INFO - Changed grip on container FALCON_TUBE_50_ML_2363304638688 to a top grip.

2024-09-27 15:50:06,705<20644>:XArm6@192.168.1.204:INFO - Finished moving robot arm to target destination: Isolab_50mL_Foldable_Tube_Rack->deck 1.

2024-09-27 15:50:07,250<13700>:Minerva.API.MinervaAPI:SYNTHESIS_STEP - Remove supernatant and redisperse [FALCON_TUBE_50_ML_2363304638688]: Chemical Ethanol: 15.0 mL

2024-09-27 15:50:15,629<20644>:XArm6@192.168.1.204:INFO - Finished moving robot arm to source destination: Isolab_50mL_Foldable_Tube_Rack->deck 1.

2024-09-27 15:50:40,814<20644>:XArm6@192.168.1.204:INFO - Changed grip on container FALCON_TUBE_50_ML_2363304638976 to a sideways grip.

2024-09-27 15:50:53,609<20644>:XArm6@192.168.1.204:INFO - Finished moving robot arm to target destination: CapperDecapper@ArduinoController@COM25.

2024-09-27 15:50:54,153<23296>:CapperDecapper@ArduinoController@COM25:INFO - Opening Container...

2024-09-27 15:50:54,252<23296>:CapperDecapper@ArduinoController@COM25:INFO - Clamp opened.

2024-09-27 15:50:58,696<23296>:CapperDecapper@ArduinoController@COM25:INFO - Clamp closed.

2024-09-27 15:50:58,729<23296>:CapperDecapper@ArduinoController@COM25:INFO - Wrist turning counterclockwise

2024-09-27 15:51:00,772<23296>:CapperDecapper@ArduinoController@COM25:INFO - Stopped turning wrist

2024-09-27 15:51:02,128<20644>:XArm6@192.168.1.204:INFO - Finished moving robot arm to source destination: CapperDecapper@ArduinoController@COM25.

2024-09-27 15:51:19,186<20644>:XArm6@192.168.1.204:INFO - Finished moving robot arm to target destination: SwitchingValveVici@COM3.

2024-09-27 15:51:19,760<13700>:SwitchingValveVici@COM3:INFO - Valve SwitchingValveVici@COM3 is currently in position 8 (connected to Outlet).

2024-09-27 15:51:19,761<13700>:SwitchingValveVici@COM3:INFO - Moved valve SwitchingValveVici@COM3 to position 8 (connected to Outlet).

2024-09-27 15:51:19,855<22688>:Aladdin@COM12:INFO - Infusion/withdrawing volume set to 30.0 mL at a rate of 70.0 mL/min

2024-09-27 15:51:45,883<22688>:Aladdin@COM12:INFO - Finished withdrawing 30.0 mL at a rate of 70.0 mL/min

2024-09-27 15:51:45,916<13700>:SwitchingValveVici@COM3:INFO - Valve SwitchingValveVici@COM3 is currently in position 8 (connected to Outlet).

2024-09-27 15:51:46,412<13700>:SwitchingValveVici@COM3:INFO - Moved valve SwitchingValveVici@COM3 to position 3 (connected to Waste_Container).

2024-09-27 15:51:46,507<22688>:Aladdin@COM12:INFO - Infusion/withdrawing volume set to 30.0 mL at a rate of 70.0 mL/min

2024-09-27 15:52:12,489<22688>:Aladdin@COM12:INFO - Finished infusing 30.0 mL at a rate of 70.0 mL/min

2024-09-27 15:52:12,489<13700>:SwitchingValveVici@COM3:INFO - Performing purging step into container Container[Waste_Container]: 2.06 L at SwitchingValveVici@COM3->slot 3...

2024-09-27 15:52:12,523<13700>:SwitchingValveVici@COM3:INFO - Valve SwitchingValveVici@COM3 is currently in position 3 (connected to Waste_Container).

2024-09-27 15:52:13,018<13700>:SwitchingValveVici@COM3:INFO - Moved valve SwitchingValveVici@COM3 to position 8 (connected to Outlet).

2024-09-27 15:52:13,110<22688>:Aladdin@COM12:INFO - Infusion/withdrawing volume set to 10.0 mL at a rate of 70.0 mL/min

2024-09-27 15:52:21,963<22688>:Aladdin@COM12:INFO - Finished withdrawing 10.0 mL at a rate of 70.0 mL/min

2024-09-27 15:52:21,996<13700>:SwitchingValveVici@COM3:INFO - Valve SwitchingValveVici@COM3 is currently in position 8 (connected to Outlet).

2024-09-27 15:52:22,491<13700>:SwitchingValveVici@COM3:INFO - Moved valve SwitchingValveVici@COM3 to position 3 (connected to Waste_Container).

2024-09-27 15:52:22,586<22688>:Aladdin@COM12:INFO - Infusion/withdrawing volume set to 10.0 mL at a rate of 70.0 mL/min

2024-09-27 15:52:31,453<22688>:Aladdin@COM12:INFO - Finished infusing 10.0 mL at a rate of 70.0 mL/min

2024-09-27 15:52:31,455<13700>:SwitchingValveVici@COM3:INFO - Performing addition step for chemical Chemical Ethanol: 15.0 mL (Ethanol_wash -> FALCON_TUBE_50_ML_2363304638976)

2024-09-27 15:52:31,487<13700>:SwitchingValveVici@COM3:INFO - Valve SwitchingValveVici@COM3 is currently in position 3 (connected to Waste_Container).

2024-09-27 15:52:31,633<13700>:SwitchingValveVici@COM3:INFO - Moved valve SwitchingValveVici@COM3 to position 2 (connected to Ethanol_wash).

2024-09-27 15:52:31,725<22688>:Aladdin@COM12:INFO - Infusion/withdrawing volume set to 15.0 mL at a rate of 70.0 mL/min

2024-09-27 15:52:44,733<22688>:Aladdin@COM12:INFO - Finished withdrawing 15.0 mL at a rate of 70.0 mL/min

2024-09-27 15:52:44,767<13700>:SwitchingValveVici@COM3:INFO - Valve SwitchingValveVici@COM3 is currently in position 2 (connected to Ethanol_wash).

2024-09-27 15:52:45,181<13700>:SwitchingValveVici@COM3:INFO - Moved valve SwitchingValveVici@COM3 to position 8 (connected to Outlet).

2024-09-27 15:52:45,275<22688>:Aladdin@COM12:INFO - Infusion/withdrawing volume set to 15.0 mL at a rate of 70.0 mL/min

2024-09-27 15:52:58,313<22688>:Aladdin@COM12:INFO - Finished infusing 15.0 mL at a rate of 70.0 mL/min

2024-09-27 15:52:58,314<13700>:SwitchingValveVici@COM3:INFO - Finished adding Chemical Ethanol: 15.0 mL (Ethanol_wash -> FALCON_TUBE_50_ML_2363304638976)

2024-09-27 15:52:59,318<13700>:SwitchingValveVici@COM3:INFO - Performing purging step into container FALCON_TUBE_50_ML: 15.0 mL at SwitchingValveVici@COM3->slot 0...

2024-09-27 15:53:00,522<13700>:SwitchingValveVici@COM3:INFO - Valve SwitchingValveVici@COM3 is currently in position 8 (connected to Outlet).

2024-09-27 15:53:00,523<13700>:SwitchingValveVici@COM3:INFO - Moved valve SwitchingValveVici@COM3 to position 8 (connected to Outlet).

2024-09-27 15:53:00,614<22688>:Aladdin@COM12:INFO - Infusion/withdrawing volume set to 30.0 mL at a rate of 70.0 mL/min

2024-09-27 15:53:26,659<22688>:Aladdin@COM12:INFO - Finished withdrawing 30.0 mL at a rate of 70.0 mL/min

2024-09-27 15:53:27,921<22688>:Aladdin@COM12:INFO - Infusion/withdrawing volume set to 30.0 mL at a rate of 70.0 mL/min

2024-09-27 15:53:53,947<22688>:Aladdin@COM12:INFO - Finished infusing 30.0 mL at a rate of 70.0 mL/min

2024-09-27 15:53:53,948<13700>:Minerva.API.MinervaAPI:SYNTHESIS_STEP - Add Chemical [FALCON_TUBE_50_ML_2363304638976]: FALCON_TUBE_50_ML; Chemical Ethanol: 15.0 mL; 70.0 mL/min; 50 mL Syringe

2024-09-27 15:53:55,487<20644>:XArm6@192.168.1.204:INFO - Finished moving robot arm to source destination: SwitchingValveVici@COM3.

2024-09-27 15:54:04,146<20644>:XArm6@192.168.1.204:INFO - Finished moving robot arm to target destination: UP200ST@192.168.233.233.

2024-09-27 15:54:04,729<14772>:UP200ST@192.168.233.233:INFO - Amplitude changed to 50.0 %.

2024-09-27 15:54:04,757<14772>:UP200ST@192.168.233.233:INFO - Power changed to 50.0 %.

2024-09-27 15:54:04,782<14772>:UP200ST@192.168.233.233:INFO - Time limit activated.

2024-09-27 15:54:04,809<14772>:UP200ST@192.168.233.233:INFO - Time limit changed to 90.0 s.

2024-09-27 15:54:04,809<14772>:UP200ST@192.168.233.233:INFO - Starting sonication...

2024-09-27 15:54:04,835<14772>:UP200ST@192.168.233.233:INFO - Ultrasound turned on.

2024-09-27 15:55:34,878<14772>:UP200ST@192.168.233.233:INFO - Ultrasound turned off.

2024-09-27 15:55:34,879<14772>:UP200ST@192.168.233.233:INFO - Finished sonication.

2024-09-27 15:55:34,879<13700>:Minerva.API.MinervaAPI:SYNTHESIS_STEP - Sonicate [FALCON_TUBE_50_ML_2363304638976]: 90.0 s; 50 % Amplitude; 50 % Power

2024-09-27 15:55:36,474<20644>:XArm6@192.168.1.204:INFO - Finished moving robot arm to source destination: UP200ST@192.168.233.233.

2024-09-27 15:55:48,951<20644>:XArm6@192.168.1.204:INFO - Finished moving robot arm to target destination: CapperDecapper@ArduinoController@COM25.

2024-09-27 15:55:49,495<23296>:CapperDecapper@ArduinoController@COM25:INFO - Closing Container...

2024-09-27 15:55:49,525<23296>:CapperDecapper@ArduinoController@COM25:INFO - Wrist turning clockwise

2024-09-27 15:55:52,653<23296>:CapperDecapper@ArduinoController@COM25:INFO - Wrist turning clockwise

2024-09-27 15:55:53,235<23296>:CapperDecapper@ArduinoController@COM25:INFO - DC Motor Current [mA]: 275.98

2024-09-27 15:55:53,316<23296>:CapperDecapper@ArduinoController@COM25:INFO - DC Motor Current [mA]: 278.60

2024-09-27 15:55:53,398<23296>:CapperDecapper@ArduinoController@COM25:INFO - DC Motor Current [mA]: 273.75

2024-09-27 15:55:53,480<23296>:CapperDecapper@ArduinoController@COM25:INFO - DC Motor Current [mA]: 264.03

2024-09-27 15:55:53,562<23296>:CapperDecapper@ArduinoController@COM25:INFO - DC Motor Current [mA]: 261.35

2024-09-27 15:55:53,645<23296>:CapperDecapper@ArduinoController@COM25:INFO - DC Motor Current [mA]: 261.70

2024-09-27 15:55:53,726<23296>:CapperDecapper@ArduinoController@COM25:INFO - DC Motor Current [mA]: 253.92

2024-09-27 15:55:53,808<23296>:CapperDecapper@ArduinoController@COM25:INFO - DC Motor Current [mA]: 258.40

2024-09-27 15:55:53,890<23296>:CapperDecapper@ArduinoController@COM25:INFO - DC Motor Current [mA]: 254.28

2024-09-27 15:55:53,972<23296>:CapperDecapper@ArduinoController@COM25:INFO - DC Motor Current [mA]: 244.95

2024-09-27 15:55:54,054<23296>:CapperDecapper@ArduinoController@COM25:INFO - DC Motor Current [mA]: 257.68

2024-09-27 15:55:54,135<23296>:CapperDecapper@ArduinoController@COM25:INFO - DC Motor Current [mA]: 268.05

2024-09-27 15:55:54,218<23296>:CapperDecapper@ArduinoController@COM25:INFO - DC Motor Current [mA]: 275.45

2024-09-27 15:55:54,299<23296>:CapperDecapper@ArduinoController@COM25:INFO - DC Motor Current [mA]: 272.35

2024-09-27 15:55:54,381<23296>:CapperDecapper@ArduinoController@COM25:INFO - DC Motor Current [mA]: 278.95

2024-09-27 15:55:54,463<23296>:CapperDecapper@ArduinoController@COM25:INFO - DC Motor Current [mA]: 280.98

2024-09-27 15:55:54,545<23296>:CapperDecapper@ArduinoController@COM25:INFO - DC Motor Current [mA]: 289.13

2024-09-27 15:55:54,627<23296>:CapperDecapper@ArduinoController@COM25:INFO - DC Motor Current [mA]: 285.82

2024-09-27 15:55:54,709<23296>:CapperDecapper@ArduinoController@COM25:INFO - DC Motor Current [mA]: 282.28

2024-09-27 15:55:54,791<23296>:CapperDecapper@ArduinoController@COM25:INFO - DC Motor Current [mA]: 270.33

2024-09-27 15:55:54,872<23296>:CapperDecapper@ArduinoController@COM25:INFO - DC Motor Current [mA]: 271.42

2024-09-27 15:55:54,954<23296>:CapperDecapper@ArduinoController@COM25:INFO - DC Motor Current [mA]: 256.83

2024-09-27 15:55:55,036<23296>:CapperDecapper@ArduinoController@COM25:INFO - DC Motor Current [mA]: 257.67

2024-09-27 15:55:55,118<23296>:CapperDecapper@ArduinoController@COM25:INFO - DC Motor Current [mA]: 253.73

2024-09-27 15:55:55,201<23296>:CapperDecapper@ArduinoController@COM25:INFO - DC Motor Current [mA]: 248.63

2024-09-27 15:55:55,283<23296>:CapperDecapper@ArduinoController@COM25:INFO - DC Motor Current [mA]: 242.78

2024-09-27 15:55:55,365<23296>:CapperDecapper@ArduinoController@COM25:INFO - DC Motor Current [mA]: 244.75

2024-09-27 15:55:55,447<23296>:CapperDecapper@ArduinoController@COM25:INFO - DC Motor Current [mA]: 238.92

2024-09-27 15:55:55,528<23296>:CapperDecapper@ArduinoController@COM25:INFO - DC Motor Current [mA]: 234.88

2024-09-27 15:55:55,610<23296>:CapperDecapper@ArduinoController@COM25:INFO - DC Motor Current [mA]: 239.33

2024-09-27 15:55:55,692<23296>:CapperDecapper@ArduinoController@COM25:INFO - DC Motor Current [mA]: 247.45

2024-09-27 15:55:55,774<23296>:CapperDecapper@ArduinoController@COM25:INFO - DC Motor Current [mA]: 257.22

2024-09-27 15:55:55,807<23296>:CapperDecapper@ArduinoController@COM25:INFO - Stopped turning wrist

2024-09-27 15:55:57,796<23296>:CapperDecapper@ArduinoController@COM25:INFO - Clamp opened.

2024-09-27 15:55:59,221<20644>:XArm6@192.168.1.204:INFO - Finished moving robot arm to source destination: CapperDecapper@ArduinoController@COM25.

2024-09-27 15:56:27,697<20644>:XArm6@192.168.1.204:INFO - Changed grip on container FALCON_TUBE_50_ML_2363304638976 to a top grip.

2024-09-27 15:56:43,453<20644>:XArm6@192.168.1.204:INFO - Finished moving robot arm to target destination: Isolab_50mL_Foldable_Tube_Rack->deck 1.

2024-09-27 15:56:54,125<20644>:XArm6@192.168.1.204:INFO - Finished moving robot arm to source destination: Isolab_50mL_Foldable_Tube_Rack->deck 1.

2024-09-27 15:57:18,089<20644>:XArm6@192.168.1.204:INFO - Changed grip on container FALCON_TUBE_50_ML_2363304718592 to a sideways grip.

2024-09-27 15:57:23,454<20644>:XArm6@192.168.1.204:INFO - Finished moving robot arm to target destination: UP200ST@192.168.233.233.

2024-09-27 15:57:24,031<14772>:UP200ST@192.168.233.233:INFO - Amplitude changed to 50.0 %.

2024-09-27 15:57:24,059<14772>:UP200ST@192.168.233.233:INFO - Power changed to 50.0 %.

2024-09-27 15:57:24,084<14772>:UP200ST@192.168.233.233:INFO - Time limit activated.

2024-09-27 15:57:24,125<14772>:UP200ST@192.168.233.233:INFO - Time limit changed to 10.0 s.

2024-09-27 15:57:24,126<14772>:UP200ST@192.168.233.233:INFO - Starting sonication...

2024-09-27 15:57:24,162<14772>:UP200ST@192.168.233.233:INFO - Ultrasound turned on.

2024-09-27 15:57:34,207<14772>:UP200ST@192.168.233.233:INFO - Ultrasound turned off.

2024-09-27 15:57:34,207<14772>:UP200ST@192.168.233.233:INFO - Finished sonication.

2024-09-27 15:57:35,935<20644>:XArm6@192.168.1.204:INFO - Finished moving robot arm to source destination: UP200ST@192.168.233.233.

2024-09-27 15:57:55,417<20644>:XArm6@192.168.1.204:INFO - Changed grip on container FALCON_TUBE_50_ML_2363304718592 to a top grip.

2024-09-27 15:58:11,344<20644>:XArm6@192.168.1.204:INFO - Finished moving robot arm to target destination: Isolab_50mL_Foldable_Tube_Rack->deck 1.

2024-09-27 15:58:11,887<13700>:Minerva.API.MinervaAPI:SYNTHESIS_STEP - Sonicate [FALCON_TUBE_50_ML_2363304718592]: 10.0 s; 50 % Amplitude; 50 % Power

2024-09-27 15:58:11,887<13700>:Minerva.API.MinervaAPI:SYNTHESIS_STEP - Remove supernatant and redisperse [FALCON_TUBE_50_ML_2363304638976]: Chemical Ethanol: 15.0 mL

2024-09-27 15:58:20,936<20644>:XArm6@192.168.1.204:INFO - Finished moving robot arm to source destination: Isolab_50mL_Foldable_Tube_Rack->deck 1.

2024-09-27 15:58:46,194<20644>:XArm6@192.168.1.204:INFO - Changed grip on container FALCON_TUBE_50_ML_2363304639264 to a sideways grip.

2024-09-27 15:58:59,000<20644>:XArm6@192.168.1.204:INFO - Finished moving robot arm to target destination: CapperDecapper@ArduinoController@COM25.

2024-09-27 15:58:59,548<23296>:CapperDecapper@ArduinoController@COM25:INFO - Opening Container...

2024-09-27 15:58:59,645<23296>:CapperDecapper@ArduinoController@COM25:INFO - Clamp opened.

2024-09-27 15:59:04,096<23296>:CapperDecapper@ArduinoController@COM25:INFO - Clamp closed.

2024-09-27 15:59:04,129<23296>:CapperDecapper@ArduinoController@COM25:INFO - Wrist turning counterclockwise

2024-09-27 15:59:06,176<23296>:CapperDecapper@ArduinoController@COM25:INFO - Stopped turning wrist

2024-09-27 15:59:07,533<20644>:XArm6@192.168.1.204:INFO - Finished moving robot arm to source destination: CapperDecapper@ArduinoController@COM25.

2024-09-27 15:59:24,018<20644>:XArm6@192.168.1.204:INFO - Finished moving robot arm to target destination: SwitchingValveVici@COM3.

2024-09-27 15:59:24,593<13700>:SwitchingValveVici@COM3:INFO - Valve SwitchingValveVici@COM3 is currently in position 8 (connected to Outlet).

2024-09-27 15:59:24,593<13700>:SwitchingValveVici@COM3:INFO - Moved valve SwitchingValveVici@COM3 to position 8 (connected to Outlet).

2024-09-27 15:59:24,675<22688>:Aladdin@COM12:INFO - Infusion/withdrawing volume set to 30.0 mL at a rate of 70.0 mL/min

2024-09-27 15:59:50,718<22688>:Aladdin@COM12:INFO - Finished withdrawing 30.0 mL at a rate of 70.0 mL/min

2024-09-27 15:59:50,749<13700>:SwitchingValveVici@COM3:INFO - Valve SwitchingValveVici@COM3 is currently in position 8 (connected to Outlet).

2024-09-27 15:59:51,243<13700>:SwitchingValveVici@COM3:INFO - Moved valve SwitchingValveVici@COM3 to position 3 (connected to Waste_Container).

2024-09-27 15:59:51,326<22688>:Aladdin@COM12:INFO - Infusion/withdrawing volume set to 30.0 mL at a rate of 70.0 mL/min

2024-09-27 16:00:17,177<22688>:Aladdin@COM12:INFO - Finished infusing 30.0 mL at a rate of 70.0 mL/min

2024-09-27 16:00:17,178<13700>:SwitchingValveVici@COM3:INFO - Performing purging step into container Container[Waste_Container]: 2.09 L at SwitchingValveVici@COM3->slot 3...

2024-09-27 16:00:17,209<13700>:SwitchingValveVici@COM3:INFO - Valve SwitchingValveVici@COM3 is currently in position 3 (connected to Waste_Container).

2024-09-27 16:00:17,704<13700>:SwitchingValveVici@COM3:INFO - Moved valve SwitchingValveVici@COM3 to position 8 (connected to Outlet).

2024-09-27 16:00:17,801<22688>:Aladdin@COM12:INFO - Infusion/withdrawing volume set to 10.0 mL at a rate of 70.0 mL/min

2024-09-27 16:00:26,670<22688>:Aladdin@COM12:INFO - Finished withdrawing 10.0 mL at a rate of 70.0 mL/min

2024-09-27 16:00:26,703<13700>:SwitchingValveVici@COM3:INFO - Valve SwitchingValveVici@COM3 is currently in position 8 (connected to Outlet).

2024-09-27 16:00:27,197<13700>:SwitchingValveVici@COM3:INFO - Moved valve SwitchingValveVici@COM3 to position 3 (connected to Waste_Container).

2024-09-27 16:00:27,292<22688>:Aladdin@COM12:INFO - Infusion/withdrawing volume set to 10.0 mL at a rate of 70.0 mL/min

2024-09-27 16:00:36,223<22688>:Aladdin@COM12:INFO - Finished infusing 10.0 mL at a rate of 70.0 mL/min

2024-09-27 16:00:36,225<13700>:SwitchingValveVici@COM3:INFO - Performing addition step for chemical Chemical Ethanol: 15.0 mL (Ethanol_wash -> FALCON_TUBE_50_ML_2363304639264)

2024-09-27 16:00:36,256<13700>:SwitchingValveVici@COM3:INFO - Valve SwitchingValveVici@COM3 is currently in position 3 (connected to Waste_Container).

2024-09-27 16:00:36,401<13700>:SwitchingValveVici@COM3:INFO - Moved valve SwitchingValveVici@COM3 to position 2 (connected to Ethanol_wash).

2024-09-27 16:00:36,495<22688>:Aladdin@COM12:INFO - Infusion/withdrawing volume set to 15.0 mL at a rate of 70.0 mL/min

2024-09-27 16:00:49,503<22688>:Aladdin@COM12:INFO - Finished withdrawing 15.0 mL at a rate of 70.0 mL/min

2024-09-27 16:00:49,535<13700>:SwitchingValveVici@COM3:INFO - Valve SwitchingValveVici@COM3 is currently in position 2 (connected to Ethanol_wash).

2024-09-27 16:00:49,948<13700>:SwitchingValveVici@COM3:INFO - Moved valve SwitchingValveVici@COM3 to position 8 (connected to Outlet).

2024-09-27 16:00:50,030<22688>:Aladdin@COM12:INFO - Infusion/withdrawing volume set to 15.0 mL at a rate of 70.0 mL/min

2024-09-27 16:01:03,083<22688>:Aladdin@COM12:INFO - Finished infusing 15.0 mL at a rate of 70.0 mL/min

2024-09-27 16:01:03,084<13700>:SwitchingValveVici@COM3:INFO - Finished adding Chemical Ethanol: 15.0 mL (Ethanol_wash -> FALCON_TUBE_50_ML_2363304639264)

2024-09-27 16:01:04,091<13700>:SwitchingValveVici@COM3:INFO - Performing purging step into container FALCON_TUBE_50_ML: 15.0 mL at SwitchingValveVici@COM3->slot 0...

2024-09-27 16:01:05,304<13700>:SwitchingValveVici@COM3:INFO - Valve SwitchingValveVici@COM3 is currently in position 8 (connected to Outlet).

2024-09-27 16:01:05,304<13700>:SwitchingValveVici@COM3:INFO - Moved valve SwitchingValveVici@COM3 to position 8 (connected to Outlet).

2024-09-27 16:01:05,383<22688>:Aladdin@COM12:INFO - Infusion/withdrawing volume set to 30.0 mL at a rate of 70.0 mL/min

2024-09-27 16:01:31,348<22688>:Aladdin@COM12:INFO - Finished withdrawing 30.0 mL at a rate of 70.0 mL/min

2024-09-27 16:01:32,594<22688>:Aladdin@COM12:INFO - Infusion/withdrawing volume set to 30.0 mL at a rate of 70.0 mL/min

2024-09-27 16:01:58,607<22688>:Aladdin@COM12:INFO - Finished infusing 30.0 mL at a rate of 70.0 mL/min

2024-09-27 16:01:58,608<13700>:Minerva.API.MinervaAPI:SYNTHESIS_STEP - Add Chemical [FALCON_TUBE_50_ML_2363304639264]: FALCON_TUBE_50_ML; Chemical Ethanol: 15.0 mL; 70.0 mL/min; 50 mL Syringe

2024-09-27 16:02:00,147<20644>:XArm6@192.168.1.204:INFO - Finished moving robot arm to source destination: SwitchingValveVici@COM3.

2024-09-27 16:02:08,807<20644>:XArm6@192.168.1.204:INFO - Finished moving robot arm to target destination: UP200ST@192.168.233.233.

2024-09-27 16:02:09,392<14772>:UP200ST@192.168.233.233:INFO - Amplitude changed to 50.0 %.

2024-09-27 16:02:09,419<14772>:UP200ST@192.168.233.233:INFO - Power changed to 50.0 %.

2024-09-27 16:02:09,445<14772>:UP200ST@192.168.233.233:INFO - Time limit activated.

2024-09-27 16:02:09,472<14772>:UP200ST@192.168.233.233:INFO - Time limit changed to 90.0 s.

2024-09-27 16:02:09,472<14772>:UP200ST@192.168.233.233:INFO - Starting sonication...

2024-09-27 16:02:09,497<14772>:UP200ST@192.168.233.233:INFO - Ultrasound turned on.

2024-09-27 16:03:39,547<14772>:UP200ST@192.168.233.233:INFO - Ultrasound turned off.

2024-09-27 16:03:39,548<14772>:UP200ST@192.168.233.233:INFO - Finished sonication.

2024-09-27 16:03:39,548<13700>:Minerva.API.MinervaAPI:SYNTHESIS_STEP - Sonicate [FALCON_TUBE_50_ML_2363304639264]: 90.0 s; 50 % Amplitude; 50 % Power

2024-09-27 16:03:41,160<20644>:XArm6@192.168.1.204:INFO - Finished moving robot arm to source destination: UP200ST@192.168.233.233.

2024-09-27 16:03:53,658<20644>:XArm6@192.168.1.204:INFO - Finished moving robot arm to target destination: CapperDecapper@ArduinoController@COM25.

2024-09-27 16:03:54,206<23296>:CapperDecapper@ArduinoController@COM25:INFO - Closing Container...

2024-09-27 16:03:54,241<23296>:CapperDecapper@ArduinoController@COM25:INFO - Wrist turning clockwise

2024-09-27 16:03:57,349<23296>:CapperDecapper@ArduinoController@COM25:INFO - Wrist turning clockwise

2024-09-27 16:03:57,934<23296>:CapperDecapper@ArduinoController@COM25:INFO - DC Motor Current [mA]: 300.25

2024-09-27 16:03:57,967<23296>:CapperDecapper@ArduinoController@COM25:INFO - Stopped turning wrist

2024-09-27 16:03:59,957<23296>:CapperDecapper@ArduinoController@COM25:INFO - Clamp opened.

2024-09-27 16:04:03,235<20644>:XArm6@192.168.1.204:INFO - Finished moving robot arm to source destination: CapperDecapper@ArduinoController@COM25.

2024-09-27 16:04:29,832<20644>:XArm6@192.168.1.204:INFO - Changed grip on container FALCON_TUBE_50_ML_2363304639264 to a top grip.

2024-09-27 16:04:46,015<20644>:XArm6@192.168.1.204:INFO - Finished moving robot arm to target destination: Isolab_50mL_Foldable_Tube_Rack->deck 1.

2024-09-27 16:04:46,561<13700>:Minerva.API.MinervaAPI:SYNTHESIS_STEP - Remove supernatant and redisperse [FALCON_TUBE_50_ML_2363304639264]: Chemical Ethanol: 15.0 mL

2024-09-27 16:04:56,048<20644>:XArm6@192.168.1.204:INFO - Finished moving robot arm to source destination: Isolab_50mL_Foldable_Tube_Rack->deck 1.

2024-09-27 16:05:20,762<20644>:XArm6@192.168.1.204:INFO - Changed grip on container FALCON_TUBE_50_ML_2363304639552 to a sideways grip.

2024-09-27 16:05:33,562<20644>:XArm6@192.168.1.204:INFO - Finished moving robot arm to target destination: CapperDecapper@ArduinoController@COM25.

2024-09-27 16:05:34,107<23296>:CapperDecapper@ArduinoController@COM25:INFO - Opening Container...

2024-09-27 16:05:34,205<23296>:CapperDecapper@ArduinoController@COM25:INFO - Clamp opened.

2024-09-27 16:05:38,656<23296>:CapperDecapper@ArduinoController@COM25:INFO - Clamp closed.

2024-09-27 16:05:38,689<23296>:CapperDecapper@ArduinoController@COM25:INFO - Wrist turning counterclockwise

2024-09-27 16:05:40,732<23296>:CapperDecapper@ArduinoController@COM25:INFO - Stopped turning wrist

2024-09-27 16:05:42,087<20644>:XArm6@192.168.1.204:INFO - Finished moving robot arm to source destination: CapperDecapper@ArduinoController@COM25.

2024-09-27 16:05:59,139<20644>:XArm6@192.168.1.204:INFO - Finished moving robot arm to target destination: SwitchingValveVici@COM3.

2024-09-27 16:05:59,714<13700>:SwitchingValveVici@COM3:INFO - Valve SwitchingValveVici@COM3 is currently in position 8 (connected to Outlet).

2024-09-27 16:05:59,714<13700>:SwitchingValveVici@COM3:INFO - Moved valve SwitchingValveVici@COM3 to position 8 (connected to Outlet).

2024-09-27 16:05:59,797<22688>:Aladdin@COM12:INFO - Infusion/withdrawing volume set to 30.0 mL at a rate of 70.0 mL/min

2024-09-27 16:06:25,746<22688>:Aladdin@COM12:INFO - Finished withdrawing 30.0 mL at a rate of 70.0 mL/min

2024-09-27 16:06:25,776<13700>:SwitchingValveVici@COM3:INFO - Valve SwitchingValveVici@COM3 is currently in position 8 (connected to Outlet).

2024-09-27 16:06:26,271<13700>:SwitchingValveVici@COM3:INFO - Moved valve SwitchingValveVici@COM3 to position 3 (connected to Waste_Container).

2024-09-27 16:06:26,353<22688>:Aladdin@COM12:INFO - Infusion/withdrawing volume set to 30.0 mL at a rate of 70.0 mL/min

2024-09-27 16:06:52,318<22688>:Aladdin@COM12:INFO - Finished infusing 30.0 mL at a rate of 70.0 mL/min

2024-09-27 16:06:52,319<13700>:SwitchingValveVici@COM3:INFO - Performing purging step into container Container[Waste_Container]: 2.12 L at SwitchingValveVici@COM3->slot 3...

2024-09-27 16:06:52,348<13700>:SwitchingValveVici@COM3:INFO - Valve SwitchingValveVici@COM3 is currently in position 3 (connected to Waste_Container).

2024-09-27 16:06:52,843<13700>:SwitchingValveVici@COM3:INFO - Moved valve SwitchingValveVici@COM3 to position 8 (connected to Outlet).

2024-09-27 16:06:52,925<22688>:Aladdin@COM12:INFO - Infusion/withdrawing volume set to 10.0 mL at a rate of 70.0 mL/min

2024-09-27 16:07:01,825<22688>:Aladdin@COM12:INFO - Finished withdrawing 10.0 mL at a rate of 70.0 mL/min

2024-09-27 16:07:01,855<13700>:SwitchingValveVici@COM3:INFO - Valve SwitchingValveVici@COM3 is currently in position 8 (connected to Outlet).

2024-09-27 16:07:02,352<13700>:SwitchingValveVici@COM3:INFO - Moved valve SwitchingValveVici@COM3 to position 3 (connected to Waste_Container).

2024-09-27 16:07:02,449<22688>:Aladdin@COM12:INFO - Infusion/withdrawing volume set to 10.0 mL at a rate of 70.0 mL/min

2024-09-27 16:07:11,348<22688>:Aladdin@COM12:INFO - Finished infusing 10.0 mL at a rate of 70.0 mL/min

2024-09-27 16:07:11,350<13700>:SwitchingValveVici@COM3:INFO - Performing addition step for chemical Chemical Ethanol: 15.0 mL (Ethanol_wash -> FALCON_TUBE_50_ML_2363304639552)

2024-09-27 16:07:11,381<13700>:SwitchingValveVici@COM3:INFO - Valve SwitchingValveVici@COM3 is currently in position 3 (connected to Waste_Container).

2024-09-27 16:07:11,537<13700>:SwitchingValveVici@COM3:INFO - Moved valve SwitchingValveVici@COM3 to position 2 (connected to Ethanol_wash).

2024-09-27 16:07:11,620<22688>:Aladdin@COM12:INFO - Infusion/withdrawing volume set to 15.0 mL at a rate of 70.0 mL/min

2024-09-27 16:07:24,627<22688>:Aladdin@COM12:INFO - Finished withdrawing 15.0 mL at a rate of 70.0 mL/min

2024-09-27 16:07:24,659<13700>:SwitchingValveVici@COM3:INFO - Valve SwitchingValveVici@COM3 is currently in position 2 (connected to Ethanol_wash).

2024-09-27 16:07:25,072<13700>:SwitchingValveVici@COM3:INFO - Moved valve SwitchingValveVici@COM3 to position 8 (connected to Outlet).

2024-09-27 16:07:25,153<22688>:Aladdin@COM12:INFO - Infusion/withdrawing volume set to 15.0 mL at a rate of 70.0 mL/min

2024-09-27 16:07:38,240<22688>:Aladdin@COM12:INFO - Finished infusing 15.0 mL at a rate of 70.0 mL/min

2024-09-27 16:07:38,241<13700>:SwitchingValveVici@COM3:INFO - Finished adding Chemical Ethanol: 15.0 mL (Ethanol_wash -> FALCON_TUBE_50_ML_2363304639552)

2024-09-27 16:07:39,252<13700>:SwitchingValveVici@COM3:INFO - Performing purging step into container FALCON_TUBE_50_ML: 15.0 mL at SwitchingValveVici@COM3->slot 0...

2024-09-27 16:07:40,460<13700>:SwitchingValveVici@COM3:INFO - Valve SwitchingValveVici@COM3 is currently in position 8 (connected to Outlet).

2024-09-27 16:07:40,461<13700>:SwitchingValveVici@COM3:INFO - Moved valve SwitchingValveVici@COM3 to position 8 (connected to Outlet).

2024-09-27 16:07:40,557<22688>:Aladdin@COM12:INFO - Infusion/withdrawing volume set to 30.0 mL at a rate of 70.0 mL/min

2024-09-27 16:08:06,570<22688>:Aladdin@COM12:INFO - Finished withdrawing 30.0 mL at a rate of 70.0 mL/min

2024-09-27 16:08:07,832<22688>:Aladdin@COM12:INFO - Infusion/withdrawing volume set to 30.0 mL at a rate of 70.0 mL/min

2024-09-27 16:08:33,893<22688>:Aladdin@COM12:INFO - Finished infusing 30.0 mL at a rate of 70.0 mL/min

2024-09-27 16:08:33,894<13700>:Minerva.API.MinervaAPI:SYNTHESIS_STEP - Add Chemical [FALCON_TUBE_50_ML_2363304639552]: FALCON_TUBE_50_ML; Chemical Ethanol: 15.0 mL; 70.0 mL/min; 50 mL Syringe

2024-09-27 16:08:35,444<20644>:XArm6@192.168.1.204:INFO - Finished moving robot arm to source destination: SwitchingValveVici@COM3.

2024-09-27 16:08:44,346<20644>:XArm6@192.168.1.204:INFO - Finished moving robot arm to target destination: UP200ST@192.168.233.233.

2024-09-27 16:08:44,930<14772>:UP200ST@192.168.233.233:INFO - Amplitude changed to 50.0 %.

2024-09-27 16:08:44,958<14772>:UP200ST@192.168.233.233:INFO - Power changed to 50.0 %.

2024-09-27 16:08:44,983<14772>:UP200ST@192.168.233.233:INFO - Time limit activated.

2024-09-27 16:08:45,010<14772>:UP200ST@192.168.233.233:INFO - Time limit changed to 90.0 s.

2024-09-27 16:08:45,010<14772>:UP200ST@192.168.233.233:INFO - Starting sonication...

2024-09-27 16:08:45,035<14772>:UP200ST@192.168.233.233:INFO - Ultrasound turned on.

2024-09-27 16:10:15,073<14772>:UP200ST@192.168.233.233:INFO - Ultrasound turned off.

2024-09-27 16:10:15,073<14772>:UP200ST@192.168.233.233:INFO - Finished sonication.

2024-09-27 16:10:15,073<13700>:Minerva.API.MinervaAPI:SYNTHESIS_STEP - Sonicate [FALCON_TUBE_50_ML_2363304639552]: 90.0 s; 50 % Amplitude; 50 % Power

2024-09-27 16:10:18,292<20644>:XArm6@192.168.1.204:INFO - Finished moving robot arm to source destination: UP200ST@192.168.233.233.

2024-09-27 16:10:30,306<20644>:XArm6@192.168.1.204:INFO - Finished moving robot arm to target destination: CapperDecapper@ArduinoController@COM25.

2024-09-27 16:10:30,853<23296>:CapperDecapper@ArduinoController@COM25:INFO - Closing Container...

2024-09-27 16:10:30,885<23296>:CapperDecapper@ArduinoController@COM25:INFO - Wrist turning clockwise

2024-09-27 16:10:34,009<23296>:CapperDecapper@ArduinoController@COM25:INFO - Wrist turning clockwise

2024-09-27 16:10:34,594<23296>:CapperDecapper@ArduinoController@COM25:INFO - DC Motor Current [mA]: 272.63

2024-09-27 16:10:34,677<23296>:CapperDecapper@ArduinoController@COM25:INFO - DC Motor Current [mA]: 273.88

2024-09-27 16:10:34,758<23296>:CapperDecapper@ArduinoController@COM25:INFO - DC Motor Current [mA]: 264.75

2024-09-27 16:10:34,840<23296>:CapperDecapper@ArduinoController@COM25:INFO - DC Motor Current [mA]: 260.85

2024-09-27 16:10:34,922<23296>:CapperDecapper@ArduinoController@COM25:INFO - DC Motor Current [mA]: 264.10

2024-09-27 16:10:35,004<23296>:CapperDecapper@ArduinoController@COM25:INFO - DC Motor Current [mA]: 252.15

2024-09-27 16:10:35,086<23296>:CapperDecapper@ArduinoController@COM25:INFO - DC Motor Current [mA]: 239.93

2024-09-27 16:10:35,169<23296>:CapperDecapper@ArduinoController@COM25:INFO - DC Motor Current [mA]: 252.23

2024-09-27 16:10:35,251<23296>:CapperDecapper@ArduinoController@COM25:INFO - DC Motor Current [mA]: 253.42

2024-09-27 16:10:35,333<23296>:CapperDecapper@ArduinoController@COM25:INFO - DC Motor Current [mA]: 256.73

2024-09-27 16:10:35,414<23296>:CapperDecapper@ArduinoController@COM25:INFO - DC Motor Current [mA]: 258.02

2024-09-27 16:10:35,496<23296>:CapperDecapper@ArduinoController@COM25:INFO - DC Motor Current [mA]: 259.65

2024-09-27 16:10:35,578<23296>:CapperDecapper@ArduinoController@COM25:INFO - DC Motor Current [mA]: 262.05

2024-09-27 16:10:35,660<23296>:CapperDecapper@ArduinoController@COM25:INFO - DC Motor Current [mA]: 269.17

2024-09-27 16:10:35,742<23296>:CapperDecapper@ArduinoController@COM25:INFO - DC Motor Current [mA]: 271.75

2024-09-27 16:10:35,824<23296>:CapperDecapper@ArduinoController@COM25:INFO - DC Motor Current [mA]: 283.48

2024-09-27 16:10:35,906<23296>:CapperDecapper@ArduinoController@COM25:INFO - DC Motor Current [mA]: 290.35

2024-09-27 16:10:35,987<23296>:CapperDecapper@ArduinoController@COM25:INFO - DC Motor Current [mA]: 291.55

2024-09-27 16:10:36,069<23296>:CapperDecapper@ArduinoController@COM25:INFO - DC Motor Current [mA]: 274.60

2024-09-27 16:10:36,151<23296>:CapperDecapper@ArduinoController@COM25:INFO - DC Motor Current [mA]: 263.63

2024-09-27 16:10:36,233<23296>:CapperDecapper@ArduinoController@COM25:INFO - DC Motor Current [mA]: 252.10

2024-09-27 16:10:36,314<23296>:CapperDecapper@ArduinoController@COM25:INFO - DC Motor Current [mA]: 245.50

2024-09-27 16:10:36,396<23296>:CapperDecapper@ArduinoController@COM25:INFO - DC Motor Current [mA]: 241.90

2024-09-27 16:10:36,478<23296>:CapperDecapper@ArduinoController@COM25:INFO - DC Motor Current [mA]: 230.77

2024-09-27 16:10:36,560<23296>:CapperDecapper@ArduinoController@COM25:INFO - DC Motor Current [mA]: 236.45

2024-09-27 16:10:36,642<23296>:CapperDecapper@ArduinoController@COM25:INFO - DC Motor Current [mA]: 235.10

2024-09-27 16:10:36,724<23296>:CapperDecapper@ArduinoController@COM25:INFO - DC Motor Current [mA]: 240.63

2024-09-27 16:10:36,806<23296>:CapperDecapper@ArduinoController@COM25:INFO - DC Motor Current [mA]: 245.50

2024-09-27 16:10:36,888<23296>:CapperDecapper@ArduinoController@COM25:INFO - DC Motor Current [mA]: 244.13

2024-09-27 16:10:36,970<23296>:CapperDecapper@ArduinoController@COM25:INFO - DC Motor Current [mA]: 253.15

2024-09-27 16:10:37,052<23296>:CapperDecapper@ArduinoController@COM25:INFO - DC Motor Current [mA]: 255.35

2024-09-27 16:10:37,134<23296>:CapperDecapper@ArduinoController@COM25:INFO - DC Motor Current [mA]: 254.40

2024-09-27 16:10:37,167<23296>:CapperDecapper@ArduinoController@COM25:INFO - Stopped turning wrist

2024-09-27 16:10:39,157<23296>:CapperDecapper@ArduinoController@COM25:INFO - Clamp opened.

2024-09-27 16:10:42,439<20644>:XArm6@192.168.1.204:INFO - Finished moving robot arm to source destination: CapperDecapper@ArduinoController@COM25.

2024-09-27 16:11:09,372<20644>:XArm6@192.168.1.204:INFO - Changed grip on container FALCON_TUBE_50_ML_2363304639552 to a top grip.

2024-09-27 16:11:24,595<20644>:XArm6@192.168.1.204:INFO - Finished moving robot arm to target destination: Isolab_50mL_Foldable_Tube_Rack->deck 1.

2024-09-27 16:11:28,692<13936>:DHT22Sensor@ArduinoController@COM25:INFO - Temperature: 22.30 C, Humidity: 26.30 %

2024-09-27 16:11:33,597<20644>:XArm6@192.168.1.204:INFO - Finished moving robot arm to source destination: Isolab_50mL_Foldable_Tube_Rack->deck 1.

2024-09-27 16:11:59,349<20644>:XArm6@192.168.1.204:INFO - Changed grip on container FALCON_TUBE_50_ML_2363304718592 to a sideways grip.

2024-09-27 16:12:04,924<20644>:XArm6@192.168.1.204:INFO - Finished moving robot arm to target destination: UP200ST@192.168.233.233.

2024-09-27 16:12:05,504<14772>:UP200ST@192.168.233.233:INFO - Amplitude changed to 50.0 %.

2024-09-27 16:12:05,533<14772>:UP200ST@192.168.233.233:INFO - Power changed to 50.0 %.

2024-09-27 16:12:05,558<14772>:UP200ST@192.168.233.233:INFO - Time limit activated.

2024-09-27 16:12:05,585<14772>:UP200ST@192.168.233.233:INFO - Time limit changed to 10.0 s.

2024-09-27 16:12:05,585<14772>:UP200ST@192.168.233.233:INFO - Starting sonication...

2024-09-27 16:12:05,611<14772>:UP200ST@192.168.233.233:INFO - Ultrasound turned on.

2024-09-27 16:12:15,648<14772>:UP200ST@192.168.233.233:INFO - Ultrasound turned off.

2024-09-27 16:12:15,648<14772>:UP200ST@192.168.233.233:INFO - Finished sonication.

2024-09-27 16:12:17,385<20644>:XArm6@192.168.1.204:INFO - Finished moving robot arm to source destination: UP200ST@192.168.233.233.

2024-09-27 16:12:37,095<20644>:XArm6@192.168.1.204:INFO - Changed grip on container FALCON_TUBE_50_ML_2363304718592 to a top grip.

2024-09-27 16:12:52,668<20644>:XArm6@192.168.1.204:INFO - Finished moving robot arm to target destination: Isolab_50mL_Foldable_Tube_Rack->deck 1.

2024-09-27 16:12:53,216<13700>:Minerva.API.MinervaAPI:SYNTHESIS_STEP - Sonicate [FALCON_TUBE_50_ML_2363304718592]: 10.0 s; 50 % Amplitude; 50 % Power

2024-09-27 16:12:53,216<13700>:Minerva.API.MinervaAPI:SYNTHESIS_STEP - Remove supernatant and redisperse [FALCON_TUBE_50_ML_2363304639552]: Chemical Ethanol: 15.0 mL

2024-09-27 16:13:01,470<20644>:XArm6@192.168.1.204:INFO - Finished moving robot arm to source destination: Isolab_50mL_Foldable_Tube_Rack->deck 1.

2024-09-27 16:13:26,469<20644>:XArm6@192.168.1.204:INFO - Changed grip on container FALCON_TUBE_50_ML_2363304639840 to a sideways grip.

2024-09-27 16:13:39,265<20644>:XArm6@192.168.1.204:INFO - Finished moving robot arm to target destination: CapperDecapper@ArduinoController@COM25.

2024-09-27 16:13:39,805<23296>:CapperDecapper@ArduinoController@COM25:INFO - Opening Container...

2024-09-27 16:13:39,903<23296>:CapperDecapper@ArduinoController@COM25:INFO - Clamp opened.

2024-09-27 16:13:44,346<23296>:CapperDecapper@ArduinoController@COM25:INFO - Clamp closed.

2024-09-27 16:13:44,378<23296>:CapperDecapper@ArduinoController@COM25:INFO - Wrist turning counterclockwise

2024-09-27 16:13:46,422<23296>:CapperDecapper@ArduinoController@COM25:INFO - Stopped turning wrist

2024-09-27 16:13:47,784<20644>:XArm6@192.168.1.204:INFO - Finished moving robot arm to source destination: CapperDecapper@ArduinoController@COM25.

2024-09-27 16:14:04,360<20644>:XArm6@192.168.1.204:INFO - Finished moving robot arm to target destination: SwitchingValveVici@COM3.

2024-09-27 16:14:04,939<13700>:SwitchingValveVici@COM3:INFO - Valve SwitchingValveVici@COM3 is currently in position 8 (connected to Outlet).

2024-09-27 16:14:04,940<13700>:SwitchingValveVici@COM3:INFO - Moved valve SwitchingValveVici@COM3 to position 8 (connected to Outlet).

2024-09-27 16:14:05,022<22688>:Aladdin@COM12:INFO - Infusion/withdrawing volume set to 30.0 mL at a rate of 70.0 mL/min

2024-09-27 16:14:31,035<22688>:Aladdin@COM12:INFO - Finished withdrawing 30.0 mL at a rate of 70.0 mL/min

2024-09-27 16:14:31,067<13700>:SwitchingValveVici@COM3:INFO - Valve SwitchingValveVici@COM3 is currently in position 8 (connected to Outlet).

2024-09-27 16:14:31,563<13700>:SwitchingValveVici@COM3:INFO - Moved valve SwitchingValveVici@COM3 to position 3 (connected to Waste_Container).

2024-09-27 16:14:31,643<22688>:Aladdin@COM12:INFO - Infusion/withdrawing volume set to 30.0 mL at a rate of 70.0 mL/min

2024-09-27 16:14:57,607<22688>:Aladdin@COM12:INFO - Finished infusing 30.0 mL at a rate of 70.0 mL/min

2024-09-27 16:14:57,608<13700>:SwitchingValveVici@COM3:INFO - Performing purging step into container Container[Waste_Container]: 2.15 L at SwitchingValveVici@COM3->slot 3...

2024-09-27 16:14:57,639<13700>:SwitchingValveVici@COM3:INFO - Valve SwitchingValveVici@COM3 is currently in position 3 (connected to Waste_Container).

2024-09-27 16:14:58,134<13700>:SwitchingValveVici@COM3:INFO - Moved valve SwitchingValveVici@COM3 to position 8 (connected to Outlet).

2024-09-27 16:14:58,215<22688>:Aladdin@COM12:INFO - Infusion/withdrawing volume set to 10.0 mL at a rate of 70.0 mL/min

2024-09-27 16:15:07,100<22688>:Aladdin@COM12:INFO - Finished withdrawing 10.0 mL at a rate of 70.0 mL/min

2024-09-27 16:15:07,130<13700>:SwitchingValveVici@COM3:INFO - Valve SwitchingValveVici@COM3 is currently in position 8 (connected to Outlet).

2024-09-27 16:15:07,626<13700>:SwitchingValveVici@COM3:INFO - Moved valve SwitchingValveVici@COM3 to position 3 (connected to Waste_Container).

2024-09-27 16:15:07,722<22688>:Aladdin@COM12:INFO - Infusion/withdrawing volume set to 10.0 mL at a rate of 70.0 mL/min

2024-09-27 16:15:16,558<22688>:Aladdin@COM12:INFO - Finished infusing 10.0 mL at a rate of 70.0 mL/min

2024-09-27 16:15:16,560<13700>:SwitchingValveVici@COM3:INFO - Performing addition step for chemical Chemical Ethanol: 15.0 mL (Ethanol_wash -> FALCON_TUBE_50_ML_2363304639840)

2024-09-27 16:15:16,591<13700>:SwitchingValveVici@COM3:INFO - Valve SwitchingValveVici@COM3 is currently in position 3 (connected to Waste_Container).

2024-09-27 16:15:16,747<13700>:SwitchingValveVici@COM3:INFO - Moved valve SwitchingValveVici@COM3 to position 2 (connected to Ethanol_wash).

2024-09-27 16:15:16,829<22688>:Aladdin@COM12:INFO - Infusion/withdrawing volume set to 15.0 mL at a rate of 70.0 mL/min

2024-09-27 16:15:29,883<22688>:Aladdin@COM12:INFO - Finished withdrawing 15.0 mL at a rate of 70.0 mL/min

2024-09-27 16:15:29,916<13700>:SwitchingValveVici@COM3:INFO - Valve SwitchingValveVici@COM3 is currently in position 2 (connected to Ethanol_wash).

2024-09-27 16:15:30,330<13700>:SwitchingValveVici@COM3:INFO - Moved valve SwitchingValveVici@COM3 to position 8 (connected to Outlet).

2024-09-27 16:15:30,410<22688>:Aladdin@COM12:INFO - Infusion/withdrawing volume set to 15.0 mL at a rate of 70.0 mL/min

2024-09-27 16:15:43,513<22688>:Aladdin@COM12:INFO - Finished infusing 15.0 mL at a rate of 70.0 mL/min

2024-09-27 16:15:43,513<13700>:SwitchingValveVici@COM3:INFO - Finished adding Chemical Ethanol: 15.0 mL (Ethanol_wash -> FALCON_TUBE_50_ML_2363304639840)

2024-09-27 16:15:44,527<13700>:SwitchingValveVici@COM3:INFO - Performing purging step into container FALCON_TUBE_50_ML: 15.0 mL at SwitchingValveVici@COM3->slot 0...

2024-09-27 16:15:45,733<13700>:SwitchingValveVici@COM3:INFO - Valve SwitchingValveVici@COM3 is currently in position 8 (connected to Outlet).

2024-09-27 16:15:45,734<13700>:SwitchingValveVici@COM3:INFO - Moved valve SwitchingValveVici@COM3 to position 8 (connected to Outlet).

2024-09-27 16:15:45,828<22688>:Aladdin@COM12:INFO - Infusion/withdrawing volume set to 30.0 mL at a rate of 70.0 mL/min

2024-09-27 16:16:11,761<22688>:Aladdin@COM12:INFO - Finished withdrawing 30.0 mL at a rate of 70.0 mL/min

2024-09-27 16:16:13,023<22688>:Aladdin@COM12:INFO - Infusion/withdrawing volume set to 30.0 mL at a rate of 70.0 mL/min

2024-09-27 16:16:38,955<22688>:Aladdin@COM12:INFO - Finished infusing 30.0 mL at a rate of 70.0 mL/min

2024-09-27 16:16:38,956<13700>:Minerva.API.MinervaAPI:SYNTHESIS_STEP - Add Chemical [FALCON_TUBE_50_ML_2363304639840]: FALCON_TUBE_50_ML; Chemical Ethanol: 15.0 mL; 70.0 mL/min; 50 mL Syringe

2024-09-27 16:16:40,507<20644>:XArm6@192.168.1.204:INFO - Finished moving robot arm to source destination: SwitchingValveVici@COM3.

2024-09-27 16:16:49,168<20644>:XArm6@192.168.1.204:INFO - Finished moving robot arm to target destination: UP200ST@192.168.233.233.

2024-09-27 16:16:49,745<14772>:UP200ST@192.168.233.233:INFO - Amplitude changed to 50.0 %.

2024-09-27 16:16:49,773<14772>:UP200ST@192.168.233.233:INFO - Power changed to 50.0 %.

2024-09-27 16:16:49,797<14772>:UP200ST@192.168.233.233:INFO - Time limit activated.

2024-09-27 16:16:49,825<14772>:UP200ST@192.168.233.233:INFO - Time limit changed to 90.0 s.

2024-09-27 16:16:49,825<14772>:UP200ST@192.168.233.233:INFO - Starting sonication...

2024-09-27 16:16:49,850<14772>:UP200ST@192.168.233.233:INFO - Ultrasound turned on.

2024-09-27 16:18:19,946<14772>:UP200ST@192.168.233.233:INFO - Ultrasound turned off.

2024-09-27 16:18:19,946<14772>:UP200ST@192.168.233.233:INFO - Finished sonication.

2024-09-27 16:18:19,946<13700>:Minerva.API.MinervaAPI:SYNTHESIS_STEP - Sonicate [FALCON_TUBE_50_ML_2363304639840]: 90.0 s; 50 % Amplitude; 50 % Power

2024-09-27 16:18:21,556<20644>:XArm6@192.168.1.204:INFO - Finished moving robot arm to source destination: UP200ST@192.168.233.233.

2024-09-27 16:18:34,260<20644>:XArm6@192.168.1.204:INFO - Finished moving robot arm to target destination: CapperDecapper@ArduinoController@COM25.

2024-09-27 16:18:34,808<23296>:CapperDecapper@ArduinoController@COM25:INFO - Closing Container...

2024-09-27 16:18:34,839<23296>:CapperDecapper@ArduinoController@COM25:INFO - Wrist turning clockwise

2024-09-27 16:18:37,965<23296>:CapperDecapper@ArduinoController@COM25:INFO - Wrist turning clockwise

2024-09-27 16:18:38,563<23296>:CapperDecapper@ArduinoController@COM25:INFO - DC Motor Current [mA]: 301.40

2024-09-27 16:18:38,596<23296>:CapperDecapper@ArduinoController@COM25:INFO - Stopped turning wrist

2024-09-27 16:18:40,588<23296>:CapperDecapper@ArduinoController@COM25:INFO - Clamp opened.

2024-09-27 16:18:42,007<20644>:XArm6@192.168.1.204:INFO - Finished moving robot arm to source destination: CapperDecapper@ArduinoController@COM25.

2024-09-27 16:19:10,465<20644>:XArm6@192.168.1.204:INFO - Changed grip on container FALCON_TUBE_50_ML_2363304639840 to a top grip.

2024-09-27 16:19:26,034<20644>:XArm6@192.168.1.204:INFO - Finished moving robot arm to target destination: Isolab_50mL_Foldable_Tube_Rack->deck 1.

2024-09-27 16:19:26,583<13700>:Minerva.API.MinervaAPI:SYNTHESIS_STEP - Remove supernatant and redisperse [FALCON_TUBE_50_ML_2363304639840]: Chemical Ethanol: 15.0 mL

2024-09-27 16:19:34,963<20644>:XArm6@192.168.1.204:INFO - Finished moving robot arm to source destination: Isolab_50mL_Foldable_Tube_Rack->deck 1.

2024-09-27 16:19:59,978<20644>:XArm6@192.168.1.204:INFO - Changed grip on container FALCON_TUBE_50_ML_2363304640128 to a sideways grip.

2024-09-27 16:20:13,353<20644>:XArm6@192.168.1.204:INFO - Finished moving robot arm to target destination: CapperDecapper@ArduinoController@COM25.

2024-09-27 16:20:13,897<23296>:CapperDecapper@ArduinoController@COM25:INFO - Opening Container...

2024-09-27 16:20:13,994<23296>:CapperDecapper@ArduinoController@COM25:INFO - Clamp opened.

2024-09-27 16:20:18,448<23296>:CapperDecapper@ArduinoController@COM25:INFO - Clamp closed.

2024-09-27 16:20:18,481<23296>:CapperDecapper@ArduinoController@COM25:INFO - Wrist turning counterclockwise

2024-09-27 16:20:20,526<23296>:CapperDecapper@ArduinoController@COM25:INFO - Stopped turning wrist

2024-09-27 16:20:21,879<20644>:XArm6@192.168.1.204:INFO - Finished moving robot arm to source destination: CapperDecapper@ArduinoController@COM25.

2024-09-27 16:20:38,942<20644>:XArm6@192.168.1.204:INFO - Finished moving robot arm to target destination: SwitchingValveVici@COM3.

2024-09-27 16:20:39,520<13700>:SwitchingValveVici@COM3:INFO - Valve SwitchingValveVici@COM3 is currently in position 8 (connected to Outlet).

2024-09-27 16:20:39,521<13700>:SwitchingValveVici@COM3:INFO - Moved valve SwitchingValveVici@COM3 to position 8 (connected to Outlet).

2024-09-27 16:20:39,606<22688>:Aladdin@COM12:INFO - Infusion/withdrawing volume set to 30.0 mL at a rate of 70.0 mL/min

2024-09-27 16:21:05,603<22688>:Aladdin@COM12:INFO - Finished withdrawing 30.0 mL at a rate of 70.0 mL/min

2024-09-27 16:21:05,633<13700>:SwitchingValveVici@COM3:INFO - Valve SwitchingValveVici@COM3 is currently in position 8 (connected to Outlet).

2024-09-27 16:21:06,127<13700>:SwitchingValveVici@COM3:INFO - Moved valve SwitchingValveVici@COM3 to position 3 (connected to Waste_Container).

2024-09-27 16:21:06,210<22688>:Aladdin@COM12:INFO - Infusion/withdrawing volume set to 30.0 mL at a rate of 70.0 mL/min

2024-09-27 16:21:32,252<22688>:Aladdin@COM12:INFO - Finished infusing 30.0 mL at a rate of 70.0 mL/min

2024-09-27 16:21:32,253<13700>:SwitchingValveVici@COM3:INFO - Performing purging step into container Container[Waste_Container]: 2.18 L at SwitchingValveVici@COM3->slot 3...

2024-09-27 16:21:32,284<13700>:SwitchingValveVici@COM3:INFO - Valve SwitchingValveVici@COM3 is currently in position 3 (connected to Waste_Container).

2024-09-27 16:21:32,779<13700>:SwitchingValveVici@COM3:INFO - Moved valve SwitchingValveVici@COM3 to position 8 (connected to Outlet).

2024-09-27 16:21:32,875<22688>:Aladdin@COM12:INFO - Infusion/withdrawing volume set to 10.0 mL at a rate of 70.0 mL/min

2024-09-27 16:21:41,766<22688>:Aladdin@COM12:INFO - Finished withdrawing 10.0 mL at a rate of 70.0 mL/min

2024-09-27 16:21:41,798<13700>:SwitchingValveVici@COM3:INFO - Valve SwitchingValveVici@COM3 is currently in position 8 (connected to Outlet).

2024-09-27 16:21:42,294<13700>:SwitchingValveVici@COM3:INFO - Moved valve SwitchingValveVici@COM3 to position 3 (connected to Waste_Container).

2024-09-27 16:21:42,388<22688>:Aladdin@COM12:INFO - Infusion/withdrawing volume set to 10.0 mL at a rate of 70.0 mL/min

2024-09-27 16:21:51,277<22688>:Aladdin@COM12:INFO - Finished infusing 10.0 mL at a rate of 70.0 mL/min

2024-09-27 16:21:51,280<13700>:SwitchingValveVici@COM3:INFO - Performing addition step for chemical Chemical Ethanol: 15.0 mL (Ethanol_wash -> FALCON_TUBE_50_ML_2363304640128)

2024-09-27 16:21:51,311<13700>:SwitchingValveVici@COM3:INFO - Valve SwitchingValveVici@COM3 is currently in position 3 (connected to Waste_Container).

2024-09-27 16:21:51,467<13700>:SwitchingValveVici@COM3:INFO - Moved valve SwitchingValveVici@COM3 to position 2 (connected to Ethanol_wash).

2024-09-27 16:21:51,565<22688>:Aladdin@COM12:INFO - Infusion/withdrawing volume set to 15.0 mL at a rate of 70.0 mL/min

2024-09-27 16:22:04,612<22688>:Aladdin@COM12:INFO - Finished withdrawing 15.0 mL at a rate of 70.0 mL/min

2024-09-27 16:22:04,645<13700>:SwitchingValveVici@COM3:INFO - Valve SwitchingValveVici@COM3 is currently in position 2 (connected to Ethanol_wash).

2024-09-27 16:22:05,059<13700>:SwitchingValveVici@COM3:INFO - Moved valve SwitchingValveVici@COM3 to position 8 (connected to Outlet).

2024-09-27 16:22:05,155<22688>:Aladdin@COM12:INFO - Infusion/withdrawing volume set to 15.0 mL at a rate of 70.0 mL/min

2024-09-27 16:22:18,217<22688>:Aladdin@COM12:INFO - Finished infusing 15.0 mL at a rate of 70.0 mL/min

2024-09-27 16:22:18,217<13700>:SwitchingValveVici@COM3:INFO - Finished adding Chemical Ethanol: 15.0 mL (Ethanol_wash -> FALCON_TUBE_50_ML_2363304640128)

2024-09-27 16:22:19,226<13700>:SwitchingValveVici@COM3:INFO - Performing purging step into container FALCON_TUBE_50_ML: 15.0 mL at SwitchingValveVici@COM3->slot 0...

2024-09-27 16:22:20,437<13700>:SwitchingValveVici@COM3:INFO - Valve SwitchingValveVici@COM3 is currently in position 8 (connected to Outlet).

2024-09-27 16:22:20,438<13700>:SwitchingValveVici@COM3:INFO - Moved valve SwitchingValveVici@COM3 to position 8 (connected to Outlet).

2024-09-27 16:22:20,534<22688>:Aladdin@COM12:INFO - Infusion/withdrawing volume set to 30.0 mL at a rate of 70.0 mL/min

2024-09-27 16:22:46,577<22688>:Aladdin@COM12:INFO - Finished withdrawing 30.0 mL at a rate of 70.0 mL/min

2024-09-27 16:22:47,839<22688>:Aladdin@COM12:INFO - Infusion/withdrawing volume set to 30.0 mL at a rate of 70.0 mL/min

2024-09-27 16:23:13,770<22688>:Aladdin@COM12:INFO - Finished infusing 30.0 mL at a rate of 70.0 mL/min

2024-09-27 16:23:13,771<13700>:Minerva.API.MinervaAPI:SYNTHESIS_STEP - Add Chemical [FALCON_TUBE_50_ML_2363304640128]: FALCON_TUBE_50_ML; Chemical Ethanol: 15.0 mL; 70.0 mL/min; 50 mL Syringe

2024-09-27 16:23:15,321<20644>:XArm6@192.168.1.204:INFO - Finished moving robot arm to source destination: SwitchingValveVici@COM3.

2024-09-27 16:23:23,994<20644>:XArm6@192.168.1.204:INFO - Finished moving robot arm to target destination: UP200ST@192.168.233.233.

2024-09-27 16:23:24,578<14772>:UP200ST@192.168.233.233:INFO - Amplitude changed to 50.0 %.

2024-09-27 16:23:24,605<14772>:UP200ST@192.168.233.233:INFO - Power changed to 50.0 %.

2024-09-27 16:23:24,630<14772>:UP200ST@192.168.233.233:INFO - Time limit activated.

2024-09-27 16:23:24,657<14772>:UP200ST@192.168.233.233:INFO - Time limit changed to 90.0 s.

2024-09-27 16:23:24,657<14772>:UP200ST@192.168.233.233:INFO - Starting sonication...

2024-09-27 16:23:24,682<14772>:UP200ST@192.168.233.233:INFO - Ultrasound turned on.

2024-09-27 16:24:54,779<14772>:UP200ST@192.168.233.233:INFO - Ultrasound turned off.

2024-09-27 16:24:54,779<14772>:UP200ST@192.168.233.233:INFO - Finished sonication.

2024-09-27 16:24:54,780<13700>:Minerva.API.MinervaAPI:SYNTHESIS_STEP - Sonicate [FALCON_TUBE_50_ML_2363304640128]: 90.0 s; 50 % Amplitude; 50 % Power

2024-09-27 16:24:56,386<20644>:XArm6@192.168.1.204:INFO - Finished moving robot arm to source destination: UP200ST@192.168.233.233.

2024-09-27 16:25:08,917<20644>:XArm6@192.168.1.204:INFO - Finished moving robot arm to target destination: CapperDecapper@ArduinoController@COM25.

2024-09-27 16:25:09,466<23296>:CapperDecapper@ArduinoController@COM25:INFO - Closing Container...

2024-09-27 16:25:09,497<23296>:CapperDecapper@ArduinoController@COM25:INFO - Wrist turning clockwise

2024-09-27 16:25:12,624<23296>:CapperDecapper@ArduinoController@COM25:INFO - Wrist turning clockwise

2024-09-27 16:25:13,210<23296>:CapperDecapper@ArduinoController@COM25:INFO - DC Motor Current [mA]: 237.75

2024-09-27 16:25:13,292<23296>:CapperDecapper@ArduinoController@COM25:INFO - DC Motor Current [mA]: 243.05

2024-09-27 16:25:13,374<23296>:CapperDecapper@ArduinoController@COM25:INFO - DC Motor Current [mA]: 238.52

2024-09-27 16:25:13,456<23296>:CapperDecapper@ArduinoController@COM25:INFO - DC Motor Current [mA]: 232.73

2024-09-27 16:25:13,538<23296>:CapperDecapper@ArduinoController@COM25:INFO - DC Motor Current [mA]: 216.15

2024-09-27 16:25:13,620<23296>:CapperDecapper@ArduinoController@COM25:INFO - DC Motor Current [mA]: 209.80

2024-09-27 16:25:13,702<23296>:CapperDecapper@ArduinoController@COM25:INFO - DC Motor Current [mA]: 201.77

2024-09-27 16:25:13,783<23296>:CapperDecapper@ArduinoController@COM25:INFO - DC Motor Current [mA]: 213.23

2024-09-27 16:25:13,866<23296>:CapperDecapper@ArduinoController@COM25:INFO - DC Motor Current [mA]: 228.98

2024-09-27 16:25:13,947<23296>:CapperDecapper@ArduinoController@COM25:INFO - DC Motor Current [mA]: 239.25

2024-09-27 16:25:14,029<23296>:CapperDecapper@ArduinoController@COM25:INFO - DC Motor Current [mA]: 254.88

2024-09-27 16:25:14,111<23296>:CapperDecapper@ArduinoController@COM25:INFO - DC Motor Current [mA]: 268.52

2024-09-27 16:25:14,193<23296>:CapperDecapper@ArduinoController@COM25:INFO - DC Motor Current [mA]: 254.45

2024-09-27 16:25:14,275<23296>:CapperDecapper@ArduinoController@COM25:INFO - DC Motor Current [mA]: 257.03

2024-09-27 16:25:14,358<23296>:CapperDecapper@ArduinoController@COM25:INFO - DC Motor Current [mA]: 240.52

2024-09-27 16:25:14,440<23296>:CapperDecapper@ArduinoController@COM25:INFO - DC Motor Current [mA]: 221.65

2024-09-27 16:25:14,521<23296>:CapperDecapper@ArduinoController@COM25:INFO - DC Motor Current [mA]: 217.35

2024-09-27 16:25:14,603<23296>:CapperDecapper@ArduinoController@COM25:INFO - DC Motor Current [mA]: 213.02

2024-09-27 16:25:14,685<23296>:CapperDecapper@ArduinoController@COM25:INFO - DC Motor Current [mA]: 222.95

2024-09-27 16:25:14,767<23296>:CapperDecapper@ArduinoController@COM25:INFO - DC Motor Current [mA]: 223.50

2024-09-27 16:25:14,849<23296>:CapperDecapper@ArduinoController@COM25:INFO - DC Motor Current [mA]: 222.10

2024-09-27 16:25:14,931<23296>:CapperDecapper@ArduinoController@COM25:INFO - DC Motor Current [mA]: 219.90

2024-09-27 16:25:15,013<23296>:CapperDecapper@ArduinoController@COM25:INFO - DC Motor Current [mA]: 204.00

2024-09-27 16:25:15,095<23296>:CapperDecapper@ArduinoController@COM25:INFO - DC Motor Current [mA]: 197.88

2024-09-27 16:25:15,177<23296>:CapperDecapper@ArduinoController@COM25:INFO - DC Motor Current [mA]: 201.38

2024-09-27 16:25:15,259<23296>:CapperDecapper@ArduinoController@COM25:INFO - DC Motor Current [mA]: 205.20

2024-09-27 16:25:15,341<23296>:CapperDecapper@ArduinoController@COM25:INFO - DC Motor Current [mA]: 223.75

2024-09-27 16:25:15,423<23296>:CapperDecapper@ArduinoController@COM25:INFO - DC Motor Current [mA]: 232.20

2024-09-27 16:25:15,505<23296>:CapperDecapper@ArduinoController@COM25:INFO - DC Motor Current [mA]: 242.43

2024-09-27 16:25:15,587<23296>:CapperDecapper@ArduinoController@COM25:INFO - DC Motor Current [mA]: 248.05

2024-09-27 16:25:15,669<23296>:CapperDecapper@ArduinoController@COM25:INFO - DC Motor Current [mA]: 236.90

2024-09-27 16:25:15,751<23296>:CapperDecapper@ArduinoController@COM25:INFO - DC Motor Current [mA]: 235.85

2024-09-27 16:25:15,783<23296>:CapperDecapper@ArduinoController@COM25:INFO - Stopped turning wrist

2024-09-27 16:25:17,774<23296>:CapperDecapper@ArduinoController@COM25:INFO - Clamp opened.

2024-09-27 16:25:19,203<20644>:XArm6@192.168.1.204:INFO - Finished moving robot arm to source destination: CapperDecapper@ArduinoController@COM25.

2024-09-27 16:25:47,506<20644>:XArm6@192.168.1.204:INFO - Changed grip on container FALCON_TUBE_50_ML_2363304640128 to a top grip.

2024-09-27 16:26:02,971<20644>:XArm6@192.168.1.204:INFO - Finished moving robot arm to target destination: Isolab_50mL_Foldable_Tube_Rack->deck 1.

2024-09-27 16:26:12,657<20644>:XArm6@192.168.1.204:INFO - Finished moving robot arm to source destination: Isolab_50mL_Foldable_Tube_Rack->deck 1.

2024-09-27 16:26:37,664<20644>:XArm6@192.168.1.204:INFO - Changed grip on container FALCON_TUBE_50_ML_2363304718592 to a sideways grip.

2024-09-27 16:26:43,036<20644>:XArm6@192.168.1.204:INFO - Finished moving robot arm to target destination: UP200ST@192.168.233.233.

2024-09-27 16:26:43,621<14772>:UP200ST@192.168.233.233:INFO - Amplitude changed to 50.0 %.

2024-09-27 16:26:43,649<14772>:UP200ST@192.168.233.233:INFO - Power changed to 50.0 %.

2024-09-27 16:26:43,674<14772>:UP200ST@192.168.233.233:INFO - Time limit activated.

2024-09-27 16:26:43,701<14772>:UP200ST@192.168.233.233:INFO - Time limit changed to 10.0 s.

2024-09-27 16:26:43,701<14772>:UP200ST@192.168.233.233:INFO - Starting sonication...

2024-09-27 16:26:43,728<14772>:UP200ST@192.168.233.233:INFO - Ultrasound turned on.

2024-09-27 16:26:53,772<14772>:UP200ST@192.168.233.233:INFO - Ultrasound turned off.

2024-09-27 16:26:53,772<14772>:UP200ST@192.168.233.233:INFO - Finished sonication.

2024-09-27 16:26:55,510<20644>:XArm6@192.168.1.204:INFO - Finished moving robot arm to source destination: UP200ST@192.168.233.233.

2024-09-27 16:27:15,451<20644>:XArm6@192.168.1.204:INFO - Changed grip on container FALCON_TUBE_50_ML_2363304718592 to a top grip.

2024-09-27 16:27:31,369<20644>:XArm6@192.168.1.204:INFO - Finished moving robot arm to target destination: Isolab_50mL_Foldable_Tube_Rack->deck 1.

2024-09-27 16:27:31,911<13700>:Minerva.API.MinervaAPI:SYNTHESIS_STEP - Sonicate [FALCON_TUBE_50_ML_2363304718592]: 10.0 s; 50 % Amplitude; 50 % Power

2024-09-27 16:27:31,912<13700>:Minerva.API.MinervaAPI:SYNTHESIS_STEP - Remove supernatant and redisperse [FALCON_TUBE_50_ML_2363304640128]: Chemical Ethanol: 15.0 mL

2024-09-27 16:27:35,282<13700>:RobotCen@COM8:INFO - Rotor moved to slot 1.

2024-09-27 16:27:35,328<13700>:RobotCen@COM8:INFO - Opened RobotCen Lid.

2024-09-27 16:27:41,570<20644>:XArm6@192.168.1.204:INFO - Finished moving robot arm to source destination: Isolab_50mL_Foldable_Tube_Rack->deck 1.

2024-09-27 16:28:02,201<20644>:XArm6@192.168.1.204:INFO - Finished moving robot arm to target destination: RobotCen@COM8.

2024-09-27 16:28:10,922<13700>:RobotCen@COM8:INFO - Rotor moved to slot 5.

2024-09-27 16:28:10,969<13700>:RobotCen@COM8:INFO - Opened RobotCen Lid.

2024-09-27 16:28:24,176<20644>:XArm6@192.168.1.204:INFO - Finished moving robot arm to source destination: Isolab_50mL_Foldable_Tube_Rack->deck 1.

2024-09-27 16:28:40,746<20644>:XArm6@192.168.1.204:INFO - Finished moving robot arm to target destination: RobotCen@COM8.

2024-09-27 16:28:47,871<13700>:RobotCen@COM8:INFO - Rotor moved to slot 2.

2024-09-27 16:28:47,919<13700>:RobotCen@COM8:INFO - Opened RobotCen Lid.

2024-09-27 16:29:02,890<20644>:XArm6@192.168.1.204:INFO - Finished moving robot arm to source destination: Isolab_50mL_Foldable_Tube_Rack->deck 1.

2024-09-27 16:29:18,771<20644>:XArm6@192.168.1.204:INFO - Finished moving robot arm to target destination: RobotCen@COM8.

2024-09-27 16:29:27,492<13700>:RobotCen@COM8:INFO - Rotor moved to slot 6.

2024-09-27 16:29:27,539<13700>:RobotCen@COM8:INFO - Opened RobotCen Lid.

2024-09-27 16:29:42,442<20644>:XArm6@192.168.1.204:INFO - Finished moving robot arm to source destination: Isolab_50mL_Foldable_Tube_Rack->deck 1.

2024-09-27 16:29:56,570<20644>:XArm6@192.168.1.204:INFO - Finished moving robot arm to target destination: RobotCen@COM8.

2024-09-27 16:30:04,394<13700>:RobotCen@COM8:INFO - Rotor moved to slot 4.

2024-09-27 16:30:04,442<13700>:RobotCen@COM8:INFO - Opened RobotCen Lid.

2024-09-27 16:30:19,411<20644>:XArm6@192.168.1.204:INFO - Finished moving robot arm to source destination: Isolab_50mL_Foldable_Tube_Rack->deck 1.

2024-09-27 16:30:33,599<20644>:XArm6@192.168.1.204:INFO - Finished moving robot arm to target destination: RobotCen@COM8.

2024-09-27 16:30:44,606<13700>:RobotCen@COM8:INFO - Rotor moved to slot 8.

2024-09-27 16:30:44,652<13700>:RobotCen@COM8:INFO - Opened RobotCen Lid.

2024-09-27 16:31:01,801<20644>:XArm6@192.168.1.204:INFO - Finished moving robot arm to source destination: Isolab_50mL_Foldable_Tube_Rack->deck 1.

2024-09-27 16:31:15,373<20644>:XArm6@192.168.1.204:INFO - Finished moving robot arm to target destination: RobotCen@COM8.

2024-09-27 16:31:18,247<3028>:RobotCen@COM8:INFO - Time set to 1200 seconds

2024-09-27 16:31:18,295<3028>:RobotCen@COM8:INFO - Speed set to 11731 rpm

2024-09-27 16:31:18,343<3028>:RobotCen@COM8:INFO - Temperature set to 25 degrees Celsius

2024-09-27 16:31:21,331<3028>:RobotCen@COM8:INFO - Closed RobotCen Lid.

2024-09-27 16:31:21,363<3028>:RobotCen@COM8:INFO - Centrifugation run started. Time: 1200 seconds; Speed: 11731 rpm; Temperature: 25 degrees Celsius

2024-09-27 16:32:15,350<3028>:RobotCen@COM8:INFO - Centrifugation speed setpoint reached.

2024-09-27 16:53:37,934<3028>:RobotCen@COM8:INFO - Centrifugation run finished.

2024-09-27 16:53:39,948<3028>:RobotCen@COM8:INFO - Homing to first rotor position...

2024-09-27 16:54:08,295<3028>:RobotCen@COM8:INFO - Rotor set to first position 1100

2024-09-27 16:54:08,295<3028>:RobotCen@COM8:INFO - Setting next rotor position...

2024-09-27 16:54:12,672<3028>:RobotCen@COM8:INFO - Bottle number set to 8.

2024-09-27 16:54:19,242<13700>:RobotCen@COM8:INFO - Rotor moved to slot 1.

2024-09-27 16:54:22,243<13700>:RobotCen@COM8:INFO - Opened RobotCen Lid.

2024-09-27 16:54:31,104<20644>:XArm6@192.168.1.204:INFO - Finished moving robot arm to source destination: RobotCen@COM8.

2024-09-27 16:54:47,169<20644>:XArm6@192.168.1.204:INFO - Finished moving robot arm to target destination: Isolab_50mL_Foldable_Tube_Rack->deck 1.

2024-09-27 16:54:55,883<13700>:RobotCen@COM8:INFO - Rotor moved to slot 5.

2024-09-27 16:54:55,930<13700>:RobotCen@COM8:INFO - Opened RobotCen Lid.

2024-09-27 16:55:09,521<20644>:XArm6@192.168.1.204:INFO - Finished moving robot arm to source destination: RobotCen@COM8.

2024-09-27 16:55:25,740<20644>:XArm6@192.168.1.204:INFO - Finished moving robot arm to target destination: Isolab_50mL_Foldable_Tube_Rack->deck 1.

2024-09-27 16:55:32,858<13700>:RobotCen@COM8:INFO - Rotor moved to slot 2.

2024-09-27 16:55:32,905<13700>:RobotCen@COM8:INFO - Opened RobotCen Lid.

2024-09-27 16:55:47,072<20644>:XArm6@192.168.1.204:INFO - Finished moving robot arm to source destination: RobotCen@COM8.

2024-09-27 16:56:04,331<20644>:XArm6@192.168.1.204:INFO - Finished moving robot arm to target destination: Isolab_50mL_Foldable_Tube_Rack->deck 1.

2024-09-27 16:56:13,047<13700>:RobotCen@COM8:INFO - Rotor moved to slot 6.

2024-09-27 16:56:13,093<13700>:RobotCen@COM8:INFO - Opened RobotCen Lid.

2024-09-27 16:56:26,699<20644>:XArm6@192.168.1.204:INFO - Finished moving robot arm to source destination: RobotCen@COM8.

2024-09-27 16:56:43,731<20644>:XArm6@192.168.1.204:INFO - Finished moving robot arm to target destination: Isolab_50mL_Foldable_Tube_Rack->deck 1.

2024-09-27 16:56:49,253<13700>:RobotCen@COM8:INFO - Rotor moved to slot 4.

2024-09-27 16:56:49,300<13700>:RobotCen@COM8:INFO - Opened RobotCen Lid.

2024-09-27 16:57:03,084<20644>:XArm6@192.168.1.204:INFO - Finished moving robot arm to source destination: RobotCen@COM8.

2024-09-27 16:57:19,549<20644>:XArm6@192.168.1.204:INFO - Finished moving robot arm to target destination: Isolab_50mL_Foldable_Tube_Rack->deck 1.

2024-09-27 16:57:28,271<13700>:RobotCen@COM8:INFO - Rotor moved to slot 8.

2024-09-27 16:57:28,319<13700>:RobotCen@COM8:INFO - Opened RobotCen Lid.

2024-09-27 16:57:44,245<20644>:XArm6@192.168.1.204:INFO - Finished moving robot arm to source destination: RobotCen@COM8.

2024-09-27 16:57:58,493<20644>:XArm6@192.168.1.204:INFO - Finished moving robot arm to target destination: Isolab_50mL_Foldable_Tube_Rack->deck 1.

2024-09-27 16:57:59,039<13700>:Minerva.API.MinervaAPI:SYNTHESIS_STEP - Centrifuge [FALCON_TUBE_50_ML_2363304638688]: 1200.0 seconds; 11731.38283132503 rpm; 25.0 degrees Celsius

2024-09-27 16:58:05,493<20644>:XArm6@192.168.1.204:INFO - Finished moving robot arm to source destination: Isolab_50mL_Foldable_Tube_Rack->deck 1.

2024-09-27 16:58:30,741<20644>:XArm6@192.168.1.204:INFO - Changed grip on container FALCON_TUBE_50_ML_2363304638688 to a sideways grip.

2024-09-27 16:58:44,080<20644>:XArm6@192.168.1.204:INFO - Finished moving robot arm to target destination: CapperDecapper@ArduinoController@COM25.

2024-09-27 16:58:44,638<23296>:CapperDecapper@ArduinoController@COM25:INFO - Opening Container...

2024-09-27 16:58:44,736<23296>:CapperDecapper@ArduinoController@COM25:INFO - Clamp opened.

2024-09-27 16:58:49,193<23296>:CapperDecapper@ArduinoController@COM25:INFO - Clamp closed.

2024-09-27 16:58:49,226<23296>:CapperDecapper@ArduinoController@COM25:INFO - Wrist turning counterclockwise

2024-09-27 16:58:51,274<23296>:CapperDecapper@ArduinoController@COM25:INFO - Stopped turning wrist

2024-09-27 16:58:52,629<20644>:XArm6@192.168.1.204:INFO - Finished moving robot arm to source destination: CapperDecapper@ArduinoController@COM25.

2024-09-27 16:59:09,717<20644>:XArm6@192.168.1.204:INFO - Finished moving robot arm to target destination: SwitchingValveVici@COM3.

2024-09-27 16:59:10,295<13700>:SwitchingValveVici@COM3:INFO - Valve SwitchingValveVici@COM3 is currently in position 8 (connected to Outlet).

2024-09-27 16:59:10,295<13700>:SwitchingValveVici@COM3:INFO - Moved valve SwitchingValveVici@COM3 to position 8 (connected to Outlet).

2024-09-27 16:59:10,386<22688>:Aladdin@COM12:INFO - Infusion/withdrawing volume set to 15.0 mL at a rate of 70.0 mL/min

2024-09-27 16:59:23,413<22688>:Aladdin@COM12:INFO - Finished withdrawing 15.0 mL at a rate of 70.0 mL/min

2024-09-27 16:59:23,449<13700>:SwitchingValveVici@COM3:INFO - Valve SwitchingValveVici@COM3 is currently in position 8 (connected to Outlet).

2024-09-27 16:59:23,945<13700>:SwitchingValveVici@COM3:INFO - Moved valve SwitchingValveVici@COM3 to position 3 (connected to Waste_Container).

2024-09-27 16:59:24,035<22688>:Aladdin@COM12:INFO - Infusion/withdrawing volume set to 15.0 mL at a rate of 70.0 mL/min

2024-09-27 16:59:37,127<22688>:Aladdin@COM12:INFO - Finished infusing 15.0 mL at a rate of 70.0 mL/min

2024-09-27 16:59:37,128<13700>:SwitchingValveVici@COM3:INFO - Performing purging step into container Container[Waste_Container]: 2.195 L at SwitchingValveVici@COM3->slot 3...

2024-09-27 16:59:37,163<13700>:SwitchingValveVici@COM3:INFO - Valve SwitchingValveVici@COM3 is currently in position 3 (connected to Waste_Container).

2024-09-27 16:59:37,658<13700>:SwitchingValveVici@COM3:INFO - Moved valve SwitchingValveVici@COM3 to position 8 (connected to Outlet).

2024-09-27 16:59:37,749<22688>:Aladdin@COM12:INFO - Infusion/withdrawing volume set to 10.0 mL at a rate of 70.0 mL/min

2024-09-27 16:59:46,459<22688>:Aladdin@COM12:INFO - Finished withdrawing 10.0 mL at a rate of 70.0 mL/min

2024-09-27 16:59:46,496<13700>:SwitchingValveVici@COM3:INFO - Valve SwitchingValveVici@COM3 is currently in position 8 (connected to Outlet).

2024-09-27 16:59:46,993<13700>:SwitchingValveVici@COM3:INFO - Moved valve SwitchingValveVici@COM3 to position 3 (connected to Waste_Container).

2024-09-27 16:59:47,083<22688>:Aladdin@COM12:INFO - Infusion/withdrawing volume set to 10.0 mL at a rate of 70.0 mL/min

2024-09-27 16:59:55,986<22688>:Aladdin@COM12:INFO - Finished infusing 10.0 mL at a rate of 70.0 mL/min

2024-09-27 16:59:55,988<13700>:SwitchingValveVici@COM3:INFO - Performing addition step for chemical Chemical Ethanol: 15.0 mL (Ethanol_wash -> FALCON_TUBE_50_ML_2363304638688)

2024-09-27 16:59:56,023<13700>:SwitchingValveVici@COM3:INFO - Valve SwitchingValveVici@COM3 is currently in position 3 (connected to Waste_Container).

2024-09-27 16:59:56,182<13700>:SwitchingValveVici@COM3:INFO - Moved valve SwitchingValveVici@COM3 to position 2 (connected to Ethanol_wash).

2024-09-27 16:59:56,273<22688>:Aladdin@COM12:INFO - Infusion/withdrawing volume set to 15.0 mL at a rate of 70.0 mL/min

2024-09-27 17:00:09,348<22688>:Aladdin@COM12:INFO - Finished withdrawing 15.0 mL at a rate of 70.0 mL/min

2024-09-27 17:00:09,386<13700>:SwitchingValveVici@COM3:INFO - Valve SwitchingValveVici@COM3 is currently in position 2 (connected to Ethanol_wash).

2024-09-27 17:00:09,802<13700>:SwitchingValveVici@COM3:INFO - Moved valve SwitchingValveVici@COM3 to position 8 (connected to Outlet).

2024-09-27 17:00:09,892<22688>:Aladdin@COM12:INFO - Infusion/withdrawing volume set to 15.0 mL at a rate of 70.0 mL/min

2024-09-27 17:00:22,950<22688>:Aladdin@COM12:INFO - Finished infusing 15.0 mL at a rate of 70.0 mL/min

2024-09-27 17:00:22,951<13700>:SwitchingValveVici@COM3:INFO - Finished adding Chemical Ethanol: 15.0 mL (Ethanol_wash -> FALCON_TUBE_50_ML_2363304638688)

2024-09-27 17:00:23,964<13700>:SwitchingValveVici@COM3:INFO - Performing purging step into container FALCON_TUBE_50_ML: 15.0 mL at SwitchingValveVici@COM3->slot 0...

2024-09-27 17:00:25,178<13700>:SwitchingValveVici@COM3:INFO - Valve SwitchingValveVici@COM3 is currently in position 8 (connected to Outlet).

2024-09-27 17:00:25,178<13700>:SwitchingValveVici@COM3:INFO - Moved valve SwitchingValveVici@COM3 to position 8 (connected to Outlet).

2024-09-27 17:00:25,267<22688>:Aladdin@COM12:INFO - Infusion/withdrawing volume set to 30.0 mL at a rate of 70.0 mL/min

2024-09-27 17:00:51,317<22688>:Aladdin@COM12:INFO - Finished withdrawing 30.0 mL at a rate of 70.0 mL/min

2024-09-27 17:00:52,581<22688>:Aladdin@COM12:INFO - Infusion/withdrawing volume set to 30.0 mL at a rate of 70.0 mL/min

2024-09-27 17:01:18,539<22688>:Aladdin@COM12:INFO - Finished infusing 30.0 mL at a rate of 70.0 mL/min

2024-09-27 17:01:18,540<13700>:Minerva.API.MinervaAPI:SYNTHESIS_STEP - Add Chemical [FALCON_TUBE_50_ML_2363304638688]: FALCON_TUBE_50_ML; Chemical Ethanol: 15.0 mL; 70.0 mL/min; 50 mL Syringe

2024-09-27 17:01:20,077<20644>:XArm6@192.168.1.204:INFO - Finished moving robot arm to source destination: SwitchingValveVici@COM3.

2024-09-27 17:01:28,939<20644>:XArm6@192.168.1.204:INFO - Finished moving robot arm to target destination: UP200ST@192.168.233.233.

2024-09-27 17:01:29,512<14772>:UP200ST@192.168.233.233:INFO - Amplitude changed to 50.0 %.

2024-09-27 17:01:29,550<14772>:UP200ST@192.168.233.233:INFO - Power changed to 50.0 %.

2024-09-27 17:01:29,574<14772>:UP200ST@192.168.233.233:INFO - Time limit activated.

2024-09-27 17:01:29,600<14772>:UP200ST@192.168.233.233:INFO - Time limit changed to 90.0 s.

2024-09-27 17:01:29,600<14772>:UP200ST@192.168.233.233:INFO - Starting sonication...

2024-09-27 17:01:29,625<14772>:UP200ST@192.168.233.233:INFO - Ultrasound turned on.

2024-09-27 17:02:59,708<14772>:UP200ST@192.168.233.233:INFO - Ultrasound turned off.

2024-09-27 17:02:59,709<14772>:UP200ST@192.168.233.233:INFO - Finished sonication.

2024-09-27 17:02:59,709<13700>:Minerva.API.MinervaAPI:SYNTHESIS_STEP - Sonicate [FALCON_TUBE_50_ML_2363304638688]: 90.0 s; 50 % Amplitude; 50 % Power

2024-09-27 17:03:01,327<20644>:XArm6@192.168.1.204:INFO - Finished moving robot arm to source destination: UP200ST@192.168.233.233.

2024-09-27 17:03:13,847<20644>:XArm6@192.168.1.204:INFO - Finished moving robot arm to target destination: CapperDecapper@ArduinoController@COM25.

2024-09-27 17:03:14,395<23296>:CapperDecapper@ArduinoController@COM25:INFO - Closing Container...

2024-09-27 17:03:14,425<23296>:CapperDecapper@ArduinoController@COM25:INFO - Wrist turning clockwise

2024-09-27 17:03:17,554<23296>:CapperDecapper@ArduinoController@COM25:INFO - Wrist turning clockwise

2024-09-27 17:03:18,148<23296>:CapperDecapper@ArduinoController@COM25:INFO - DC Motor Current [mA]: 243.15

2024-09-27 17:03:18,230<23296>:CapperDecapper@ArduinoController@COM25:INFO - DC Motor Current [mA]: 239.95

2024-09-27 17:03:18,312<23296>:CapperDecapper@ArduinoController@COM25:INFO - DC Motor Current [mA]: 229.30

2024-09-27 17:03:18,394<23296>:CapperDecapper@ArduinoController@COM25:INFO - DC Motor Current [mA]: 228.02

2024-09-27 17:03:18,476<23296>:CapperDecapper@ArduinoController@COM25:INFO - DC Motor Current [mA]: 220.55

2024-09-27 17:03:18,558<23296>:CapperDecapper@ArduinoController@COM25:INFO - DC Motor Current [mA]: 221.00

2024-09-27 17:03:18,640<23296>:CapperDecapper@ArduinoController@COM25:INFO - DC Motor Current [mA]: 231.55

2024-09-27 17:03:18,721<23296>:CapperDecapper@ArduinoController@COM25:INFO - DC Motor Current [mA]: 250.27

2024-09-27 17:03:18,803<23296>:CapperDecapper@ArduinoController@COM25:INFO - DC Motor Current [mA]: 261.58

2024-09-27 17:03:18,885<23296>:CapperDecapper@ArduinoController@COM25:INFO - DC Motor Current [mA]: 269.27

2024-09-27 17:03:18,968<23296>:CapperDecapper@ArduinoController@COM25:INFO - DC Motor Current [mA]: 281.15

2024-09-27 17:03:19,050<23296>:CapperDecapper@ArduinoController@COM25:INFO - DC Motor Current [mA]: 283.92

2024-09-27 17:03:19,132<23296>:CapperDecapper@ArduinoController@COM25:INFO - DC Motor Current [mA]: 271.50

2024-09-27 17:03:19,213<23296>:CapperDecapper@ArduinoController@COM25:INFO - DC Motor Current [mA]: 254.70

2024-09-27 17:03:19,295<23296>:CapperDecapper@ArduinoController@COM25:INFO - DC Motor Current [mA]: 235.82

2024-09-27 17:03:19,377<23296>:CapperDecapper@ArduinoController@COM25:INFO - DC Motor Current [mA]: 220.23

2024-09-27 17:03:19,459<23296>:CapperDecapper@ArduinoController@COM25:INFO - DC Motor Current [mA]: 221.03

2024-09-27 17:03:19,541<23296>:CapperDecapper@ArduinoController@COM25:INFO - DC Motor Current [mA]: 227.40

2024-09-27 17:03:19,623<23296>:CapperDecapper@ArduinoController@COM25:INFO - DC Motor Current [mA]: 227.25

2024-09-27 17:03:19,705<23296>:CapperDecapper@ArduinoController@COM25:INFO - DC Motor Current [mA]: 229.93

2024-09-27 17:03:19,787<23296>:CapperDecapper@ArduinoController@COM25:INFO - DC Motor Current [mA]: 219.80

2024-09-27 17:03:19,869<23296>:CapperDecapper@ArduinoController@COM25:INFO - DC Motor Current [mA]: 212.33

2024-09-27 17:03:19,950<23296>:CapperDecapper@ArduinoController@COM25:INFO - DC Motor Current [mA]: 207.95

2024-09-27 17:03:20,032<23296>:CapperDecapper@ArduinoController@COM25:INFO - DC Motor Current [mA]: 208.95

2024-09-27 17:03:20,114<23296>:CapperDecapper@ArduinoController@COM25:INFO - DC Motor Current [mA]: 218.10

2024-09-27 17:03:20,196<23296>:CapperDecapper@ArduinoController@COM25:INFO - DC Motor Current [mA]: 236.55

2024-09-27 17:03:20,278<23296>:CapperDecapper@ArduinoController@COM25:INFO - DC Motor Current [mA]: 239.47

2024-09-27 17:03:20,360<23296>:CapperDecapper@ArduinoController@COM25:INFO - DC Motor Current [mA]: 252.60

2024-09-27 17:03:20,442<23296>:CapperDecapper@ArduinoController@COM25:INFO - DC Motor Current [mA]: 258.88

2024-09-27 17:03:20,524<23296>:CapperDecapper@ArduinoController@COM25:INFO - DC Motor Current [mA]: 265.20

2024-09-27 17:03:20,607<23296>:CapperDecapper@ArduinoController@COM25:INFO - DC Motor Current [mA]: 242.77

2024-09-27 17:03:20,689<23296>:CapperDecapper@ArduinoController@COM25:INFO - DC Motor Current [mA]: 237.70

2024-09-27 17:03:20,721<23296>:CapperDecapper@ArduinoController@COM25:INFO - Stopped turning wrist

2024-09-27 17:03:22,708<23296>:CapperDecapper@ArduinoController@COM25:INFO - Clamp opened.

2024-09-27 17:03:25,985<20644>:XArm6@192.168.1.204:INFO - Finished moving robot arm to source destination: CapperDecapper@ArduinoController@COM25.

2024-09-27 17:03:53,418<20644>:XArm6@192.168.1.204:INFO - Changed grip on container FALCON_TUBE_50_ML_2363304638688 to a top grip.

2024-09-27 17:04:08,455<20644>:XArm6@192.168.1.204:INFO - Finished moving robot arm to target destination: Isolab_50mL_Foldable_Tube_Rack->deck 1.

2024-09-27 17:04:09,004<13700>:Minerva.API.MinervaAPI:SYNTHESIS_STEP - Remove supernatant and redisperse [FALCON_TUBE_50_ML_2363304638688]: Chemical Ethanol: 15.0 mL

2024-09-27 17:04:17,377<20644>:XArm6@192.168.1.204:INFO - Finished moving robot arm to source destination: Isolab_50mL_Foldable_Tube_Rack->deck 1.

2024-09-27 17:04:42,900<20644>:XArm6@192.168.1.204:INFO - Changed grip on container FALCON_TUBE_50_ML_2363304638976 to a sideways grip.

2024-09-27 17:04:55,731<20644>:XArm6@192.168.1.204:INFO - Finished moving robot arm to target destination: CapperDecapper@ArduinoController@COM25.

2024-09-27 17:04:56,276<23296>:CapperDecapper@ArduinoController@COM25:INFO - Opening Container...

2024-09-27 17:04:56,373<23296>:CapperDecapper@ArduinoController@COM25:INFO - Clamp opened.

2024-09-27 17:05:00,826<23296>:CapperDecapper@ArduinoController@COM25:INFO - Clamp closed.

2024-09-27 17:05:00,858<23296>:CapperDecapper@ArduinoController@COM25:INFO - Wrist turning counterclockwise

2024-09-27 17:05:02,902<23296>:CapperDecapper@ArduinoController@COM25:INFO - Stopped turning wrist

2024-09-27 17:05:04,256<20644>:XArm6@192.168.1.204:INFO - Finished moving robot arm to source destination: CapperDecapper@ArduinoController@COM25.

2024-09-27 17:05:20,757<20644>:XArm6@192.168.1.204:INFO - Finished moving robot arm to target destination: SwitchingValveVici@COM3.

2024-09-27 17:05:21,336<13700>:SwitchingValveVici@COM3:INFO - Valve SwitchingValveVici@COM3 is currently in position 8 (connected to Outlet).

2024-09-27 17:05:21,337<13700>:SwitchingValveVici@COM3:INFO - Moved valve SwitchingValveVici@COM3 to position 8 (connected to Outlet).

2024-09-27 17:05:21,428<22688>:Aladdin@COM12:INFO - Infusion/withdrawing volume set to 15.0 mL at a rate of 70.0 mL/min

2024-09-27 17:05:34,455<22688>:Aladdin@COM12:INFO - Finished withdrawing 15.0 mL at a rate of 70.0 mL/min

2024-09-27 17:05:34,492<13700>:SwitchingValveVici@COM3:INFO - Valve SwitchingValveVici@COM3 is currently in position 8 (connected to Outlet).

2024-09-27 17:05:34,987<13700>:SwitchingValveVici@COM3:INFO - Moved valve SwitchingValveVici@COM3 to position 3 (connected to Waste_Container).

2024-09-27 17:05:35,078<22688>:Aladdin@COM12:INFO - Infusion/withdrawing volume set to 15.0 mL at a rate of 70.0 mL/min

2024-09-27 17:05:48,104<22688>:Aladdin@COM12:INFO - Finished infusing 15.0 mL at a rate of 70.0 mL/min

2024-09-27 17:05:48,105<13700>:SwitchingValveVici@COM3:INFO - Performing purging step into container Container[Waste_Container]: 2.21 L at SwitchingValveVici@COM3->slot 3...

2024-09-27 17:05:48,141<13700>:SwitchingValveVici@COM3:INFO - Valve SwitchingValveVici@COM3 is currently in position 3 (connected to Waste_Container).

2024-09-27 17:05:48,636<13700>:SwitchingValveVici@COM3:INFO - Moved valve SwitchingValveVici@COM3 to position 8 (connected to Outlet).

2024-09-27 17:05:48,726<22688>:Aladdin@COM12:INFO - Infusion/withdrawing volume set to 10.0 mL at a rate of 70.0 mL/min

2024-09-27 17:05:57,614<22688>:Aladdin@COM12:INFO - Finished withdrawing 10.0 mL at a rate of 70.0 mL/min

2024-09-27 17:05:57,651<13700>:SwitchingValveVici@COM3:INFO - Valve SwitchingValveVici@COM3 is currently in position 8 (connected to Outlet).

2024-09-27 17:05:58,148<13700>:SwitchingValveVici@COM3:INFO - Moved valve SwitchingValveVici@COM3 to position 3 (connected to Waste_Container).

2024-09-27 17:05:58,237<22688>:Aladdin@COM12:INFO - Infusion/withdrawing volume set to 10.0 mL at a rate of 70.0 mL/min

2024-09-27 17:06:07,122<22688>:Aladdin@COM12:INFO - Finished infusing 10.0 mL at a rate of 70.0 mL/min

2024-09-27 17:06:07,124<13700>:SwitchingValveVici@COM3:INFO - Performing addition step for chemical Chemical Ethanol: 15.0 mL (Ethanol_wash -> FALCON_TUBE_50_ML_2363304638976)

2024-09-27 17:06:07,162<13700>:SwitchingValveVici@COM3:INFO - Valve SwitchingValveVici@COM3 is currently in position 3 (connected to Waste_Container).

2024-09-27 17:06:07,321<13700>:SwitchingValveVici@COM3:INFO - Moved valve SwitchingValveVici@COM3 to position 2 (connected to Ethanol_wash).

2024-09-27 17:06:07,410<22688>:Aladdin@COM12:INFO - Infusion/withdrawing volume set to 15.0 mL at a rate of 70.0 mL/min

2024-09-27 17:06:20,437<22688>:Aladdin@COM12:INFO - Finished withdrawing 15.0 mL at a rate of 70.0 mL/min

2024-09-27 17:06:20,475<13700>:SwitchingValveVici@COM3:INFO - Valve SwitchingValveVici@COM3 is currently in position 2 (connected to Ethanol_wash).

2024-09-27 17:06:20,891<13700>:SwitchingValveVici@COM3:INFO - Moved valve SwitchingValveVici@COM3 to position 8 (connected to Outlet).

2024-09-27 17:06:20,980<22688>:Aladdin@COM12:INFO - Infusion/withdrawing volume set to 15.0 mL at a rate of 70.0 mL/min

2024-09-27 17:06:34,038<22688>:Aladdin@COM12:INFO - Finished infusing 15.0 mL at a rate of 70.0 mL/min

2024-09-27 17:06:34,039<13700>:SwitchingValveVici@COM3:INFO - Finished adding Chemical Ethanol: 15.0 mL (Ethanol_wash -> FALCON_TUBE_50_ML_2363304638976)

2024-09-27 17:06:35,040<13700>:SwitchingValveVici@COM3:INFO - Performing purging step into container FALCON_TUBE_50_ML: 15.0 mL at SwitchingValveVici@COM3->slot 0...

2024-09-27 17:06:36,252<13700>:SwitchingValveVici@COM3:INFO - Valve SwitchingValveVici@COM3 is currently in position 8 (connected to Outlet).

2024-09-27 17:06:36,252<13700>:SwitchingValveVici@COM3:INFO - Moved valve SwitchingValveVici@COM3 to position 8 (connected to Outlet).

2024-09-27 17:06:36,339<22688>:Aladdin@COM12:INFO - Infusion/withdrawing volume set to 30.0 mL at a rate of 70.0 mL/min

2024-09-27 17:07:02,311<22688>:Aladdin@COM12:INFO - Finished withdrawing 30.0 mL at a rate of 70.0 mL/min

2024-09-27 17:07:03,574<22688>:Aladdin@COM12:INFO - Infusion/withdrawing volume set to 30.0 mL at a rate of 70.0 mL/min

2024-09-27 17:07:29,450<22688>:Aladdin@COM12:INFO - Finished infusing 30.0 mL at a rate of 70.0 mL/min

2024-09-27 17:07:29,450<13700>:Minerva.API.MinervaAPI:SYNTHESIS_STEP - Add Chemical [FALCON_TUBE_50_ML_2363304638976]: FALCON_TUBE_50_ML; Chemical Ethanol: 15.0 mL; 70.0 mL/min; 50 mL Syringe

2024-09-27 17:07:30,942<20644>:XArm6@192.168.1.204:INFO - Finished moving robot arm to source destination: SwitchingValveVici@COM3.

2024-09-27 17:07:39,857<20644>:XArm6@192.168.1.204:INFO - Finished moving robot arm to target destination: UP200ST@192.168.233.233.

2024-09-27 17:07:40,437<14772>:UP200ST@192.168.233.233:INFO - Amplitude changed to 50.0 %.

2024-09-27 17:07:40,465<14772>:UP200ST@192.168.233.233:INFO - Power changed to 50.0 %.

2024-09-27 17:07:40,490<14772>:UP200ST@192.168.233.233:INFO - Time limit activated.

2024-09-27 17:07:40,518<14772>:UP200ST@192.168.233.233:INFO - Time limit changed to 90.0 s.

2024-09-27 17:07:40,518<14772>:UP200ST@192.168.233.233:INFO - Starting sonication...

2024-09-27 17:07:40,543<14772>:UP200ST@192.168.233.233:INFO - Ultrasound turned on.

2024-09-27 17:09:10,602<14772>:UP200ST@192.168.233.233:INFO - Ultrasound turned off.

2024-09-27 17:09:10,603<14772>:UP200ST@192.168.233.233:INFO - Finished sonication.

2024-09-27 17:09:10,603<13700>:Minerva.API.MinervaAPI:SYNTHESIS_STEP - Sonicate [FALCON_TUBE_50_ML_2363304638976]: 90.0 s; 50 % Amplitude; 50 % Power

2024-09-27 17:09:13,794<20644>:XArm6@192.168.1.204:INFO - Finished moving robot arm to source destination: UP200ST@192.168.233.233.

2024-09-27 17:09:25,277<20644>:XArm6@192.168.1.204:INFO - Finished moving robot arm to target destination: CapperDecapper@ArduinoController@COM25.

2024-09-27 17:09:25,841<23296>:CapperDecapper@ArduinoController@COM25:INFO - Closing Container...

2024-09-27 17:09:25,871<23296>:CapperDecapper@ArduinoController@COM25:INFO - Wrist turning clockwise

2024-09-27 17:09:28,998<23296>:CapperDecapper@ArduinoController@COM25:INFO - Wrist turning clockwise

2024-09-27 17:09:29,584<23296>:CapperDecapper@ArduinoController@COM25:INFO - DC Motor Current [mA]: 207.73

2024-09-27 17:09:29,665<23296>:CapperDecapper@ArduinoController@COM25:INFO - DC Motor Current [mA]: 201.20

2024-09-27 17:09:29,747<23296>:CapperDecapper@ArduinoController@COM25:INFO - DC Motor Current [mA]: 206.88

2024-09-27 17:09:29,829<23296>:CapperDecapper@ArduinoController@COM25:INFO - DC Motor Current [mA]: 209.10

2024-09-27 17:09:29,911<23296>:CapperDecapper@ArduinoController@COM25:INFO - DC Motor Current [mA]: 207.07

2024-09-27 17:09:29,993<23296>:CapperDecapper@ArduinoController@COM25:INFO - DC Motor Current [mA]: 214.75

2024-09-27 17:09:30,075<23296>:CapperDecapper@ArduinoController@COM25:INFO - DC Motor Current [mA]: 228.63

2024-09-27 17:09:30,157<23296>:CapperDecapper@ArduinoController@COM25:INFO - DC Motor Current [mA]: 237.93

2024-09-27 17:09:30,239<23296>:CapperDecapper@ArduinoController@COM25:INFO - DC Motor Current [mA]: 236.85

2024-09-27 17:09:30,321<23296>:CapperDecapper@ArduinoController@COM25:INFO - DC Motor Current [mA]: 242.35

2024-09-27 17:09:30,403<23296>:CapperDecapper@ArduinoController@COM25:INFO - DC Motor Current [mA]: 251.05

2024-09-27 17:09:30,484<23296>:CapperDecapper@ArduinoController@COM25:INFO - DC Motor Current [mA]: 250.02

2024-09-27 17:09:30,566<23296>:CapperDecapper@ArduinoController@COM25:INFO - DC Motor Current [mA]: 238.72

2024-09-27 17:09:30,648<23296>:CapperDecapper@ArduinoController@COM25:INFO - DC Motor Current [mA]: 224.03

2024-09-27 17:09:30,730<23296>:CapperDecapper@ArduinoController@COM25:INFO - DC Motor Current [mA]: 210.23

2024-09-27 17:09:30,812<23296>:CapperDecapper@ArduinoController@COM25:INFO - DC Motor Current [mA]: 210.73

2024-09-27 17:09:30,894<23296>:CapperDecapper@ArduinoController@COM25:INFO - DC Motor Current [mA]: 211.40

2024-09-27 17:09:30,976<23296>:CapperDecapper@ArduinoController@COM25:INFO - DC Motor Current [mA]: 205.05

2024-09-27 17:09:31,058<23296>:CapperDecapper@ArduinoController@COM25:INFO - DC Motor Current [mA]: 209.60

2024-09-27 17:09:31,140<23296>:CapperDecapper@ArduinoController@COM25:INFO - DC Motor Current [mA]: 201.10

2024-09-27 17:09:31,222<23296>:CapperDecapper@ArduinoController@COM25:INFO - DC Motor Current [mA]: 202.35

2024-09-27 17:09:31,304<23296>:CapperDecapper@ArduinoController@COM25:INFO - DC Motor Current [mA]: 203.97

2024-09-27 17:09:31,386<23296>:CapperDecapper@ArduinoController@COM25:INFO - DC Motor Current [mA]: 207.25

2024-09-27 17:09:31,468<23296>:CapperDecapper@ArduinoController@COM25:INFO - DC Motor Current [mA]: 212.88

2024-09-27 17:09:31,550<23296>:CapperDecapper@ArduinoController@COM25:INFO - DC Motor Current [mA]: 222.90

2024-09-27 17:09:31,631<23296>:CapperDecapper@ArduinoController@COM25:INFO - DC Motor Current [mA]: 238.60

2024-09-27 17:09:31,713<23296>:CapperDecapper@ArduinoController@COM25:INFO - DC Motor Current [mA]: 237.92

2024-09-27 17:09:31,795<23296>:CapperDecapper@ArduinoController@COM25:INFO - DC Motor Current [mA]: 239.65

2024-09-27 17:09:31,877<23296>:CapperDecapper@ArduinoController@COM25:INFO - DC Motor Current [mA]: 246.90

2024-09-27 17:09:31,959<23296>:CapperDecapper@ArduinoController@COM25:INFO - DC Motor Current [mA]: 242.40

2024-09-27 17:09:32,041<23296>:CapperDecapper@ArduinoController@COM25:INFO - DC Motor Current [mA]: 225.88

2024-09-27 17:09:32,123<23296>:CapperDecapper@ArduinoController@COM25:INFO - DC Motor Current [mA]: 212.75

2024-09-27 17:09:32,156<23296>:CapperDecapper@ArduinoController@COM25:INFO - Stopped turning wrist

2024-09-27 17:09:34,147<23296>:CapperDecapper@ArduinoController@COM25:INFO - Clamp opened.

2024-09-27 17:09:35,573<20644>:XArm6@192.168.1.204:INFO - Finished moving robot arm to source destination: CapperDecapper@ArduinoController@COM25.

2024-09-27 17:10:04,243<20644>:XArm6@192.168.1.204:INFO - Changed grip on container FALCON_TUBE_50_ML_2363304638976 to a top grip.

2024-09-27 17:10:19,370<20644>:XArm6@192.168.1.204:INFO - Finished moving robot arm to target destination: Isolab_50mL_Foldable_Tube_Rack->deck 1.

2024-09-27 17:10:29,060<20644>:XArm6@192.168.1.204:INFO - Finished moving robot arm to source destination: Isolab_50mL_Foldable_Tube_Rack->deck 1.

2024-09-27 17:10:54,667<20644>:XArm6@192.168.1.204:INFO - Changed grip on container FALCON_TUBE_50_ML_2363304718592 to a sideways grip.

2024-09-27 17:11:00,295<20644>:XArm6@192.168.1.204:INFO - Finished moving robot arm to target destination: UP200ST@192.168.233.233.

2024-09-27 17:11:00,877<14772>:UP200ST@192.168.233.233:INFO - Amplitude changed to 50.0 %.

2024-09-27 17:11:00,904<14772>:UP200ST@192.168.233.233:INFO - Power changed to 50.0 %.

2024-09-27 17:11:00,931<14772>:UP200ST@192.168.233.233:INFO - Time limit activated.

2024-09-27 17:11:00,958<14772>:UP200ST@192.168.233.233:INFO - Time limit changed to 10.0 s.

2024-09-27 17:11:00,958<14772>:UP200ST@192.168.233.233:INFO - Starting sonication...

2024-09-27 17:11:00,984<14772>:UP200ST@192.168.233.233:INFO - Ultrasound turned on.

2024-09-27 17:11:11,029<14772>:UP200ST@192.168.233.233:INFO - Ultrasound turned off.

2024-09-27 17:11:11,029<14772>:UP200ST@192.168.233.233:INFO - Finished sonication.

2024-09-27 17:11:12,760<20644>:XArm6@192.168.1.204:INFO - Finished moving robot arm to source destination: UP200ST@192.168.233.233.

2024-09-27 17:11:30,343<13936>:DHT22Sensor@ArduinoController@COM25:INFO - Temperature: 22.30 C, Humidity: 26.30 %

2024-09-27 17:11:32,589<20644>:XArm6@192.168.1.204:INFO - Changed grip on container FALCON_TUBE_50_ML_2363304718592 to a top grip.

2024-09-27 17:11:48,693<20644>:XArm6@192.168.1.204:INFO - Finished moving robot arm to target destination: Isolab_50mL_Foldable_Tube_Rack->deck 1.

2024-09-27 17:11:49,240<13700>:Minerva.API.MinervaAPI:SYNTHESIS_STEP - Sonicate [FALCON_TUBE_50_ML_2363304718592]: 10.0 s; 50 % Amplitude; 50 % Power

2024-09-27 17:11:49,241<13700>:Minerva.API.MinervaAPI:SYNTHESIS_STEP - Remove supernatant and redisperse [FALCON_TUBE_50_ML_2363304638976]: Chemical Ethanol: 15.0 mL

2024-09-27 17:11:57,721<20644>:XArm6@192.168.1.204:INFO - Finished moving robot arm to source destination: Isolab_50mL_Foldable_Tube_Rack->deck 1.

2024-09-27 17:12:23,314<20644>:XArm6@192.168.1.204:INFO - Changed grip on container FALCON_TUBE_50_ML_2363304639264 to a sideways grip.

2024-09-27 17:12:36,119<20644>:XArm6@192.168.1.204:INFO - Finished moving robot arm to target destination: CapperDecapper@ArduinoController@COM25.

2024-09-27 17:12:36,661<23296>:CapperDecapper@ArduinoController@COM25:INFO - Opening Container...

2024-09-27 17:12:36,757<23296>:CapperDecapper@ArduinoController@COM25:INFO - Clamp opened.

2024-09-27 17:12:41,213<23296>:CapperDecapper@ArduinoController@COM25:INFO - Clamp closed.

2024-09-27 17:12:41,246<23296>:CapperDecapper@ArduinoController@COM25:INFO - Wrist turning counterclockwise

2024-09-27 17:12:43,294<23296>:CapperDecapper@ArduinoController@COM25:INFO - Stopped turning wrist

2024-09-27 17:12:44,648<20644>:XArm6@192.168.1.204:INFO - Finished moving robot arm to source destination: CapperDecapper@ArduinoController@COM25.

2024-09-27 17:13:01,154<20644>:XArm6@192.168.1.204:INFO - Finished moving robot arm to target destination: SwitchingValveVici@COM3.

2024-09-27 17:13:01,735<13700>:SwitchingValveVici@COM3:INFO - Valve SwitchingValveVici@COM3 is currently in position 8 (connected to Outlet).

2024-09-27 17:13:01,736<13700>:SwitchingValveVici@COM3:INFO - Moved valve SwitchingValveVici@COM3 to position 8 (connected to Outlet).

2024-09-27 17:13:01,832<22688>:Aladdin@COM12:INFO - Infusion/withdrawing volume set to 15.0 mL at a rate of 70.0 mL/min

2024-09-27 17:13:14,858<22688>:Aladdin@COM12:INFO - Finished withdrawing 15.0 mL at a rate of 70.0 mL/min

2024-09-27 17:13:14,891<13700>:SwitchingValveVici@COM3:INFO - Valve SwitchingValveVici@COM3 is currently in position 8 (connected to Outlet).

2024-09-27 17:13:15,388<13700>:SwitchingValveVici@COM3:INFO - Moved valve SwitchingValveVici@COM3 to position 3 (connected to Waste_Container).

2024-09-27 17:13:15,480<22688>:Aladdin@COM12:INFO - Infusion/withdrawing volume set to 15.0 mL at a rate of 70.0 mL/min

2024-09-27 17:13:28,569<22688>:Aladdin@COM12:INFO - Finished infusing 15.0 mL at a rate of 70.0 mL/min

2024-09-27 17:13:28,570<13700>:SwitchingValveVici@COM3:INFO - Performing purging step into container Container[Waste_Container]: 2.225 L at SwitchingValveVici@COM3->slot 3...

2024-09-27 17:13:28,605<13700>:SwitchingValveVici@COM3:INFO - Valve SwitchingValveVici@COM3 is currently in position 3 (connected to Waste_Container).

2024-09-27 17:13:29,101<13700>:SwitchingValveVici@COM3:INFO - Moved valve SwitchingValveVici@COM3 to position 8 (connected to Outlet).

2024-09-27 17:13:29,194<22688>:Aladdin@COM12:INFO - Infusion/withdrawing volume set to 10.0 mL at a rate of 70.0 mL/min

2024-09-27 17:13:38,064<22688>:Aladdin@COM12:INFO - Finished withdrawing 10.0 mL at a rate of 70.0 mL/min

2024-09-27 17:13:38,098<13700>:SwitchingValveVici@COM3:INFO - Valve SwitchingValveVici@COM3 is currently in position 8 (connected to Outlet).

2024-09-27 17:13:38,594<13700>:SwitchingValveVici@COM3:INFO - Moved valve SwitchingValveVici@COM3 to position 3 (connected to Waste_Container).

2024-09-27 17:13:38,687<22688>:Aladdin@COM12:INFO - Infusion/withdrawing volume set to 10.0 mL at a rate of 70.0 mL/min

2024-09-27 17:13:47,542<22688>:Aladdin@COM12:INFO - Finished infusing 10.0 mL at a rate of 70.0 mL/min

2024-09-27 17:13:47,544<13700>:SwitchingValveVici@COM3:INFO - Performing addition step for chemical Chemical Ethanol: 15.0 mL (Ethanol_wash -> FALCON_TUBE_50_ML_2363304639264)

2024-09-27 17:13:47,577<13700>:SwitchingValveVici@COM3:INFO - Valve SwitchingValveVici@COM3 is currently in position 3 (connected to Waste_Container).

2024-09-27 17:13:47,734<13700>:SwitchingValveVici@COM3:INFO - Moved valve SwitchingValveVici@COM3 to position 2 (connected to Ethanol_wash).

2024-09-27 17:13:47,828<22688>:Aladdin@COM12:INFO - Infusion/withdrawing volume set to 15.0 mL at a rate of 70.0 mL/min

2024-09-27 17:14:00,854<22688>:Aladdin@COM12:INFO - Finished withdrawing 15.0 mL at a rate of 70.0 mL/min

2024-09-27 17:14:00,890<13700>:SwitchingValveVici@COM3:INFO - Valve SwitchingValveVici@COM3 is currently in position 2 (connected to Ethanol_wash).

2024-09-27 17:14:01,304<13700>:SwitchingValveVici@COM3:INFO - Moved valve SwitchingValveVici@COM3 to position 8 (connected to Outlet).

2024-09-27 17:14:01,397<22688>:Aladdin@COM12:INFO - Infusion/withdrawing volume set to 15.0 mL at a rate of 70.0 mL/min

2024-09-27 17:14:14,439<22688>:Aladdin@COM12:INFO - Finished infusing 15.0 mL at a rate of 70.0 mL/min

2024-09-27 17:14:14,439<13700>:SwitchingValveVici@COM3:INFO - Finished adding Chemical Ethanol: 15.0 mL (Ethanol_wash -> FALCON_TUBE_50_ML_2363304639264)

2024-09-27 17:14:15,446<13700>:SwitchingValveVici@COM3:INFO - Performing purging step into container FALCON_TUBE_50_ML: 15.0 mL at SwitchingValveVici@COM3->slot 0...

2024-09-27 17:14:16,651<13700>:SwitchingValveVici@COM3:INFO - Valve SwitchingValveVici@COM3 is currently in position 8 (connected to Outlet).

2024-09-27 17:14:16,651<13700>:SwitchingValveVici@COM3:INFO - Moved valve SwitchingValveVici@COM3 to position 8 (connected to Outlet).

2024-09-27 17:14:16,739<22688>:Aladdin@COM12:INFO - Infusion/withdrawing volume set to 30.0 mL at a rate of 70.0 mL/min

2024-09-27 17:14:42,599<22688>:Aladdin@COM12:INFO - Finished withdrawing 30.0 mL at a rate of 70.0 mL/min

2024-09-27 17:14:43,861<22688>:Aladdin@COM12:INFO - Infusion/withdrawing volume set to 30.0 mL at a rate of 70.0 mL/min

2024-09-27 17:15:09,882<22688>:Aladdin@COM12:INFO - Finished infusing 30.0 mL at a rate of 70.0 mL/min

2024-09-27 17:15:09,883<13700>:Minerva.API.MinervaAPI:SYNTHESIS_STEP - Add Chemical [FALCON_TUBE_50_ML_2363304639264]: FALCON_TUBE_50_ML; Chemical Ethanol: 15.0 mL; 70.0 mL/min; 50 mL Syringe

2024-09-27 17:15:11,431<20644>:XArm6@192.168.1.204:INFO - Finished moving robot arm to source destination: SwitchingValveVici@COM3.

2024-09-27 17:15:20,109<20644>:XArm6@192.168.1.204:INFO - Finished moving robot arm to target destination: UP200ST@192.168.233.233.

2024-09-27 17:15:20,691<14772>:UP200ST@192.168.233.233:INFO - Amplitude changed to 50.0 %.

2024-09-27 17:15:20,720<14772>:UP200ST@192.168.233.233:INFO - Power changed to 50.0 %.

2024-09-27 17:15:20,745<14772>:UP200ST@192.168.233.233:INFO - Time limit activated.

2024-09-27 17:15:20,772<14772>:UP200ST@192.168.233.233:INFO - Time limit changed to 90.0 s.

2024-09-27 17:15:20,772<14772>:UP200ST@192.168.233.233:INFO - Starting sonication...

2024-09-27 17:15:20,798<14772>:UP200ST@192.168.233.233:INFO - Ultrasound turned on.

2024-09-27 17:16:50,858<14772>:UP200ST@192.168.233.233:INFO - Ultrasound turned off.

2024-09-27 17:16:50,859<14772>:UP200ST@192.168.233.233:INFO - Finished sonication.

2024-09-27 17:16:50,859<13700>:Minerva.API.MinervaAPI:SYNTHESIS_STEP - Sonicate [FALCON_TUBE_50_ML_2363304639264]: 90.0 s; 50 % Amplitude; 50 % Power

2024-09-27 17:16:52,476<20644>:XArm6@192.168.1.204:INFO - Finished moving robot arm to source destination: UP200ST@192.168.233.233.

2024-09-27 17:17:04,978<20644>:XArm6@192.168.1.204:INFO - Finished moving robot arm to target destination: CapperDecapper@ArduinoController@COM25.

2024-09-27 17:17:05,522<23296>:CapperDecapper@ArduinoController@COM25:INFO - Closing Container...

2024-09-27 17:17:05,552<23296>:CapperDecapper@ArduinoController@COM25:INFO - Wrist turning clockwise

2024-09-27 17:17:08,684<23296>:CapperDecapper@ArduinoController@COM25:INFO - Wrist turning clockwise

2024-09-27 17:17:09,266<23296>:CapperDecapper@ArduinoController@COM25:INFO - DC Motor Current [mA]: 226.90

2024-09-27 17:17:09,348<23296>:CapperDecapper@ArduinoController@COM25:INFO - DC Motor Current [mA]: 237.35

2024-09-27 17:17:09,430<23296>:CapperDecapper@ArduinoController@COM25:INFO - DC Motor Current [mA]: 241.60

2024-09-27 17:17:09,512<23296>:CapperDecapper@ArduinoController@COM25:INFO - DC Motor Current [mA]: 251.35

2024-09-27 17:17:09,594<23296>:CapperDecapper@ArduinoController@COM25:INFO - DC Motor Current [mA]: 247.38

2024-09-27 17:17:09,676<23296>:CapperDecapper@ArduinoController@COM25:INFO - DC Motor Current [mA]: 250.70

2024-09-27 17:17:09,758<23296>:CapperDecapper@ArduinoController@COM25:INFO - DC Motor Current [mA]: 262.45

2024-09-27 17:17:09,840<23296>:CapperDecapper@ArduinoController@COM25:INFO - DC Motor Current [mA]: 258.90

2024-09-27 17:17:09,922<23296>:CapperDecapper@ArduinoController@COM25:INFO - DC Motor Current [mA]: 266.85

2024-09-27 17:17:10,004<23296>:CapperDecapper@ArduinoController@COM25:INFO - DC Motor Current [mA]: 269.92

2024-09-27 17:17:10,086<23296>:CapperDecapper@ArduinoController@COM25:INFO - DC Motor Current [mA]: 269.07

2024-09-27 17:17:10,168<23296>:CapperDecapper@ArduinoController@COM25:INFO - DC Motor Current [mA]: 266.15

2024-09-27 17:17:10,250<23296>:CapperDecapper@ArduinoController@COM25:INFO - DC Motor Current [mA]: 253.25

2024-09-27 17:17:10,331<23296>:CapperDecapper@ArduinoController@COM25:INFO - DC Motor Current [mA]: 236.98

2024-09-27 17:17:10,413<23296>:CapperDecapper@ArduinoController@COM25:INFO - DC Motor Current [mA]: 231.95

2024-09-27 17:17:10,496<23296>:CapperDecapper@ArduinoController@COM25:INFO - DC Motor Current [mA]: 224.52

2024-09-27 17:17:10,578<23296>:CapperDecapper@ArduinoController@COM25:INFO - DC Motor Current [mA]: 217.22

2024-09-27 17:17:10,659<23296>:CapperDecapper@ArduinoController@COM25:INFO - DC Motor Current [mA]: 217.53

2024-09-27 17:17:10,741<23296>:CapperDecapper@ArduinoController@COM25:INFO - DC Motor Current [mA]: 229.45

2024-09-27 17:17:10,823<23296>:CapperDecapper@ArduinoController@COM25:INFO - DC Motor Current [mA]: 225.35

2024-09-27 17:17:10,904<23296>:CapperDecapper@ArduinoController@COM25:INFO - DC Motor Current [mA]: 223.23

2024-09-27 17:17:10,986<23296>:CapperDecapper@ArduinoController@COM25:INFO - DC Motor Current [mA]: 225.67

2024-09-27 17:17:11,068<23296>:CapperDecapper@ArduinoController@COM25:INFO - DC Motor Current [mA]: 236.20

2024-09-27 17:17:11,150<23296>:CapperDecapper@ArduinoController@COM25:INFO - DC Motor Current [mA]: 240.85

2024-09-27 17:17:11,233<23296>:CapperDecapper@ArduinoController@COM25:INFO - DC Motor Current [mA]: 248.60

2024-09-27 17:17:11,314<23296>:CapperDecapper@ArduinoController@COM25:INFO - DC Motor Current [mA]: 254.90

2024-09-27 17:17:11,396<23296>:CapperDecapper@ArduinoController@COM25:INFO - DC Motor Current [mA]: 258.13

2024-09-27 17:17:11,479<23296>:CapperDecapper@ArduinoController@COM25:INFO - DC Motor Current [mA]: 258.45

2024-09-27 17:17:11,561<23296>:CapperDecapper@ArduinoController@COM25:INFO - DC Motor Current [mA]: 258.00

2024-09-27 17:17:11,643<23296>:CapperDecapper@ArduinoController@COM25:INFO - DC Motor Current [mA]: 246.70

2024-09-27 17:17:11,725<23296>:CapperDecapper@ArduinoController@COM25:INFO - DC Motor Current [mA]: 234.45

2024-09-27 17:17:11,806<23296>:CapperDecapper@ArduinoController@COM25:INFO - DC Motor Current [mA]: 225.98

2024-09-27 17:17:11,839<23296>:CapperDecapper@ArduinoController@COM25:INFO - Stopped turning wrist

2024-09-27 17:17:13,830<23296>:CapperDecapper@ArduinoController@COM25:INFO - Clamp opened.

2024-09-27 17:17:15,251<20644>:XArm6@192.168.1.204:INFO - Finished moving robot arm to source destination: CapperDecapper@ArduinoController@COM25.

2024-09-27 17:17:44,207<20644>:XArm6@192.168.1.204:INFO - Changed grip on container FALCON_TUBE_50_ML_2363304639264 to a top grip.

2024-09-27 17:18:00,227<20644>:XArm6@192.168.1.204:INFO - Finished moving robot arm to target destination: Isolab_50mL_Foldable_Tube_Rack->deck 1.

2024-09-27 17:18:00,772<13700>:Minerva.API.MinervaAPI:SYNTHESIS_STEP - Remove supernatant and redisperse [FALCON_TUBE_50_ML_2363304639264]: Chemical Ethanol: 15.0 mL

2024-09-27 17:18:08,978<20644>:XArm6@192.168.1.204:INFO - Finished moving robot arm to source destination: Isolab_50mL_Foldable_Tube_Rack->deck 1.

2024-09-27 17:18:33,836<20644>:XArm6@192.168.1.204:INFO - Changed grip on container FALCON_TUBE_50_ML_2363304639552 to a sideways grip.

2024-09-27 17:18:46,654<20644>:XArm6@192.168.1.204:INFO - Finished moving robot arm to target destination: CapperDecapper@ArduinoController@COM25.

2024-09-27 17:18:47,203<23296>:CapperDecapper@ArduinoController@COM25:INFO - Opening Container...

2024-09-27 17:18:47,301<23296>:CapperDecapper@ArduinoController@COM25:INFO - Clamp opened.

2024-09-27 17:18:51,748<23296>:CapperDecapper@ArduinoController@COM25:INFO - Clamp closed.

2024-09-27 17:18:51,781<23296>:CapperDecapper@ArduinoController@COM25:INFO - Wrist turning counterclockwise

2024-09-27 17:18:53,821<23296>:CapperDecapper@ArduinoController@COM25:INFO - Stopped turning wrist

2024-09-27 17:18:55,172<20644>:XArm6@192.168.1.204:INFO - Finished moving robot arm to source destination: CapperDecapper@ArduinoController@COM25.

2024-09-27 17:19:11,662<20644>:XArm6@192.168.1.204:INFO - Finished moving robot arm to target destination: SwitchingValveVici@COM3.

2024-09-27 17:19:12,235<13700>:SwitchingValveVici@COM3:INFO - Valve SwitchingValveVici@COM3 is currently in position 8 (connected to Outlet).

2024-09-27 17:19:12,236<13700>:SwitchingValveVici@COM3:INFO - Moved valve SwitchingValveVici@COM3 to position 8 (connected to Outlet).

2024-09-27 17:19:12,318<22688>:Aladdin@COM12:INFO - Infusion/withdrawing volume set to 15.0 mL at a rate of 70.0 mL/min

2024-09-27 17:19:25,377<22688>:Aladdin@COM12:INFO - Finished withdrawing 15.0 mL at a rate of 70.0 mL/min

2024-09-27 17:19:25,418<13700>:SwitchingValveVici@COM3:INFO - Valve SwitchingValveVici@COM3 is currently in position 8 (connected to Outlet).

2024-09-27 17:19:25,914<13700>:SwitchingValveVici@COM3:INFO - Moved valve SwitchingValveVici@COM3 to position 3 (connected to Waste_Container).

2024-09-27 17:19:25,998<22688>:Aladdin@COM12:INFO - Infusion/withdrawing volume set to 15.0 mL at a rate of 70.0 mL/min

2024-09-27 17:19:39,201<22688>:Aladdin@COM12:INFO - Finished infusing 15.0 mL at a rate of 70.0 mL/min

2024-09-27 17:19:39,202<13700>:SwitchingValveVici@COM3:INFO - Performing purging step into container Container[Waste_Container]: 2.24 L at SwitchingValveVici@COM3->slot 3...

2024-09-27 17:19:39,243<13700>:SwitchingValveVici@COM3:INFO - Valve SwitchingValveVici@COM3 is currently in position 3 (connected to Waste_Container).

2024-09-27 17:19:39,740<13700>:SwitchingValveVici@COM3:INFO - Moved valve SwitchingValveVici@COM3 to position 8 (connected to Outlet).

2024-09-27 17:19:39,824<22688>:Aladdin@COM12:INFO - Infusion/withdrawing volume set to 10.0 mL at a rate of 70.0 mL/min

2024-09-27 17:19:48,694<22688>:Aladdin@COM12:INFO - Finished withdrawing 10.0 mL at a rate of 70.0 mL/min

2024-09-27 17:19:48,737<13700>:SwitchingValveVici@COM3:INFO - Valve SwitchingValveVici@COM3 is currently in position 8 (connected to Outlet).

2024-09-27 17:19:49,234<13700>:SwitchingValveVici@COM3:INFO - Moved valve SwitchingValveVici@COM3 to position 3 (connected to Waste_Container).

2024-09-27 17:19:49,316<22688>:Aladdin@COM12:INFO - Infusion/withdrawing volume set to 10.0 mL at a rate of 70.0 mL/min

2024-09-27 17:19:58,219<22688>:Aladdin@COM12:INFO - Finished infusing 10.0 mL at a rate of 70.0 mL/min

2024-09-27 17:19:58,222<13700>:SwitchingValveVici@COM3:INFO - Performing addition step for chemical Chemical Ethanol: 15.0 mL (Ethanol_wash -> FALCON_TUBE_50_ML_2363304639552)

2024-09-27 17:19:58,250<13700>:SwitchingValveVici@COM3:INFO - Valve SwitchingValveVici@COM3 is currently in position 3 (connected to Waste_Container).

2024-09-27 17:19:58,407<13700>:SwitchingValveVici@COM3:INFO - Moved valve SwitchingValveVici@COM3 to position 2 (connected to Ethanol_wash).

2024-09-27 17:19:58,490<22688>:Aladdin@COM12:INFO - Infusion/withdrawing volume set to 15.0 mL at a rate of 70.0 mL/min

2024-09-27 17:20:11,549<22688>:Aladdin@COM12:INFO - Finished withdrawing 15.0 mL at a rate of 70.0 mL/min

2024-09-27 17:20:11,591<13700>:SwitchingValveVici@COM3:INFO - Valve SwitchingValveVici@COM3 is currently in position 2 (connected to Ethanol_wash).

2024-09-27 17:20:12,008<13700>:SwitchingValveVici@COM3:INFO - Moved valve SwitchingValveVici@COM3 to position 8 (connected to Outlet).

2024-09-27 17:20:12,091<22688>:Aladdin@COM12:INFO - Infusion/withdrawing volume set to 15.0 mL at a rate of 70.0 mL/min

2024-09-27 17:20:25,293<22688>:Aladdin@COM12:INFO - Finished infusing 15.0 mL at a rate of 70.0 mL/min

2024-09-27 17:20:25,294<13700>:SwitchingValveVici@COM3:INFO - Finished adding Chemical Ethanol: 15.0 mL (Ethanol_wash -> FALCON_TUBE_50_ML_2363304639552)

2024-09-27 17:20:26,307<13700>:SwitchingValveVici@COM3:INFO - Performing purging step into container FALCON_TUBE_50_ML: 15.0 mL at SwitchingValveVici@COM3->slot 0...

2024-09-27 17:20:27,512<13700>:SwitchingValveVici@COM3:INFO - Valve SwitchingValveVici@COM3 is currently in position 8 (connected to Outlet).

2024-09-27 17:20:27,512<13700>:SwitchingValveVici@COM3:INFO - Moved valve SwitchingValveVici@COM3 to position 8 (connected to Outlet).

2024-09-27 17:20:27,594<22688>:Aladdin@COM12:INFO - Infusion/withdrawing volume set to 30.0 mL at a rate of 70.0 mL/min

2024-09-27 17:20:53,437<22688>:Aladdin@COM12:INFO - Finished withdrawing 30.0 mL at a rate of 70.0 mL/min

2024-09-27 17:20:54,682<22688>:Aladdin@COM12:INFO - Infusion/withdrawing volume set to 30.0 mL at a rate of 70.0 mL/min

2024-09-27 17:21:20,654<22688>:Aladdin@COM12:INFO - Finished infusing 30.0 mL at a rate of 70.0 mL/min

2024-09-27 17:21:20,654<13700>:Minerva.API.MinervaAPI:SYNTHESIS_STEP - Add Chemical [FALCON_TUBE_50_ML_2363304639552]: FALCON_TUBE_50_ML; Chemical Ethanol: 15.0 mL; 70.0 mL/min; 50 mL Syringe

2024-09-27 17:21:22,200<20644>:XArm6@192.168.1.204:INFO - Finished moving robot arm to source destination: SwitchingValveVici@COM3.

2024-09-27 17:21:30,864<20644>:XArm6@192.168.1.204:INFO - Finished moving robot arm to target destination: UP200ST@192.168.233.233.

2024-09-27 17:21:31,447<14772>:UP200ST@192.168.233.233:INFO - Amplitude changed to 50.0 %.

2024-09-27 17:21:31,475<14772>:UP200ST@192.168.233.233:INFO - Power changed to 50.0 %.

2024-09-27 17:21:31,500<14772>:UP200ST@192.168.233.233:INFO - Time limit activated.

2024-09-27 17:21:31,527<14772>:UP200ST@192.168.233.233:INFO - Time limit changed to 90.0 s.

2024-09-27 17:21:31,527<14772>:UP200ST@192.168.233.233:INFO - Starting sonication...

2024-09-27 17:21:31,552<14772>:UP200ST@192.168.233.233:INFO - Ultrasound turned on.

2024-09-27 17:23:01,611<14772>:UP200ST@192.168.233.233:INFO - Ultrasound turned off.

2024-09-27 17:23:01,611<14772>:UP200ST@192.168.233.233:INFO - Finished sonication.

2024-09-27 17:23:01,611<13700>:Minerva.API.MinervaAPI:SYNTHESIS_STEP - Sonicate [FALCON_TUBE_50_ML_2363304639552]: 90.0 s; 50 % Amplitude; 50 % Power

2024-09-27 17:23:03,223<20644>:XArm6@192.168.1.204:INFO - Finished moving robot arm to source destination: UP200ST@192.168.233.233.

2024-09-27 17:23:15,727<20644>:XArm6@192.168.1.204:INFO - Finished moving robot arm to target destination: CapperDecapper@ArduinoController@COM25.

2024-09-27 17:23:16,276<23296>:CapperDecapper@ArduinoController@COM25:INFO - Closing Container...

2024-09-27 17:23:16,306<23296>:CapperDecapper@ArduinoController@COM25:INFO - Wrist turning clockwise

2024-09-27 17:23:19,435<23296>:CapperDecapper@ArduinoController@COM25:INFO - Wrist turning clockwise

2024-09-27 17:23:20,017<23296>:CapperDecapper@ArduinoController@COM25:INFO - DC Motor Current [mA]: 234.98

2024-09-27 17:23:20,099<23296>:CapperDecapper@ArduinoController@COM25:INFO - DC Motor Current [mA]: 223.70

2024-09-27 17:23:20,181<23296>:CapperDecapper@ArduinoController@COM25:INFO - DC Motor Current [mA]: 218.48

2024-09-27 17:23:20,263<23296>:CapperDecapper@ArduinoController@COM25:INFO - DC Motor Current [mA]: 214.90

2024-09-27 17:23:20,345<23296>:CapperDecapper@ArduinoController@COM25:INFO - DC Motor Current [mA]: 208.27

2024-09-27 17:23:20,426<23296>:CapperDecapper@ArduinoController@COM25:INFO - DC Motor Current [mA]: 225.85

2024-09-27 17:23:20,508<23296>:CapperDecapper@ArduinoController@COM25:INFO - DC Motor Current [mA]: 247.95

2024-09-27 17:23:20,590<23296>:CapperDecapper@ArduinoController@COM25:INFO - DC Motor Current [mA]: 253.47

2024-09-27 17:23:20,672<23296>:CapperDecapper@ArduinoController@COM25:INFO - DC Motor Current [mA]: 270.77

2024-09-27 17:23:20,754<23296>:CapperDecapper@ArduinoController@COM25:INFO - DC Motor Current [mA]: 268.92

2024-09-27 17:23:20,836<23296>:CapperDecapper@ArduinoController@COM25:INFO - DC Motor Current [mA]: 268.00

2024-09-27 17:23:20,918<23296>:CapperDecapper@ArduinoController@COM25:INFO - DC Motor Current [mA]: 257.48

2024-09-27 17:23:21,000<23296>:CapperDecapper@ArduinoController@COM25:INFO - DC Motor Current [mA]: 230.40

2024-09-27 17:23:21,082<23296>:CapperDecapper@ArduinoController@COM25:INFO - DC Motor Current [mA]: 203.88

2024-09-27 17:23:21,164<23296>:CapperDecapper@ArduinoController@COM25:INFO - DC Motor Current [mA]: 200.08

2024-09-27 17:23:21,245<23296>:CapperDecapper@ArduinoController@COM25:INFO - DC Motor Current [mA]: 209.98

2024-09-27 17:23:21,328<23296>:CapperDecapper@ArduinoController@COM25:INFO - DC Motor Current [mA]: 218.33

2024-09-27 17:23:21,410<23296>:CapperDecapper@ArduinoController@COM25:INFO - DC Motor Current [mA]: 226.50

2024-09-27 17:23:21,492<23296>:CapperDecapper@ArduinoController@COM25:INFO - DC Motor Current [mA]: 231.10

2024-09-27 17:23:21,574<23296>:CapperDecapper@ArduinoController@COM25:INFO - DC Motor Current [mA]: 216.57

2024-09-27 17:23:21,655<23296>:CapperDecapper@ArduinoController@COM25:INFO - DC Motor Current [mA]: 212.45

2024-09-27 17:23:21,737<23296>:CapperDecapper@ArduinoController@COM25:INFO - DC Motor Current [mA]: 203.18

2024-09-27 17:23:21,820<23296>:CapperDecapper@ArduinoController@COM25:INFO - DC Motor Current [mA]: 208.25

2024-09-27 17:23:21,901<23296>:CapperDecapper@ArduinoController@COM25:INFO - DC Motor Current [mA]: 221.00

2024-09-27 17:23:21,983<23296>:CapperDecapper@ArduinoController@COM25:INFO - DC Motor Current [mA]: 241.63

2024-09-27 17:23:22,065<23296>:CapperDecapper@ArduinoController@COM25:INFO - DC Motor Current [mA]: 258.00

2024-09-27 17:23:22,147<23296>:CapperDecapper@ArduinoController@COM25:INFO - DC Motor Current [mA]: 261.42

2024-09-27 17:23:22,229<23296>:CapperDecapper@ArduinoController@COM25:INFO - DC Motor Current [mA]: 268.73

2024-09-27 17:23:22,311<23296>:CapperDecapper@ArduinoController@COM25:INFO - DC Motor Current [mA]: 259.95

2024-09-27 17:23:22,393<23296>:CapperDecapper@ArduinoController@COM25:INFO - DC Motor Current [mA]: 239.82

2024-09-27 17:23:22,475<23296>:CapperDecapper@ArduinoController@COM25:INFO - DC Motor Current [mA]: 216.98

2024-09-27 17:23:22,557<23296>:CapperDecapper@ArduinoController@COM25:INFO - DC Motor Current [mA]: 198.77

2024-09-27 17:23:22,589<23296>:CapperDecapper@ArduinoController@COM25:INFO - Stopped turning wrist

2024-09-27 17:23:24,576<23296>:CapperDecapper@ArduinoController@COM25:INFO - Clamp opened.

2024-09-27 17:23:25,999<20644>:XArm6@192.168.1.204:INFO - Finished moving robot arm to source destination: CapperDecapper@ArduinoController@COM25.

2024-09-27 17:23:54,422<20644>:XArm6@192.168.1.204:INFO - Changed grip on container FALCON_TUBE_50_ML_2363304639552 to a top grip.

2024-09-27 17:24:09,622<20644>:XArm6@192.168.1.204:INFO - Finished moving robot arm to target destination: Isolab_50mL_Foldable_Tube_Rack->deck 1.

2024-09-27 17:24:19,313<20644>:XArm6@192.168.1.204:INFO - Finished moving robot arm to source destination: Isolab_50mL_Foldable_Tube_Rack->deck 1.

2024-09-27 17:24:44,995<20644>:XArm6@192.168.1.204:INFO - Changed grip on container FALCON_TUBE_50_ML_2363304718592 to a sideways grip.

2024-09-27 17:24:50,625<20644>:XArm6@192.168.1.204:INFO - Finished moving robot arm to target destination: UP200ST@192.168.233.233.

2024-09-27 17:24:51,208<14772>:UP200ST@192.168.233.233:INFO - Amplitude changed to 50.0 %.

2024-09-27 17:24:51,235<14772>:UP200ST@192.168.233.233:INFO - Power changed to 50.0 %.

2024-09-27 17:24:51,260<14772>:UP200ST@192.168.233.233:INFO - Time limit activated.

2024-09-27 17:24:51,286<14772>:UP200ST@192.168.233.233:INFO - Time limit changed to 10.0 s.

2024-09-27 17:24:51,287<14772>:UP200ST@192.168.233.233:INFO - Starting sonication...

2024-09-27 17:24:51,311<14772>:UP200ST@192.168.233.233:INFO - Ultrasound turned on.

2024-09-27 17:25:01,346<14772>:UP200ST@192.168.233.233:INFO - Ultrasound turned off.

2024-09-27 17:25:01,346<14772>:UP200ST@192.168.233.233:INFO - Finished sonication.

2024-09-27 17:25:03,085<20644>:XArm6@192.168.1.204:INFO - Finished moving robot arm to source destination: UP200ST@192.168.233.233.

2024-09-27 17:25:22,709<20644>:XArm6@192.168.1.204:INFO - Changed grip on container FALCON_TUBE_50_ML_2363304718592 to a top grip.

2024-09-27 17:25:38,167<20644>:XArm6@192.168.1.204:INFO - Finished moving robot arm to target destination: Isolab_50mL_Foldable_Tube_Rack->deck 1.

2024-09-27 17:25:38,713<13700>:Minerva.API.MinervaAPI:SYNTHESIS_STEP - Sonicate [FALCON_TUBE_50_ML_2363304718592]: 10.0 s; 50 % Amplitude; 50 % Power

2024-09-27 17:25:38,713<13700>:Minerva.API.MinervaAPI:SYNTHESIS_STEP - Remove supernatant and redisperse [FALCON_TUBE_50_ML_2363304639552]: Chemical Ethanol: 15.0 mL

2024-09-27 17:25:46,947<20644>:XArm6@192.168.1.204:INFO - Finished moving robot arm to source destination: Isolab_50mL_Foldable_Tube_Rack->deck 1.

2024-09-27 17:26:11,811<20644>:XArm6@192.168.1.204:INFO - Changed grip on container FALCON_TUBE_50_ML_2363304639840 to a sideways grip.

2024-09-27 17:26:24,622<20644>:XArm6@192.168.1.204:INFO - Finished moving robot arm to target destination: CapperDecapper@ArduinoController@COM25.

2024-09-27 17:26:25,165<23296>:CapperDecapper@ArduinoController@COM25:INFO - Opening Container...

2024-09-27 17:26:25,263<23296>:CapperDecapper@ArduinoController@COM25:INFO - Clamp opened.

2024-09-27 17:26:29,703<23296>:CapperDecapper@ArduinoController@COM25:INFO - Clamp closed.

2024-09-27 17:26:29,736<23296>:CapperDecapper@ArduinoController@COM25:INFO - Wrist turning counterclockwise

2024-09-27 17:26:31,784<23296>:CapperDecapper@ArduinoController@COM25:INFO - Stopped turning wrist

2024-09-27 17:26:33,145<20644>:XArm6@192.168.1.204:INFO - Finished moving robot arm to source destination: CapperDecapper@ArduinoController@COM25.

2024-09-27 17:26:49,634<20644>:XArm6@192.168.1.204:INFO - Finished moving robot arm to target destination: SwitchingValveVici@COM3.

2024-09-27 17:26:50,212<13700>:SwitchingValveVici@COM3:INFO - Valve SwitchingValveVici@COM3 is currently in position 8 (connected to Outlet).

2024-09-27 17:26:50,213<13700>:SwitchingValveVici@COM3:INFO - Moved valve SwitchingValveVici@COM3 to position 8 (connected to Outlet).

2024-09-27 17:26:50,306<22688>:Aladdin@COM12:INFO - Infusion/withdrawing volume set to 15.0 mL at a rate of 70.0 mL/min

2024-09-27 17:27:03,348<22688>:Aladdin@COM12:INFO - Finished withdrawing 15.0 mL at a rate of 70.0 mL/min

2024-09-27 17:27:03,380<13700>:SwitchingValveVici@COM3:INFO - Valve SwitchingValveVici@COM3 is currently in position 8 (connected to Outlet).

2024-09-27 17:27:03,875<13700>:SwitchingValveVici@COM3:INFO - Moved valve SwitchingValveVici@COM3 to position 3 (connected to Waste_Container).

2024-09-27 17:27:03,970<22688>:Aladdin@COM12:INFO - Infusion/withdrawing volume set to 15.0 mL at a rate of 70.0 mL/min

2024-09-27 17:27:16,995<22688>:Aladdin@COM12:INFO - Finished infusing 15.0 mL at a rate of 70.0 mL/min

2024-09-27 17:27:16,996<13700>:SwitchingValveVici@COM3:INFO - Performing purging step into container Container[Waste_Container]: 2.255 L at SwitchingValveVici@COM3->slot 3...

2024-09-27 17:27:17,030<13700>:SwitchingValveVici@COM3:INFO - Valve SwitchingValveVici@COM3 is currently in position 3 (connected to Waste_Container).

2024-09-27 17:27:17,524<13700>:SwitchingValveVici@COM3:INFO - Moved valve SwitchingValveVici@COM3 to position 8 (connected to Outlet).

2024-09-27 17:27:17,618<22688>:Aladdin@COM12:INFO - Infusion/withdrawing volume set to 10.0 mL at a rate of 70.0 mL/min

2024-09-27 17:27:26,456<22688>:Aladdin@COM12:INFO - Finished withdrawing 10.0 mL at a rate of 70.0 mL/min

2024-09-27 17:27:26,490<13700>:SwitchingValveVici@COM3:INFO - Valve SwitchingValveVici@COM3 is currently in position 8 (connected to Outlet).

2024-09-27 17:27:26,986<13700>:SwitchingValveVici@COM3:INFO - Moved valve SwitchingValveVici@COM3 to position 3 (connected to Waste_Container).

2024-09-27 17:27:27,078<22688>:Aladdin@COM12:INFO - Infusion/withdrawing volume set to 10.0 mL at a rate of 70.0 mL/min

2024-09-27 17:27:35,981<22688>:Aladdin@COM12:INFO - Finished infusing 10.0 mL at a rate of 70.0 mL/min

2024-09-27 17:27:35,983<13700>:SwitchingValveVici@COM3:INFO - Performing addition step for chemical Chemical Ethanol: 15.0 mL (Ethanol_wash -> FALCON_TUBE_50_ML_2363304639840)

2024-09-27 17:27:36,017<13700>:SwitchingValveVici@COM3:INFO - Valve SwitchingValveVici@COM3 is currently in position 3 (connected to Waste_Container).

2024-09-27 17:27:36,174<13700>:SwitchingValveVici@COM3:INFO - Moved valve SwitchingValveVici@COM3 to position 2 (connected to Ethanol_wash).

2024-09-27 17:27:36,268<22688>:Aladdin@COM12:INFO - Infusion/withdrawing volume set to 15.0 mL at a rate of 70.0 mL/min

2024-09-27 17:27:49,293<22688>:Aladdin@COM12:INFO - Finished withdrawing 15.0 mL at a rate of 70.0 mL/min

2024-09-27 17:27:49,328<13700>:SwitchingValveVici@COM3:INFO - Valve SwitchingValveVici@COM3 is currently in position 2 (connected to Ethanol_wash).

2024-09-27 17:27:49,742<13700>:SwitchingValveVici@COM3:INFO - Moved valve SwitchingValveVici@COM3 to position 8 (connected to Outlet).

2024-09-27 17:27:49,837<22688>:Aladdin@COM12:INFO - Infusion/withdrawing volume set to 15.0 mL at a rate of 70.0 mL/min

2024-09-27 17:28:02,877<22688>:Aladdin@COM12:INFO - Finished infusing 15.0 mL at a rate of 70.0 mL/min

2024-09-27 17:28:02,878<13700>:SwitchingValveVici@COM3:INFO - Finished adding Chemical Ethanol: 15.0 mL (Ethanol_wash -> FALCON_TUBE_50_ML_2363304639840)

2024-09-27 17:28:03,891<13700>:SwitchingValveVici@COM3:INFO - Performing purging step into container FALCON_TUBE_50_ML: 15.0 mL at SwitchingValveVici@COM3->slot 0...

2024-09-27 17:28:05,101<13700>:SwitchingValveVici@COM3:INFO - Valve SwitchingValveVici@COM3 is currently in position 8 (connected to Outlet).

2024-09-27 17:28:05,102<13700>:SwitchingValveVici@COM3:INFO - Moved valve SwitchingValveVici@COM3 to position 8 (connected to Outlet).

2024-09-27 17:28:05,195<22688>:Aladdin@COM12:INFO - Infusion/withdrawing volume set to 30.0 mL at a rate of 70.0 mL/min

2024-09-27 17:28:31,246<22688>:Aladdin@COM12:INFO - Finished withdrawing 30.0 mL at a rate of 70.0 mL/min

2024-09-27 17:28:32,507<22688>:Aladdin@COM12:INFO - Infusion/withdrawing volume set to 30.0 mL at a rate of 70.0 mL/min

2024-09-27 17:28:58,557<22688>:Aladdin@COM12:INFO - Finished infusing 30.0 mL at a rate of 70.0 mL/min

2024-09-27 17:28:58,558<13700>:Minerva.API.MinervaAPI:SYNTHESIS_STEP - Add Chemical [FALCON_TUBE_50_ML_2363304639840]: FALCON_TUBE_50_ML; Chemical Ethanol: 15.0 mL; 70.0 mL/min; 50 mL Syringe

2024-09-27 17:29:00,120<20644>:XArm6@192.168.1.204:INFO - Finished moving robot arm to source destination: SwitchingValveVici@COM3.

2024-09-27 17:29:08,780<20644>:XArm6@192.168.1.204:INFO - Finished moving robot arm to target destination: UP200ST@192.168.233.233.

2024-09-27 17:29:09,365<14772>:UP200ST@192.168.233.233:INFO - Amplitude changed to 50.0 %.

2024-09-27 17:29:09,394<14772>:UP200ST@192.168.233.233:INFO - Power changed to 50.0 %.

2024-09-27 17:29:09,419<14772>:UP200ST@192.168.233.233:INFO - Time limit activated.

2024-09-27 17:29:09,446<14772>:UP200ST@192.168.233.233:INFO - Time limit changed to 90.0 s.

2024-09-27 17:29:09,446<14772>:UP200ST@192.168.233.233:INFO - Starting sonication...

2024-09-27 17:29:09,471<14772>:UP200ST@192.168.233.233:INFO - Ultrasound turned on.

2024-09-27 17:30:39,540<14772>:UP200ST@192.168.233.233:INFO - Ultrasound turned off.

2024-09-27 17:30:39,541<14772>:UP200ST@192.168.233.233:INFO - Finished sonication.

2024-09-27 17:30:39,541<13700>:Minerva.API.MinervaAPI:SYNTHESIS_STEP - Sonicate [FALCON_TUBE_50_ML_2363304639840]: 90.0 s; 50 % Amplitude; 50 % Power

2024-09-27 17:30:41,143<20644>:XArm6@192.168.1.204:INFO - Finished moving robot arm to source destination: UP200ST@192.168.233.233.

2024-09-27 17:30:54,200<20644>:XArm6@192.168.1.204:INFO - Finished moving robot arm to target destination: CapperDecapper@ArduinoController@COM25.

2024-09-27 17:30:54,747<23296>:CapperDecapper@ArduinoController@COM25:INFO - Closing Container...

2024-09-27 17:30:54,778<23296>:CapperDecapper@ArduinoController@COM25:INFO - Wrist turning clockwise

2024-09-27 17:30:57,896<23296>:CapperDecapper@ArduinoController@COM25:INFO - Wrist turning clockwise

2024-09-27 17:30:58,480<23296>:CapperDecapper@ArduinoController@COM25:INFO - DC Motor Current [mA]: 219.88

2024-09-27 17:30:58,563<23296>:CapperDecapper@ArduinoController@COM25:INFO - DC Motor Current [mA]: 214.23

2024-09-27 17:30:58,644<23296>:CapperDecapper@ArduinoController@COM25:INFO - DC Motor Current [mA]: 216.17

2024-09-27 17:30:58,726<23296>:CapperDecapper@ArduinoController@COM25:INFO - DC Motor Current [mA]: 205.32

2024-09-27 17:30:58,808<23296>:CapperDecapper@ArduinoController@COM25:INFO - DC Motor Current [mA]: 209.88

2024-09-27 17:30:58,890<23296>:CapperDecapper@ArduinoController@COM25:INFO - DC Motor Current [mA]: 221.15

2024-09-27 17:30:58,972<23296>:CapperDecapper@ArduinoController@COM25:INFO - DC Motor Current [mA]: 241.77

2024-09-27 17:30:59,054<23296>:CapperDecapper@ArduinoController@COM25:INFO - DC Motor Current [mA]: 241.98

2024-09-27 17:30:59,136<23296>:CapperDecapper@ArduinoController@COM25:INFO - DC Motor Current [mA]: 249.40

2024-09-27 17:30:59,217<23296>:CapperDecapper@ArduinoController@COM25:INFO - DC Motor Current [mA]: 251.22

2024-09-27 17:30:59,299<23296>:CapperDecapper@ArduinoController@COM25:INFO - DC Motor Current [mA]: 247.80

2024-09-27 17:30:59,381<23296>:CapperDecapper@ArduinoController@COM25:INFO - DC Motor Current [mA]: 229.55

2024-09-27 17:30:59,464<23296>:CapperDecapper@ArduinoController@COM25:INFO - DC Motor Current [mA]: 211.55

2024-09-27 17:30:59,545<23296>:CapperDecapper@ArduinoController@COM25:INFO - DC Motor Current [mA]: 203.60

2024-09-27 17:30:59,627<23296>:CapperDecapper@ArduinoController@COM25:INFO - DC Motor Current [mA]: 205.33

2024-09-27 17:30:59,709<23296>:CapperDecapper@ArduinoController@COM25:INFO - DC Motor Current [mA]: 210.45

2024-09-27 17:30:59,791<23296>:CapperDecapper@ArduinoController@COM25:INFO - DC Motor Current [mA]: 216.20

2024-09-27 17:30:59,873<23296>:CapperDecapper@ArduinoController@COM25:INFO - DC Motor Current [mA]: 211.63

2024-09-27 17:30:59,955<23296>:CapperDecapper@ArduinoController@COM25:INFO - DC Motor Current [mA]: 217.68

2024-09-27 17:31:00,037<23296>:CapperDecapper@ArduinoController@COM25:INFO - DC Motor Current [mA]: 201.28

2024-09-27 17:31:00,119<23296>:CapperDecapper@ArduinoController@COM25:INFO - DC Motor Current [mA]: 198.35

2024-09-27 17:31:00,201<23296>:CapperDecapper@ArduinoController@COM25:INFO - DC Motor Current [mA]: 203.73

2024-09-27 17:31:00,283<23296>:CapperDecapper@ArduinoController@COM25:INFO - DC Motor Current [mA]: 203.40

2024-09-27 17:31:00,364<23296>:CapperDecapper@ArduinoController@COM25:INFO - DC Motor Current [mA]: 217.13

2024-09-27 17:31:00,446<23296>:CapperDecapper@ArduinoController@COM25:INFO - DC Motor Current [mA]: 233.02

2024-09-27 17:31:00,528<23296>:CapperDecapper@ArduinoController@COM25:INFO - DC Motor Current [mA]: 237.57

2024-09-27 17:31:00,611<23296>:CapperDecapper@ArduinoController@COM25:INFO - DC Motor Current [mA]: 246.02

2024-09-27 17:31:00,693<23296>:CapperDecapper@ArduinoController@COM25:INFO - DC Motor Current [mA]: 245.78

2024-09-27 17:31:00,774<23296>:CapperDecapper@ArduinoController@COM25:INFO - DC Motor Current [mA]: 227.90

2024-09-27 17:31:00,856<23296>:CapperDecapper@ArduinoController@COM25:INFO - DC Motor Current [mA]: 215.00

2024-09-27 17:31:00,938<23296>:CapperDecapper@ArduinoController@COM25:INFO - DC Motor Current [mA]: 200.70

2024-09-27 17:31:01,020<23296>:CapperDecapper@ArduinoController@COM25:INFO - DC Motor Current [mA]: 203.73

2024-09-27 17:31:01,053<23296>:CapperDecapper@ArduinoController@COM25:INFO - Stopped turning wrist

2024-09-27 17:31:03,044<23296>:CapperDecapper@ArduinoController@COM25:INFO - Clamp opened.

2024-09-27 17:31:06,341<20644>:XArm6@192.168.1.204:INFO - Finished moving robot arm to source destination: CapperDecapper@ArduinoController@COM25.

2024-09-27 17:31:33,818<20644>:XArm6@192.168.1.204:INFO - Changed grip on container FALCON_TUBE_50_ML_2363304639840 to a top grip.

2024-09-27 17:31:50,015<20644>:XArm6@192.168.1.204:INFO - Finished moving robot arm to target destination: Isolab_50mL_Foldable_Tube_Rack->deck 1.

2024-09-27 17:31:50,563<13700>:Minerva.API.MinervaAPI:SYNTHESIS_STEP - Remove supernatant and redisperse [FALCON_TUBE_50_ML_2363304639840]: Chemical Ethanol: 15.0 mL

2024-09-27 17:32:00,397<20644>:XArm6@192.168.1.204:INFO - Finished moving robot arm to source destination: Isolab_50mL_Foldable_Tube_Rack->deck 1.

2024-09-27 17:32:24,585<20644>:XArm6@192.168.1.204:INFO - Changed grip on container FALCON_TUBE_50_ML_2363304640128 to a sideways grip.

2024-09-27 17:32:37,377<20644>:XArm6@192.168.1.204:INFO - Finished moving robot arm to target destination: CapperDecapper@ArduinoController@COM25.

2024-09-27 17:32:37,915<23296>:CapperDecapper@ArduinoController@COM25:INFO - Opening Container...

2024-09-27 17:32:38,013<23296>:CapperDecapper@ArduinoController@COM25:INFO - Clamp opened.

2024-09-27 17:32:42,462<23296>:CapperDecapper@ArduinoController@COM25:INFO - Clamp closed.

2024-09-27 17:32:42,495<23296>:CapperDecapper@ArduinoController@COM25:INFO - Wrist turning counterclockwise

2024-09-27 17:32:44,543<23296>:CapperDecapper@ArduinoController@COM25:INFO - Stopped turning wrist

2024-09-27 17:32:45,898<20644>:XArm6@192.168.1.204:INFO - Finished moving robot arm to source destination: CapperDecapper@ArduinoController@COM25.

2024-09-27 17:33:02,938<20644>:XArm6@192.168.1.204:INFO - Finished moving robot arm to target destination: SwitchingValveVici@COM3.

2024-09-27 17:33:03,505<13700>:SwitchingValveVici@COM3:INFO - Valve SwitchingValveVici@COM3 is currently in position 8 (connected to Outlet).

2024-09-27 17:33:03,506<13700>:SwitchingValveVici@COM3:INFO - Moved valve SwitchingValveVici@COM3 to position 8 (connected to Outlet).

2024-09-27 17:33:03,587<22688>:Aladdin@COM12:INFO - Infusion/withdrawing volume set to 15.0 mL at a rate of 70.0 mL/min

2024-09-27 17:33:16,693<22688>:Aladdin@COM12:INFO - Finished withdrawing 15.0 mL at a rate of 70.0 mL/min

2024-09-27 17:33:16,722<13700>:SwitchingValveVici@COM3:INFO - Valve SwitchingValveVici@COM3 is currently in position 8 (connected to Outlet).

2024-09-27 17:33:17,217<13700>:SwitchingValveVici@COM3:INFO - Moved valve SwitchingValveVici@COM3 to position 3 (connected to Waste_Container).

2024-09-27 17:33:17,299<22688>:Aladdin@COM12:INFO - Infusion/withdrawing volume set to 15.0 mL at a rate of 70.0 mL/min

2024-09-27 17:33:30,357<22688>:Aladdin@COM12:INFO - Finished infusing 15.0 mL at a rate of 70.0 mL/min

2024-09-27 17:33:30,358<13700>:SwitchingValveVici@COM3:INFO - Performing purging step into container Container[Waste_Container]: 2.27 L at SwitchingValveVici@COM3->slot 3...

2024-09-27 17:33:30,400<13700>:SwitchingValveVici@COM3:INFO - Valve SwitchingValveVici@COM3 is currently in position 3 (connected to Waste_Container).

2024-09-27 17:33:30,897<13700>:SwitchingValveVici@COM3:INFO - Moved valve SwitchingValveVici@COM3 to position 8 (connected to Outlet).

2024-09-27 17:33:30,980<22688>:Aladdin@COM12:INFO - Infusion/withdrawing volume set to 10.0 mL at a rate of 70.0 mL/min

2024-09-27 17:33:39,866<22688>:Aladdin@COM12:INFO - Finished withdrawing 10.0 mL at a rate of 70.0 mL/min

2024-09-27 17:33:39,897<13700>:SwitchingValveVici@COM3:INFO - Valve SwitchingValveVici@COM3 is currently in position 8 (connected to Outlet).

2024-09-27 17:33:40,392<13700>:SwitchingValveVici@COM3:INFO - Moved valve SwitchingValveVici@COM3 to position 3 (connected to Waste_Container).

2024-09-27 17:33:40,473<22688>:Aladdin@COM12:INFO - Infusion/withdrawing volume set to 10.0 mL at a rate of 70.0 mL/min

2024-09-27 17:33:49,374<22688>:Aladdin@COM12:INFO - Finished infusing 10.0 mL at a rate of 70.0 mL/min

2024-09-27 17:33:49,376<13700>:SwitchingValveVici@COM3:INFO - Performing addition step for chemical Chemical Ethanol: 15.0 mL (Ethanol_wash -> FALCON_TUBE_50_ML_2363304640128)

2024-09-27 17:33:49,417<13700>:SwitchingValveVici@COM3:INFO - Valve SwitchingValveVici@COM3 is currently in position 3 (connected to Waste_Container).

2024-09-27 17:33:49,578<13700>:SwitchingValveVici@COM3:INFO - Moved valve SwitchingValveVici@COM3 to position 2 (connected to Ethanol_wash).

2024-09-27 17:33:49,661<22688>:Aladdin@COM12:INFO - Infusion/withdrawing volume set to 15.0 mL at a rate of 70.0 mL/min

2024-09-27 17:34:02,734<22688>:Aladdin@COM12:INFO - Finished withdrawing 15.0 mL at a rate of 70.0 mL/min

2024-09-27 17:34:02,765<13700>:SwitchingValveVici@COM3:INFO - Valve SwitchingValveVici@COM3 is currently in position 2 (connected to Ethanol_wash).

2024-09-27 17:34:03,179<13700>:SwitchingValveVici@COM3:INFO - Moved valve SwitchingValveVici@COM3 to position 8 (connected to Outlet).

2024-09-27 17:34:03,261<22688>:Aladdin@COM12:INFO - Infusion/withdrawing volume set to 15.0 mL at a rate of 70.0 mL/min

2024-09-27 17:34:16,302<22688>:Aladdin@COM12:INFO - Finished infusing 15.0 mL at a rate of 70.0 mL/min

2024-09-27 17:34:16,303<13700>:SwitchingValveVici@COM3:INFO - Finished adding Chemical Ethanol: 15.0 mL (Ethanol_wash -> FALCON_TUBE_50_ML_2363304640128)

2024-09-27 17:34:17,304<13700>:SwitchingValveVici@COM3:INFO - Performing purging step into container FALCON_TUBE_50_ML: 15.0 mL at SwitchingValveVici@COM3->slot 0...

2024-09-27 17:34:18,507<13700>:SwitchingValveVici@COM3:INFO - Valve SwitchingValveVici@COM3 is currently in position 8 (connected to Outlet).

2024-09-27 17:34:18,508<13700>:SwitchingValveVici@COM3:INFO - Moved valve SwitchingValveVici@COM3 to position 8 (connected to Outlet).

2024-09-27 17:34:18,602<22688>:Aladdin@COM12:INFO - Infusion/withdrawing volume set to 30.0 mL at a rate of 70.0 mL/min

2024-09-27 17:34:44,603<22688>:Aladdin@COM12:INFO - Finished withdrawing 30.0 mL at a rate of 70.0 mL/min

2024-09-27 17:34:45,867<22688>:Aladdin@COM12:INFO - Infusion/withdrawing volume set to 30.0 mL at a rate of 70.0 mL/min

2024-09-27 17:35:11,932<22688>:Aladdin@COM12:INFO - Finished infusing 30.0 mL at a rate of 70.0 mL/min

2024-09-27 17:35:11,932<13700>:Minerva.API.MinervaAPI:SYNTHESIS_STEP - Add Chemical [FALCON_TUBE_50_ML_2363304640128]: FALCON_TUBE_50_ML; Chemical Ethanol: 15.0 mL; 70.0 mL/min; 50 mL Syringe

2024-09-27 17:35:13,423<20644>:XArm6@192.168.1.204:INFO - Finished moving robot arm to source destination: SwitchingValveVici@COM3.

2024-09-27 17:35:22,311<20644>:XArm6@192.168.1.204:INFO - Finished moving robot arm to target destination: UP200ST@192.168.233.233.

2024-09-27 17:35:22,889<14772>:UP200ST@192.168.233.233:INFO - Amplitude changed to 50.0 %.

2024-09-27 17:35:22,917<14772>:UP200ST@192.168.233.233:INFO - Power changed to 50.0 %.

2024-09-27 17:35:22,943<14772>:UP200ST@192.168.233.233:INFO - Time limit activated.

2024-09-27 17:35:22,971<14772>:UP200ST@192.168.233.233:INFO - Time limit changed to 90.0 s.

2024-09-27 17:35:22,971<14772>:UP200ST@192.168.233.233:INFO - Starting sonication...

2024-09-27 17:35:22,996<14772>:UP200ST@192.168.233.233:INFO - Ultrasound turned on.

2024-09-27 17:36:53,045<14772>:UP200ST@192.168.233.233:INFO - Ultrasound turned off.

2024-09-27 17:36:53,045<14772>:UP200ST@192.168.233.233:INFO - Finished sonication.

2024-09-27 17:36:53,046<13700>:Minerva.API.MinervaAPI:SYNTHESIS_STEP - Sonicate [FALCON_TUBE_50_ML_2363304640128]: 90.0 s; 50 % Amplitude; 50 % Power

2024-09-27 17:36:56,253<20644>:XArm6@192.168.1.204:INFO - Finished moving robot arm to source destination: UP200ST@192.168.233.233.

2024-09-27 17:37:07,911<20644>:XArm6@192.168.1.204:INFO - Finished moving robot arm to target destination: CapperDecapper@ArduinoController@COM25.

2024-09-27 17:37:08,457<23296>:CapperDecapper@ArduinoController@COM25:INFO - Closing Container...

2024-09-27 17:37:08,487<23296>:CapperDecapper@ArduinoController@COM25:INFO - Wrist turning clockwise

2024-09-27 17:37:11,607<23296>:CapperDecapper@ArduinoController@COM25:INFO - Wrist turning clockwise

2024-09-27 17:37:12,193<23296>:CapperDecapper@ArduinoController@COM25:INFO - DC Motor Current [mA]: 218.95

2024-09-27 17:37:12,275<23296>:CapperDecapper@ArduinoController@COM25:INFO - DC Motor Current [mA]: 220.42

2024-09-27 17:37:12,356<23296>:CapperDecapper@ArduinoController@COM25:INFO - DC Motor Current [mA]: 227.93

2024-09-27 17:37:12,438<23296>:CapperDecapper@ArduinoController@COM25:INFO - DC Motor Current [mA]: 234.32

2024-09-27 17:37:12,520<23296>:CapperDecapper@ArduinoController@COM25:INFO - DC Motor Current [mA]: 231.68

2024-09-27 17:37:12,602<23296>:CapperDecapper@ArduinoController@COM25:INFO - DC Motor Current [mA]: 233.72

2024-09-27 17:37:12,684<23296>:CapperDecapper@ArduinoController@COM25:INFO - DC Motor Current [mA]: 235.50

2024-09-27 17:37:12,766<23296>:CapperDecapper@ArduinoController@COM25:INFO - DC Motor Current [mA]: 245.30

2024-09-27 17:37:12,848<23296>:CapperDecapper@ArduinoController@COM25:INFO - DC Motor Current [mA]: 257.42

2024-09-27 17:37:12,930<23296>:CapperDecapper@ArduinoController@COM25:INFO - DC Motor Current [mA]: 259.15

2024-09-27 17:37:13,012<23296>:CapperDecapper@ArduinoController@COM25:INFO - DC Motor Current [mA]: 262.75

2024-09-27 17:37:13,094<23296>:CapperDecapper@ArduinoController@COM25:INFO - DC Motor Current [mA]: 252.15

2024-09-27 17:37:13,176<23296>:CapperDecapper@ArduinoController@COM25:INFO - DC Motor Current [mA]: 235.10

2024-09-27 17:37:13,257<23296>:CapperDecapper@ArduinoController@COM25:INFO - DC Motor Current [mA]: 223.52

2024-09-27 17:37:13,341<23296>:CapperDecapper@ArduinoController@COM25:INFO - DC Motor Current [mA]: 218.05

2024-09-27 17:37:13,422<23296>:CapperDecapper@ArduinoController@COM25:INFO - DC Motor Current [mA]: 212.55

2024-09-27 17:37:13,504<23296>:CapperDecapper@ArduinoController@COM25:INFO - DC Motor Current [mA]: 204.15

2024-09-27 17:37:13,586<23296>:CapperDecapper@ArduinoController@COM25:INFO - DC Motor Current [mA]: 210.22

2024-09-27 17:37:13,668<23296>:CapperDecapper@ArduinoController@COM25:INFO - DC Motor Current [mA]: 216.53

2024-09-27 17:37:13,750<23296>:CapperDecapper@ArduinoController@COM25:INFO - DC Motor Current [mA]: 215.93

2024-09-27 17:37:13,832<23296>:CapperDecapper@ArduinoController@COM25:INFO - DC Motor Current [mA]: 221.38

2024-09-27 17:37:13,914<23296>:CapperDecapper@ArduinoController@COM25:INFO - DC Motor Current [mA]: 226.88

2024-09-27 17:37:13,996<23296>:CapperDecapper@ArduinoController@COM25:INFO - DC Motor Current [mA]: 222.42

2024-09-27 17:37:14,078<23296>:CapperDecapper@ArduinoController@COM25:INFO - DC Motor Current [mA]: 227.68

2024-09-27 17:37:14,160<23296>:CapperDecapper@ArduinoController@COM25:INFO - DC Motor Current [mA]: 233.55

2024-09-27 17:37:14,241<23296>:CapperDecapper@ArduinoController@COM25:INFO - DC Motor Current [mA]: 238.88

2024-09-27 17:37:14,323<23296>:CapperDecapper@ArduinoController@COM25:INFO - DC Motor Current [mA]: 251.38

2024-09-27 17:37:14,405<23296>:CapperDecapper@ArduinoController@COM25:INFO - DC Motor Current [mA]: 252.85

2024-09-27 17:37:14,487<23296>:CapperDecapper@ArduinoController@COM25:INFO - DC Motor Current [mA]: 249.02

2024-09-27 17:37:14,569<23296>:CapperDecapper@ArduinoController@COM25:INFO - DC Motor Current [mA]: 247.38

2024-09-27 17:37:14,651<23296>:CapperDecapper@ArduinoController@COM25:INFO - DC Motor Current [mA]: 235.42

2024-09-27 17:37:14,733<23296>:CapperDecapper@ArduinoController@COM25:INFO - DC Motor Current [mA]: 217.30

2024-09-27 17:37:14,765<23296>:CapperDecapper@ArduinoController@COM25:INFO - Stopped turning wrist

2024-09-27 17:37:16,751<23296>:CapperDecapper@ArduinoController@COM25:INFO - Clamp opened.

2024-09-27 17:37:18,170<20644>:XArm6@192.168.1.204:INFO - Finished moving robot arm to source destination: CapperDecapper@ArduinoController@COM25.

2024-09-27 17:37:46,500<20644>:XArm6@192.168.1.204:INFO - Changed grip on container FALCON_TUBE_50_ML_2363304640128 to a top grip.

2024-09-27 17:38:02,517<20644>:XArm6@192.168.1.204:INFO - Finished moving robot arm to target destination: Isolab_50mL_Foldable_Tube_Rack->deck 1.

2024-09-27 17:38:13,155<20644>:XArm6@192.168.1.204:INFO - Finished moving robot arm to source destination: Isolab_50mL_Foldable_Tube_Rack->deck 1.

2024-09-27 17:38:37,190<20644>:XArm6@192.168.1.204:INFO - Changed grip on container FALCON_TUBE_50_ML_2363304718592 to a sideways grip.

2024-09-27 17:38:42,798<20644>:XArm6@192.168.1.204:INFO - Finished moving robot arm to target destination: UP200ST@192.168.233.233.

2024-09-27 17:38:43,376<14772>:UP200ST@192.168.233.233:INFO - Amplitude changed to 50.0 %.

2024-09-27 17:38:43,403<14772>:UP200ST@192.168.233.233:INFO - Power changed to 50.0 %.

2024-09-27 17:38:43,429<14772>:UP200ST@192.168.233.233:INFO - Time limit activated.

2024-09-27 17:38:43,457<14772>:UP200ST@192.168.233.233:INFO - Time limit changed to 10.0 s.

2024-09-27 17:38:43,457<14772>:UP200ST@192.168.233.233:INFO - Starting sonication...

2024-09-27 17:38:43,482<14772>:UP200ST@192.168.233.233:INFO - Ultrasound turned on.

2024-09-27 17:38:53,526<14772>:UP200ST@192.168.233.233:INFO - Ultrasound turned off.

2024-09-27 17:38:53,527<14772>:UP200ST@192.168.233.233:INFO - Finished sonication.

2024-09-27 17:38:55,248<20644>:XArm6@192.168.1.204:INFO - Finished moving robot arm to source destination: UP200ST@192.168.233.233.

2024-09-27 17:39:14,910<20644>:XArm6@192.168.1.204:INFO - Changed grip on container FALCON_TUBE_50_ML_2363304718592 to a top grip.

2024-09-27 17:39:30,903<20644>:XArm6@192.168.1.204:INFO - Finished moving robot arm to target destination: Isolab_50mL_Foldable_Tube_Rack->deck 1.

2024-09-27 17:39:31,447<13700>:Minerva.API.MinervaAPI:SYNTHESIS_STEP - Sonicate [FALCON_TUBE_50_ML_2363304718592]: 10.0 s; 50 % Amplitude; 50 % Power

2024-09-27 17:39:31,449<13700>:Minerva.API.MinervaAPI:SYNTHESIS_STEP - Remove supernatant and redisperse [FALCON_TUBE_50_ML_2363304640128]: Chemical Ethanol: 15.0 mL

2024-09-27 17:39:34,818<13700>:RobotCen@COM8:INFO - Rotor moved to slot 1.

2024-09-27 17:39:34,865<13700>:RobotCen@COM8:INFO - Opened RobotCen Lid.

2024-09-27 17:39:41,099<20644>:XArm6@192.168.1.204:INFO - Finished moving robot arm to source destination: Isolab_50mL_Foldable_Tube_Rack->deck 1.

2024-09-27 17:39:59,083<20644>:XArm6@192.168.1.204:INFO - Finished moving robot arm to target destination: RobotCen@COM8.

2024-09-27 17:40:10,077<13700>:RobotCen@COM8:INFO - Rotor moved to slot 5.

2024-09-27 17:40:10,124<13700>:RobotCen@COM8:INFO - Opened RobotCen Lid.

2024-09-27 17:40:24,796<20644>:XArm6@192.168.1.204:INFO - Finished moving robot arm to source destination: Isolab_50mL_Foldable_Tube_Rack->deck 1.

2024-09-27 17:40:38,178<20644>:XArm6@192.168.1.204:INFO - Finished moving robot arm to target destination: RobotCen@COM8.

2024-09-27 17:40:47,590<13700>:RobotCen@COM8:INFO - Rotor moved to slot 2.

2024-09-27 17:40:47,637<13700>:RobotCen@COM8:INFO - Opened RobotCen Lid.

2024-09-27 17:41:03,117<20644>:XArm6@192.168.1.204:INFO - Finished moving robot arm to source destination: Isolab_50mL_Foldable_Tube_Rack->deck 1.

2024-09-27 17:41:16,105<20644>:XArm6@192.168.1.204:INFO - Finished moving robot arm to target destination: RobotCen@COM8.

2024-09-27 17:41:27,114<13700>:RobotCen@COM8:INFO - Rotor moved to slot 6.

2024-09-27 17:41:27,162<13700>:RobotCen@COM8:INFO - Opened RobotCen Lid.

2024-09-27 17:41:42,579<20644>:XArm6@192.168.1.204:INFO - Finished moving robot arm to source destination: Isolab_50mL_Foldable_Tube_Rack->deck 1.

2024-09-27 17:41:56,853<20644>:XArm6@192.168.1.204:INFO - Finished moving robot arm to target destination: RobotCen@COM8.

2024-09-27 17:42:04,643<13700>:RobotCen@COM8:INFO - Rotor moved to slot 4.

2024-09-27 17:42:04,690<13700>:RobotCen@COM8:INFO - Opened RobotCen Lid.

2024-09-27 17:42:17,750<20644>:XArm6@192.168.1.204:INFO - Finished moving robot arm to source destination: Isolab_50mL_Foldable_Tube_Rack->deck 1.

2024-09-27 17:42:31,544<20644>:XArm6@192.168.1.204:INFO - Finished moving robot arm to target destination: RobotCen@COM8.

2024-09-27 17:42:42,571<13700>:RobotCen@COM8:INFO - Rotor moved to slot 8.

2024-09-27 17:42:42,618<13700>:RobotCen@COM8:INFO - Opened RobotCen Lid.

2024-09-27 17:42:56,949<20644>:XArm6@192.168.1.204:INFO - Finished moving robot arm to source destination: Isolab_50mL_Foldable_Tube_Rack->deck 1.

2024-09-27 17:43:13,702<20644>:XArm6@192.168.1.204:INFO - Finished moving robot arm to target destination: RobotCen@COM8.

2024-09-27 17:43:14,283<3028>:RobotCen@COM8:INFO - Time set to 1200 seconds

2024-09-27 17:43:14,330<3028>:RobotCen@COM8:INFO - Speed set to 11731 rpm

2024-09-27 17:43:14,378<3028>:RobotCen@COM8:INFO - Temperature set to 25 degrees Celsius

2024-09-27 17:43:17,366<3028>:RobotCen@COM8:INFO - Closed RobotCen Lid.

2024-09-27 17:43:17,398<3028>:RobotCen@COM8:INFO - Centrifugation run started. Time: 1200 seconds; Speed: 11731 rpm; Temperature: 25 degrees Celsius

2024-09-27 17:44:09,832<3028>:RobotCen@COM8:INFO - Centrifugation speed setpoint reached.

2024-09-27 18:05:30,906<3028>:RobotCen@COM8:INFO - Centrifugation run finished.

2024-09-27 18:05:32,921<3028>:RobotCen@COM8:INFO - Homing to first rotor position...

2024-09-27 18:06:00,141<3028>:RobotCen@COM8:INFO - Rotor set to first position 1100

2024-09-27 18:06:00,141<3028>:RobotCen@COM8:INFO - Setting next rotor position...

2024-09-27 18:06:04,501<3028>:RobotCen@COM8:INFO - Bottle number set to 8.

2024-09-27 18:06:11,070<13700>:RobotCen@COM8:INFO - Rotor moved to slot 1.

2024-09-27 18:06:14,070<13700>:RobotCen@COM8:INFO - Opened RobotCen Lid.

2024-09-27 18:06:23,197<20644>:XArm6@192.168.1.204:INFO - Finished moving robot arm to source destination: RobotCen@COM8.

2024-09-27 18:06:40,084<20644>:XArm6@192.168.1.204:INFO - Finished moving robot arm to target destination: Isolab_50mL_Foldable_Tube_Rack->deck 1.

2024-09-27 18:06:48,818<13700>:RobotCen@COM8:INFO - Rotor moved to slot 5.

2024-09-27 18:06:48,865<13700>:RobotCen@COM8:INFO - Opened RobotCen Lid.

2024-09-27 18:07:02,496<20644>:XArm6@192.168.1.204:INFO - Finished moving robot arm to source destination: RobotCen@COM8.

2024-09-27 18:07:19,362<20644>:XArm6@192.168.1.204:INFO - Finished moving robot arm to target destination: Isolab_50mL_Foldable_Tube_Rack->deck 1.

2024-09-27 18:07:26,489<13700>:RobotCen@COM8:INFO - Rotor moved to slot 2.

2024-09-27 18:07:26,536<13700>:RobotCen@COM8:INFO - Opened RobotCen Lid.

2024-09-27 18:07:40,401<20644>:XArm6@192.168.1.204:INFO - Finished moving robot arm to source destination: RobotCen@COM8.

2024-09-27 18:07:57,539<20644>:XArm6@192.168.1.204:INFO - Finished moving robot arm to target destination: Isolab_50mL_Foldable_Tube_Rack->deck 1.

2024-09-27 18:08:06,268<13700>:RobotCen@COM8:INFO - Rotor moved to slot 6.

2024-09-27 18:08:06,315<13700>:RobotCen@COM8:INFO - Opened RobotCen Lid.

2024-09-27 18:08:22,928<20644>:XArm6@192.168.1.204:INFO - Finished moving robot arm to source destination: RobotCen@COM8.

2024-09-27 18:08:37,295<20644>:XArm6@192.168.1.204:INFO - Finished moving robot arm to target destination: Isolab_50mL_Foldable_Tube_Rack->deck 1.

2024-09-27 18:08:42,821<13700>:RobotCen@COM8:INFO - Rotor moved to slot 4.

2024-09-27 18:08:42,867<13700>:RobotCen@COM8:INFO - Opened RobotCen Lid.

2024-09-27 18:08:57,099<20644>:XArm6@192.168.1.204:INFO - Finished moving robot arm to source destination: RobotCen@COM8.

2024-09-27 18:09:13,598<20644>:XArm6@192.168.1.204:INFO - Finished moving robot arm to target destination: Isolab_50mL_Foldable_Tube_Rack->deck 1.

2024-09-27 18:09:22,327<13700>:RobotCen@COM8:INFO - Rotor moved to slot 8.

2024-09-27 18:09:22,374<13700>:RobotCen@COM8:INFO - Opened RobotCen Lid.

2024-09-27 18:09:36,282<20644>:XArm6@192.168.1.204:INFO - Finished moving robot arm to source destination: RobotCen@COM8.

2024-09-27 18:09:54,052<20644>:XArm6@192.168.1.204:INFO - Finished moving robot arm to target destination: Isolab_50mL_Foldable_Tube_Rack->deck 1.

2024-09-27 18:09:54,608<13700>:Minerva.API.MinervaAPI:SYNTHESIS_STEP - Centrifuge [FALCON_TUBE_50_ML_2363304638688]: 1200.0 seconds; 11731.38283132503 rpm; 25.0 degrees Celsius

2024-09-27 18:10:01,165<20644>:XArm6@192.168.1.204:INFO - Finished moving robot arm to source destination: Isolab_50mL_Foldable_Tube_Rack->deck 1.

2024-09-27 18:10:26,605<20644>:XArm6@192.168.1.204:INFO - Changed grip on container FALCON_TUBE_50_ML_2363304638688 to a sideways grip.

2024-09-27 18:10:40,117<20644>:XArm6@192.168.1.204:INFO - Finished moving robot arm to target destination: CapperDecapper@ArduinoController@COM25.

2024-09-27 18:10:40,674<23296>:CapperDecapper@ArduinoController@COM25:INFO - Opening Container...

2024-09-27 18:10:40,772<23296>:CapperDecapper@ArduinoController@COM25:INFO - Clamp opened.

2024-09-27 18:10:45,231<23296>:CapperDecapper@ArduinoController@COM25:INFO - Clamp closed.

2024-09-27 18:10:45,264<23296>:CapperDecapper@ArduinoController@COM25:INFO - Wrist turning counterclockwise

2024-09-27 18:10:47,312<23296>:CapperDecapper@ArduinoController@COM25:INFO - Stopped turning wrist

2024-09-27 18:10:48,707<20644>:XArm6@192.168.1.204:INFO - Finished moving robot arm to source destination: CapperDecapper@ArduinoController@COM25.

2024-09-27 18:11:05,352<20644>:XArm6@192.168.1.204:INFO - Finished moving robot arm to target destination: SwitchingValveVici@COM3.

2024-09-27 18:11:05,948<13700>:SwitchingValveVici@COM3:INFO - Valve SwitchingValveVici@COM3 is currently in position 8 (connected to Outlet).

2024-09-27 18:11:05,949<13700>:SwitchingValveVici@COM3:INFO - Moved valve SwitchingValveVici@COM3 to position 8 (connected to Outlet).

2024-09-27 18:11:06,041<22688>:Aladdin@COM12:INFO - Infusion/withdrawing volume set to 15.0 mL at a rate of 70.0 mL/min

2024-09-27 18:11:19,128<22688>:Aladdin@COM12:INFO - Finished withdrawing 15.0 mL at a rate of 70.0 mL/min

2024-09-27 18:11:19,163<13700>:SwitchingValveVici@COM3:INFO - Valve SwitchingValveVici@COM3 is currently in position 8 (connected to Outlet).

2024-09-27 18:11:19,658<13700>:SwitchingValveVici@COM3:INFO - Moved valve SwitchingValveVici@COM3 to position 3 (connected to Waste_Container).

2024-09-27 18:11:19,751<22688>:Aladdin@COM12:INFO - Infusion/withdrawing volume set to 15.0 mL at a rate of 70.0 mL/min

2024-09-27 18:11:31,123<13936>:DHT22Sensor@ArduinoController@COM25:INFO - Temperature: 22.50 C, Humidity: 26.50 %

2024-09-27 18:11:32,919<22688>:Aladdin@COM12:INFO - Finished infusing 15.0 mL at a rate of 70.0 mL/min

2024-09-27 18:11:32,920<13700>:SwitchingValveVici@COM3:INFO - Performing purging step into container Container[Waste_Container]: 2.285 L at SwitchingValveVici@COM3->slot 3...

2024-09-27 18:11:32,955<13700>:SwitchingValveVici@COM3:INFO - Valve SwitchingValveVici@COM3 is currently in position 3 (connected to Waste_Container).

2024-09-27 18:11:33,451<13700>:SwitchingValveVici@COM3:INFO - Moved valve SwitchingValveVici@COM3 to position 8 (connected to Outlet).

2024-09-27 18:11:33,543<22688>:Aladdin@COM12:INFO - Infusion/withdrawing volume set to 10.0 mL at a rate of 70.0 mL/min

2024-09-27 18:11:42,362<22688>:Aladdin@COM12:INFO - Finished withdrawing 10.0 mL at a rate of 70.0 mL/min

2024-09-27 18:11:42,398<13700>:SwitchingValveVici@COM3:INFO - Valve SwitchingValveVici@COM3 is currently in position 8 (connected to Outlet).

2024-09-27 18:11:42,895<13700>:SwitchingValveVici@COM3:INFO - Moved valve SwitchingValveVici@COM3 to position 3 (connected to Waste_Container).

2024-09-27 18:11:42,986<22688>:Aladdin@COM12:INFO - Infusion/withdrawing volume set to 10.0 mL at a rate of 70.0 mL/min

2024-09-27 18:11:51,775<22688>:Aladdin@COM12:INFO - Finished infusing 10.0 mL at a rate of 70.0 mL/min

2024-09-27 18:11:51,777<13700>:SwitchingValveVici@COM3:INFO - Performing addition step for chemical Chemical Ethanol: 15.0 mL (Ethanol_wash -> FALCON_TUBE_50_ML_2363304638688)

2024-09-27 18:11:51,811<13700>:SwitchingValveVici@COM3:INFO - Valve SwitchingValveVici@COM3 is currently in position 3 (connected to Waste_Container).

2024-09-27 18:11:51,970<13700>:SwitchingValveVici@COM3:INFO - Moved valve SwitchingValveVici@COM3 to position 2 (connected to Ethanol_wash).

2024-09-27 18:11:52,062<22688>:Aladdin@COM12:INFO - Infusion/withdrawing volume set to 15.0 mL at a rate of 70.0 mL/min

2024-09-27 18:12:05,054<22688>:Aladdin@COM12:INFO - Finished withdrawing 15.0 mL at a rate of 70.0 mL/min

2024-09-27 18:12:05,091<13700>:SwitchingValveVici@COM3:INFO - Valve SwitchingValveVici@COM3 is currently in position 2 (connected to Ethanol_wash).

2024-09-27 18:12:05,506<13700>:SwitchingValveVici@COM3:INFO - Moved valve SwitchingValveVici@COM3 to position 8 (connected to Outlet).

2024-09-27 18:12:05,598<22688>:Aladdin@COM12:INFO - Infusion/withdrawing volume set to 15.0 mL at a rate of 70.0 mL/min

2024-09-27 18:12:18,798<22688>:Aladdin@COM12:INFO - Finished infusing 15.0 mL at a rate of 70.0 mL/min

2024-09-27 18:12:18,799<13700>:SwitchingValveVici@COM3:INFO - Finished adding Chemical Ethanol: 15.0 mL (Ethanol_wash -> FALCON_TUBE_50_ML_2363304638688)

2024-09-27 18:12:19,806<13700>:SwitchingValveVici@COM3:INFO - Performing purging step into container FALCON_TUBE_50_ML: 15.0 mL at SwitchingValveVici@COM3->slot 0...

2024-09-27 18:12:21,042<13700>:SwitchingValveVici@COM3:INFO - Valve SwitchingValveVici@COM3 is currently in position 8 (connected to Outlet).

2024-09-27 18:12:21,043<13700>:SwitchingValveVici@COM3:INFO - Moved valve SwitchingValveVici@COM3 to position 8 (connected to Outlet).

2024-09-27 18:12:21,130<22688>:Aladdin@COM12:INFO - Infusion/withdrawing volume set to 30.0 mL at a rate of 70.0 mL/min

2024-09-27 18:12:46,986<22688>:Aladdin@COM12:INFO - Finished withdrawing 30.0 mL at a rate of 70.0 mL/min

2024-09-27 18:12:48,263<22688>:Aladdin@COM12:INFO - Infusion/withdrawing volume set to 30.0 mL at a rate of 70.0 mL/min

2024-09-27 18:13:14,183<22688>:Aladdin@COM12:INFO - Finished infusing 30.0 mL at a rate of 70.0 mL/min

2024-09-27 18:13:14,183<13700>:Minerva.API.MinervaAPI:SYNTHESIS_STEP - Add Chemical [FALCON_TUBE_50_ML_2363304638688]: FALCON_TUBE_50_ML; Chemical Ethanol: 15.0 mL; 70.0 mL/min; 50 mL Syringe

2024-09-27 18:13:15,712<20644>:XArm6@192.168.1.204:INFO - Finished moving robot arm to source destination: SwitchingValveVici@COM3.

2024-09-27 18:13:24,418<20644>:XArm6@192.168.1.204:INFO - Finished moving robot arm to target destination: UP200ST@192.168.233.233.

2024-09-27 18:13:25,025<14772>:UP200ST@192.168.233.233:INFO - Amplitude changed to 50.0 %.

2024-09-27 18:13:25,079<14772>:UP200ST@192.168.233.233:INFO - Power changed to 50.0 %.

2024-09-27 18:13:25,124<14772>:UP200ST@192.168.233.233:INFO - Time limit activated.

2024-09-27 18:13:25,170<14772>:UP200ST@192.168.233.233:INFO - Time limit changed to 90.0 s.

2024-09-27 18:13:25,171<14772>:UP200ST@192.168.233.233:INFO - Starting sonication...

2024-09-27 18:13:25,215<14772>:UP200ST@192.168.233.233:INFO - Ultrasound turned on.

2024-09-27 18:14:55,258<14772>:UP200ST@192.168.233.233:INFO - Ultrasound turned off.

2024-09-27 18:14:55,258<14772>:UP200ST@192.168.233.233:INFO - Finished sonication.

2024-09-27 18:14:55,259<13700>:Minerva.API.MinervaAPI:SYNTHESIS_STEP - Sonicate [FALCON_TUBE_50_ML_2363304638688]: 90.0 s; 50 % Amplitude; 50 % Power

2024-09-27 18:14:56,849<20644>:XArm6@192.168.1.204:INFO - Finished moving robot arm to source destination: UP200ST@192.168.233.233.

2024-09-27 18:15:09,990<20644>:XArm6@192.168.1.204:INFO - Finished moving robot arm to target destination: CapperDecapper@ArduinoController@COM25.

2024-09-27 18:15:10,545<23296>:CapperDecapper@ArduinoController@COM25:INFO - Closing Container...

2024-09-27 18:15:10,574<23296>:CapperDecapper@ArduinoController@COM25:INFO - Wrist turning clockwise

2024-09-27 18:15:13,666<23296>:CapperDecapper@ArduinoController@COM25:INFO - Wrist turning clockwise

2024-09-27 18:15:14,252<23296>:CapperDecapper@ArduinoController@COM25:INFO - DC Motor Current [mA]: 208.93

2024-09-27 18:15:14,334<23296>:CapperDecapper@ArduinoController@COM25:INFO - DC Motor Current [mA]: 224.15

2024-09-27 18:15:14,416<23296>:CapperDecapper@ArduinoController@COM25:INFO - DC Motor Current [mA]: 233.93

2024-09-27 18:15:14,498<23296>:CapperDecapper@ArduinoController@COM25:INFO - DC Motor Current [mA]: 240.80

2024-09-27 18:15:14,579<23296>:CapperDecapper@ArduinoController@COM25:INFO - DC Motor Current [mA]: 244.67

2024-09-27 18:15:14,661<23296>:CapperDecapper@ArduinoController@COM25:INFO - DC Motor Current [mA]: 239.80

2024-09-27 18:15:14,743<23296>:CapperDecapper@ArduinoController@COM25:INFO - DC Motor Current [mA]: 245.55

2024-09-27 18:15:14,825<23296>:CapperDecapper@ArduinoController@COM25:INFO - DC Motor Current [mA]: 255.80

2024-09-27 18:15:14,907<23296>:CapperDecapper@ArduinoController@COM25:INFO - DC Motor Current [mA]: 257.52

2024-09-27 18:15:14,989<23296>:CapperDecapper@ArduinoController@COM25:INFO - DC Motor Current [mA]: 257.27

2024-09-27 18:15:15,071<23296>:CapperDecapper@ArduinoController@COM25:INFO - DC Motor Current [mA]: 259.57

2024-09-27 18:15:15,153<23296>:CapperDecapper@ArduinoController@COM25:INFO - DC Motor Current [mA]: 254.00

2024-09-27 18:15:15,235<23296>:CapperDecapper@ArduinoController@COM25:INFO - DC Motor Current [mA]: 244.35

2024-09-27 18:15:15,317<23296>:CapperDecapper@ArduinoController@COM25:INFO - DC Motor Current [mA]: 236.20

2024-09-27 18:15:15,399<23296>:CapperDecapper@ArduinoController@COM25:INFO - DC Motor Current [mA]: 221.65

2024-09-27 18:15:15,481<23296>:CapperDecapper@ArduinoController@COM25:INFO - DC Motor Current [mA]: 210.18

2024-09-27 18:15:15,563<23296>:CapperDecapper@ArduinoController@COM25:INFO - DC Motor Current [mA]: 213.67

2024-09-27 18:15:15,645<23296>:CapperDecapper@ArduinoController@COM25:INFO - DC Motor Current [mA]: 208.90

2024-09-27 18:15:15,727<23296>:CapperDecapper@ArduinoController@COM25:INFO - DC Motor Current [mA]: 210.52

2024-09-27 18:15:15,808<23296>:CapperDecapper@ArduinoController@COM25:INFO - DC Motor Current [mA]: 216.15

2024-09-27 18:15:15,890<23296>:CapperDecapper@ArduinoController@COM25:INFO - DC Motor Current [mA]: 226.92

2024-09-27 18:15:15,972<23296>:CapperDecapper@ArduinoController@COM25:INFO - DC Motor Current [mA]: 231.63

2024-09-27 18:15:16,054<23296>:CapperDecapper@ArduinoController@COM25:INFO - DC Motor Current [mA]: 233.08

2024-09-27 18:15:16,136<23296>:CapperDecapper@ArduinoController@COM25:INFO - DC Motor Current [mA]: 232.35

2024-09-27 18:15:16,218<23296>:CapperDecapper@ArduinoController@COM25:INFO - DC Motor Current [mA]: 238.82

2024-09-27 18:15:16,300<23296>:CapperDecapper@ArduinoController@COM25:INFO - DC Motor Current [mA]: 244.40

2024-09-27 18:15:16,382<23296>:CapperDecapper@ArduinoController@COM25:INFO - DC Motor Current [mA]: 252.73

2024-09-27 18:15:16,464<23296>:CapperDecapper@ArduinoController@COM25:INFO - DC Motor Current [mA]: 259.63

2024-09-27 18:15:16,546<23296>:CapperDecapper@ArduinoController@COM25:INFO - DC Motor Current [mA]: 257.67

2024-09-27 18:15:16,628<23296>:CapperDecapper@ArduinoController@COM25:INFO - DC Motor Current [mA]: 242.40

2024-09-27 18:15:16,710<23296>:CapperDecapper@ArduinoController@COM25:INFO - DC Motor Current [mA]: 234.70

2024-09-27 18:15:16,792<23296>:CapperDecapper@ArduinoController@COM25:INFO - DC Motor Current [mA]: 222.82

2024-09-27 18:15:16,824<23296>:CapperDecapper@ArduinoController@COM25:INFO - Stopped turning wrist

2024-09-27 18:15:18,815<23296>:CapperDecapper@ArduinoController@COM25:INFO - Clamp opened.

2024-09-27 18:15:20,218<20644>:XArm6@192.168.1.204:INFO - Finished moving robot arm to source destination: CapperDecapper@ArduinoController@COM25.

2024-09-27 18:15:48,621<20644>:XArm6@192.168.1.204:INFO - Changed grip on container FALCON_TUBE_50_ML_2363304638688 to a top grip.

2024-09-27 18:16:05,180<20644>:XArm6@192.168.1.204:INFO - Finished moving robot arm to target destination: Isolab_50mL_Foldable_Tube_Rack->deck 1.

2024-09-27 18:16:05,738<13700>:Minerva.API.MinervaAPI:SYNTHESIS_STEP - Remove supernatant and redisperse [FALCON_TUBE_50_ML_2363304638688]: Chemical Ethanol: 15.0 mL

2024-09-27 18:16:14,251<20644>:XArm6@192.168.1.204:INFO - Finished moving robot arm to source destination: Isolab_50mL_Foldable_Tube_Rack->deck 1.

2024-09-27 18:16:39,958<20644>:XArm6@192.168.1.204:INFO - Changed grip on container FALCON_TUBE_50_ML_2363304638976 to a sideways grip.

2024-09-27 18:16:52,919<20644>:XArm6@192.168.1.204:INFO - Finished moving robot arm to target destination: CapperDecapper@ArduinoController@COM25.

2024-09-27 18:16:53,474<23296>:CapperDecapper@ArduinoController@COM25:INFO - Opening Container...

2024-09-27 18:16:53,571<23296>:CapperDecapper@ArduinoController@COM25:INFO - Clamp opened.

2024-09-27 18:16:58,035<23296>:CapperDecapper@ArduinoController@COM25:INFO - Clamp closed.

2024-09-27 18:16:58,068<23296>:CapperDecapper@ArduinoController@COM25:INFO - Wrist turning counterclockwise

2024-09-27 18:17:00,112<23296>:CapperDecapper@ArduinoController@COM25:INFO - Stopped turning wrist

2024-09-27 18:17:01,510<20644>:XArm6@192.168.1.204:INFO - Finished moving robot arm to source destination: CapperDecapper@ArduinoController@COM25.

2024-09-27 18:17:18,098<20644>:XArm6@192.168.1.204:INFO - Finished moving robot arm to target destination: SwitchingValveVici@COM3.

2024-09-27 18:17:18,687<13700>:SwitchingValveVici@COM3:INFO - Valve SwitchingValveVici@COM3 is currently in position 8 (connected to Outlet).

2024-09-27 18:17:18,687<13700>:SwitchingValveVici@COM3:INFO - Moved valve SwitchingValveVici@COM3 to position 8 (connected to Outlet).

2024-09-27 18:17:18,772<22688>:Aladdin@COM12:INFO - Infusion/withdrawing volume set to 15.0 mL at a rate of 70.0 mL/min

2024-09-27 18:17:31,907<22688>:Aladdin@COM12:INFO - Finished withdrawing 15.0 mL at a rate of 70.0 mL/min

2024-09-27 18:17:31,936<13700>:SwitchingValveVici@COM3:INFO - Valve SwitchingValveVici@COM3 is currently in position 8 (connected to Outlet).

2024-09-27 18:17:32,432<13700>:SwitchingValveVici@COM3:INFO - Moved valve SwitchingValveVici@COM3 to position 3 (connected to Waste_Container).

2024-09-27 18:17:32,514<22688>:Aladdin@COM12:INFO - Infusion/withdrawing volume set to 15.0 mL at a rate of 70.0 mL/min

2024-09-27 18:17:45,521<22688>:Aladdin@COM12:INFO - Finished infusing 15.0 mL at a rate of 70.0 mL/min

2024-09-27 18:17:45,522<13700>:SwitchingValveVici@COM3:INFO - Performing purging step into container Container[Waste_Container]: 2.3 L at SwitchingValveVici@COM3->slot 3...

2024-09-27 18:17:45,552<13700>:SwitchingValveVici@COM3:INFO - Valve SwitchingValveVici@COM3 is currently in position 3 (connected to Waste_Container).

2024-09-27 18:17:46,047<13700>:SwitchingValveVici@COM3:INFO - Moved valve SwitchingValveVici@COM3 to position 8 (connected to Outlet).

2024-09-27 18:17:46,128<22688>:Aladdin@COM12:INFO - Infusion/withdrawing volume set to 10.0 mL at a rate of 70.0 mL/min

2024-09-27 18:17:55,030<22688>:Aladdin@COM12:INFO - Finished withdrawing 10.0 mL at a rate of 70.0 mL/min

2024-09-27 18:17:55,062<13700>:SwitchingValveVici@COM3:INFO - Valve SwitchingValveVici@COM3 is currently in position 8 (connected to Outlet).

2024-09-27 18:17:55,557<13700>:SwitchingValveVici@COM3:INFO - Moved valve SwitchingValveVici@COM3 to position 3 (connected to Waste_Container).

2024-09-27 18:17:55,636<22688>:Aladdin@COM12:INFO - Infusion/withdrawing volume set to 10.0 mL at a rate of 70.0 mL/min

2024-09-27 18:18:04,443<22688>:Aladdin@COM12:INFO - Finished infusing 10.0 mL at a rate of 70.0 mL/min

2024-09-27 18:18:04,445<13700>:SwitchingValveVici@COM3:INFO - Performing addition step for chemical Chemical Ethanol: 15.0 mL (Ethanol_wash -> FALCON_TUBE_50_ML_2363304638976)

2024-09-27 18:18:04,475<13700>:SwitchingValveVici@COM3:INFO - Valve SwitchingValveVici@COM3 is currently in position 3 (connected to Waste_Container).

2024-09-27 18:18:04,632<13700>:SwitchingValveVici@COM3:INFO - Moved valve SwitchingValveVici@COM3 to position 2 (connected to Ethanol_wash).

2024-09-27 18:18:04,714<22688>:Aladdin@COM12:INFO - Infusion/withdrawing volume set to 15.0 mL at a rate of 70.0 mL/min

2024-09-27 18:18:17,770<22688>:Aladdin@COM12:INFO - Finished withdrawing 15.0 mL at a rate of 70.0 mL/min

2024-09-27 18:18:17,802<13700>:SwitchingValveVici@COM3:INFO - Valve SwitchingValveVici@COM3 is currently in position 2 (connected to Ethanol_wash).

2024-09-27 18:18:18,215<13700>:SwitchingValveVici@COM3:INFO - Moved valve SwitchingValveVici@COM3 to position 8 (connected to Outlet).

2024-09-27 18:18:18,297<22688>:Aladdin@COM12:INFO - Infusion/withdrawing volume set to 15.0 mL at a rate of 70.0 mL/min

2024-09-27 18:18:31,432<22688>:Aladdin@COM12:INFO - Finished infusing 15.0 mL at a rate of 70.0 mL/min

2024-09-27 18:18:31,433<13700>:SwitchingValveVici@COM3:INFO - Finished adding Chemical Ethanol: 15.0 mL (Ethanol_wash -> FALCON_TUBE_50_ML_2363304638976)

2024-09-27 18:18:32,448<13700>:SwitchingValveVici@COM3:INFO - Performing purging step into container FALCON_TUBE_50_ML: 15.0 mL at SwitchingValveVici@COM3->slot 0...

2024-09-27 18:18:33,685<13700>:SwitchingValveVici@COM3:INFO - Valve SwitchingValveVici@COM3 is currently in position 8 (connected to Outlet).

2024-09-27 18:18:33,685<13700>:SwitchingValveVici@COM3:INFO - Moved valve SwitchingValveVici@COM3 to position 8 (connected to Outlet).

2024-09-27 18:18:33,767<22688>:Aladdin@COM12:INFO - Infusion/withdrawing volume set to 30.0 mL at a rate of 70.0 mL/min

2024-09-27 18:18:59,653<22688>:Aladdin@COM12:INFO - Finished withdrawing 30.0 mL at a rate of 70.0 mL/min

2024-09-27 18:19:00,946<22688>:Aladdin@COM12:INFO - Infusion/withdrawing volume set to 30.0 mL at a rate of 70.0 mL/min

2024-09-27 18:19:26,883<22688>:Aladdin@COM12:INFO - Finished infusing 30.0 mL at a rate of 70.0 mL/min

2024-09-27 18:19:26,884<13700>:Minerva.API.MinervaAPI:SYNTHESIS_STEP - Add Chemical [FALCON_TUBE_50_ML_2363304638976]: FALCON_TUBE_50_ML; Chemical Ethanol: 15.0 mL; 70.0 mL/min; 50 mL Syringe

2024-09-27 18:19:28,408<20644>:XArm6@192.168.1.204:INFO - Finished moving robot arm to source destination: SwitchingValveVici@COM3.

2024-09-27 18:19:37,123<20644>:XArm6@192.168.1.204:INFO - Finished moving robot arm to target destination: UP200ST@192.168.233.233.

2024-09-27 18:19:37,713<14772>:UP200ST@192.168.233.233:INFO - Amplitude changed to 50.0 %.

2024-09-27 18:19:37,740<14772>:UP200ST@192.168.233.233:INFO - Power changed to 50.0 %.

2024-09-27 18:19:37,765<14772>:UP200ST@192.168.233.233:INFO - Time limit activated.

2024-09-27 18:19:37,793<14772>:UP200ST@192.168.233.233:INFO - Time limit changed to 90.0 s.

2024-09-27 18:19:37,793<14772>:UP200ST@192.168.233.233:INFO - Starting sonication...

2024-09-27 18:19:37,818<14772>:UP200ST@192.168.233.233:INFO - Ultrasound turned on.

2024-09-27 18:21:07,862<14772>:UP200ST@192.168.233.233:INFO - Ultrasound turned off.

2024-09-27 18:21:07,863<14772>:UP200ST@192.168.233.233:INFO - Finished sonication.

2024-09-27 18:21:07,863<13700>:Minerva.API.MinervaAPI:SYNTHESIS_STEP - Sonicate [FALCON_TUBE_50_ML_2363304638976]: 90.0 s; 50 % Amplitude; 50 % Power

2024-09-27 18:21:09,450<20644>:XArm6@192.168.1.204:INFO - Finished moving robot arm to source destination: UP200ST@192.168.233.233.

2024-09-27 18:21:22,007<20644>:XArm6@192.168.1.204:INFO - Finished moving robot arm to target destination: CapperDecapper@ArduinoController@COM25.

2024-09-27 18:21:22,560<23296>:CapperDecapper@ArduinoController@COM25:INFO - Closing Container...

2024-09-27 18:21:22,592<23296>:CapperDecapper@ArduinoController@COM25:INFO - Wrist turning clockwise

2024-09-27 18:21:25,726<23296>:CapperDecapper@ArduinoController@COM25:INFO - Wrist turning clockwise

2024-09-27 18:21:26,316<23296>:CapperDecapper@ArduinoController@COM25:INFO - DC Motor Current [mA]: 247.90

2024-09-27 18:21:26,398<23296>:CapperDecapper@ArduinoController@COM25:INFO - DC Motor Current [mA]: 240.65

2024-09-27 18:21:26,480<23296>:CapperDecapper@ArduinoController@COM25:INFO - DC Motor Current [mA]: 241.70

2024-09-27 18:21:26,562<23296>:CapperDecapper@ArduinoController@COM25:INFO - DC Motor Current [mA]: 241.33

2024-09-27 18:21:26,643<23296>:CapperDecapper@ArduinoController@COM25:INFO - DC Motor Current [mA]: 238.30

2024-09-27 18:21:26,725<23296>:CapperDecapper@ArduinoController@COM25:INFO - DC Motor Current [mA]: 252.27

2024-09-27 18:21:26,807<23296>:CapperDecapper@ArduinoController@COM25:INFO - DC Motor Current [mA]: 277.13

2024-09-27 18:21:26,889<23296>:CapperDecapper@ArduinoController@COM25:INFO - DC Motor Current [mA]: 278.32

2024-09-27 18:21:26,971<23296>:CapperDecapper@ArduinoController@COM25:INFO - DC Motor Current [mA]: 280.08

2024-09-27 18:21:27,053<23296>:CapperDecapper@ArduinoController@COM25:INFO - DC Motor Current [mA]: 271.88

2024-09-27 18:21:27,135<23296>:CapperDecapper@ArduinoController@COM25:INFO - DC Motor Current [mA]: 257.27

2024-09-27 18:21:27,217<23296>:CapperDecapper@ArduinoController@COM25:INFO - DC Motor Current [mA]: 251.30

2024-09-27 18:21:27,299<23296>:CapperDecapper@ArduinoController@COM25:INFO - DC Motor Current [mA]: 238.32

2024-09-27 18:21:27,381<23296>:CapperDecapper@ArduinoController@COM25:INFO - DC Motor Current [mA]: 237.58

2024-09-27 18:21:27,463<23296>:CapperDecapper@ArduinoController@COM25:INFO - DC Motor Current [mA]: 235.70

2024-09-27 18:21:27,545<23296>:CapperDecapper@ArduinoController@COM25:INFO - DC Motor Current [mA]: 234.98

2024-09-27 18:21:27,627<23296>:CapperDecapper@ArduinoController@COM25:INFO - DC Motor Current [mA]: 235.08

2024-09-27 18:21:27,708<23296>:CapperDecapper@ArduinoController@COM25:INFO - DC Motor Current [mA]: 235.40

2024-09-27 18:21:27,790<23296>:CapperDecapper@ArduinoController@COM25:INFO - DC Motor Current [mA]: 232.43

2024-09-27 18:21:27,872<23296>:CapperDecapper@ArduinoController@COM25:INFO - DC Motor Current [mA]: 232.20

2024-09-27 18:21:27,954<23296>:CapperDecapper@ArduinoController@COM25:INFO - DC Motor Current [mA]: 230.30

2024-09-27 18:21:28,036<23296>:CapperDecapper@ArduinoController@COM25:INFO - DC Motor Current [mA]: 232.23

2024-09-27 18:21:28,118<23296>:CapperDecapper@ArduinoController@COM25:INFO - DC Motor Current [mA]: 237.48

2024-09-27 18:21:28,200<23296>:CapperDecapper@ArduinoController@COM25:INFO - DC Motor Current [mA]: 241.72

2024-09-27 18:21:28,282<23296>:CapperDecapper@ArduinoController@COM25:INFO - DC Motor Current [mA]: 258.85

2024-09-27 18:21:28,364<23296>:CapperDecapper@ArduinoController@COM25:INFO - DC Motor Current [mA]: 268.28

2024-09-27 18:21:28,446<23296>:CapperDecapper@ArduinoController@COM25:INFO - DC Motor Current [mA]: 274.50

2024-09-27 18:21:28,528<23296>:CapperDecapper@ArduinoController@COM25:INFO - DC Motor Current [mA]: 262.60

2024-09-27 18:21:28,609<23296>:CapperDecapper@ArduinoController@COM25:INFO - DC Motor Current [mA]: 261.00

2024-09-27 18:21:28,691<23296>:CapperDecapper@ArduinoController@COM25:INFO - DC Motor Current [mA]: 255.45

2024-09-27 18:21:28,773<23296>:CapperDecapper@ArduinoController@COM25:INFO - DC Motor Current [mA]: 234.95

2024-09-27 18:21:28,855<23296>:CapperDecapper@ArduinoController@COM25:INFO - DC Motor Current [mA]: 232.68

2024-09-27 18:21:28,888<23296>:CapperDecapper@ArduinoController@COM25:INFO - Stopped turning wrist

2024-09-27 18:21:30,878<23296>:CapperDecapper@ArduinoController@COM25:INFO - Clamp opened.

2024-09-27 18:21:34,095<20644>:XArm6@192.168.1.204:INFO - Finished moving robot arm to source destination: CapperDecapper@ArduinoController@COM25.

2024-09-27 18:22:01,302<20644>:XArm6@192.168.1.204:INFO - Changed grip on container FALCON_TUBE_50_ML_2363304638976 to a top grip.

2024-09-27 18:22:17,399<20644>:XArm6@192.168.1.204:INFO - Finished moving robot arm to target destination: Isolab_50mL_Foldable_Tube_Rack->deck 1.

2024-09-27 18:22:27,137<20644>:XArm6@192.168.1.204:INFO - Finished moving robot arm to source destination: Isolab_50mL_Foldable_Tube_Rack->deck 1.

2024-09-27 18:22:52,576<20644>:XArm6@192.168.1.204:INFO - Changed grip on container FALCON_TUBE_50_ML_2363304718592 to a sideways grip.

2024-09-27 18:22:57,965<20644>:XArm6@192.168.1.204:INFO - Finished moving robot arm to target destination: UP200ST@192.168.233.233.

2024-09-27 18:22:58,554<14772>:UP200ST@192.168.233.233:INFO - Amplitude changed to 50.0 %.

2024-09-27 18:22:58,602<14772>:UP200ST@192.168.233.233:INFO - Power changed to 50.0 %.

2024-09-27 18:22:58,626<14772>:UP200ST@192.168.233.233:INFO - Time limit activated.

2024-09-27 18:22:58,665<14772>:UP200ST@192.168.233.233:INFO - Time limit changed to 10.0 s.

2024-09-27 18:22:58,666<14772>:UP200ST@192.168.233.233:INFO - Starting sonication...

2024-09-27 18:22:58,702<14772>:UP200ST@192.168.233.233:INFO - Ultrasound turned on.

2024-09-27 18:23:08,742<14772>:UP200ST@192.168.233.233:INFO - Ultrasound turned off.

2024-09-27 18:23:08,742<14772>:UP200ST@192.168.233.233:INFO - Finished sonication.

2024-09-27 18:23:10,468<20644>:XArm6@192.168.1.204:INFO - Finished moving robot arm to source destination: UP200ST@192.168.233.233.

2024-09-27 18:23:30,083<20644>:XArm6@192.168.1.204:INFO - Changed grip on container FALCON_TUBE_50_ML_2363304718592 to a top grip.

2024-09-27 18:23:46,817<20644>:XArm6@192.168.1.204:INFO - Finished moving robot arm to target destination: Isolab_50mL_Foldable_Tube_Rack->deck 1.

2024-09-27 18:23:47,370<13700>:Minerva.API.MinervaAPI:SYNTHESIS_STEP - Sonicate [FALCON_TUBE_50_ML_2363304718592]: 10.0 s; 50 % Amplitude; 50 % Power

2024-09-27 18:23:47,371<13700>:Minerva.API.MinervaAPI:SYNTHESIS_STEP - Remove supernatant and redisperse [FALCON_TUBE_50_ML_2363304638976]: Chemical Ethanol: 15.0 mL

2024-09-27 18:23:56,008<20644>:XArm6@192.168.1.204:INFO - Finished moving robot arm to source destination: Isolab_50mL_Foldable_Tube_Rack->deck 1.

2024-09-27 18:24:21,993<20644>:XArm6@192.168.1.204:INFO - Changed grip on container FALCON_TUBE_50_ML_2363304639264 to a sideways grip.

2024-09-27 18:24:35,536<20644>:XArm6@192.168.1.204:INFO - Finished moving robot arm to target destination: CapperDecapper@ArduinoController@COM25.

2024-09-27 18:24:36,089<23296>:CapperDecapper@ArduinoController@COM25:INFO - Opening Container...

2024-09-27 18:24:36,186<23296>:CapperDecapper@ArduinoController@COM25:INFO - Clamp opened.

2024-09-27 18:24:40,646<23296>:CapperDecapper@ArduinoController@COM25:INFO - Clamp closed.

2024-09-27 18:24:40,678<23296>:CapperDecapper@ArduinoController@COM25:INFO - Wrist turning counterclockwise

2024-09-27 18:24:42,722<23296>:CapperDecapper@ArduinoController@COM25:INFO - Stopped turning wrist

2024-09-27 18:24:44,110<20644>:XArm6@192.168.1.204:INFO - Finished moving robot arm to source destination: CapperDecapper@ArduinoController@COM25.

2024-09-27 18:25:01,206<20644>:XArm6@192.168.1.204:INFO - Finished moving robot arm to target destination: SwitchingValveVici@COM3.

2024-09-27 18:25:01,797<13700>:SwitchingValveVici@COM3:INFO - Valve SwitchingValveVici@COM3 is currently in position 8 (connected to Outlet).

2024-09-27 18:25:01,798<13700>:SwitchingValveVici@COM3:INFO - Moved valve SwitchingValveVici@COM3 to position 8 (connected to Outlet).

2024-09-27 18:25:01,879<22688>:Aladdin@COM12:INFO - Infusion/withdrawing volume set to 15.0 mL at a rate of 70.0 mL/min

2024-09-27 18:25:14,919<22688>:Aladdin@COM12:INFO - Finished withdrawing 15.0 mL at a rate of 70.0 mL/min

2024-09-27 18:25:14,950<13700>:SwitchingValveVici@COM3:INFO - Valve SwitchingValveVici@COM3 is currently in position 8 (connected to Outlet).

2024-09-27 18:25:15,445<13700>:SwitchingValveVici@COM3:INFO - Moved valve SwitchingValveVici@COM3 to position 3 (connected to Waste_Container).

2024-09-27 18:25:15,541<22688>:Aladdin@COM12:INFO - Infusion/withdrawing volume set to 15.0 mL at a rate of 70.0 mL/min

2024-09-27 18:25:28,677<22688>:Aladdin@COM12:INFO - Finished infusing 15.0 mL at a rate of 70.0 mL/min

2024-09-27 18:25:28,678<13700>:SwitchingValveVici@COM3:INFO - Performing purging step into container Container[Waste_Container]: 2.315 L at SwitchingValveVici@COM3->slot 3...

2024-09-27 18:25:28,709<13700>:SwitchingValveVici@COM3:INFO - Valve SwitchingValveVici@COM3 is currently in position 3 (connected to Waste_Container).

2024-09-27 18:25:29,205<13700>:SwitchingValveVici@COM3:INFO - Moved valve SwitchingValveVici@COM3 to position 8 (connected to Outlet).

2024-09-27 18:25:29,300<22688>:Aladdin@COM12:INFO - Infusion/withdrawing volume set to 10.0 mL at a rate of 70.0 mL/min

2024-09-27 18:25:38,200<22688>:Aladdin@COM12:INFO - Finished withdrawing 10.0 mL at a rate of 70.0 mL/min

2024-09-27 18:25:38,233<13700>:SwitchingValveVici@COM3:INFO - Valve SwitchingValveVici@COM3 is currently in position 8 (connected to Outlet).

2024-09-27 18:25:38,730<13700>:SwitchingValveVici@COM3:INFO - Moved valve SwitchingValveVici@COM3 to position 3 (connected to Waste_Container).

2024-09-27 18:25:38,823<22688>:Aladdin@COM12:INFO - Infusion/withdrawing volume set to 10.0 mL at a rate of 70.0 mL/min

2024-09-27 18:25:47,660<22688>:Aladdin@COM12:INFO - Finished infusing 10.0 mL at a rate of 70.0 mL/min

2024-09-27 18:25:47,663<13700>:SwitchingValveVici@COM3:INFO - Performing addition step for chemical Chemical Ethanol: 15.0 mL (Ethanol_wash -> FALCON_TUBE_50_ML_2363304639264)

2024-09-27 18:25:47,696<13700>:SwitchingValveVici@COM3:INFO - Valve SwitchingValveVici@COM3 is currently in position 3 (connected to Waste_Container).

2024-09-27 18:25:47,852<13700>:SwitchingValveVici@COM3:INFO - Moved valve SwitchingValveVici@COM3 to position 2 (connected to Ethanol_wash).

2024-09-27 18:25:47,932<22688>:Aladdin@COM12:INFO - Infusion/withdrawing volume set to 15.0 mL at a rate of 70.0 mL/min

2024-09-27 18:26:00,955<22688>:Aladdin@COM12:INFO - Finished withdrawing 15.0 mL at a rate of 70.0 mL/min

2024-09-27 18:26:00,989<13700>:SwitchingValveVici@COM3:INFO - Valve SwitchingValveVici@COM3 is currently in position 2 (connected to Ethanol_wash).

2024-09-27 18:26:01,404<13700>:SwitchingValveVici@COM3:INFO - Moved valve SwitchingValveVici@COM3 to position 8 (connected to Outlet).

2024-09-27 18:26:01,498<22688>:Aladdin@COM12:INFO - Infusion/withdrawing volume set to 15.0 mL at a rate of 70.0 mL/min

2024-09-27 18:26:14,650<22688>:Aladdin@COM12:INFO - Finished infusing 15.0 mL at a rate of 70.0 mL/min

2024-09-27 18:26:14,651<13700>:SwitchingValveVici@COM3:INFO - Finished adding Chemical Ethanol: 15.0 mL (Ethanol_wash -> FALCON_TUBE_50_ML_2363304639264)

2024-09-27 18:26:15,658<13700>:SwitchingValveVici@COM3:INFO - Performing purging step into container FALCON_TUBE_50_ML: 15.0 mL at SwitchingValveVici@COM3->slot 0...

2024-09-27 18:26:16,903<13700>:SwitchingValveVici@COM3:INFO - Valve SwitchingValveVici@COM3 is currently in position 8 (connected to Outlet).

2024-09-27 18:26:16,904<13700>:SwitchingValveVici@COM3:INFO - Moved valve SwitchingValveVici@COM3 to position 8 (connected to Outlet).

2024-09-27 18:26:16,998<22688>:Aladdin@COM12:INFO - Infusion/withdrawing volume set to 30.0 mL at a rate of 70.0 mL/min

2024-09-27 18:26:43,062<22688>:Aladdin@COM12:INFO - Finished withdrawing 30.0 mL at a rate of 70.0 mL/min

2024-09-27 18:26:44,354<22688>:Aladdin@COM12:INFO - Infusion/withdrawing volume set to 30.0 mL at a rate of 70.0 mL/min

2024-09-27 18:27:10,337<22688>:Aladdin@COM12:INFO - Finished infusing 30.0 mL at a rate of 70.0 mL/min

2024-09-27 18:27:10,338<13700>:Minerva.API.MinervaAPI:SYNTHESIS_STEP - Add Chemical [FALCON_TUBE_50_ML_2363304639264]: FALCON_TUBE_50_ML; Chemical Ethanol: 15.0 mL; 70.0 mL/min; 50 mL Syringe

2024-09-27 18:27:11,867<20644>:XArm6@192.168.1.204:INFO - Finished moving robot arm to source destination: SwitchingValveVici@COM3.

2024-09-27 18:27:20,561<20644>:XArm6@192.168.1.204:INFO - Finished moving robot arm to target destination: UP200ST@192.168.233.233.

2024-09-27 18:27:21,163<14772>:UP200ST@192.168.233.233:INFO - Amplitude changed to 50.0 %.

2024-09-27 18:27:21,191<14772>:UP200ST@192.168.233.233:INFO - Power changed to 50.0 %.

2024-09-27 18:27:21,215<14772>:UP200ST@192.168.233.233:INFO - Time limit activated.

2024-09-27 18:27:21,243<14772>:UP200ST@192.168.233.233:INFO - Time limit changed to 90.0 s.

2024-09-27 18:27:21,243<14772>:UP200ST@192.168.233.233:INFO - Starting sonication...

2024-09-27 18:27:21,268<14772>:UP200ST@192.168.233.233:INFO - Ultrasound turned on.

2024-09-27 18:28:51,319<14772>:UP200ST@192.168.233.233:INFO - Ultrasound turned off.

2024-09-27 18:28:51,319<14772>:UP200ST@192.168.233.233:INFO - Finished sonication.

2024-09-27 18:28:51,320<13700>:Minerva.API.MinervaAPI:SYNTHESIS_STEP - Sonicate [FALCON_TUBE_50_ML_2363304639264]: 90.0 s; 50 % Amplitude; 50 % Power

2024-09-27 18:28:54,540<20644>:XArm6@192.168.1.204:INFO - Finished moving robot arm to source destination: UP200ST@192.168.233.233.

2024-09-27 18:29:06,143<20644>:XArm6@192.168.1.204:INFO - Finished moving robot arm to target destination: CapperDecapper@ArduinoController@COM25.

2024-09-27 18:29:06,696<23296>:CapperDecapper@ArduinoController@COM25:INFO - Closing Container...

2024-09-27 18:29:06,729<23296>:CapperDecapper@ArduinoController@COM25:INFO - Wrist turning clockwise

2024-09-27 18:29:09,861<23296>:CapperDecapper@ArduinoController@COM25:INFO - Wrist turning clockwise

2024-09-27 18:29:10,447<23296>:CapperDecapper@ArduinoController@COM25:INFO - DC Motor Current [mA]: 235.43

2024-09-27 18:29:10,529<23296>:CapperDecapper@ArduinoController@COM25:INFO - DC Motor Current [mA]: 236.40

2024-09-27 18:29:10,611<23296>:CapperDecapper@ArduinoController@COM25:INFO - DC Motor Current [mA]: 242.92

2024-09-27 18:29:10,693<23296>:CapperDecapper@ArduinoController@COM25:INFO - DC Motor Current [mA]: 241.18

2024-09-27 18:29:10,775<23296>:CapperDecapper@ArduinoController@COM25:INFO - DC Motor Current [mA]: 248.10

2024-09-27 18:29:10,857<23296>:CapperDecapper@ArduinoController@COM25:INFO - DC Motor Current [mA]: 231.58

2024-09-27 18:29:10,939<23296>:CapperDecapper@ArduinoController@COM25:INFO - DC Motor Current [mA]: 232.42

2024-09-27 18:29:11,021<23296>:CapperDecapper@ArduinoController@COM25:INFO - DC Motor Current [mA]: 245.90

2024-09-27 18:29:11,102<23296>:CapperDecapper@ArduinoController@COM25:INFO - DC Motor Current [mA]: 256.90

2024-09-27 18:29:11,184<23296>:CapperDecapper@ArduinoController@COM25:INFO - DC Motor Current [mA]: 261.13

2024-09-27 18:29:11,266<23296>:CapperDecapper@ArduinoController@COM25:INFO - DC Motor Current [mA]: 263.95

2024-09-27 18:29:11,348<23296>:CapperDecapper@ArduinoController@COM25:INFO - DC Motor Current [mA]: 248.98

2024-09-27 18:29:11,430<23296>:CapperDecapper@ArduinoController@COM25:INFO - DC Motor Current [mA]: 242.40

2024-09-27 18:29:11,512<23296>:CapperDecapper@ArduinoController@COM25:INFO - DC Motor Current [mA]: 222.30

2024-09-27 18:29:11,594<23296>:CapperDecapper@ArduinoController@COM25:INFO - DC Motor Current [mA]: 212.25

2024-09-27 18:29:11,676<23296>:CapperDecapper@ArduinoController@COM25:INFO - DC Motor Current [mA]: 207.13

2024-09-27 18:29:11,758<23296>:CapperDecapper@ArduinoController@COM25:INFO - DC Motor Current [mA]: 213.67

2024-09-27 18:29:11,840<23296>:CapperDecapper@ArduinoController@COM25:INFO - DC Motor Current [mA]: 220.70

2024-09-27 18:29:11,921<23296>:CapperDecapper@ArduinoController@COM25:INFO - DC Motor Current [mA]: 233.78

2024-09-27 18:29:12,003<23296>:CapperDecapper@ArduinoController@COM25:INFO - DC Motor Current [mA]: 237.55

2024-09-27 18:29:12,085<23296>:CapperDecapper@ArduinoController@COM25:INFO - DC Motor Current [mA]: 242.50

2024-09-27 18:29:12,167<23296>:CapperDecapper@ArduinoController@COM25:INFO - DC Motor Current [mA]: 240.42

2024-09-27 18:29:12,249<23296>:CapperDecapper@ArduinoController@COM25:INFO - DC Motor Current [mA]: 234.80

2024-09-27 18:29:12,331<23296>:CapperDecapper@ArduinoController@COM25:INFO - DC Motor Current [mA]: 221.67

2024-09-27 18:29:12,413<23296>:CapperDecapper@ArduinoController@COM25:INFO - DC Motor Current [mA]: 225.30

2024-09-27 18:29:12,496<23296>:CapperDecapper@ArduinoController@COM25:INFO - DC Motor Current [mA]: 239.50

2024-09-27 18:29:12,577<23296>:CapperDecapper@ArduinoController@COM25:INFO - DC Motor Current [mA]: 247.35

2024-09-27 18:29:12,659<23296>:CapperDecapper@ArduinoController@COM25:INFO - DC Motor Current [mA]: 245.95

2024-09-27 18:29:12,741<23296>:CapperDecapper@ArduinoController@COM25:INFO - DC Motor Current [mA]: 258.52

2024-09-27 18:29:12,823<23296>:CapperDecapper@ArduinoController@COM25:INFO - DC Motor Current [mA]: 243.18

2024-09-27 18:29:12,905<23296>:CapperDecapper@ArduinoController@COM25:INFO - DC Motor Current [mA]: 231.55

2024-09-27 18:29:12,987<23296>:CapperDecapper@ArduinoController@COM25:INFO - DC Motor Current [mA]: 217.45

2024-09-27 18:29:13,019<23296>:CapperDecapper@ArduinoController@COM25:INFO - Stopped turning wrist

2024-09-27 18:29:15,010<23296>:CapperDecapper@ArduinoController@COM25:INFO - Clamp opened.

2024-09-27 18:29:16,404<20644>:XArm6@192.168.1.204:INFO - Finished moving robot arm to source destination: CapperDecapper@ArduinoController@COM25.

2024-09-27 18:29:44,992<20644>:XArm6@192.168.1.204:INFO - Changed grip on container FALCON_TUBE_50_ML_2363304639264 to a top grip.

2024-09-27 18:30:01,956<20644>:XArm6@192.168.1.204:INFO - Finished moving robot arm to target destination: Isolab_50mL_Foldable_Tube_Rack->deck 1.

2024-09-27 18:30:02,516<13700>:Minerva.API.MinervaAPI:SYNTHESIS_STEP - Remove supernatant and redisperse [FALCON_TUBE_50_ML_2363304639264]: Chemical Ethanol: 15.0 mL

2024-09-27 18:30:10,852<20644>:XArm6@192.168.1.204:INFO - Finished moving robot arm to source destination: Isolab_50mL_Foldable_Tube_Rack->deck 1.

2024-09-27 18:30:36,649<20644>:XArm6@192.168.1.204:INFO - Changed grip on container FALCON_TUBE_50_ML_2363304639552 to a sideways grip.

2024-09-27 18:30:49,588<20644>:XArm6@192.168.1.204:INFO - Finished moving robot arm to target destination: CapperDecapper@ArduinoController@COM25.

2024-09-27 18:30:50,140<23296>:CapperDecapper@ArduinoController@COM25:INFO - Opening Container...

2024-09-27 18:30:50,236<23296>:CapperDecapper@ArduinoController@COM25:INFO - Clamp opened.

2024-09-27 18:30:54,707<23296>:CapperDecapper@ArduinoController@COM25:INFO - Clamp closed.

2024-09-27 18:30:54,740<23296>:CapperDecapper@ArduinoController@COM25:INFO - Wrist turning counterclockwise

2024-09-27 18:30:56,776<23296>:CapperDecapper@ArduinoController@COM25:INFO - Stopped turning wrist

2024-09-27 18:30:58,169<20644>:XArm6@192.168.1.204:INFO - Finished moving robot arm to source destination: CapperDecapper@ArduinoController@COM25.

2024-09-27 18:31:15,381<20644>:XArm6@192.168.1.204:INFO - Finished moving robot arm to target destination: SwitchingValveVici@COM3.

2024-09-27 18:31:15,974<13700>:SwitchingValveVici@COM3:INFO - Valve SwitchingValveVici@COM3 is currently in position 8 (connected to Outlet).

2024-09-27 18:31:15,974<13700>:SwitchingValveVici@COM3:INFO - Moved valve SwitchingValveVici@COM3 to position 8 (connected to Outlet).

2024-09-27 18:31:16,055<22688>:Aladdin@COM12:INFO - Infusion/withdrawing volume set to 15.0 mL at a rate of 70.0 mL/min

2024-09-27 18:31:29,096<22688>:Aladdin@COM12:INFO - Finished withdrawing 15.0 mL at a rate of 70.0 mL/min

2024-09-27 18:31:29,127<13700>:SwitchingValveVici@COM3:INFO - Valve SwitchingValveVici@COM3 is currently in position 8 (connected to Outlet).

2024-09-27 18:31:29,623<13700>:SwitchingValveVici@COM3:INFO - Moved valve SwitchingValveVici@COM3 to position 3 (connected to Waste_Container).

2024-09-27 18:31:29,719<22688>:Aladdin@COM12:INFO - Infusion/withdrawing volume set to 15.0 mL at a rate of 70.0 mL/min

2024-09-27 18:31:42,709<22688>:Aladdin@COM12:INFO - Finished infusing 15.0 mL at a rate of 70.0 mL/min

2024-09-27 18:31:42,710<13700>:SwitchingValveVici@COM3:INFO - Performing purging step into container Container[Waste_Container]: 2.33 L at SwitchingValveVici@COM3->slot 3...

2024-09-27 18:31:42,742<13700>:SwitchingValveVici@COM3:INFO - Valve SwitchingValveVici@COM3 is currently in position 3 (connected to Waste_Container).

2024-09-27 18:31:43,237<13700>:SwitchingValveVici@COM3:INFO - Moved valve SwitchingValveVici@COM3 to position 8 (connected to Outlet).

2024-09-27 18:31:43,332<22688>:Aladdin@COM12:INFO - Infusion/withdrawing volume set to 10.0 mL at a rate of 70.0 mL/min

2024-09-27 18:31:52,138<22688>:Aladdin@COM12:INFO - Finished withdrawing 10.0 mL at a rate of 70.0 mL/min

2024-09-27 18:31:52,169<13700>:SwitchingValveVici@COM3:INFO - Valve SwitchingValveVici@COM3 is currently in position 8 (connected to Outlet).

2024-09-27 18:31:52,665<13700>:SwitchingValveVici@COM3:INFO - Moved valve SwitchingValveVici@COM3 to position 3 (connected to Waste_Container).

2024-09-27 18:31:52,744<22688>:Aladdin@COM12:INFO - Infusion/withdrawing volume set to 10.0 mL at a rate of 70.0 mL/min

2024-09-27 18:32:01,645<22688>:Aladdin@COM12:INFO - Finished infusing 10.0 mL at a rate of 70.0 mL/min

2024-09-27 18:32:01,647<13700>:SwitchingValveVici@COM3:INFO - Performing addition step for chemical Chemical Ethanol: 15.0 mL (Ethanol_wash -> FALCON_TUBE_50_ML_2363304639552)

2024-09-27 18:32:01,678<13700>:SwitchingValveVici@COM3:INFO - Valve SwitchingValveVici@COM3 is currently in position 3 (connected to Waste_Container).

2024-09-27 18:32:01,835<13700>:SwitchingValveVici@COM3:INFO - Moved valve SwitchingValveVici@COM3 to position 2 (connected to Ethanol_wash).

2024-09-27 18:32:01,916<22688>:Aladdin@COM12:INFO - Infusion/withdrawing volume set to 15.0 mL at a rate of 70.0 mL/min

2024-09-27 18:32:14,939<22688>:Aladdin@COM12:INFO - Finished withdrawing 15.0 mL at a rate of 70.0 mL/min

2024-09-27 18:32:14,973<13700>:SwitchingValveVici@COM3:INFO - Valve SwitchingValveVici@COM3 is currently in position 2 (connected to Ethanol_wash).

2024-09-27 18:32:15,388<13700>:SwitchingValveVici@COM3:INFO - Moved valve SwitchingValveVici@COM3 to position 8 (connected to Outlet).

2024-09-27 18:32:15,483<22688>:Aladdin@COM12:INFO - Infusion/withdrawing volume set to 15.0 mL at a rate of 70.0 mL/min

2024-09-27 18:32:28,521<22688>:Aladdin@COM12:INFO - Finished infusing 15.0 mL at a rate of 70.0 mL/min

2024-09-27 18:32:28,522<13700>:SwitchingValveVici@COM3:INFO - Finished adding Chemical Ethanol: 15.0 mL (Ethanol_wash -> FALCON_TUBE_50_ML_2363304639552)

2024-09-27 18:32:29,533<13700>:SwitchingValveVici@COM3:INFO - Performing purging step into container FALCON_TUBE_50_ML: 15.0 mL at SwitchingValveVici@COM3->slot 0...

2024-09-27 18:32:30,776<13700>:SwitchingValveVici@COM3:INFO - Valve SwitchingValveVici@COM3 is currently in position 8 (connected to Outlet).

2024-09-27 18:32:30,777<13700>:SwitchingValveVici@COM3:INFO - Moved valve SwitchingValveVici@COM3 to position 8 (connected to Outlet).

2024-09-27 18:32:30,869<22688>:Aladdin@COM12:INFO - Infusion/withdrawing volume set to 30.0 mL at a rate of 70.0 mL/min

2024-09-27 18:32:56,726<22688>:Aladdin@COM12:INFO - Finished withdrawing 30.0 mL at a rate of 70.0 mL/min

2024-09-27 18:32:58,020<22688>:Aladdin@COM12:INFO - Infusion/withdrawing volume set to 30.0 mL at a rate of 70.0 mL/min

2024-09-27 18:33:23,938<22688>:Aladdin@COM12:INFO - Finished infusing 30.0 mL at a rate of 70.0 mL/min

2024-09-27 18:33:23,938<13700>:Minerva.API.MinervaAPI:SYNTHESIS_STEP - Add Chemical [FALCON_TUBE_50_ML_2363304639552]: FALCON_TUBE_50_ML; Chemical Ethanol: 15.0 mL; 70.0 mL/min; 50 mL Syringe

2024-09-27 18:33:25,463<20644>:XArm6@192.168.1.204:INFO - Finished moving robot arm to source destination: SwitchingValveVici@COM3.

2024-09-27 18:33:34,133<20644>:XArm6@192.168.1.204:INFO - Finished moving robot arm to target destination: UP200ST@192.168.233.233.

2024-09-27 18:33:34,751<14772>:UP200ST@192.168.233.233:INFO - Amplitude changed to 50.0 %.

2024-09-27 18:33:34,779<14772>:UP200ST@192.168.233.233:INFO - Power changed to 50.0 %.

2024-09-27 18:33:34,803<14772>:UP200ST@192.168.233.233:INFO - Time limit activated.

2024-09-27 18:33:34,831<14772>:UP200ST@192.168.233.233:INFO - Time limit changed to 90.0 s.

2024-09-27 18:33:34,831<14772>:UP200ST@192.168.233.233:INFO - Starting sonication...

2024-09-27 18:33:34,856<14772>:UP200ST@192.168.233.233:INFO - Ultrasound turned on.

2024-09-27 18:35:04,902<14772>:UP200ST@192.168.233.233:INFO - Ultrasound turned off.

2024-09-27 18:35:04,902<14772>:UP200ST@192.168.233.233:INFO - Finished sonication.

2024-09-27 18:35:04,903<13700>:Minerva.API.MinervaAPI:SYNTHESIS_STEP - Sonicate [FALCON_TUBE_50_ML_2363304639552]: 90.0 s; 50 % Amplitude; 50 % Power

2024-09-27 18:35:06,491<20644>:XArm6@192.168.1.204:INFO - Finished moving robot arm to source destination: UP200ST@192.168.233.233.

2024-09-27 18:35:19,681<20644>:XArm6@192.168.1.204:INFO - Finished moving robot arm to target destination: CapperDecapper@ArduinoController@COM25.

2024-09-27 18:35:20,238<23296>:CapperDecapper@ArduinoController@COM25:INFO - Closing Container...

2024-09-27 18:35:20,267<23296>:CapperDecapper@ArduinoController@COM25:INFO - Wrist turning clockwise

2024-09-27 18:35:23,355<23296>:CapperDecapper@ArduinoController@COM25:INFO - Wrist turning clockwise

2024-09-27 18:35:23,949<23296>:CapperDecapper@ArduinoController@COM25:INFO - DC Motor Current [mA]: 232.63

2024-09-27 18:35:24,031<23296>:CapperDecapper@ArduinoController@COM25:INFO - DC Motor Current [mA]: 235.77

2024-09-27 18:35:24,114<23296>:CapperDecapper@ArduinoController@COM25:INFO - DC Motor Current [mA]: 233.20

2024-09-27 18:35:24,196<23296>:CapperDecapper@ArduinoController@COM25:INFO - DC Motor Current [mA]: 231.58

2024-09-27 18:35:24,277<23296>:CapperDecapper@ArduinoController@COM25:INFO - DC Motor Current [mA]: 234.08

2024-09-27 18:35:24,359<23296>:CapperDecapper@ArduinoController@COM25:INFO - DC Motor Current [mA]: 237.58

2024-09-27 18:35:24,441<23296>:CapperDecapper@ArduinoController@COM25:INFO - DC Motor Current [mA]: 244.60

2024-09-27 18:35:24,523<23296>:CapperDecapper@ArduinoController@COM25:INFO - DC Motor Current [mA]: 264.75

2024-09-27 18:35:24,605<23296>:CapperDecapper@ArduinoController@COM25:INFO - DC Motor Current [mA]: 280.65

2024-09-27 18:35:24,687<23296>:CapperDecapper@ArduinoController@COM25:INFO - DC Motor Current [mA]: 287.95

2024-09-27 18:35:24,769<23296>:CapperDecapper@ArduinoController@COM25:INFO - DC Motor Current [mA]: 281.88

2024-09-27 18:35:24,851<23296>:CapperDecapper@ArduinoController@COM25:INFO - DC Motor Current [mA]: 273.92

2024-09-27 18:35:24,933<23296>:CapperDecapper@ArduinoController@COM25:INFO - DC Motor Current [mA]: 247.35

2024-09-27 18:35:25,015<23296>:CapperDecapper@ArduinoController@COM25:INFO - DC Motor Current [mA]: 239.35

2024-09-27 18:35:25,096<23296>:CapperDecapper@ArduinoController@COM25:INFO - DC Motor Current [mA]: 221.73

2024-09-27 18:35:25,179<23296>:CapperDecapper@ArduinoController@COM25:INFO - DC Motor Current [mA]: 213.35

2024-09-27 18:35:25,260<23296>:CapperDecapper@ArduinoController@COM25:INFO - DC Motor Current [mA]: 219.53

2024-09-27 18:35:25,343<23296>:CapperDecapper@ArduinoController@COM25:INFO - DC Motor Current [mA]: 232.13

2024-09-27 18:35:25,424<23296>:CapperDecapper@ArduinoController@COM25:INFO - DC Motor Current [mA]: 229.95

2024-09-27 18:35:25,506<23296>:CapperDecapper@ArduinoController@COM25:INFO - DC Motor Current [mA]: 223.95

2024-09-27 18:35:25,588<23296>:CapperDecapper@ArduinoController@COM25:INFO - DC Motor Current [mA]: 227.43

2024-09-27 18:35:25,669<23296>:CapperDecapper@ArduinoController@COM25:INFO - DC Motor Current [mA]: 223.20

2024-09-27 18:35:25,751<23296>:CapperDecapper@ArduinoController@COM25:INFO - DC Motor Current [mA]: 222.30

2024-09-27 18:35:25,833<23296>:CapperDecapper@ArduinoController@COM25:INFO - DC Motor Current [mA]: 224.55

2024-09-27 18:35:25,916<23296>:CapperDecapper@ArduinoController@COM25:INFO - DC Motor Current [mA]: 231.70

2024-09-27 18:35:25,997<23296>:CapperDecapper@ArduinoController@COM25:INFO - DC Motor Current [mA]: 246.72

2024-09-27 18:35:26,079<23296>:CapperDecapper@ArduinoController@COM25:INFO - DC Motor Current [mA]: 272.90

2024-09-27 18:35:26,161<23296>:CapperDecapper@ArduinoController@COM25:INFO - DC Motor Current [mA]: 284.23

2024-09-27 18:35:26,243<23296>:CapperDecapper@ArduinoController@COM25:INFO - DC Motor Current [mA]: 272.92

2024-09-27 18:35:26,325<23296>:CapperDecapper@ArduinoController@COM25:INFO - DC Motor Current [mA]: 264.80

2024-09-27 18:35:26,407<23296>:CapperDecapper@ArduinoController@COM25:INFO - DC Motor Current [mA]: 249.13

2024-09-27 18:35:26,488<23296>:CapperDecapper@ArduinoController@COM25:INFO - DC Motor Current [mA]: 231.02

2024-09-27 18:35:26,521<23296>:CapperDecapper@ArduinoController@COM25:INFO - Stopped turning wrist

2024-09-27 18:35:28,511<23296>:CapperDecapper@ArduinoController@COM25:INFO - Clamp opened.

2024-09-27 18:35:29,914<20644>:XArm6@192.168.1.204:INFO - Finished moving robot arm to source destination: CapperDecapper@ArduinoController@COM25.

2024-09-27 18:35:58,308<20644>:XArm6@192.168.1.204:INFO - Changed grip on container FALCON_TUBE_50_ML_2363304639552 to a top grip.

2024-09-27 18:36:14,629<20644>:XArm6@192.168.1.204:INFO - Finished moving robot arm to target destination: Isolab_50mL_Foldable_Tube_Rack->deck 1.

2024-09-27 18:36:24,468<20644>:XArm6@192.168.1.204:INFO - Finished moving robot arm to source destination: Isolab_50mL_Foldable_Tube_Rack->deck 1.

2024-09-27 18:36:50,397<20644>:XArm6@192.168.1.204:INFO - Changed grip on container FALCON_TUBE_50_ML_2363304718592 to a sideways grip.

2024-09-27 18:36:55,784<20644>:XArm6@192.168.1.204:INFO - Finished moving robot arm to target destination: UP200ST@192.168.233.233.

2024-09-27 18:36:56,370<14772>:UP200ST@192.168.233.233:INFO - Amplitude changed to 50.0 %.

2024-09-27 18:36:56,398<14772>:UP200ST@192.168.233.233:INFO - Power changed to 50.0 %.

2024-09-27 18:36:56,423<14772>:UP200ST@192.168.233.233:INFO - Time limit activated.

2024-09-27 18:36:56,451<14772>:UP200ST@192.168.233.233:INFO - Time limit changed to 10.0 s.

2024-09-27 18:36:56,451<14772>:UP200ST@192.168.233.233:INFO - Starting sonication...

2024-09-27 18:36:56,477<14772>:UP200ST@192.168.233.233:INFO - Ultrasound turned on.

2024-09-27 18:37:06,527<14772>:UP200ST@192.168.233.233:INFO - Ultrasound turned off.

2024-09-27 18:37:06,527<14772>:UP200ST@192.168.233.233:INFO - Finished sonication.

2024-09-27 18:37:08,243<20644>:XArm6@192.168.1.204:INFO - Finished moving robot arm to source destination: UP200ST@192.168.233.233.

2024-09-27 18:37:27,887<20644>:XArm6@192.168.1.204:INFO - Changed grip on container FALCON_TUBE_50_ML_2363304718592 to a top grip.

2024-09-27 18:37:43,458<20644>:XArm6@192.168.1.204:INFO - Finished moving robot arm to target destination: Isolab_50mL_Foldable_Tube_Rack->deck 1.

2024-09-27 18:37:44,016<13700>:Minerva.API.MinervaAPI:SYNTHESIS_STEP - Sonicate [FALCON_TUBE_50_ML_2363304718592]: 10.0 s; 50 % Amplitude; 50 % Power

2024-09-27 18:37:44,017<13700>:Minerva.API.MinervaAPI:SYNTHESIS_STEP - Remove supernatant and redisperse [FALCON_TUBE_50_ML_2363304639552]: Chemical Ethanol: 15.0 mL

2024-09-27 18:37:52,405<20644>:XArm6@192.168.1.204:INFO - Finished moving robot arm to source destination: Isolab_50mL_Foldable_Tube_Rack->deck 1.

2024-09-27 18:38:17,769<20644>:XArm6@192.168.1.204:INFO - Changed grip on container FALCON_TUBE_50_ML_2363304639840 to a sideways grip.

2024-09-27 18:38:30,722<20644>:XArm6@192.168.1.204:INFO - Finished moving robot arm to target destination: CapperDecapper@ArduinoController@COM25.

2024-09-27 18:38:31,276<23296>:CapperDecapper@ArduinoController@COM25:INFO - Opening Container...

2024-09-27 18:38:31,372<23296>:CapperDecapper@ArduinoController@COM25:INFO - Clamp opened.

2024-09-27 18:38:35,836<23296>:CapperDecapper@ArduinoController@COM25:INFO - Clamp closed.

2024-09-27 18:38:35,868<23296>:CapperDecapper@ArduinoController@COM25:INFO - Wrist turning counterclockwise

2024-09-27 18:38:37,916<23296>:CapperDecapper@ArduinoController@COM25:INFO - Stopped turning wrist

2024-09-27 18:38:39,256<20644>:XArm6@192.168.1.204:INFO - Finished moving robot arm to source destination: CapperDecapper@ArduinoController@COM25.

2024-09-27 18:38:55,835<20644>:XArm6@192.168.1.204:INFO - Finished moving robot arm to target destination: SwitchingValveVici@COM3.

2024-09-27 18:38:56,418<13700>:SwitchingValveVici@COM3:INFO - Valve SwitchingValveVici@COM3 is currently in position 8 (connected to Outlet).

2024-09-27 18:38:56,419<13700>:SwitchingValveVici@COM3:INFO - Moved valve SwitchingValveVici@COM3 to position 8 (connected to Outlet).

2024-09-27 18:38:56,513<22688>:Aladdin@COM12:INFO - Infusion/withdrawing volume set to 15.0 mL at a rate of 70.0 mL/min

2024-09-27 18:39:09,680<22688>:Aladdin@COM12:INFO - Finished withdrawing 15.0 mL at a rate of 70.0 mL/min

2024-09-27 18:39:09,713<13700>:SwitchingValveVici@COM3:INFO - Valve SwitchingValveVici@COM3 is currently in position 8 (connected to Outlet).

2024-09-27 18:39:10,208<13700>:SwitchingValveVici@COM3:INFO - Moved valve SwitchingValveVici@COM3 to position 3 (connected to Waste_Container).

2024-09-27 18:39:10,303<22688>:Aladdin@COM12:INFO - Infusion/withdrawing volume set to 15.0 mL at a rate of 70.0 mL/min

2024-09-27 18:39:23,390<22688>:Aladdin@COM12:INFO - Finished infusing 15.0 mL at a rate of 70.0 mL/min

2024-09-27 18:39:23,391<13700>:SwitchingValveVici@COM3:INFO - Performing purging step into container Container[Waste_Container]: 2.345 L at SwitchingValveVici@COM3->slot 3...

2024-09-27 18:39:23,424<13700>:SwitchingValveVici@COM3:INFO - Valve SwitchingValveVici@COM3 is currently in position 3 (connected to Waste_Container).

2024-09-27 18:39:23,920<13700>:SwitchingValveVici@COM3:INFO - Moved valve SwitchingValveVici@COM3 to position 8 (connected to Outlet).

2024-09-27 18:39:24,013<22688>:Aladdin@COM12:INFO - Infusion/withdrawing volume set to 10.0 mL at a rate of 70.0 mL/min

2024-09-27 18:39:32,802<22688>:Aladdin@COM12:INFO - Finished withdrawing 10.0 mL at a rate of 70.0 mL/min

2024-09-27 18:39:32,835<13700>:SwitchingValveVici@COM3:INFO - Valve SwitchingValveVici@COM3 is currently in position 8 (connected to Outlet).

2024-09-27 18:39:33,331<13700>:SwitchingValveVici@COM3:INFO - Moved valve SwitchingValveVici@COM3 to position 3 (connected to Waste_Container).

2024-09-27 18:39:33,426<22688>:Aladdin@COM12:INFO - Infusion/withdrawing volume set to 10.0 mL at a rate of 70.0 mL/min

2024-09-27 18:39:42,199<22688>:Aladdin@COM12:INFO - Finished infusing 10.0 mL at a rate of 70.0 mL/min

2024-09-27 18:39:42,201<13700>:SwitchingValveVici@COM3:INFO - Performing addition step for chemical Chemical Ethanol: 15.0 mL (Ethanol_wash -> FALCON_TUBE_50_ML_2363304639840)

2024-09-27 18:39:42,233<13700>:SwitchingValveVici@COM3:INFO - Valve SwitchingValveVici@COM3 is currently in position 3 (connected to Waste_Container).

2024-09-27 18:39:42,390<13700>:SwitchingValveVici@COM3:INFO - Moved valve SwitchingValveVici@COM3 to position 2 (connected to Ethanol_wash).

2024-09-27 18:39:42,470<22688>:Aladdin@COM12:INFO - Infusion/withdrawing volume set to 15.0 mL at a rate of 70.0 mL/min

2024-09-27 18:39:55,478<22688>:Aladdin@COM12:INFO - Finished withdrawing 15.0 mL at a rate of 70.0 mL/min

2024-09-27 18:39:55,511<13700>:SwitchingValveVici@COM3:INFO - Valve SwitchingValveVici@COM3 is currently in position 2 (connected to Ethanol_wash).

2024-09-27 18:39:55,926<13700>:SwitchingValveVici@COM3:INFO - Moved valve SwitchingValveVici@COM3 to position 8 (connected to Outlet).

2024-09-27 18:39:56,005<22688>:Aladdin@COM12:INFO - Infusion/withdrawing volume set to 15.0 mL at a rate of 70.0 mL/min

2024-09-27 18:40:08,997<22688>:Aladdin@COM12:INFO - Finished infusing 15.0 mL at a rate of 70.0 mL/min

2024-09-27 18:40:08,998<13700>:SwitchingValveVici@COM3:INFO - Finished adding Chemical Ethanol: 15.0 mL (Ethanol_wash -> FALCON_TUBE_50_ML_2363304639840)

2024-09-27 18:40:10,003<13700>:SwitchingValveVici@COM3:INFO - Performing purging step into container FALCON_TUBE_50_ML: 15.0 mL at SwitchingValveVici@COM3->slot 0...

2024-09-27 18:40:11,237<13700>:SwitchingValveVici@COM3:INFO - Valve SwitchingValveVici@COM3 is currently in position 8 (connected to Outlet).

2024-09-27 18:40:11,237<13700>:SwitchingValveVici@COM3:INFO - Moved valve SwitchingValveVici@COM3 to position 8 (connected to Outlet).

2024-09-27 18:40:11,329<22688>:Aladdin@COM12:INFO - Infusion/withdrawing volume set to 30.0 mL at a rate of 70.0 mL/min

2024-09-27 18:40:37,376<22688>:Aladdin@COM12:INFO - Finished withdrawing 30.0 mL at a rate of 70.0 mL/min

2024-09-27 18:40:38,669<22688>:Aladdin@COM12:INFO - Infusion/withdrawing volume set to 30.0 mL at a rate of 70.0 mL/min

2024-09-27 18:41:04,635<22688>:Aladdin@COM12:INFO - Finished infusing 30.0 mL at a rate of 70.0 mL/min

2024-09-27 18:41:04,636<13700>:Minerva.API.MinervaAPI:SYNTHESIS_STEP - Add Chemical [FALCON_TUBE_50_ML_2363304639840]: FALCON_TUBE_50_ML; Chemical Ethanol: 15.0 mL; 70.0 mL/min; 50 mL Syringe

2024-09-27 18:41:06,163<20644>:XArm6@192.168.1.204:INFO - Finished moving robot arm to source destination: SwitchingValveVici@COM3.

2024-09-27 18:41:14,844<20644>:XArm6@192.168.1.204:INFO - Finished moving robot arm to target destination: UP200ST@192.168.233.233.

2024-09-27 18:41:15,437<14772>:UP200ST@192.168.233.233:INFO - Amplitude changed to 50.0 %.

2024-09-27 18:41:15,464<14772>:UP200ST@192.168.233.233:INFO - Power changed to 50.0 %.

2024-09-27 18:41:15,510<14772>:UP200ST@192.168.233.233:INFO - Time limit activated.

2024-09-27 18:41:15,538<14772>:UP200ST@192.168.233.233:INFO - Time limit changed to 90.0 s.

2024-09-27 18:41:15,538<14772>:UP200ST@192.168.233.233:INFO - Starting sonication...

2024-09-27 18:41:15,563<14772>:UP200ST@192.168.233.233:INFO - Ultrasound turned on.

2024-09-27 18:42:45,608<14772>:UP200ST@192.168.233.233:INFO - Ultrasound turned off.

2024-09-27 18:42:45,609<14772>:UP200ST@192.168.233.233:INFO - Finished sonication.

2024-09-27 18:42:45,609<13700>:Minerva.API.MinervaAPI:SYNTHESIS_STEP - Sonicate [FALCON_TUBE_50_ML_2363304639840]: 90.0 s; 50 % Amplitude; 50 % Power

2024-09-27 18:42:47,254<20644>:XArm6@192.168.1.204:INFO - Finished moving robot arm to source destination: UP200ST@192.168.233.233.

2024-09-27 18:42:59,870<20644>:XArm6@192.168.1.204:INFO - Finished moving robot arm to target destination: CapperDecapper@ArduinoController@COM25.

2024-09-27 18:43:00,426<23296>:CapperDecapper@ArduinoController@COM25:INFO - Closing Container...

2024-09-27 18:43:00,456<23296>:CapperDecapper@ArduinoController@COM25:INFO - Wrist turning clockwise

2024-09-27 18:43:03,544<23296>:CapperDecapper@ArduinoController@COM25:INFO - Wrist turning clockwise

2024-09-27 18:43:04,130<23296>:CapperDecapper@ArduinoController@COM25:INFO - DC Motor Current [mA]: 236.12

2024-09-27 18:43:04,211<23296>:CapperDecapper@ArduinoController@COM25:INFO - DC Motor Current [mA]: 234.78

2024-09-27 18:43:04,293<23296>:CapperDecapper@ArduinoController@COM25:INFO - DC Motor Current [mA]: 235.50

2024-09-27 18:43:04,375<23296>:CapperDecapper@ArduinoController@COM25:INFO - DC Motor Current [mA]: 227.35

2024-09-27 18:43:04,457<23296>:CapperDecapper@ArduinoController@COM25:INFO - DC Motor Current [mA]: 228.85

2024-09-27 18:43:04,539<23296>:CapperDecapper@ArduinoController@COM25:INFO - DC Motor Current [mA]: 235.25

2024-09-27 18:43:04,621<23296>:CapperDecapper@ArduinoController@COM25:INFO - DC Motor Current [mA]: 239.00

2024-09-27 18:43:04,703<23296>:CapperDecapper@ArduinoController@COM25:INFO - DC Motor Current [mA]: 251.35

2024-09-27 18:43:04,785<23296>:CapperDecapper@ArduinoController@COM25:INFO - DC Motor Current [mA]: 247.65

2024-09-27 18:43:04,866<23296>:CapperDecapper@ArduinoController@COM25:INFO - DC Motor Current [mA]: 260.80

2024-09-27 18:43:04,948<23296>:CapperDecapper@ArduinoController@COM25:INFO - DC Motor Current [mA]: 260.80

2024-09-27 18:43:05,030<23296>:CapperDecapper@ArduinoController@COM25:INFO - DC Motor Current [mA]: 266.92

2024-09-27 18:43:05,112<23296>:CapperDecapper@ArduinoController@COM25:INFO - DC Motor Current [mA]: 260.58

2024-09-27 18:43:05,194<23296>:CapperDecapper@ArduinoController@COM25:INFO - DC Motor Current [mA]: 250.47

2024-09-27 18:43:05,276<23296>:CapperDecapper@ArduinoController@COM25:INFO - DC Motor Current [mA]: 235.75

2024-09-27 18:43:05,358<23296>:CapperDecapper@ArduinoController@COM25:INFO - DC Motor Current [mA]: 229.82

2024-09-27 18:43:05,440<23296>:CapperDecapper@ArduinoController@COM25:INFO - DC Motor Current [mA]: 228.47

2024-09-27 18:43:05,522<23296>:CapperDecapper@ArduinoController@COM25:INFO - DC Motor Current [mA]: 233.35

2024-09-27 18:43:05,604<23296>:CapperDecapper@ArduinoController@COM25:INFO - DC Motor Current [mA]: 233.10

2024-09-27 18:43:05,686<23296>:CapperDecapper@ArduinoController@COM25:INFO - DC Motor Current [mA]: 232.82

2024-09-27 18:43:05,767<23296>:CapperDecapper@ArduinoController@COM25:INFO - DC Motor Current [mA]: 224.83

2024-09-27 18:43:05,849<23296>:CapperDecapper@ArduinoController@COM25:INFO - DC Motor Current [mA]: 224.20

2024-09-27 18:43:05,931<23296>:CapperDecapper@ArduinoController@COM25:INFO - DC Motor Current [mA]: 221.30

2024-09-27 18:43:06,013<23296>:CapperDecapper@ArduinoController@COM25:INFO - DC Motor Current [mA]: 227.27

2024-09-27 18:43:06,095<23296>:CapperDecapper@ArduinoController@COM25:INFO - DC Motor Current [mA]: 236.78

2024-09-27 18:43:06,177<23296>:CapperDecapper@ArduinoController@COM25:INFO - DC Motor Current [mA]: 240.80

2024-09-27 18:43:06,259<23296>:CapperDecapper@ArduinoController@COM25:INFO - DC Motor Current [mA]: 246.10

2024-09-27 18:43:06,341<23296>:CapperDecapper@ArduinoController@COM25:INFO - DC Motor Current [mA]: 264.88

2024-09-27 18:43:06,422<23296>:CapperDecapper@ArduinoController@COM25:INFO - DC Motor Current [mA]: 265.92

2024-09-27 18:43:06,504<23296>:CapperDecapper@ArduinoController@COM25:INFO - DC Motor Current [mA]: 268.67

2024-09-27 18:43:06,586<23296>:CapperDecapper@ArduinoController@COM25:INFO - DC Motor Current [mA]: 253.70

2024-09-27 18:43:06,668<23296>:CapperDecapper@ArduinoController@COM25:INFO - DC Motor Current [mA]: 249.13

2024-09-27 18:43:06,701<23296>:CapperDecapper@ArduinoController@COM25:INFO - Stopped turning wrist

2024-09-27 18:43:08,692<23296>:CapperDecapper@ArduinoController@COM25:INFO - Clamp opened.

2024-09-27 18:43:10,076<20644>:XArm6@192.168.1.204:INFO - Finished moving robot arm to source destination: CapperDecapper@ArduinoController@COM25.

2024-09-27 18:43:38,499<20644>:XArm6@192.168.1.204:INFO - Changed grip on container FALCON_TUBE_50_ML_2363304639840 to a top grip.

2024-09-27 18:43:53,514<20644>:XArm6@192.168.1.204:INFO - Finished moving robot arm to target destination: Isolab_50mL_Foldable_Tube_Rack->deck 1.

2024-09-27 18:43:54,074<13700>:Minerva.API.MinervaAPI:SYNTHESIS_STEP - Remove supernatant and redisperse [FALCON_TUBE_50_ML_2363304639840]: Chemical Ethanol: 15.0 mL

2024-09-27 18:44:02,668<20644>:XArm6@192.168.1.204:INFO - Finished moving robot arm to source destination: Isolab_50mL_Foldable_Tube_Rack->deck 1.

2024-09-27 18:44:28,497<20644>:XArm6@192.168.1.204:INFO - Changed grip on container FALCON_TUBE_50_ML_2363304640128 to a sideways grip.

2024-09-27 18:44:42,040<20644>:XArm6@192.168.1.204:INFO - Finished moving robot arm to target destination: CapperDecapper@ArduinoController@COM25.

2024-09-27 18:44:42,599<23296>:CapperDecapper@ArduinoController@COM25:INFO - Opening Container...

2024-09-27 18:44:42,695<23296>:CapperDecapper@ArduinoController@COM25:INFO - Clamp opened.

2024-09-27 18:44:47,143<23296>:CapperDecapper@ArduinoController@COM25:INFO - Clamp closed.

2024-09-27 18:44:47,175<23296>:CapperDecapper@ArduinoController@COM25:INFO - Wrist turning counterclockwise

2024-09-27 18:44:49,223<23296>:CapperDecapper@ArduinoController@COM25:INFO - Stopped turning wrist

2024-09-27 18:44:50,615<20644>:XArm6@192.168.1.204:INFO - Finished moving robot arm to source destination: CapperDecapper@ArduinoController@COM25.

2024-09-27 18:45:07,150<20644>:XArm6@192.168.1.204:INFO - Finished moving robot arm to target destination: SwitchingValveVici@COM3.

2024-09-27 18:45:07,740<13700>:SwitchingValveVici@COM3:INFO - Valve SwitchingValveVici@COM3 is currently in position 8 (connected to Outlet).

2024-09-27 18:45:07,740<13700>:SwitchingValveVici@COM3:INFO - Moved valve SwitchingValveVici@COM3 to position 8 (connected to Outlet).

2024-09-27 18:45:07,836<22688>:Aladdin@COM12:INFO - Infusion/withdrawing volume set to 15.0 mL at a rate of 70.0 mL/min

2024-09-27 18:45:21,034<22688>:Aladdin@COM12:INFO - Finished withdrawing 15.0 mL at a rate of 70.0 mL/min

2024-09-27 18:45:21,065<13700>:SwitchingValveVici@COM3:INFO - Valve SwitchingValveVici@COM3 is currently in position 8 (connected to Outlet).

2024-09-27 18:45:21,562<13700>:SwitchingValveVici@COM3:INFO - Moved valve SwitchingValveVici@COM3 to position 3 (connected to Waste_Container).

2024-09-27 18:45:21,657<22688>:Aladdin@COM12:INFO - Infusion/withdrawing volume set to 15.0 mL at a rate of 70.0 mL/min

2024-09-27 18:45:34,663<22688>:Aladdin@COM12:INFO - Finished infusing 15.0 mL at a rate of 70.0 mL/min

2024-09-27 18:45:34,664<13700>:SwitchingValveVici@COM3:INFO - Performing purging step into container Container[Waste_Container]: 2.36 L at SwitchingValveVici@COM3->slot 3...

2024-09-27 18:45:34,696<13700>:SwitchingValveVici@COM3:INFO - Valve SwitchingValveVici@COM3 is currently in position 3 (connected to Waste_Container).

2024-09-27 18:45:35,191<13700>:SwitchingValveVici@COM3:INFO - Moved valve SwitchingValveVici@COM3 to position 8 (connected to Outlet).

2024-09-27 18:45:35,287<22688>:Aladdin@COM12:INFO - Infusion/withdrawing volume set to 10.0 mL at a rate of 70.0 mL/min

2024-09-27 18:45:43,997<22688>:Aladdin@COM12:INFO - Finished withdrawing 10.0 mL at a rate of 70.0 mL/min

2024-09-27 18:45:44,029<13700>:SwitchingValveVici@COM3:INFO - Valve SwitchingValveVici@COM3 is currently in position 8 (connected to Outlet).

2024-09-27 18:45:44,537<13700>:SwitchingValveVici@COM3:INFO - Moved valve SwitchingValveVici@COM3 to position 3 (connected to Waste_Container).

2024-09-27 18:45:44,619<22688>:Aladdin@COM12:INFO - Infusion/withdrawing volume set to 10.0 mL at a rate of 70.0 mL/min

2024-09-27 18:45:53,328<22688>:Aladdin@COM12:INFO - Finished infusing 10.0 mL at a rate of 70.0 mL/min

2024-09-27 18:45:53,330<13700>:SwitchingValveVici@COM3:INFO - Performing addition step for chemical Chemical Ethanol: 15.0 mL (Ethanol_wash -> FALCON_TUBE_50_ML_2363304640128)

2024-09-27 18:45:53,361<13700>:SwitchingValveVici@COM3:INFO - Valve SwitchingValveVici@COM3 is currently in position 3 (connected to Waste_Container).

2024-09-27 18:45:53,517<13700>:SwitchingValveVici@COM3:INFO - Moved valve SwitchingValveVici@COM3 to position 2 (connected to Ethanol_wash).

2024-09-27 18:45:53,598<22688>:Aladdin@COM12:INFO - Infusion/withdrawing volume set to 15.0 mL at a rate of 70.0 mL/min

2024-09-27 18:46:06,734<22688>:Aladdin@COM12:INFO - Finished withdrawing 15.0 mL at a rate of 70.0 mL/min

2024-09-27 18:46:06,768<13700>:SwitchingValveVici@COM3:INFO - Valve SwitchingValveVici@COM3 is currently in position 2 (connected to Ethanol_wash).

2024-09-27 18:46:07,181<13700>:SwitchingValveVici@COM3:INFO - Moved valve SwitchingValveVici@COM3 to position 8 (connected to Outlet).

2024-09-27 18:46:07,277<22688>:Aladdin@COM12:INFO - Infusion/withdrawing volume set to 15.0 mL at a rate of 70.0 mL/min

2024-09-27 18:46:20,268<22688>:Aladdin@COM12:INFO - Finished infusing 15.0 mL at a rate of 70.0 mL/min

2024-09-27 18:46:20,269<13700>:SwitchingValveVici@COM3:INFO - Finished adding Chemical Ethanol: 15.0 mL (Ethanol_wash -> FALCON_TUBE_50_ML_2363304640128)

2024-09-27 18:46:21,271<13700>:SwitchingValveVici@COM3:INFO - Performing purging step into container FALCON_TUBE_50_ML: 15.0 mL at SwitchingValveVici@COM3->slot 0...

2024-09-27 18:46:22,506<13700>:SwitchingValveVici@COM3:INFO - Valve SwitchingValveVici@COM3 is currently in position 8 (connected to Outlet).

2024-09-27 18:46:22,506<13700>:SwitchingValveVici@COM3:INFO - Moved valve SwitchingValveVici@COM3 to position 8 (connected to Outlet).

2024-09-27 18:46:22,601<22688>:Aladdin@COM12:INFO - Infusion/withdrawing volume set to 30.0 mL at a rate of 70.0 mL/min

2024-09-27 18:46:48,614<22688>:Aladdin@COM12:INFO - Finished withdrawing 30.0 mL at a rate of 70.0 mL/min

2024-09-27 18:46:49,909<22688>:Aladdin@COM12:INFO - Infusion/withdrawing volume set to 30.0 mL at a rate of 70.0 mL/min

2024-09-27 18:47:15,811<22688>:Aladdin@COM12:INFO - Finished infusing 30.0 mL at a rate of 70.0 mL/min

2024-09-27 18:47:15,812<13700>:Minerva.API.MinervaAPI:SYNTHESIS_STEP - Add Chemical [FALCON_TUBE_50_ML_2363304640128]: FALCON_TUBE_50_ML; Chemical Ethanol: 15.0 mL; 70.0 mL/min; 50 mL Syringe

2024-09-27 18:47:17,323<20644>:XArm6@192.168.1.204:INFO - Finished moving robot arm to source destination: SwitchingValveVici@COM3.

2024-09-27 18:47:25,979<20644>:XArm6@192.168.1.204:INFO - Finished moving robot arm to target destination: UP200ST@192.168.233.233.

2024-09-27 18:47:26,575<14772>:UP200ST@192.168.233.233:INFO - Amplitude changed to 50.0 %.

2024-09-27 18:47:26,604<14772>:UP200ST@192.168.233.233:INFO - Power changed to 50.0 %.

2024-09-27 18:47:26,628<14772>:UP200ST@192.168.233.233:INFO - Time limit activated.

2024-09-27 18:47:26,655<14772>:UP200ST@192.168.233.233:INFO - Time limit changed to 90.0 s.

2024-09-27 18:47:26,655<14772>:UP200ST@192.168.233.233:INFO - Starting sonication...

2024-09-27 18:47:26,680<14772>:UP200ST@192.168.233.233:INFO - Ultrasound turned on.

2024-09-27 18:48:56,723<14772>:UP200ST@192.168.233.233:INFO - Ultrasound turned off.

2024-09-27 18:48:56,723<14772>:UP200ST@192.168.233.233:INFO - Finished sonication.

2024-09-27 18:48:56,724<13700>:Minerva.API.MinervaAPI:SYNTHESIS_STEP - Sonicate [FALCON_TUBE_50_ML_2363304640128]: 90.0 s; 50 % Amplitude; 50 % Power

2024-09-27 18:48:59,901<20644>:XArm6@192.168.1.204:INFO - Finished moving robot arm to source destination: UP200ST@192.168.233.233.

2024-09-27 18:49:11,416<20644>:XArm6@192.168.1.204:INFO - Finished moving robot arm to target destination: CapperDecapper@ArduinoController@COM25.

2024-09-27 18:49:11,969<23296>:CapperDecapper@ArduinoController@COM25:INFO - Closing Container...

2024-09-27 18:49:12,002<23296>:CapperDecapper@ArduinoController@COM25:INFO - Wrist turning clockwise

2024-09-27 18:49:15,082<23296>:CapperDecapper@ArduinoController@COM25:INFO - Wrist turning clockwise

2024-09-27 18:49:15,672<23296>:CapperDecapper@ArduinoController@COM25:INFO - DC Motor Current [mA]: 231.83

2024-09-27 18:49:15,754<23296>:CapperDecapper@ArduinoController@COM25:INFO - DC Motor Current [mA]: 226.70

2024-09-27 18:49:15,836<23296>:CapperDecapper@ArduinoController@COM25:INFO - DC Motor Current [mA]: 227.33

2024-09-27 18:49:15,918<23296>:CapperDecapper@ArduinoController@COM25:INFO - DC Motor Current [mA]: 225.52

2024-09-27 18:49:16,000<23296>:CapperDecapper@ArduinoController@COM25:INFO - DC Motor Current [mA]: 224.70

2024-09-27 18:49:16,082<23296>:CapperDecapper@ArduinoController@COM25:INFO - DC Motor Current [mA]: 227.60

2024-09-27 18:49:16,164<23296>:CapperDecapper@ArduinoController@COM25:INFO - DC Motor Current [mA]: 234.40

2024-09-27 18:49:16,246<23296>:CapperDecapper@ArduinoController@COM25:INFO - DC Motor Current [mA]: 233.90

2024-09-27 18:49:16,328<23296>:CapperDecapper@ArduinoController@COM25:INFO - DC Motor Current [mA]: 242.88

2024-09-27 18:49:16,410<23296>:CapperDecapper@ArduinoController@COM25:INFO - DC Motor Current [mA]: 248.05

2024-09-27 18:49:16,492<23296>:CapperDecapper@ArduinoController@COM25:INFO - DC Motor Current [mA]: 248.60

2024-09-27 18:49:16,573<23296>:CapperDecapper@ArduinoController@COM25:INFO - DC Motor Current [mA]: 266.95

2024-09-27 18:49:16,655<23296>:CapperDecapper@ArduinoController@COM25:INFO - DC Motor Current [mA]: 273.05

2024-09-27 18:49:16,737<23296>:CapperDecapper@ArduinoController@COM25:INFO - DC Motor Current [mA]: 266.65

2024-09-27 18:49:16,819<23296>:CapperDecapper@ArduinoController@COM25:INFO - DC Motor Current [mA]: 260.25

2024-09-27 18:49:16,901<23296>:CapperDecapper@ArduinoController@COM25:INFO - DC Motor Current [mA]: 246.80

2024-09-27 18:49:16,983<23296>:CapperDecapper@ArduinoController@COM25:INFO - DC Motor Current [mA]: 235.00

2024-09-27 18:49:17,065<23296>:CapperDecapper@ArduinoController@COM25:INFO - DC Motor Current [mA]: 229.10

2024-09-27 18:49:17,146<23296>:CapperDecapper@ArduinoController@COM25:INFO - DC Motor Current [mA]: 230.85

2024-09-27 18:49:17,229<23296>:CapperDecapper@ArduinoController@COM25:INFO - DC Motor Current [mA]: 233.38

2024-09-27 18:49:17,310<23296>:CapperDecapper@ArduinoController@COM25:INFO - DC Motor Current [mA]: 228.32

2024-09-27 18:49:17,392<23296>:CapperDecapper@ArduinoController@COM25:INFO - DC Motor Current [mA]: 226.13

2024-09-27 18:49:17,474<23296>:CapperDecapper@ArduinoController@COM25:INFO - DC Motor Current [mA]: 227.92

2024-09-27 18:49:17,556<23296>:CapperDecapper@ArduinoController@COM25:INFO - DC Motor Current [mA]: 220.88

2024-09-27 18:49:17,639<23296>:CapperDecapper@ArduinoController@COM25:INFO - DC Motor Current [mA]: 224.13

2024-09-27 18:49:17,720<23296>:CapperDecapper@ArduinoController@COM25:INFO - DC Motor Current [mA]: 233.35

2024-09-27 18:49:17,802<23296>:CapperDecapper@ArduinoController@COM25:INFO - DC Motor Current [mA]: 240.08

2024-09-27 18:49:17,884<23296>:CapperDecapper@ArduinoController@COM25:INFO - DC Motor Current [mA]: 242.92

2024-09-27 18:49:17,966<23296>:CapperDecapper@ArduinoController@COM25:INFO - DC Motor Current [mA]: 245.70

2024-09-27 18:49:18,048<23296>:CapperDecapper@ArduinoController@COM25:INFO - DC Motor Current [mA]: 260.42

2024-09-27 18:49:18,130<23296>:CapperDecapper@ArduinoController@COM25:INFO - DC Motor Current [mA]: 270.47

2024-09-27 18:49:18,212<23296>:CapperDecapper@ArduinoController@COM25:INFO - DC Motor Current [mA]: 263.42

2024-09-27 18:49:18,245<23296>:CapperDecapper@ArduinoController@COM25:INFO - Stopped turning wrist

2024-09-27 18:49:20,234<23296>:CapperDecapper@ArduinoController@COM25:INFO - Clamp opened.

2024-09-27 18:49:21,623<20644>:XArm6@192.168.1.204:INFO - Finished moving robot arm to source destination: CapperDecapper@ArduinoController@COM25.

2024-09-27 18:49:49,999<20644>:XArm6@192.168.1.204:INFO - Changed grip on container FALCON_TUBE_50_ML_2363304640128 to a top grip.

2024-09-27 18:50:06,564<20644>:XArm6@192.168.1.204:INFO - Finished moving robot arm to target destination: Isolab_50mL_Foldable_Tube_Rack->deck 1.

2024-09-27 18:50:15,699<20644>:XArm6@192.168.1.204:INFO - Finished moving robot arm to source destination: Isolab_50mL_Foldable_Tube_Rack->deck 1.

2024-09-27 18:50:41,074<20644>:XArm6@192.168.1.204:INFO - Changed grip on container FALCON_TUBE_50_ML_2363304718592 to a sideways grip.

2024-09-27 18:50:46,643<20644>:XArm6@192.168.1.204:INFO - Finished moving robot arm to target destination: UP200ST@192.168.233.233.

2024-09-27 18:50:47,234<14772>:UP200ST@192.168.233.233:INFO - Amplitude changed to 50.0 %.

2024-09-27 18:50:47,263<14772>:UP200ST@192.168.233.233:INFO - Power changed to 50.0 %.

2024-09-27 18:50:47,288<14772>:UP200ST@192.168.233.233:INFO - Time limit activated.

2024-09-27 18:50:47,317<14772>:UP200ST@192.168.233.233:INFO - Time limit changed to 10.0 s.

2024-09-27 18:50:47,318<14772>:UP200ST@192.168.233.233:INFO - Starting sonication...

2024-09-27 18:50:47,343<14772>:UP200ST@192.168.233.233:INFO - Ultrasound turned on.

2024-09-27 18:50:57,373<14772>:UP200ST@192.168.233.233:INFO - Ultrasound turned off.

2024-09-27 18:50:57,374<14772>:UP200ST@192.168.233.233:INFO - Finished sonication.

2024-09-27 18:50:59,077<20644>:XArm6@192.168.1.204:INFO - Finished moving robot arm to source destination: UP200ST@192.168.233.233.

2024-09-27 18:51:18,932<20644>:XArm6@192.168.1.204:INFO - Changed grip on container FALCON_TUBE_50_ML_2363304718592 to a top grip.

2024-09-27 18:51:35,249<20644>:XArm6@192.168.1.204:INFO - Finished moving robot arm to target destination: Isolab_50mL_Foldable_Tube_Rack->deck 1.

2024-09-27 18:51:35,799<13700>:Minerva.API.MinervaAPI:SYNTHESIS_STEP - Sonicate [FALCON_TUBE_50_ML_2363304718592]: 10.0 s; 50 % Amplitude; 50 % Power

2024-09-27 18:51:35,800<13700>:Minerva.API.MinervaAPI:SYNTHESIS_STEP - Remove supernatant and redisperse [FALCON_TUBE_50_ML_2363304640128]: Chemical Ethanol: 15.0 mL

2024-09-27 18:51:38,785<13700>:RobotCen@COM8:INFO - Closed RobotCen Lid.
